# Pan-Cancer Analyses of the Tumor Microenvironment Reveal That Ubiquitin-Conjugating Enzyme E2C Might Be a Potential Immunotherapy Target

**DOI:** 10.1155/2021/9250207

**Published:** 2021-12-13

**Authors:** Guang-zhao Huang, Ze-qun Chen, Juan Wu, Ting-ru Shao, Chen Zou, Yi-long Ai, Xiao-zhi Lv

**Affiliations:** ^1^Department of Oral & Maxillofacial Surgery, Nanfang Hospital, Southern Medical University, Guangzhou 510080, China; ^2^Department of Gastrointestinal Surgery, Maoming People's Hospital, Maoming 525000, China; ^3^Department of Anesthesiology, The First People's Hospital of Shuangliu District, 610000, China; ^4^Foshan Stomatological Hospital, School of Stomatology and Medicine, Foshan University, Foshan 510080, China

## Abstract

Increasing evidence indicated that the tumor microenvironment (TME) played a crucial role in cancer initiation and progression. Ubiquitin-conjugating enzyme E2C (UBE2C) was differentially expressed in many cancer types. However, the immunological and prognostic roles of UBE2C were unclear. Differentially expressed genes (DEGs) of 29 cancer types were downloaded from GEPIA2 and 4 cancer types failed to download owing to no DEGs. Furthermore, the gene expression profiles, mutation data, and survival data of 33 cancer types were obtained from UCSC Xena. Clinical stage relevance, tumor mutational burden (TMB), TME relevance analysis, and gene set enrichment analysis (GSEA) of DEGs in 33 cancer types were performed. And DEGs were identified in oral squamous cell carcinoma (OSCC) by biological experiments. Previous studies indicated that UBE2C was related to the prognosis of many cancers. In our study, the higher UBE2C expression level meant a terminal clinical stage in 8 cancer types and the expression level of UBE2C was related to TMB in 20 cancer types. In addition, both immune relevance analysis and GSEA showed that UBE2C might participate in immune response in many cancers. Furthermore, the UBE2C mRNA level and protein level were all identified as upregulated in OSCC cell lines and tissues. UBE2C was differentially expressed in many cancer types and related to the pathogenesis and TME of many cancers, which might be a potential diagnostic and therapeutic biomarker.

## 1. Introduction

Cancers are severe public health problems all over the world and one of the most common leading causes of death. Annual cases are only expected to increase owing to the aging population [1]. A study showed that an estimated 1,762,450 new cancer cases and 606,880 cancer deaths occurred in the United States in 2019 [2]. In China, there were approximately 4.3 million new cancer cases and 2.9 million new cancer deaths in 2018 [3]. With the increase of various cancer morbidity, it is imperious to understand the potential mechanism of tumorigenesis and tumor progression and identify effective biomarkers for cancer diagnosis and therapy. For the past 20 years, studies have indicated that inflammatory immune cells played a crucial role in cancer-related inflammation [4]. For example, Coussens and Werb identified that during tumor formation, the tissue structure evolved into a highly specialized microenvironment characterized by chronic inflammation [5]. Hanahan and Weinberg revealed that tumor-associated inflammation exists in different stages of tumorigenesis and contributes to genomic instability, epigenetic modification, induction of cancer cell proliferation, enhancement of tumor antiapoptotic pathways, stimulation of angiogenesis, and ultimately tumor dissemination [6]. In addition, patients with *Helicobacter pylori* were more likely to develop gastric cancer [7]. *Human papillomavirus* (HPV) infection was associated with head and neck cancer tumorigeneses [8]. Immune dysregulation also increased colorectal cancer incidence [9]. Therefore, the immune system may be relevant to tumorigenesis and tumor progression significantly. Surgery, radiotherapy, and chemotherapy were conventional strategies in the treatment of cancers. In recent years, accumulating studies have shown that immunotherapy is also a targeted systemic option [10–12]. For example, nivolumab may restore antitumor immunity via disrupting PD-1-mediated signaling. Studies also indicated that nivolumab played a crucial role in the treatment of nonsquamous non-small-cell lung cancer and melanoma [12, 13]. In addition, immunotherapies on the basis of PD-1/PDL-1 showed promising clinical responses in hepatocellular carcinoma [14] and lung cancer [15]. Therefore, looking for effective immunotherapeutic targets may contribute to cancer therapy.

Ubiquitin-conjugating enzyme E2C (UBE2C), a crucial member of the E2 family, is involved in the cell cycle process through interacting with the late-promoting complex/annular port (APC/C) and identified as a novel potential tumor biomarker [16]. UBE2C is an integral component of the ubiquitin proteasome system. Studies showed that the expression levels of UBE2C were dysregulated in many cancers with unsatisfied clinical outcomes. For instance, activation of the oncogene UBE2C was concurrently underlying the initiation, progression, and metastasis of lung cancer [17]. Mechanism analysis showed that UBE2C played a significant role in the development and progression of pancreatic ductal adenocarcinoma (PDAC) by regulating cell proliferation and EMT. UBE2C was identified as a novel potential therapeutic target for pancreatic cancer [18]. Recently, increasing evidences demonstrated that immune cell infiltration and the tumor microenvironment (TME) were associated with cancer initiation and progression [6]. A study have showed that UBE2C was related to the prognosis of many cancers [19]. However, the relevance between UBE2C and immune cell infiltration and TME was still unclear.

In our study, differentially expressed genes were screened from GEPIA 2 database (http://gepia2.cancer-pku.cn/#degenes) and UBE2C was identified to be differentially expressed across cancers. Gene expression, mutation, and survival data of 33 cancer types were downloaded from UCSC Xena (https://xena.ucsc.edu/). The purpose of this study was to systematically analyze the clinical relevance and tumor immune microenvironment of UBE2C across 33 cancers. Then, clinical stage and TME relevance analyses were performed. Finally, GSEA was used to annotate the potential function of UBE2C across 33 cancers.

## 2. Materials and Methods

### 2.1. Differentially Expressed Gene Acquisition

29 cancer-type differentially expressed genes (DEGs) were downloaded from GEPIA 2 database (http://gepia2.cancer-pku.cn/#degenes) with the cutoff criteria ∣log2(fold change) | >1 and *q* value < 0.01, and LIMMA was selected as a differential method. PCPG, SARC, MESO, and UVM failed to download owing to no DEGs. And then, 33 cancer-type gene expression data, mutation data, and survival data were further obtained from UCSC Xena (https://xena.ucsc.edu/).

### 2.2. Univariate Cox Regression Analysis

Overall survival (OS), disease-specific survival (DSS), disease-free interval (DFI), and progression-free interval (PFI) data were gained from UCSC Xena (https://xena.ucsc.edu/) TCGA pan-cancer. Univariate cox regression analyses according to OS, DSS, DFI, and PFI were used to explore the prognostic value of UBE2C in 33 cancer types.

### 2.3. PPI Network Construction and GO and KEGG Enrichment Analysis

The protein-protein interaction network was constructed in STRING database (https://www.string-db.org/). UBE2C was imported to STRING database to explore the mutual regulation relationship between other genes, and the cutoff of confidence was 0.9. And the top 20 proteins were listed. Furthermore, the proteins enrolled in the PPI network were performed to Gene Ontology (GO), biological processes (BP), cellular component (CC), molecular function (MF), and Kyoto Encyclopedia of Genes and Genomes (KEGG) pathway analysis in DAVID database (https://david.ncifcrf.gov/).

### 2.4. Clinical Stage Relevance Analysis

Clinical stage data were also obtained from UCSC Xena (https://xena.ucsc.edu/) TCGA pan-cancer (PANCAN). According to clinical stage, the expression level of UBE2C was compared between stage I + II and stage III + IV with the aim at exploring UBE2C expression in the early stage and terminal stage in 33 cancer types.

### 2.5. Tumor Mutational Burden TMB) Relevance Analysis

TMB is defined as the number of mutations that exist within a tumor and related to the emergence of neoantigens that trigger antitumor immunity. In addition, TMB is also regarded as a new biomarker for prediction of response to immunotherapy [20]. Therefore, TMB data were extracted from mutation files which were downloaded from UCSC Xena (https://xena.ucsc.edu/) and the prognostic value of TMB was explored across 33 cancer types. In addition, relevance analyses between TMB and UBE2C expression levels were also performed.

### 2.6. TME Relevance Analysis

TME cells consist of a crucial element of tumor tissues. Accumulating evidences demonstrated their clinicopathologic significance in predicting outcomes and therapeutic efficacy [21, 22]. Therefore, the relevance between UBE2C and TME was explored in our study. The “ESTIMATE” package in R software was used to calculate the “immune” and “stromal” scores in 33 cancer types [23]. And the “CIBERSORT” algorithm was performed to assess the 22 immune cell infiltration [23] including naive B cells, memory B cells, plasma cells, CD8 T cells, naive CD4 T cells, resting memory CD4 T cells, memory-activated CD4 T cells, follicular helper T cells, T regulatory cells (Tregs), gamma delta T cells, resting NK cells, activated NK cells, monocytes, M0 macrophages, M1 macrophages, M2 macrophages, resting dendritic cells, activated dendritic cells, resting mast cells, activated mast cells, eosinophils, and neutrophils in 33 cancer types. Finally, the correlation analyses of “immune” and “stromal” scores and immune cell infiltration and UBE2C expression levels were explored in R software.

### 2.7. Coexpression Analysis

According to gene expression profiles in 33 cancer types, the gene expression levels of immune markers were extracted based on previous research [24–26]. Spearman's correlation analysis was performed to explore the relevance between UBE2C and these immune-related genes to reveal the potential function of UBE2C in the immune process. And the *p* value and Spearman's correlation coefficient were calculated. And a heat map was plotted.

### 2.8. Gene Set Enrichment Analysis (GSEA)

Gene set enrichment analysis (GSEA) derives its power by focusing on gene sets, that is, groups of genes that share common biological function, chromosomal location, and regulation. GSEA was used to investigate the effect of UBE2C on gene enrichment across 33 cancer types in GSEA (version 4.1.0) software.

### 2.9. Cell Culture

The normal oral epithelial cell line human oral keratinocytes (HOK) and OSCC cell lines SCC9, SCC25, and CAL27 were obtained from the Institute of Antibody Engineering, Southern Medical University (Guangzhou, China). Cell lines HOK, SCC25, and CAL27 were seeded in DMEM and SCC9 in DMEM/F12 containing 10% foetal bovine serum (FBS) (ExCell Bio Inc.) and incubated at 37°C with 5% CO_2_.

### 2.10. Collection of OSCC Tissue Samples

40 cases of OSCC tissue samples and adjacent normal tissues (ANTs) were collected from Nanfang Hospital, Southern Medical University. Respectively, all tumor specimens and ANTs were confirmed as squamous cell carcinoma and normal tissues pathologically. The Nanfang Hospital ethics committee (AF/SC-09/03.2) approved this study.

### 2.11. RNA Extraction and Quantitative Real-Time PCR (qRT-PCR)

Total RNAs of cell lines and sample tissues were extracted with TRIzol reagent (TaKaRa, Cat# 9109), and the same amount of total RNAs was reversed to cDNA on the basis of the Reverse Transcription Kit according to manufacturer's protocol (Vazyme). QRT-PCR assay was used to detect the UBE2C mRNA level with ChamQ Universal SYBR qPCR Master Mix (Vazyme Biotech Co. Ltd.). The sequences of the PCR primers were as follows: UBE2C, forward 5′-GACCTGAGGTATAAGCTCTCGC-3′ and reverse 5′-TTACCCTGGGTGTCCACGTT-3′; GAPDH, forward 5′-CGCTGA GTACGTCGTGGAGTC-3′ and reverse 5′-GCTGATGATCTT GAGGCTGTTGTC-3′.

### 2.12. Western Blot Assay

The protein of OSCC cell lines and 3 pairs of tissues were extracted by RIPA lysis buffer. Polyacrylamide gel electrophoresis (PAGE) was used to separate protein samples. Furthermore, proteins were transferred to PVDF membranes and then sealed with 5% skim milk. Subsequently, primary antibodies were incubated at 4°C overnight and second antibodies were incubated at room temperature for 1 hour. Finally, the protein level was quantified by ECL. The antibody information was as follows: UBE2C (ABclonal, 1 : 1000), GAPDH (ProteinTech, 1 : 5000), and goat anti-rabbit (ProteinTech, 1 : 10 000).

### 2.13. Immunohistochemistry

Sample tissues collected from Nanfang Hospital were fixed with 4% formaldehyde, dehydrated, immersed in wax, embedded in paraffin, and cut into 4 *μ*m sections. All tissue sections were dewaxed with xylene and rehydrated in graded ethanol including 100%, 95%, 90%, 80%, 70%, and 50%. Then, endogenous peroxidase was blocked by 3% hydrogen peroxide for 10 mins. Antigen retrieval assay was performed in a pressure cooker for 10–12 minutes with a 0.01 M citrate buffer (pH 6.0). All tissue sections were sealed with 5% BSA. Subsequently, UBE2C antibodies (ABclonal,1 : 100) were incubated at 4°C overnight and secondary antibody was incubated at room temperature for 1 hour. Finally, the sections were visualized with 3,3′-diaminobenzidine (DAB). The staining extent was scored from 4 to 0 (76–100%, 26%–5%, 6%–25%, 1%–5%, and 0%). The staining intensity was scored as strong (score = 2), weak (score = 1), or negative (score = 0). A score ranging from 0 to 8 was calculated by multiplying the staining extent and intensity [27].

## 3. Results

### 3.1. UBE2C Is Differentially Expressed in Many Cancer Types

**Figure 1:**
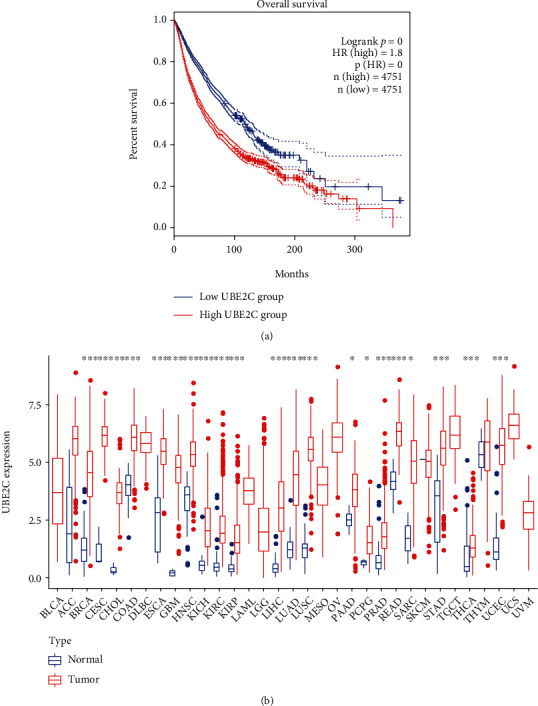
UBE2C was differentially expressed in many cancer types. (a) UBE2C overall survival analysis in 33 cancer types. The high UBE2C expression level was related to poor prognosis. (b) Differentially expressed analysis in 33 cancer types. UBE2C was differentially expressed in 24 cancer types. Owing to no normal controls, differential expression was not performed in 9 cancer types including ACC, DLBC, LAML, LGG, MESO, OV, TGCT, UCS, and UVM. ^∗^*p* < 0.5, ^∗∗^*p* < 0.01, and ^∗∗∗^*p* < 0.001.

By comparison, UBE2C was the only differentially expressed gene in many cancer types (Table 1) according to the GEPIA 2 database. 4 cancer types including PCPG, SARC, MESO, and UVM failed to download owing to no DEGs in GEPIA 2. Survival analysis indicated that patients with a low UBE2C expression level had better prognosis (Figure 1(a)). In addition, Dastsooz et al. explored the prognostic value of UBE2C in various cancers [28]. In our study, univariate Cox regression analysis in 33 cancer types also showed that UBE2C might be a prognostic biomarker in many cancers according to OS, DSS, DFI, and PFI (Figure S1). Furthermore, gene expression profiles were downloaded from UCSC Xena. Differentially expressed analysis indicated that UBE2C was differentially expressed in many types (Figure 1(b)). The *p* value of differential analysis was presented in Supplementary Table 1. There were 9 cancer types including ACC, DLBC, LAML, LGG, MESO, OV, TGCT, UCS, and UVM which were excluded owing to no normal control samples in TCGA database. In addition, SKCM was not performed differentially expressed analysis owing to no normal sample. And THYM had no significance between normal and tumor samples because of high standard deviation. However, UBE2C was upregulated in THYM according to GEPIA 2 database. To sum up, UBE2C was identified to be differentially expressed in many cancer types.

### 3.2. Functional Analysis of Proteins Enrolled in the PPI Network

**Figure 2:**
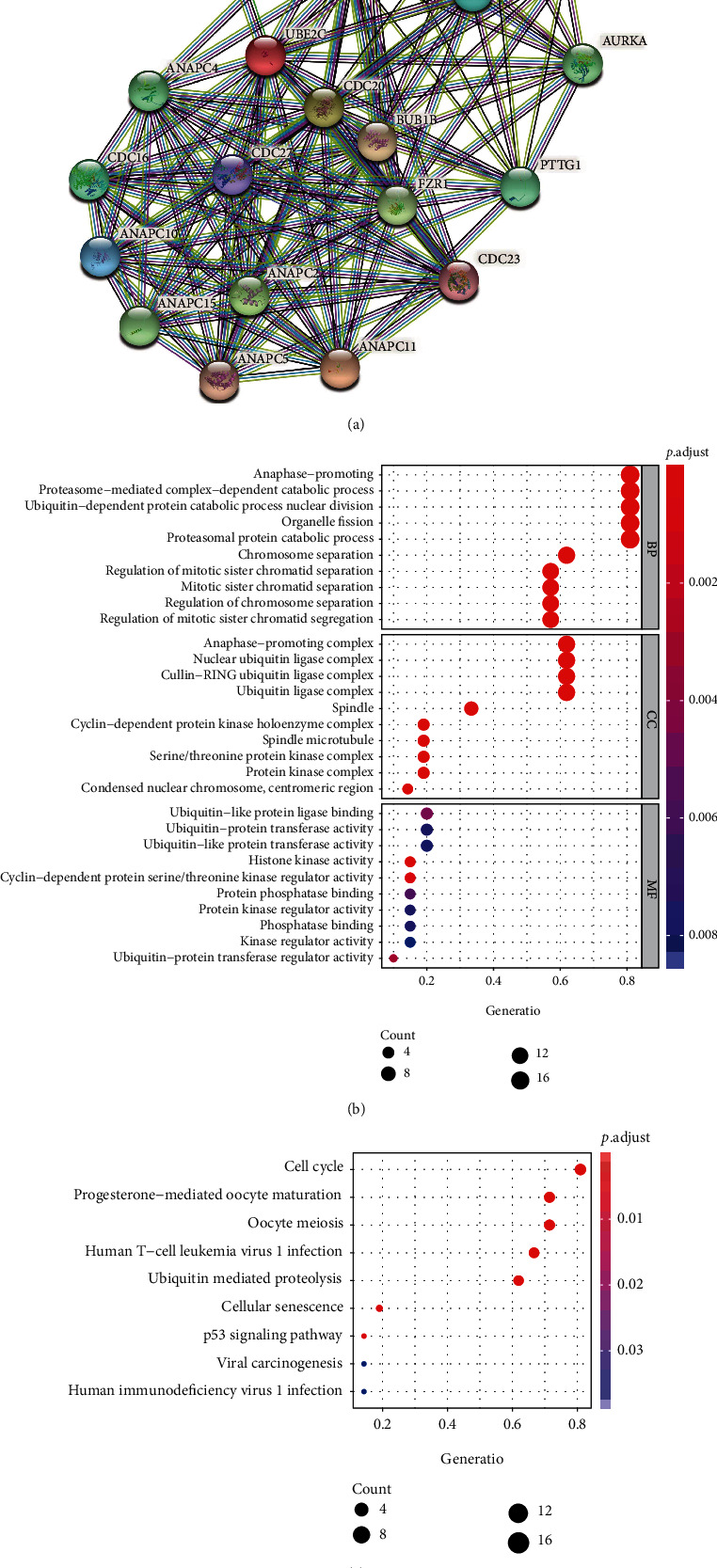
GO and KEGG enrichment analysis of proteins enrolled in the protein-protein interaction network. (a) The PPI network was constructed in STRING according to UBE2C, and the top 20 proteins were listed. (b) GO enrichment analysis according to proteins enrolled in PPI. (c) KEGG pathway enrichment analysis according to proteins enrolled in PPI.

The top 20 molecules interacting with UBE2C were listed (Figure 2(a)). Furthermore, GO enrichment analysis indicated that these genes were mainly relevant to the proteasome-mediated ubiquitin-dependent protein catabolic process in BP, ubiquitin ligase complex in CC, and histone kinase activity in MF (Figure 2(b)). In addition, KEGG pathway analysis showed that most of proteins enrolled in the PPI network are enriched in the cell cycle and p53 signaling pathway which were associated with cancer development significantly (Figure 2(c)).

### 3.3. High UBE2C Level Was Related to the Terminal Clinical Stage in Several Cancer Types

**Figure 3:**
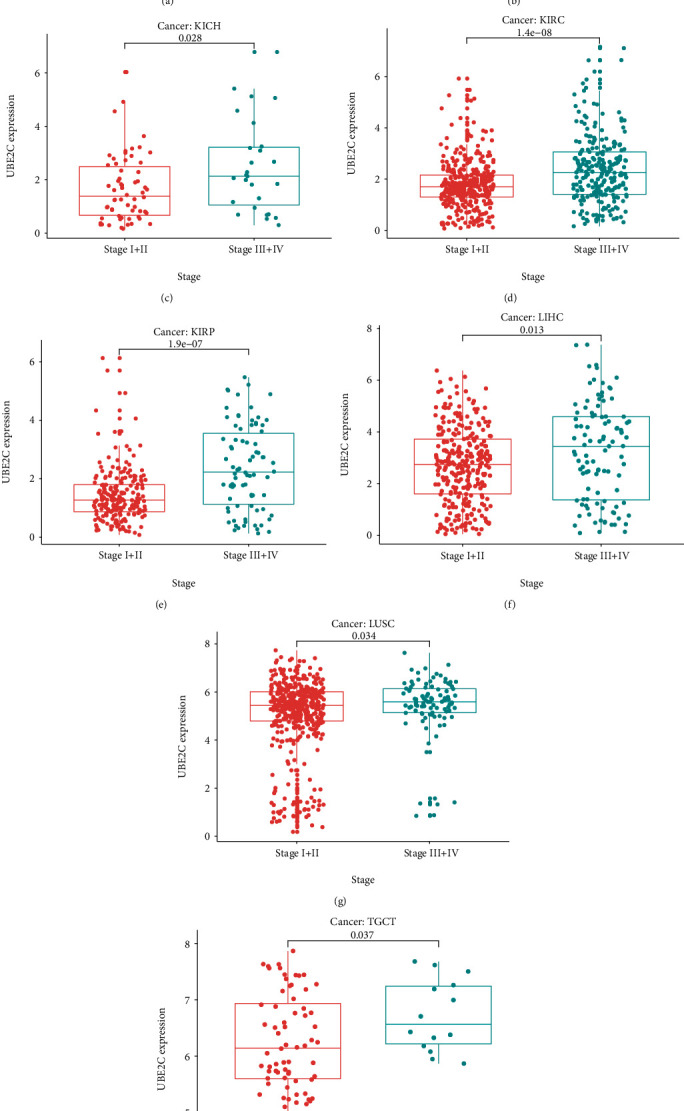
Clinical stage analysis of UBE2C. The high UBE2C expression level meant a terminal clinical stage in ACC (a), HNSC (b), KICH (c), KIRC (d), KIRP (e), LIHC (f), LUSC (g), and TGCT (h).

Compared early clinical stage I + II to III + IV, the UBE2C expression level was significantly increased in the terminal stage in 8 cancer types including ACC (Figure 3(a)), HNSC (Figure 3(b)), KICH (Figure 3(c)), KIRC (Figure 3(d)), KIRP (Figure 3(e)), LIHC (Figure 3(f)), LUSC (Figure 3(g)), and TGCT (Figure 3(h)) indicating that UBE2C might be a terminal biomarker in these cancer types.

### 3.4. Prognostic Value Analyses of TMB

**Figure 4:**
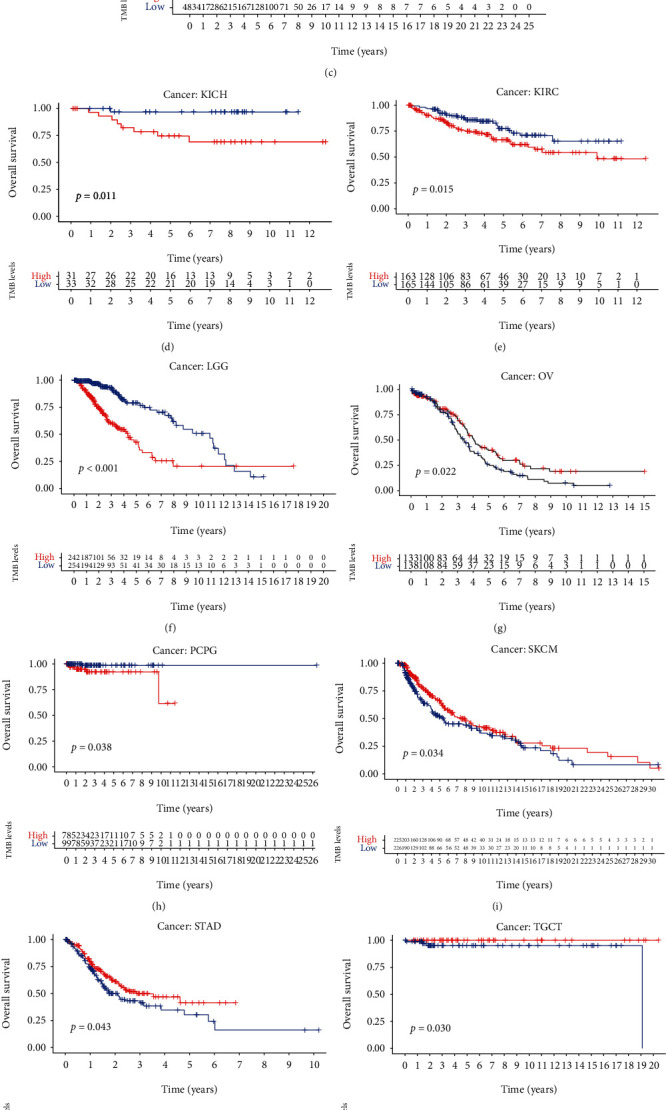
Overall survival of 13 cancer types including ACC, BLCA, BRCA, KICH, KIRC, LGG, OV, PCPG, SKCM, STAD, TGCT, THCA, and THYM according to tumor mutation burden (TMB).

According to survival data and the TMB level of each patient, TMB was associated with the survival across 13 cancer types including ACC, BLCA, BRCA, KICH, KIRC, LGG, OV, PCPG, SKCM, STAD, TGCT, THCA, and THYM. Among them, the low TMB level means poor prognosis in BLCA, SKCM, STAD, TGCT, and OV. And in the other 8 cancer types, the lower the TMB level, the better the prognosis in patients (Figures 4(a)–4(m)).

### 3.5. UBE2C Was Associated with TMB and Immune Markers

**Figure 5:**
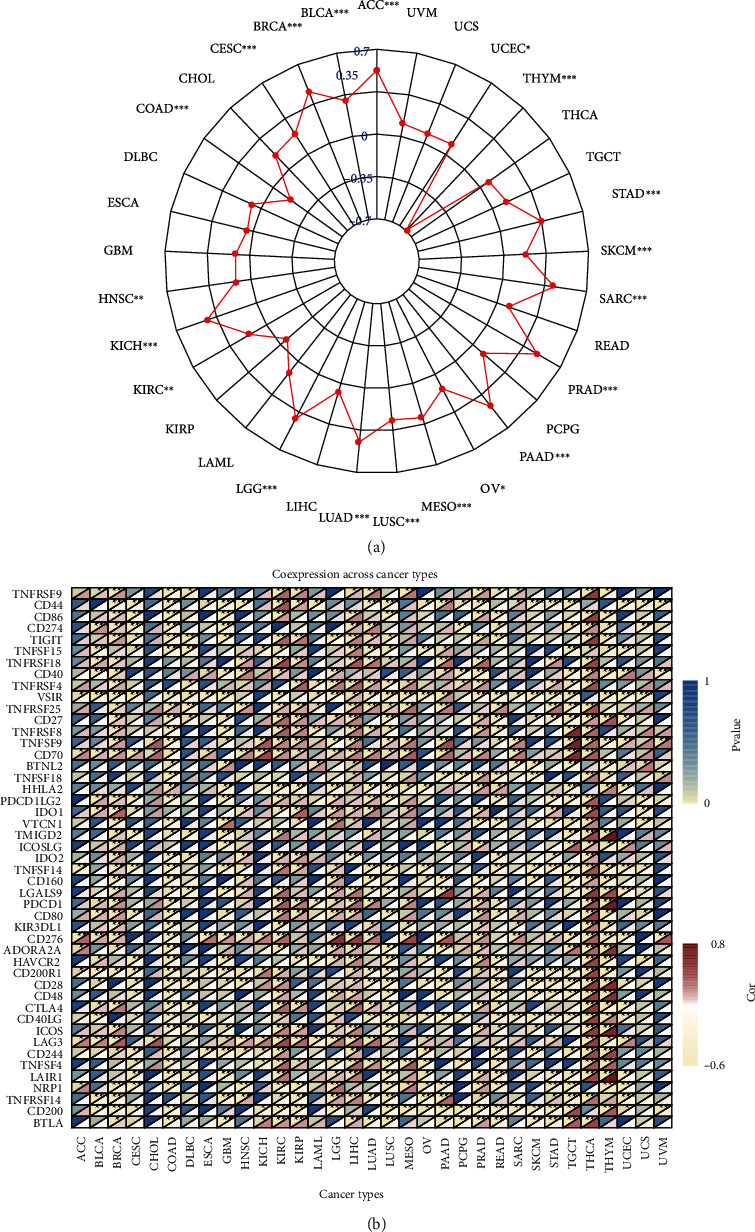
Tumor mutational burden (TMB) relevance analysis. (a) UBE2C was associated with the TMB of many cancers including BRCA, PRAD, LUAD, LGG, THYM, STAD, SARC, BLCA, PAAD, LUSC, ACC, SKCM, KICH, COAD, CESC, KIRC, HNSC, MESO, UCEC, and OV. Each gray circle represents the value of the correlation coefficient from −0.7–0.7, and each red spot represents the correlation coefficient between UBE2C and TMB. (b) The heat map of correlation analysis between UBE2C and many immune markers. Blue represents the *p* value, while red stands for Spearman's correlation coefficient.

According to TMB data and the UBE2C expression level of each patient, relevance analysis in each cancer type was performed (Table 2). Then, the radar map of TMB was plotted (Figure 5(a)) according to relevance analysis. The results showed that UBE2C was related to the TMB of 20 cancer types including BRCA, PRAD, LUAD, LGG, THYM, STAD, SARC, BLCA, PAAD, LUSC, ACC, SKCM, KICH, COAD, CESC, KIRC, HNSC, MESO, UCEC, and OV. Each gray circle represents the value of the correlation coefficient from −0.7–0.7, and each red spot represents the correlation coefficient between UBE2C and TMB. As shown in Figure 5(a), among the 20 cancer types, UBE2C has only negative associations with THYM and COAD. UBE2C had the strongest correlation with THYM (coefficient = −0.6955). And in positive correlation, UBE2C had the strongest correlation with ACC (coefficient = 0.5267). Furthermore, the coexpression analysis between UBE2C and the immune marker was performed. According to gene expression profiles in 33 cancer types, UBE2C were related to many immune markers, such as CD200 which is expressed by various cell types, including B cells, a subset of T cells, and thymocytes. And the encoded protein played a significant role in immunosuppression and regulation of antitumor activity. In addition, UBE2C was correlated with CD276 in many cancers. And CD276 was thought to participate in the regulation of T cell-mediated immune response indicating that UBE2C also played a crucial role in immune response. All of the results were shown in Figure 5(b). And the Spearman's correlation coefficient and *p* value were presented in supplementary Table 2 and supplementary Table 3.

### 3.6. TME Relevance Analysis

**Figure 6:**
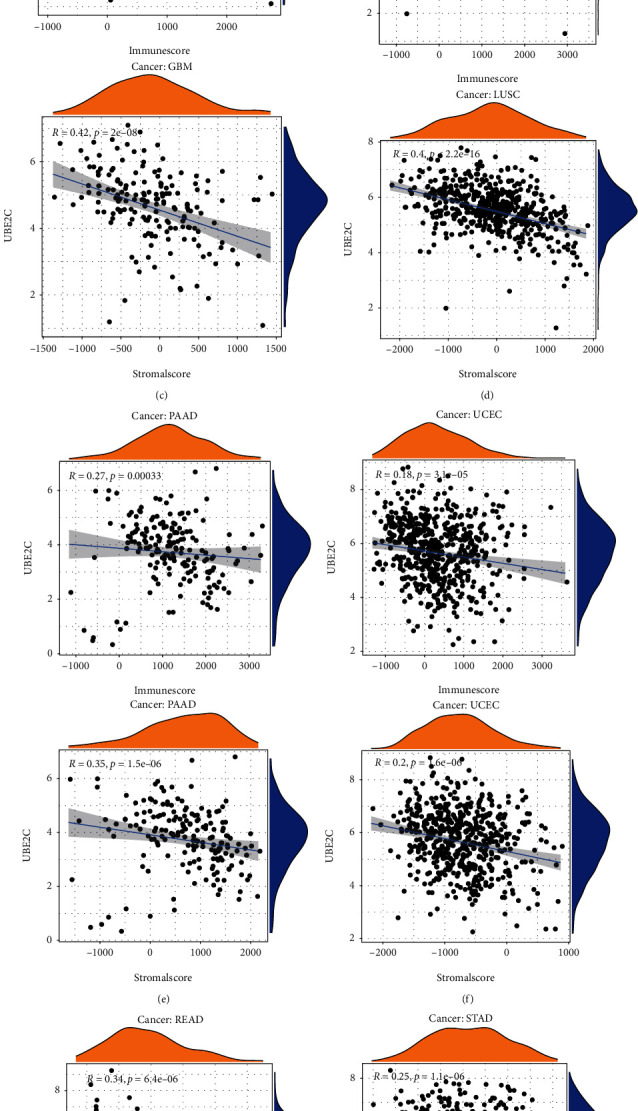
Representative results of StromalScore and ImmuneScore relevance analysis. A-H, CESE, COAD, and GBM are the examples presented.

The StromalScore, ImmuneScore, and 22 immune cell levels of each patient were calculated in R software. According to the UBE2C expression level, the relevance between StromalScore, ImmuneScore, and UBE2C were explored (Table 3). The results showed that the higher UBE2C expression level, the lower the StromalScore and ImmuneScore in CESC (Figure 6(a)), COAD (Figure 6(b)), GBM (Figure 6(c)), LUSC (Figure 6(d)), PAAD (Figure 6(e)), READ (Figure 6(f)), STAD (Figure 6(g)), and UCEC (Figure 6(h)). In addition, UBE2C was also related to the StromalScore in BRCA, HNSC, LIHC, LUAD, TGCT, THYM, and ImmuneScore in KIRC (Table 3). BRCA (Figure 6(i)) and HNSC (Figure 6(j)) were presented as examples. And the other results of StromalScore and ImmuneScore relevance analysis were displayed in supplementary file1. In these cancers, the high UBE2C expression level meant high ImmuneScore only in KIRC. Furthermore, the relationship between UBE2C expression and 22 immune cells was also explored in R software on the basis of 22 immune cell levels across 33 cancer types and the *p* values were presented in Supplementary Table 4. Majority of cancer types were associated with dysregulation of immune cell levels. For instance, in STAD, PAAD, BRCA, SARC, and LIHC, the higher UBE2C expression level meant lower B cell naïve level. In addition, about 20 cancer types were relevant to resting memory CD4 T cells, such as KIRC which UBE2C, and resting memory CD4 T cells were negatively correlated. At last, all relevance analysis results were visualized in R software (supplementary file2).

### 3.7. Gene Set Enrichment Analysis

**Figure 7:**
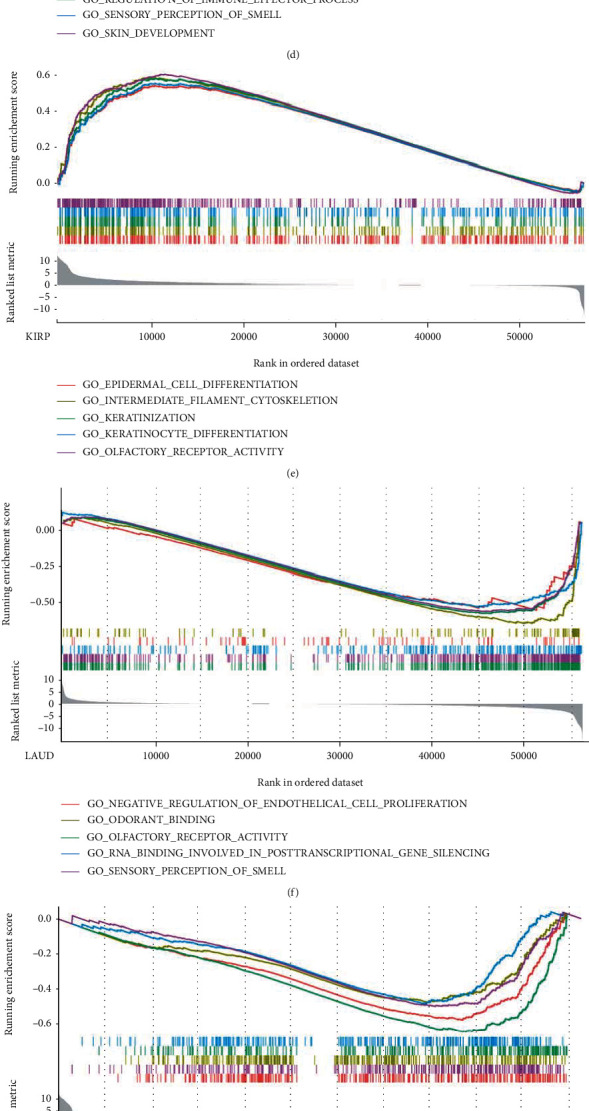
Representative results of GSEA. (a–j) The GSEA results of 10 cancer types including BRCA, CHOL, HNSC, KIRC, KIRP, LUAD, PAAD, SKCM, THCA, and UCEC.

GSEA was performed with the purpose of exploring the potential function of UBE2C in 33 cancer types. The results showed that UBE2C was related to cell differentiation in a variety of cancers, such as BRCA, UCEC, KIRP, and UVM. In addition, UBE2C participated in immune response in BRCA, CHOL, KIRC, LAML, PAAD, SKCM, THCA, and UCS. UBE2C was associated with the immunoglobulin complex in CHOL and SKCM. In UCS, UBE2C was mainly responsible for immunoglobulin production and regulation of B cell activation. And UBE2C also participated in humoral immune response mediated by circulating immunoglobulin. UBE2C also played a significant role in the metabolic process across BLCA, LIHC, and READ. In MESO, UBE2C was relevant to the cell cycle and MIRNA catabolic process. Besides, UBE2C could promote the cell migration and proliferation in BLCA, CESC, and LUAD. Several cancer types were listed as examples (Figure 7). And the top, 5 GSEA results of other cancer types were presented in supplementary file3.

### 3.8. UBE2C Was Upregulated in OSCC Cell Lines and Tissues

**Figure 8:**
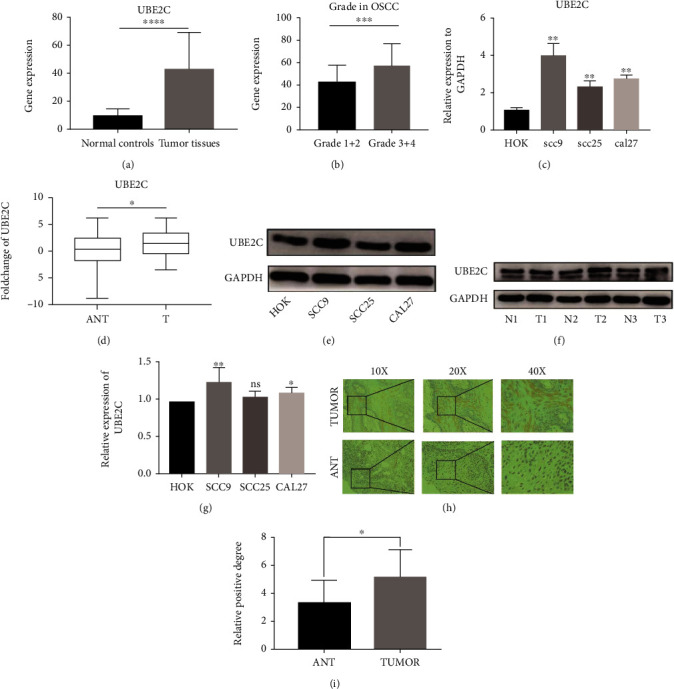
UBE2C was upregulated in OSCC. (a) The mRNA level of UBE2C in TCGA OSCC samples (32 normal samples and 319 tumor samples). (b) The high UBE2C expression level meant a high grade level in OSCC. (c, d) The mRNA and protein levels of UBE2C in SCC9 (*p* = 0.0023), SCC25 (*p* = 0.0041), CAL27 (*p* = 0.0006), and OSCC tissues (*n* = 40, *p* = 0.0192). (e, f) The UBE2C protein level in cell lines and OSCC tissues and adjacent normal tissues. (g) Statistical analysis of the UBE2C protein level in OSCC cell lines. (h) IHC staining based on OSCC tissues. (i) Statistic analysis of IHC assay (*n* = 10, *p* = 0.0432). T: tumor tissues; ANT: adjacent normal tissues; n: normal adjacent control tissues; IHC: immunohistochemistry.

First, OSCC data were downloaded from TCGA. Bioinformatics analysis indicated that UBE2C was overexpressed in TCGA (Figure 8(a)), and the higher expression level meant high grade level in OSCC (Figure 8(b)). Furthermore, the expression level of UBE2C was validated in OSCC cell lines and tissues. Our results indicated that UBE2C mRNA was upregulation in OSCC cell lines (Figure 8(c)) and in 40 OSCC patients than ANTs (Figure 8(d)), which were similar with the results in TCGA database. In addition, the protein levels of UBE2C were also upregulated in OSCC cell lines and tissues (Figures 8(e)–8(g)). Moreover, the immunohistochemistry assay also showed that UBE2C was overexpressed in OSCC tissues (Figures 8(h) and 8(i)). In addition, however, the relevance analysis had no significance between the UBE2C expression level and clinical parameters in our study (Table 4).

## 4. Discussion

The tumorigenesis and development of cancers involve multiple dysregulated processes leading to uncontrolled cell growth. According to the differentially expressed genes obtained from GEPIA2 and gene expression profiles gained from UCSC Xena, UBE2C was identified as a differentially expressed gene in 33 cancer types, which indicated that UCE2C might play an important role in carcinogenesis. The functions of UBE2C are broadly distributed across various cellular processes, including immune response, cell proliferation, migration, cell cycle, and cell differentiation. The constructed PPI network showed that some nodes were identified to play an important role in cancer development. For example, CDK1 was associated with tumor initiation in human melanoma via interacting with SOX2 [29]. In addition, CDK1 also was regarded as a potential prognostic biomarker in lung cancer [30]. Furthermore, GO enrichment analysis demonstrated that these molecules were related to the proteasome-mediated ubiquitin-dependent protein catabolic process and histone kinase activity. And according to KEGG pathway analysis, UBE2C and these genes were enrolled in the PPI network and enriched in the cell cycle and p53 signaling pathway which played a significant role in cancer initiation, progression, and therapy [31, 32]. However, a systematic analysis of the molecular characterization and functional effects of UBE2C on 33 cancer types is still lacking. Presently, accumulating evidences suggested that the immune system played an important role in the tumorigenic process [4]. Therefore, it might be helpful for understanding the potential mechanism of UBE2C in tumorigenesis and screening the potential therapeutic targets for pan-cancer to explore the role of UBE2C in TME.

In this study, we investigated the molecular characterization and TME relevance analyses of UBE2C in more than 10,000 tumor samples of 33 cancer types. The results showed that UBE2C was a differentially expressed genes in 33 cancer types. Recently, studies also showed that UBE2C was upregulated and promoted epithelial-mesenchymal transition via p53 in endometrial cancer [33]. In hepatocellular carcinoma, high UBE2C expression induced by overexpression of DNA primase subunit 1 (PRIM1) also could cause the ubiquitination and degradation of p53 [34]. Recently, a study has shown that not only did p53 become dysfunctional in most cancers but mutant p53 also acquired dominant negative activity and carcinogenic properties. And p53 remains an attractive therapeutic target for cancer therapy [35] indicating that UBE2C might be a promising therapeutic biomarker. Zhang et al. also demonstrated that the high UBE2C expression level resulted in chromosomal instability that disturbed the cell cycle and led to poor prognosis of intestinal-type gastric cancer [36]. UBE2C was regarded as an important marker of chromosomal instability and has been associated with malignant growth [37]. In addition, growing researches suggested that UBE2C was dysregulated in prostate cancer [38], cervical cancer [39], breast cancer [40], and so on. In addition, Jin et al. demonstrated that UBE2C could promote the progression of head and neck squamous cell carcinoma (HNSCC) [19]. High UBE2C expression tended to lymph node metastasis in HNSCC. Our study also indicated that UBE2C was upregulated in OSCC cell lines and tissues by RT-qPCR and IHC assay. However, due to the small sample size of this experiment, our results showed that the UBE2C expression level in OSCC had no significant relationship with the clinical parameters. In total, UBE2C could be remarkably associated with cancer development and a potential biomarker for pan-cancer. Recently, a study has demonstrated that UBE2C upregulation was relevant to the advanced histologic grade, FIGO stage, and recurrence and might be a new biomarker for the diagnosis and therapy of endometrial cancer [33]. Studies also showed that UBE2C played a crucial role in hepatocellular carcinoma [41, 42]. For instance, the lower UBE2C expression level indicated higher sensitivity for the therapy of chemotherapeutic drug, including adriamycin and 5-fluorouracil, and knockdown of UBE2C also increased the sensitivity of hepatocellular carcinoma cell to sorafenib [41]. UBE2C, as an oncogene in these cancers, played a vital role in cancer development and therapy which indicated that it might be a potential effective therapeutic target for pan-cancer. Furthermore, our study showed that TMB, a new biomarker for the prediction of immunotherapeutic response, was related to the prognosis in 13 cancer types. And the differences in prognosis between cancers may be due to tumor heterogeneity. In addition, the UBE2C expression level was remarkably related to TMB. A study demonstrated that TMB was also a useful biomarker for immune checkpoint blockade (ICB) selection across some cancer types [43]. Therefore, UBE2C might be associated with immunotherapy and might be an immunotherapeutic target in these 20 cancer types. Furthermore, TME relevance analysis also indicated that the higher UBE2C expression level meant lower StromalScore and ImmuneScore levels in many cancer types like PAAD, UCEC, and BRCA. Microenvironment-mediated drug resistance can be induced by soluble factors secreted by tumor or stromal cells [44], and the TME was significantly related to the therapeutic response and clinical outcome. Recently, a study suggested that IL-17 promoted the progression of invasive prostate adenocarcinomas under castrate conditions via creating an immunotolerant and proangiogenic TME [45]. Castrate mice without IL-17 receptor C (IL-17RC) prostate had lower rates of cellular proliferation as well as lower UBE2C protein level, indicating that UBE2C might play a role in TME of prostate cancer. In addition, our study showed that UBE2C was significantly relevant to TME in many cancer types and the TME played a crucial role in tumor initiation, progression, and metastasis process and also threw light on therapeutic efficacy [44] suggesting that UBE2C might a potential immunotherapeutic target. Unfortunately, there are no other reports about the relationship between UBE2C and TME.

At last, GSEA results showed that UBE2C was associated with the immunoglobulin complex in CHOL and SKCM and participated in the humoral immune response mediated by circulating immunoglobulin in LAML and immunoglobulin production and regulation of B cell activation in UCS. Moreover, coexpression analysis also showed that the UBE2C expression level was associated with many immune markers, such as CD200, CD276, and TMIGD2. These immune markers were related to immunosuppression and regulation of antitumor activity and participated in the regulation of T cell-mediated immune response indicating that UBE2C played a vital role in the immune response in a majority of cancer types. For example, Luo et al. demonstrated that UBE2C was involved in receptor ligand activity and the hormone activity high UBE2C expression profile meant worse biochemical recurrence-free survival in prostate cancer [46]. In addition, UBE2C might be involved in B cell receptor signaling pathways and other functions associated with immune system activation in nasopharyngeal carcinoma [47]. These results threw light on the dysregulation of UBE2C in cancers and uncover that UBE2C might be a potential diagnostic and therapeutic biomarker for various cancers.

## 5. Conclusions

In our study, UBE2C was identified to be differentially expressed in many cancer types and related to the initiation, progression, and prognosis of many cancers. TME relevance analysis indicated that UBE2C also played a crucial role in the immune response of various cancers and the results are consistent with GSEA results. In summary, the systematic analysis of UBE2C revealed that it might be a diagnostic and therapeutic biomarker across many cancers.

Though our study might have vital clinical significance, there are a few limitations in this study. First, UBE2C has not been identified as an effective biomarker in a variety of cancer samples. Second, the potential mechanism of UBE2C promoting some cancer initiation and progression should be investigated in further study. Third, the relevance between UBE2C and immune response needs further investigation.

## Figures and Tables

**Table 1:** UBE2C fold change (FC) in 29 cancer types.

Cancer type	UBE2C-FC	Cancer type	UBE2C-FC	Cancer type	UBE2C-FC
ACC	3.219	GBM	5.784	LUAD	3.44
BLCA	4.775	HNSC	1.877	LUSC	4.555
BRCA	4.527	KICH	1.684	OV	6.387
CESC	6.252	KIRC	1.942	PAAD	4.563
DLBC	4.723	KIRP	1.496	PRAD	1.439
ESCA	1.867	LAML	3.796	READ	5.368
CHOL	6.252	LGG	2.802	SKCM	2.633
COAD	4.604	LIHC	3.154	STAD	4.794
TGCT	2.657	THYM	4.923	UCS	6.664
THCA	1.249	UCEC	6.145		

**Table 2:** Tumor mutational burden (TMB) relevance analysis.

Cancer type	Cor	*p* value	Cancer type	Cor	*p* value
ACC	0.5267336	6.15*E* − 07	LUSC	0.264631	2.90E − 09
BLCA	0.2995809	6.61*E* − 10	MESO	0.2954638	0.008203379
BRCA	0.4575794	1.44*E* − 51	OV	0.1333672	0.027859984
CESC	0.1985855	0.00073181	PAAD	0.4740717	7.82*E* − 10
CHOL	0.1652712	0.335408458	PCPG	0.1081349	0.151957209
COAD	−0.175912	0.000436444	PRAD	0.4780798	6.78*E* − 29
DLBC	0.0851114	0.615292472	READ	0.0998543	0.254629595
ESCA	0.0486291	0.541429409	SARC	0.4285829	6.45*E* − 12
GBM	0.1170443	0.156568875	SKCM	0.1800089	9.49*E* − 05
HNSC	0.1262425	0.00504261	STAD	0.3498846	4.89*E* − 12
KICH	0.4329548	0.000315541	TGCT	0.1323744	0.112468468
KIRC	0.1576122	0.003989195	THCA	0.0865158	0.057690945
KIRP	−0.073869	0.219531431	THYM	−0.695502	3.28*E* − 18
LAML	0.1236121	0.334443832	UCEC	0.0980601	0.024646306
LGG	0.4104412	8.83*E* − 22	UCS	0.0828162	0.543980035
LIHC	0.0671965	0.204017719	UVM	0.1066263	0.346514073
LUAD	0.4474419	3.93*E* − 26			

**Table 3:** StromalScore and ImmuneScore relevance analyses.

Cancer type	StromalScore (*p* value)	ImmuneScore (*p* value)	Cancer type	StromalScore (*p* value)	ImmuneScore (*p* value)
ACC	0.039206664	0.043914766	LUSC	<0.0001	7.93*E* − 09
BLCA	0.02576853	0.344845775	MESO	0.350009942	0.987592237
BRCA	<0.0001	0.032604764	OV	0.022205613	0.059919863
CESC	5.63*E* − 05	0.000526389	PAAD	1.49*E*–06	0.000328994
CHOL	0.424329989	0.888603589	PCPG	0.469691421	0.091593433
COAD	5.43*E* − 10	1.27*E* − 11	PRAD	0.797111161	0.452014481
DLBC	0.656490741	0.010779696	READ	0.000502512	6.38*E* − 06
ESCA	0.025103976	0.077152512	SARC	0.001291543	0.471153803
GBM	1.98*E* − 08	1.08*E*–08	SKCM	0.001624749	0.246571531
HNSC	4.93*E* − 12	0.033907848	STAD	<0.0001	1.09*E* − 06
KICH	0.027436293	0.030604563	TGCT	0.000812132	0.01318386
KIRC	0.0125631	8.10*E* − 17	THCA	<0.0001	<0.0001
KIRP	0.839715612	0.765465463	THYM	1.19*E* − 05	0.001692186
LAML	0.489788421	0.185129925	UCEC	1.58*E* − 06	3.09*E* − 05
LGG	0.029818407	0.001691075	UCS	0.131139903	0.164492641
LIHC	1.86*E* − 06	0.670876508	UVM	0.608394004	0.848009961
LUAD	0.000178847	0.059633661			

**Table 4:** Correlation between UBE2C expression and clinical parameters in OSCC patients (*n* = 40).

Parameters	n	UBE2C (%)		*p* value
		High	Low	
		Expression	Expression	
Age (years)				0.4882
≥60	29	14	15	
<60	11	7	4	
Gender				0.7471
Male	23	13	10	
Female	17	11	6	
Stage				0.4844
I + II	28	17	11	
III + IV	12	9	3	
T classification				0.7471
T1 + T2	24	13	11	
T3 + T4	16	10	6	
N classification				0.7053
N0	22	18	4	
N1 + N2 + N3	18	13	5	

## Data Availability

All data for this study are available from the corresponding authors if required.

## Abbreviations

DEGs: Differentially expressed genes
OSCC: Oral squamous cell carcinoma
UBE2C: Ubiquitin-conjugating enzyme E2C
TCGA: The Cancer Genome Atlas
DEGs: Differentially expressed genes
GSEA: Gene set enrichment analysis
OS: Overall survival
DSS: Disease-specific survival
DFI: Disease-free interval
PFI: Progression-free interval
PPI: Protein-protein interaction
TMB: Tumor mutational burden
TME: Tumor microenvironment
GO: Gene Ontology
KEGG: Kyoto Encyclopedia of Genes and Genomes
BP: Biological processes
CC: Cellular component
MF: Molecular function
ANTs: Adjacent normal tissues.

## Additional Points

*Statement*. A preprint has previously been published according to this manuscript [48].

## Supplementary Materials

Supplementary 1 Figure S1: univariate Cox regression analysis of UBE2C according to OS (A), DSS (B), DFI (C), and PFI (D).

Supplementary 2 Supplementary Table1: the *p* value of differentially expressed analysis in Figure 1(b). Supplementary Table2: the Spearman's correlation coefficient between UBE2C and immune markers. Supplementary Table3: the *p* value of correlation analysis between UBE2C and immune markers. Supplementary Table4: the *p* value between UBE2C expression and 22 immune cells correlation analysis.

Supplementary 3 Supplementary file1: tumor microenvironment (TME) relevance analysis was listed. Supplementary file2: visualization of relevance analysis between UBE2C expression and 22 immune cell levels. Supplementary file3: the gene set enrichment analysis (GSEA) results of the other 23 cancer types.
