# Supplementary figures and images for: Pan-Cancer Analyses of the Tumor Microenvironment Reveal That Ubiquitin-Conjugating Enzyme E2C Might Be a Potential Immunotherapy Target
Source: J Immunol Res. 2021 Dec 13;2021:9250207. doi: 10.1155/2021/9250207 (PMC8689232; doi:10.1155/2021/9250207)

FigureS1


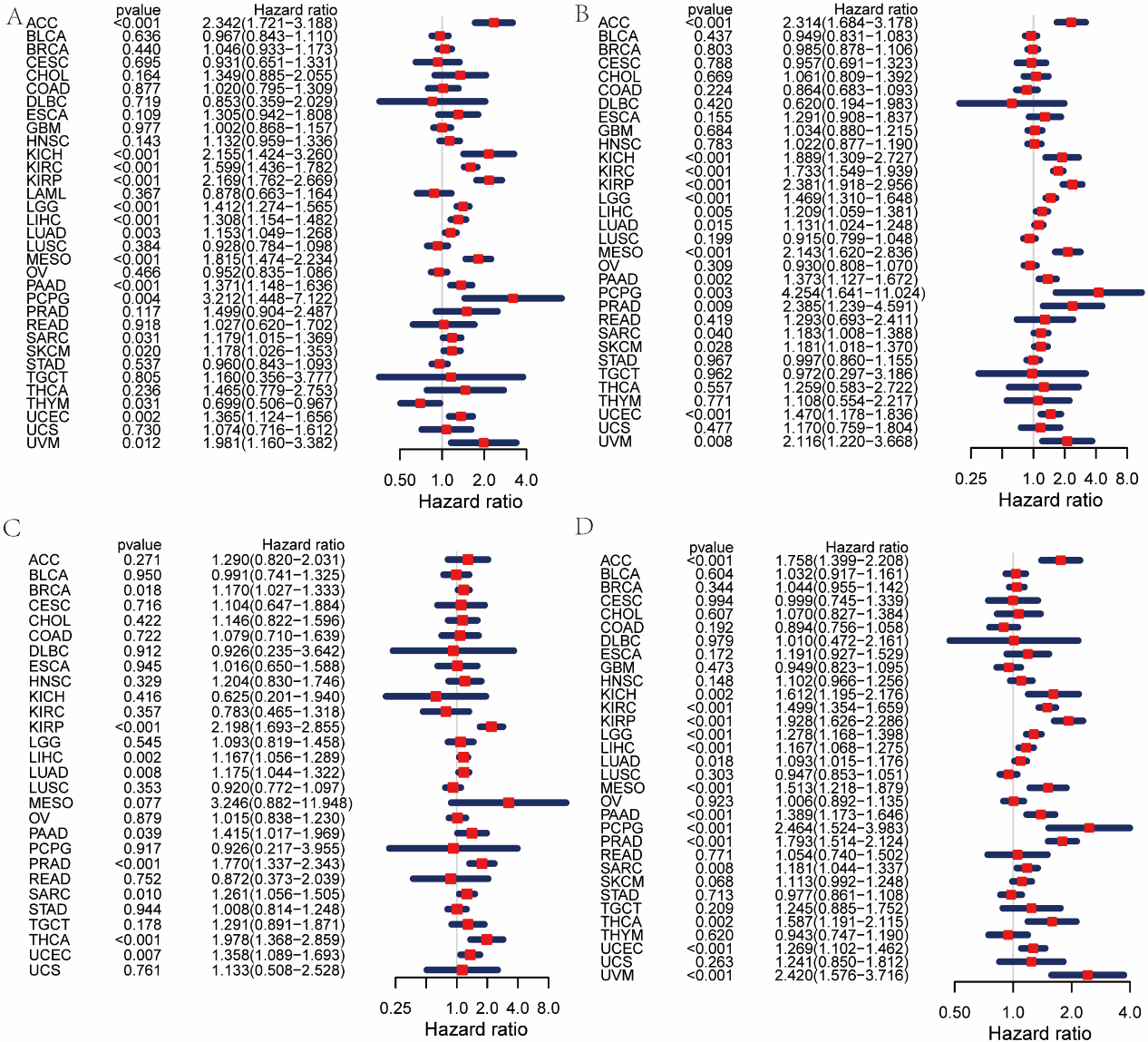

Supplement: Supplementary 1 — Figure S1: univariate Cox regression analysis of UBE2C according to OS (A), DSS (B), DFI (C), and PFI (D). [file 9250207.f1.docx]

Cancer: LIHC

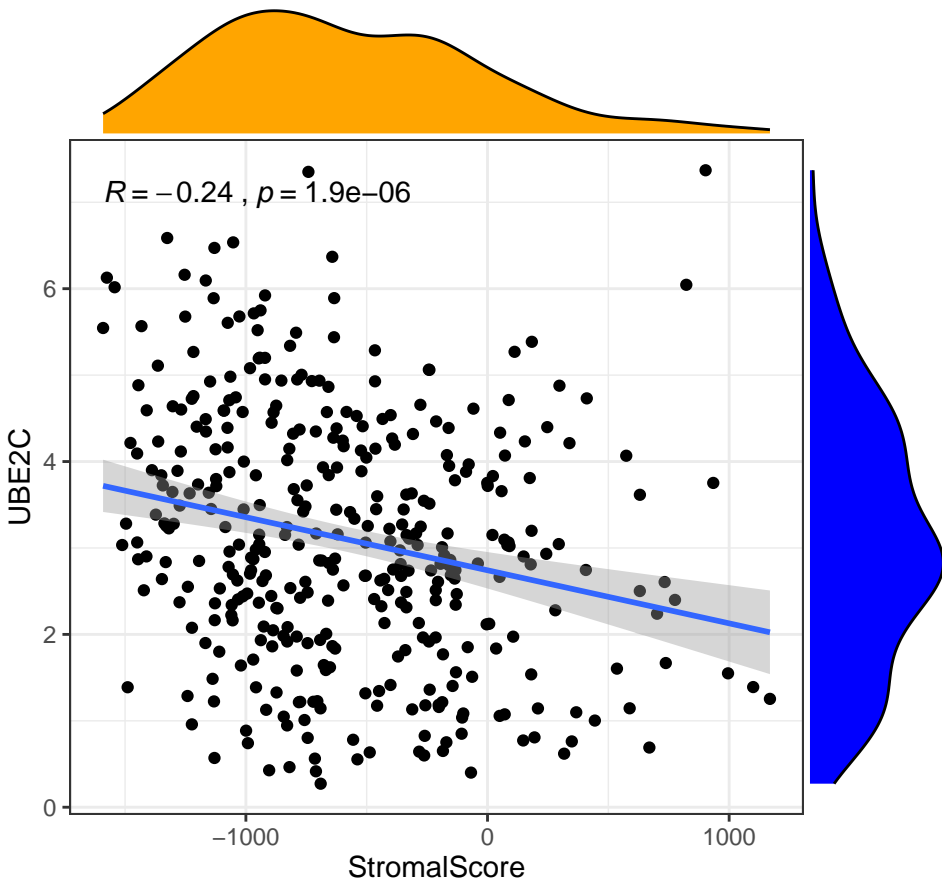

Cancer: LUAD

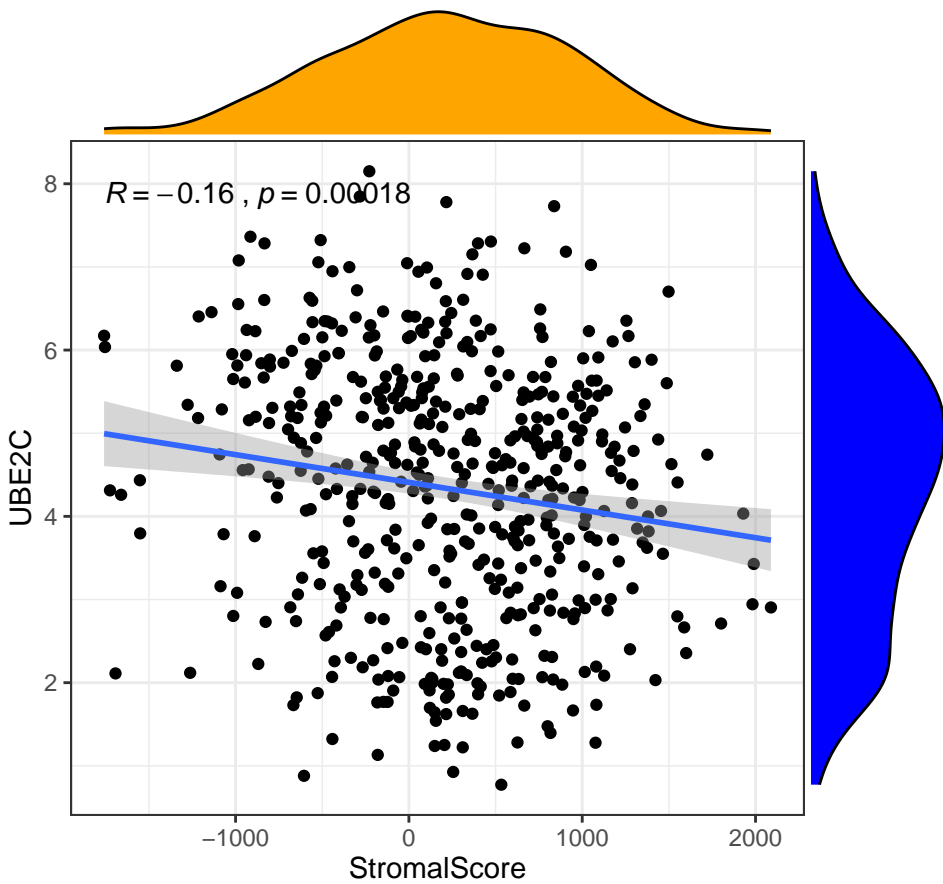

Cancer: TGCT

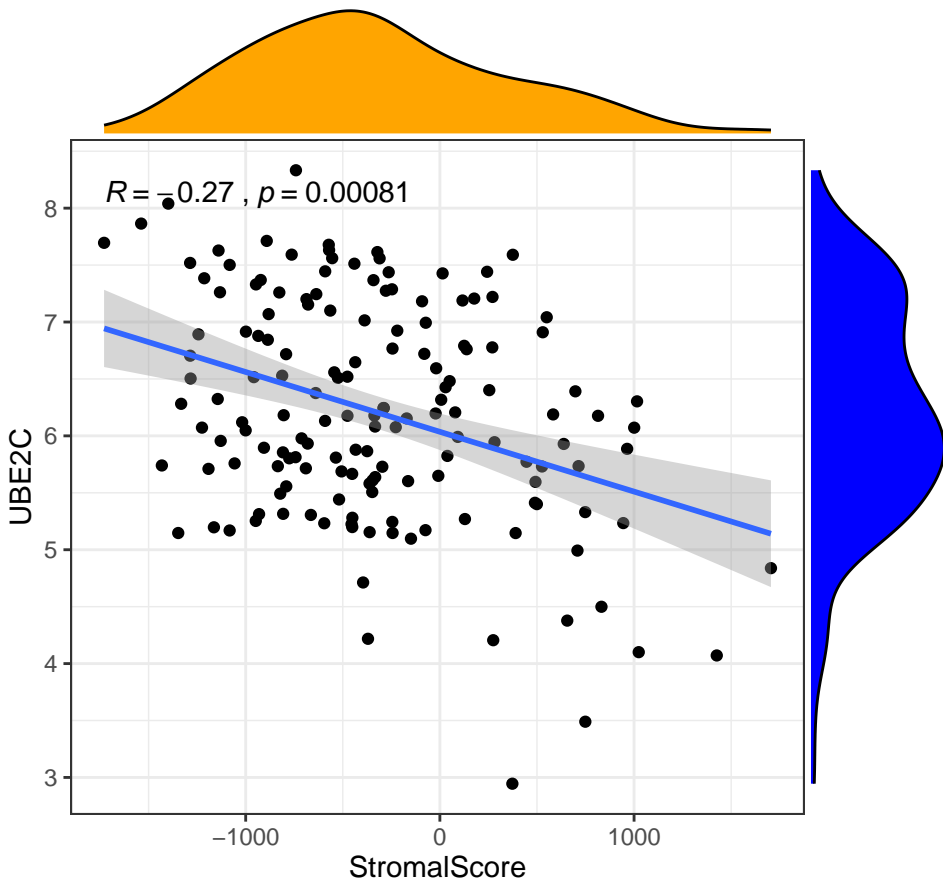

Cancer: THYM

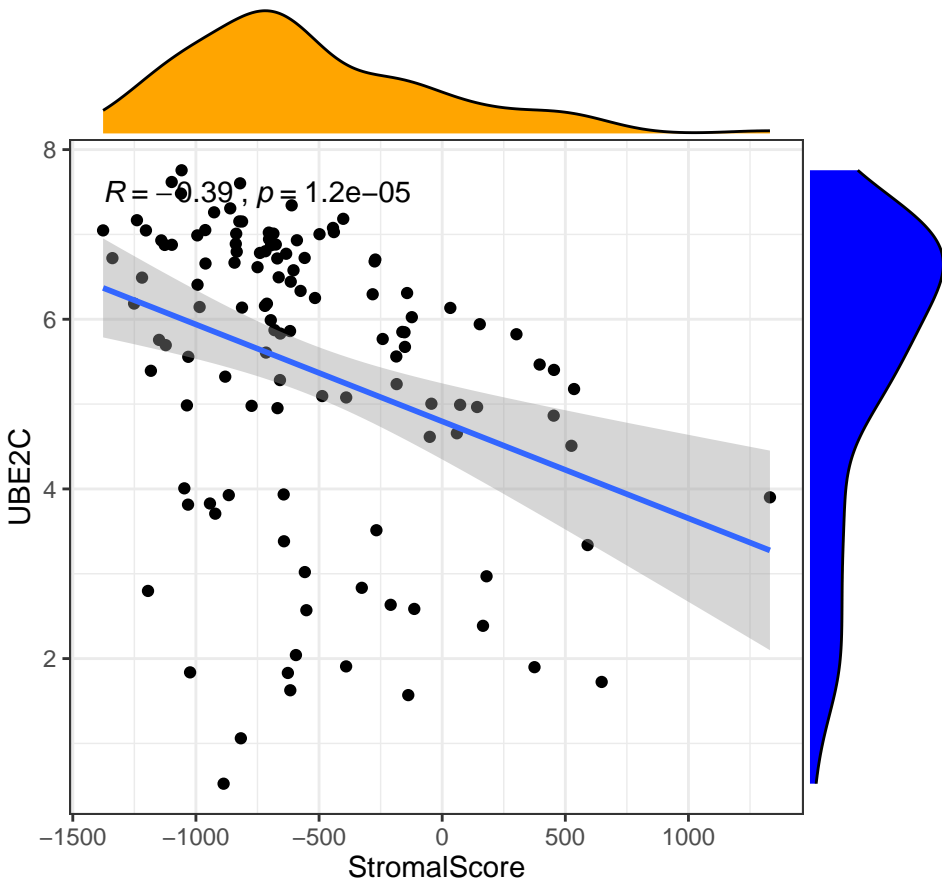

Cancer: KIRC

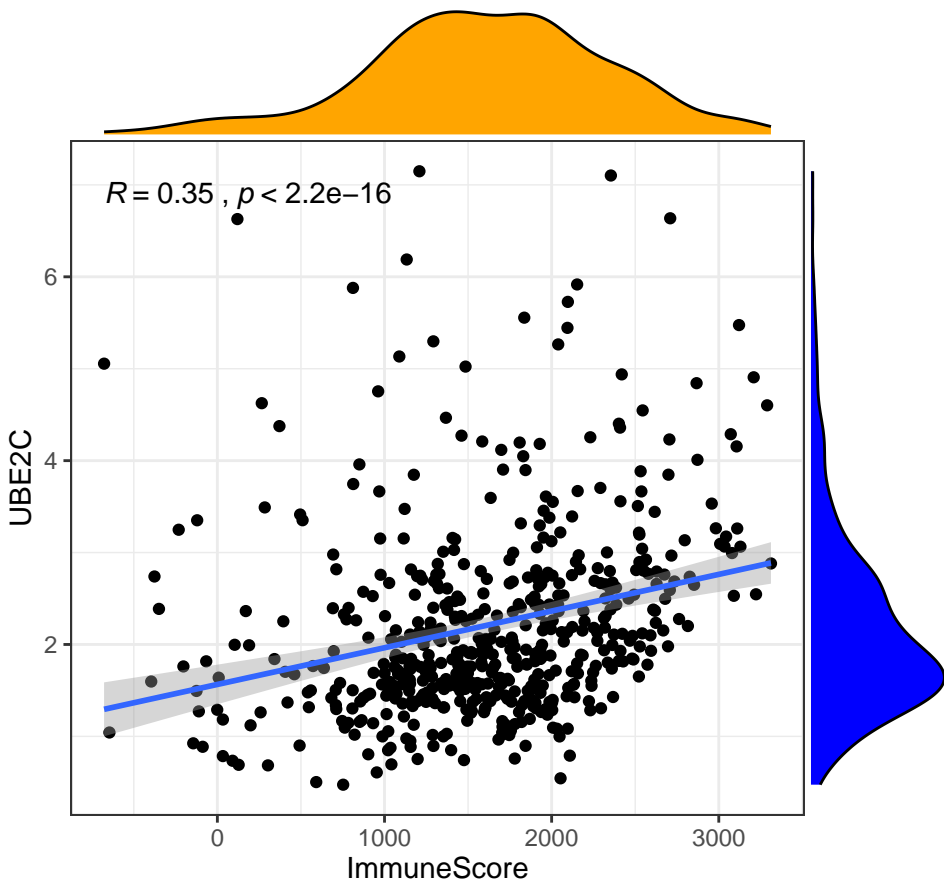

Supplement: Supplementary 3 — Supplementary file1: tumor microenvironment (TME) relevance analysis was listed. Supplementary file2: visualization of relevance analysis between UBE2C expression and 22 immune cell levels. Supplementary file3: the gene set enrichment analysis (GSEA) results of the other 23 cancer types. [file 9250207.f3.zip › Supplementary file1 (1).pdf]

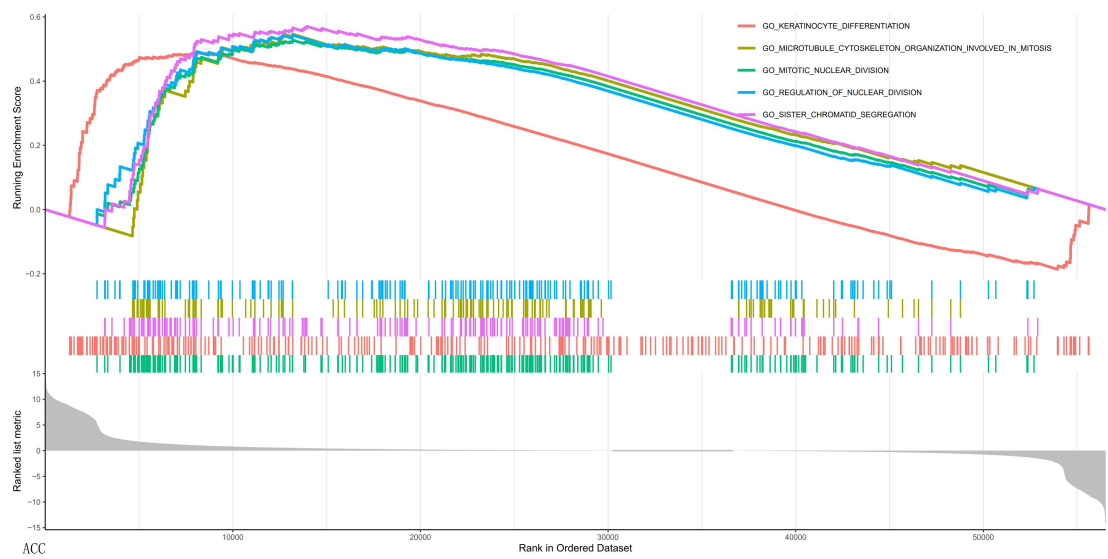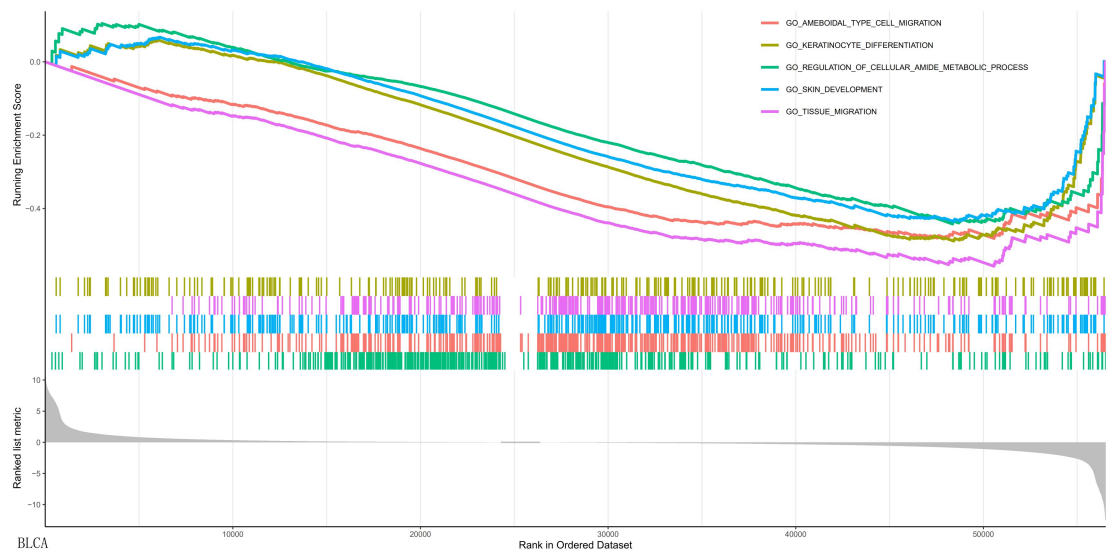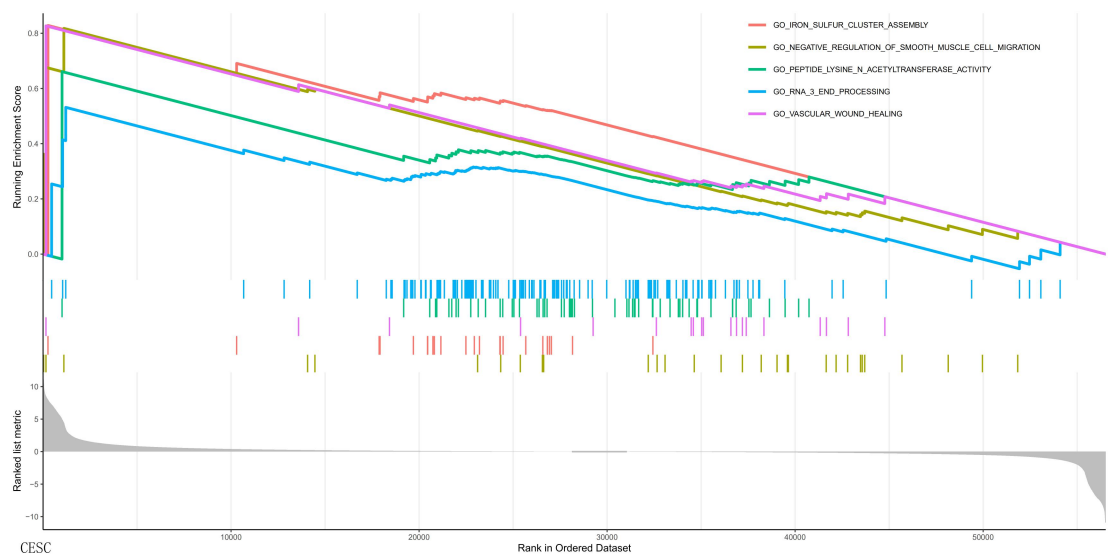

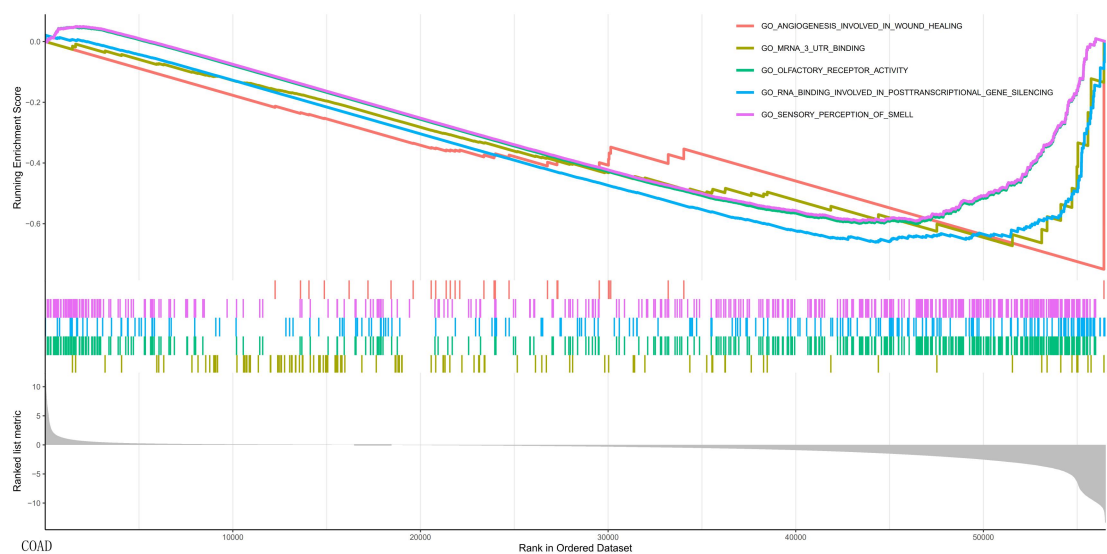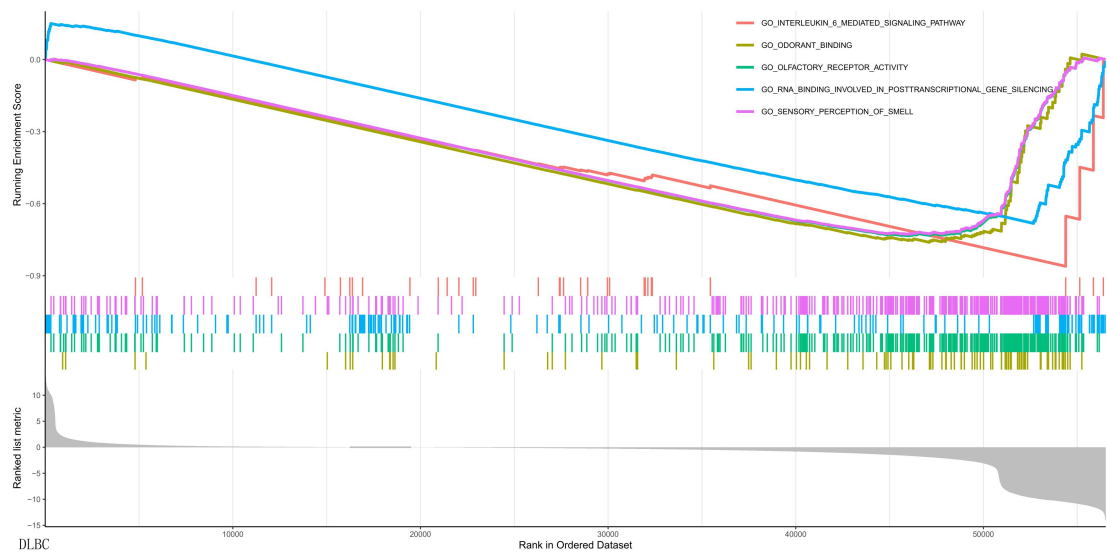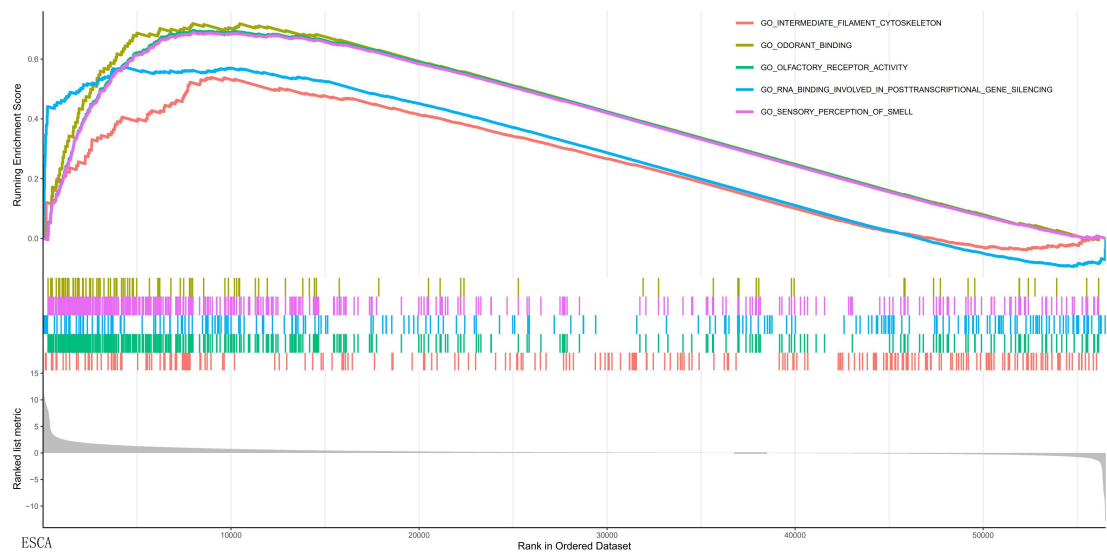

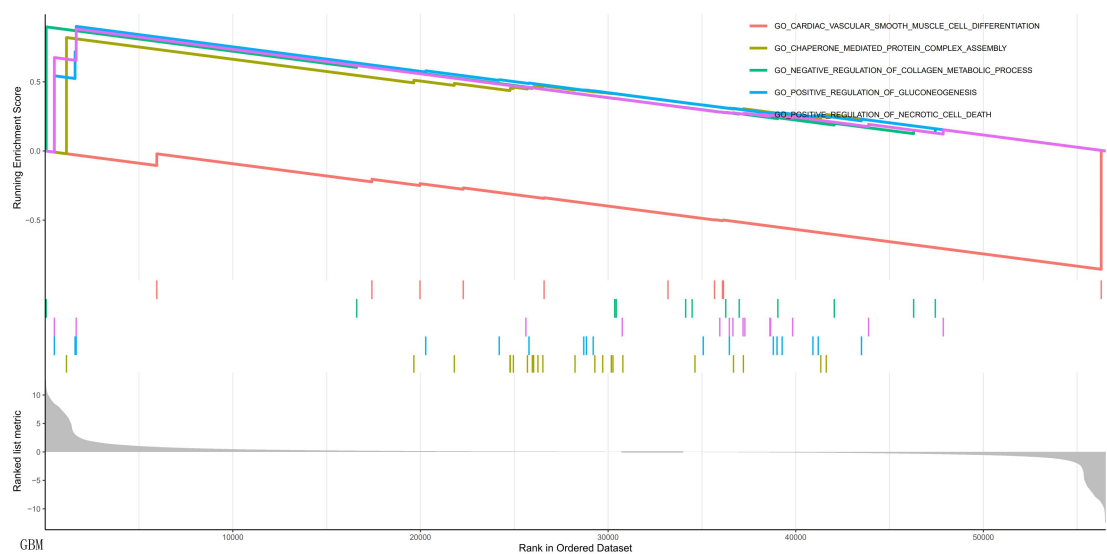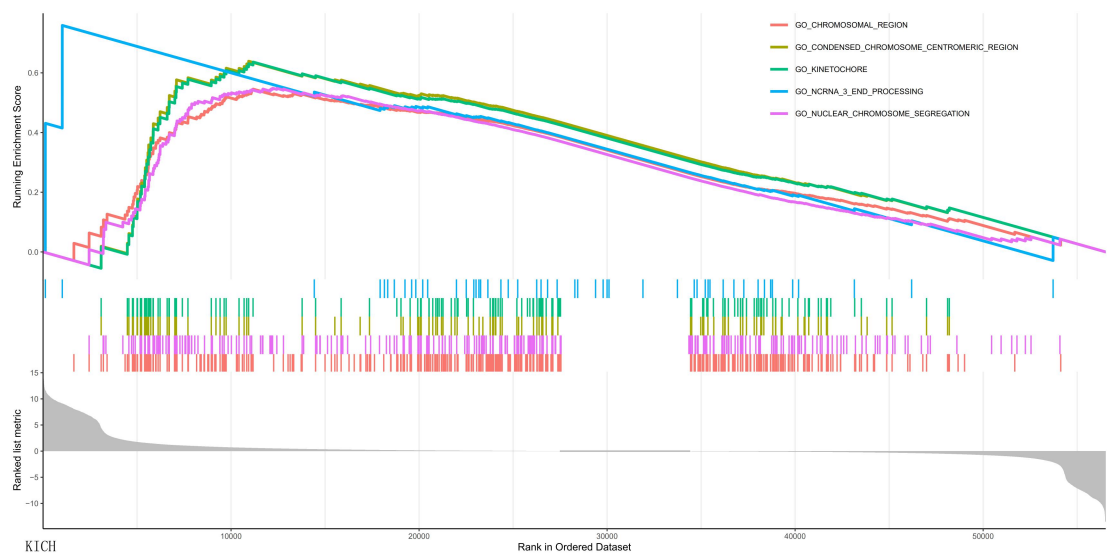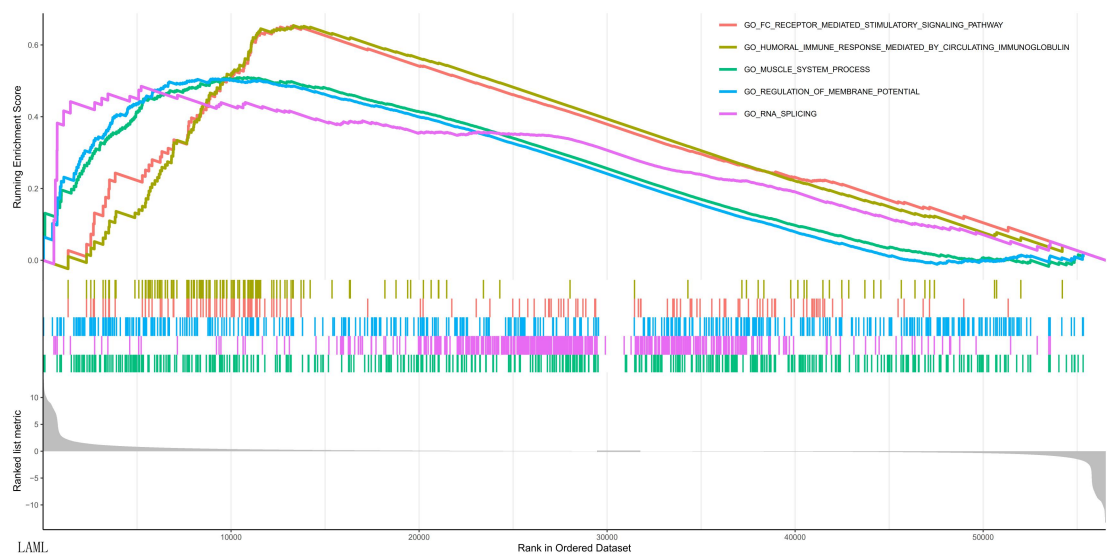

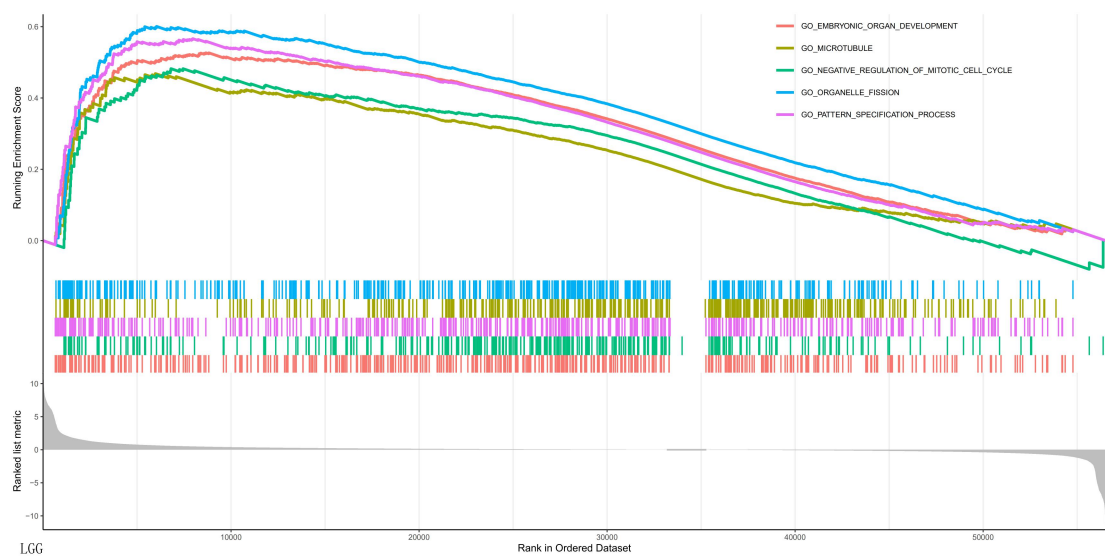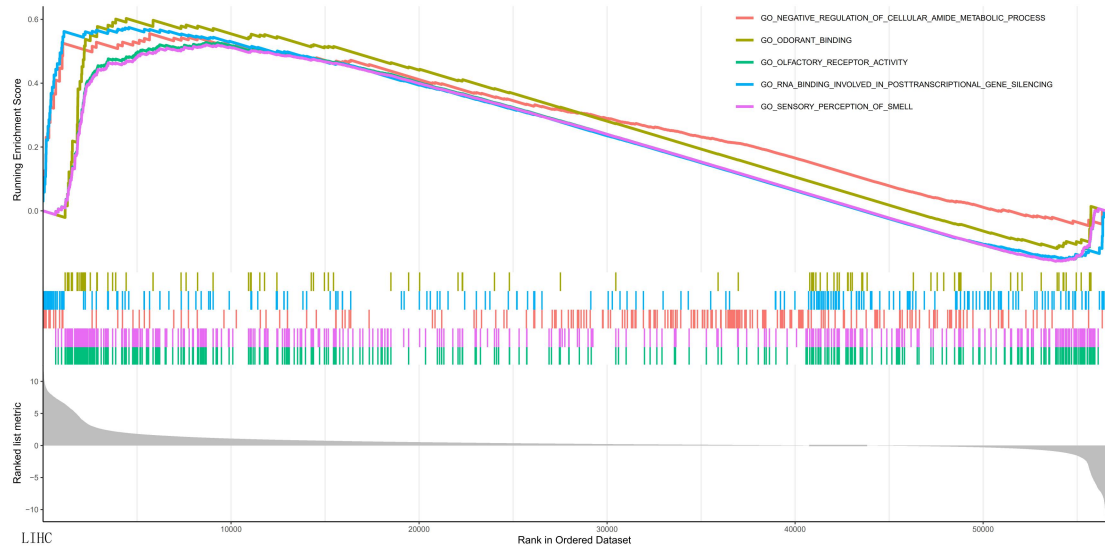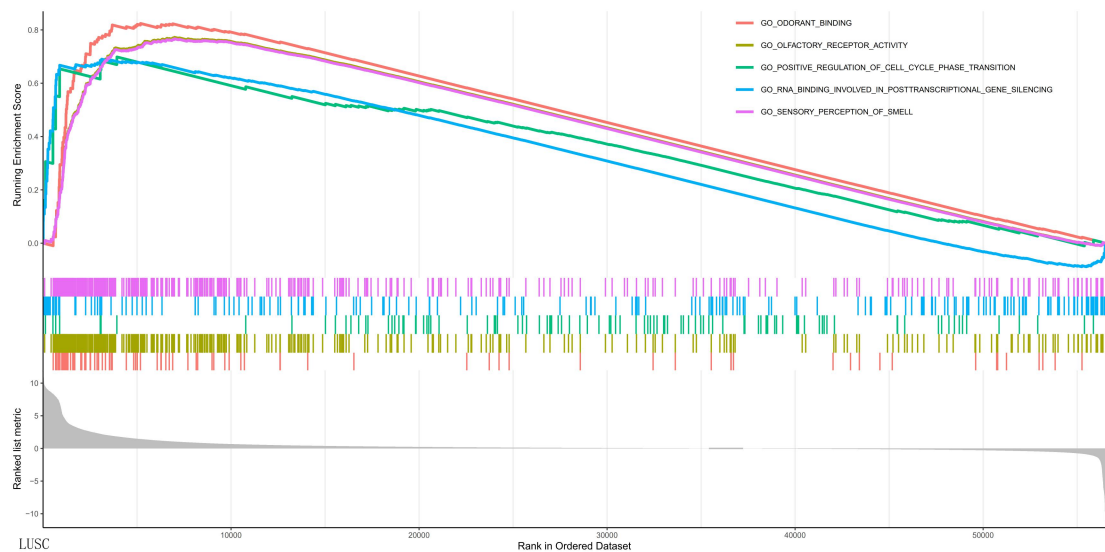

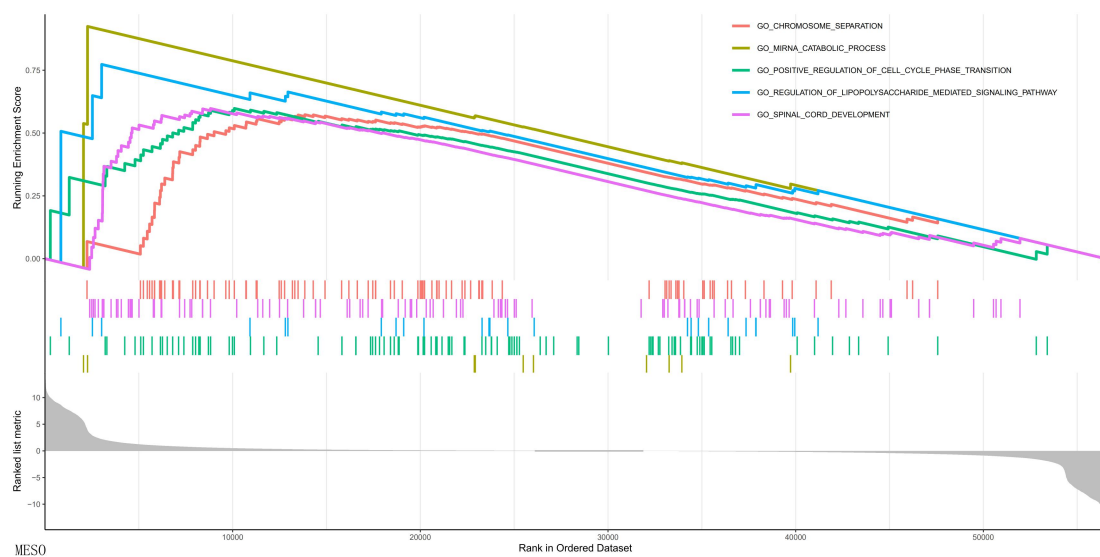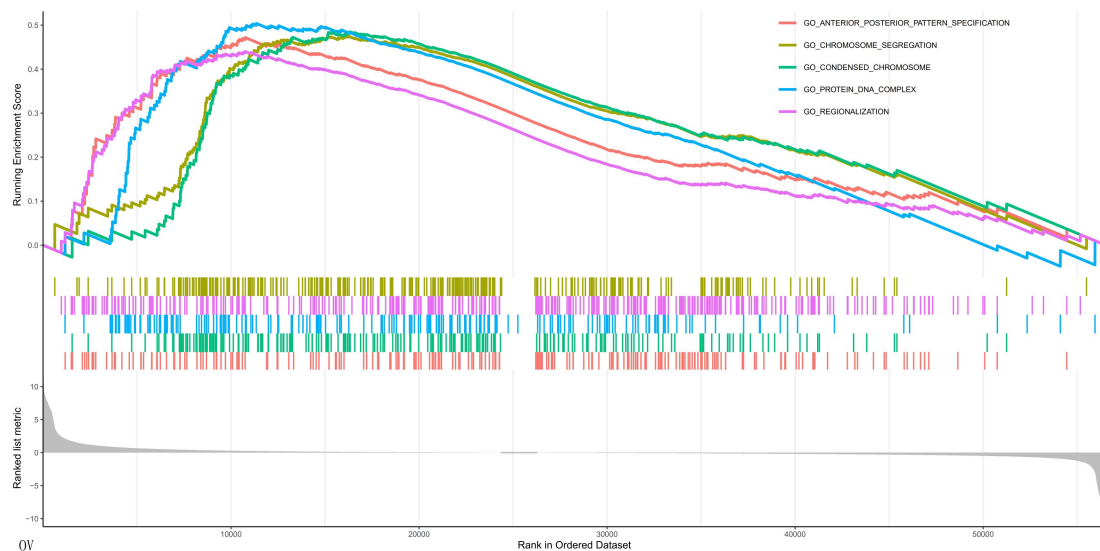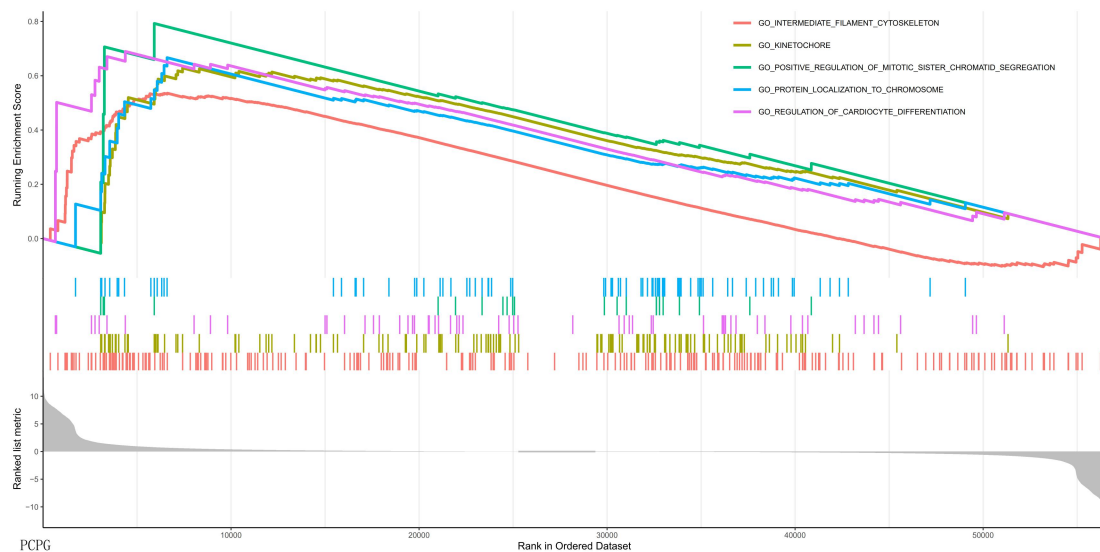

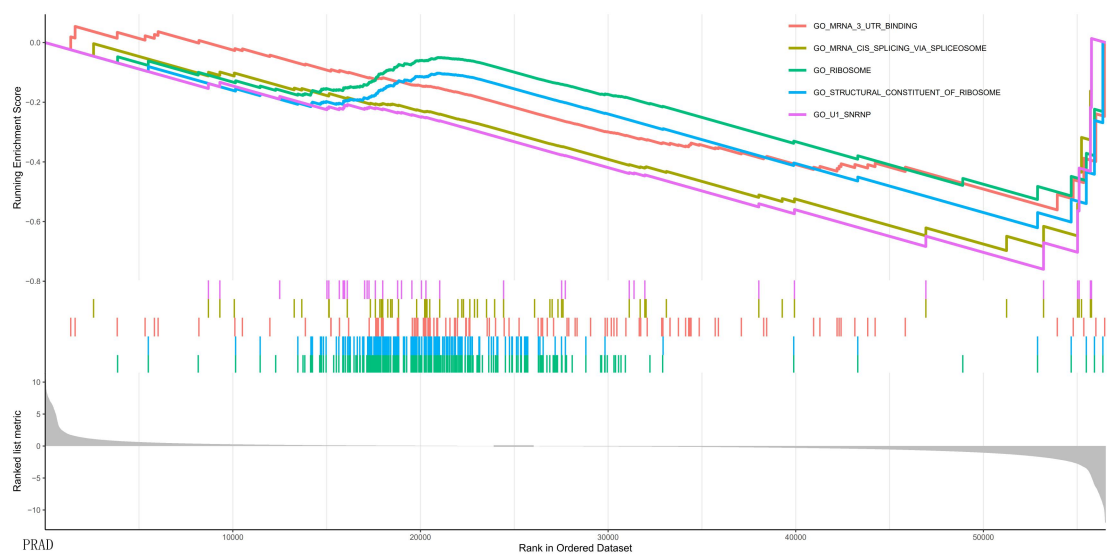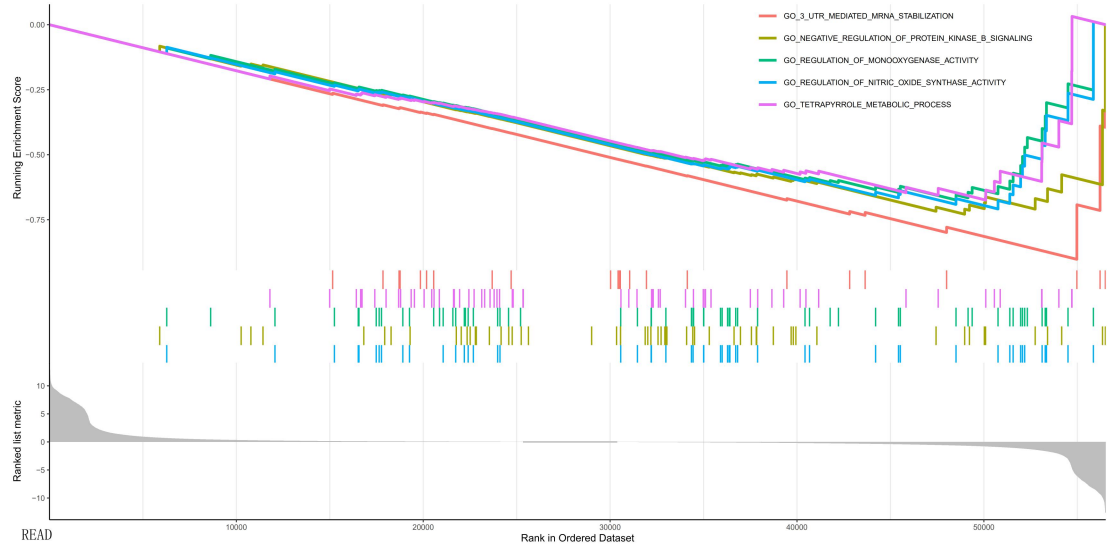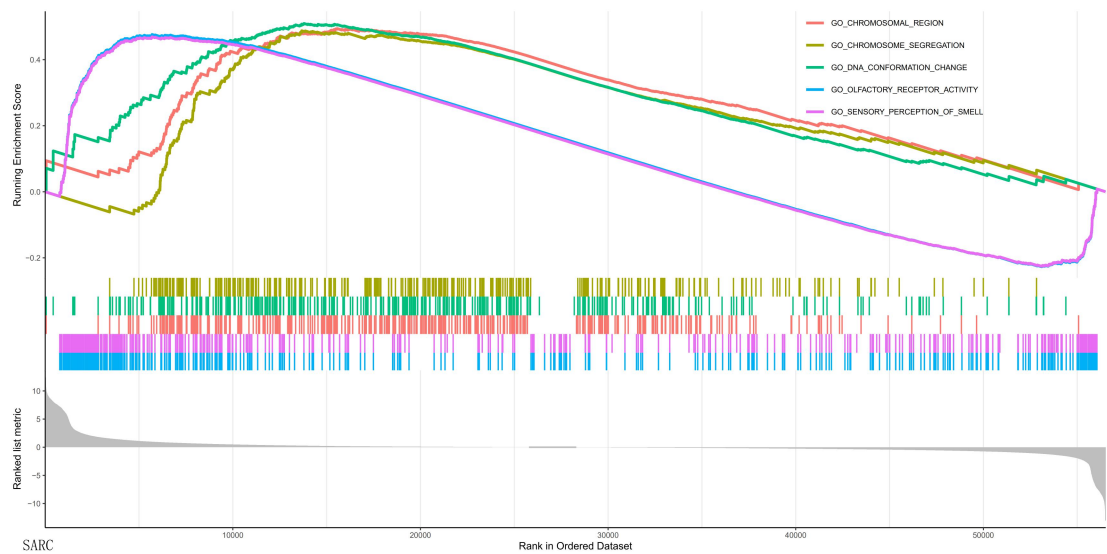

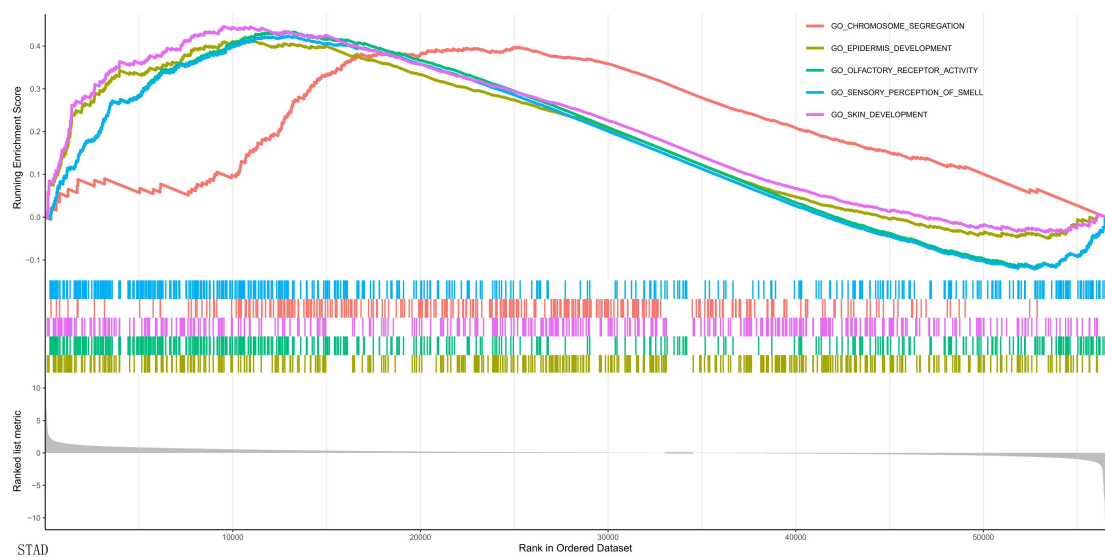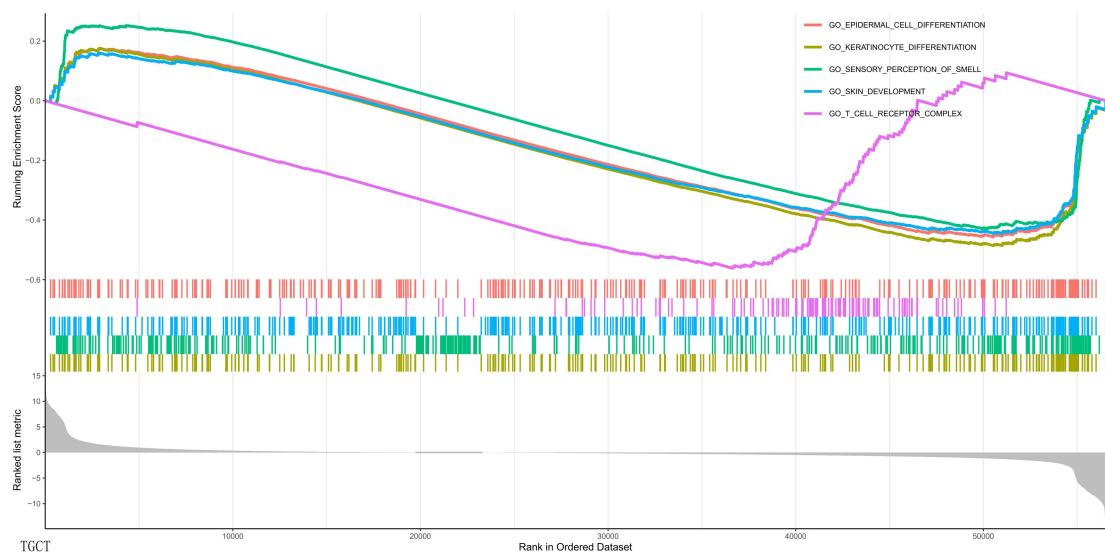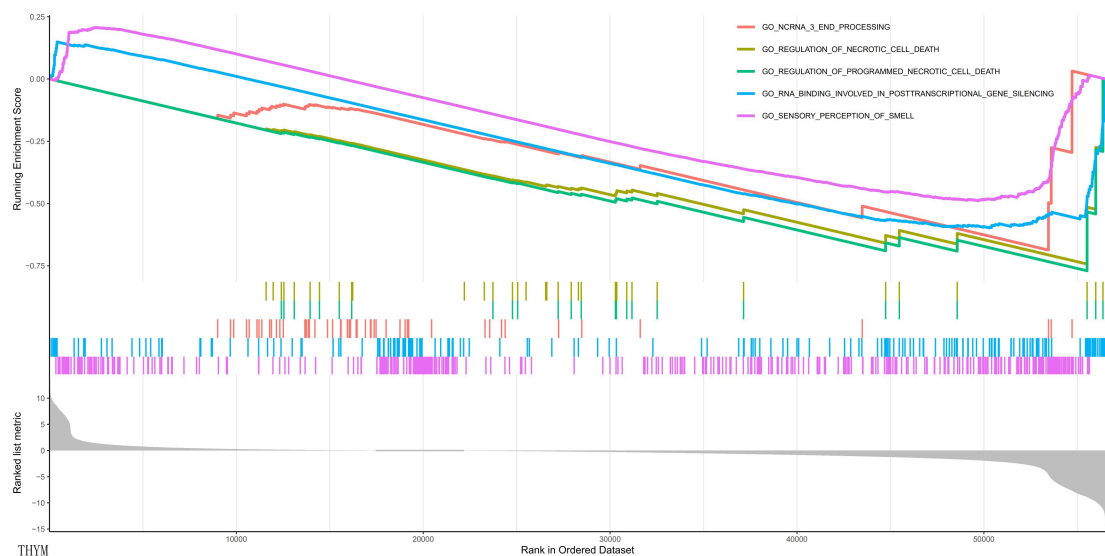

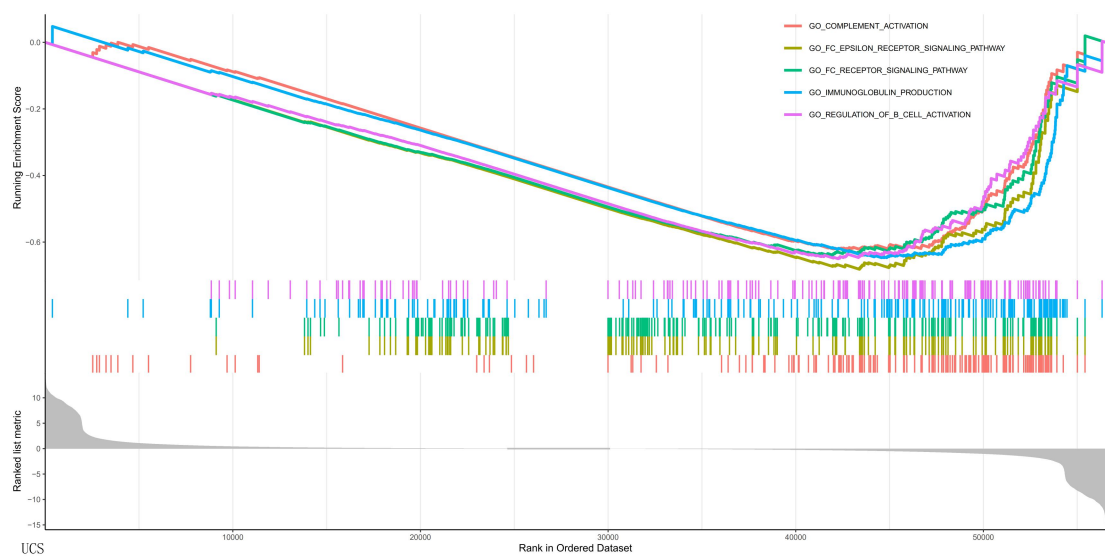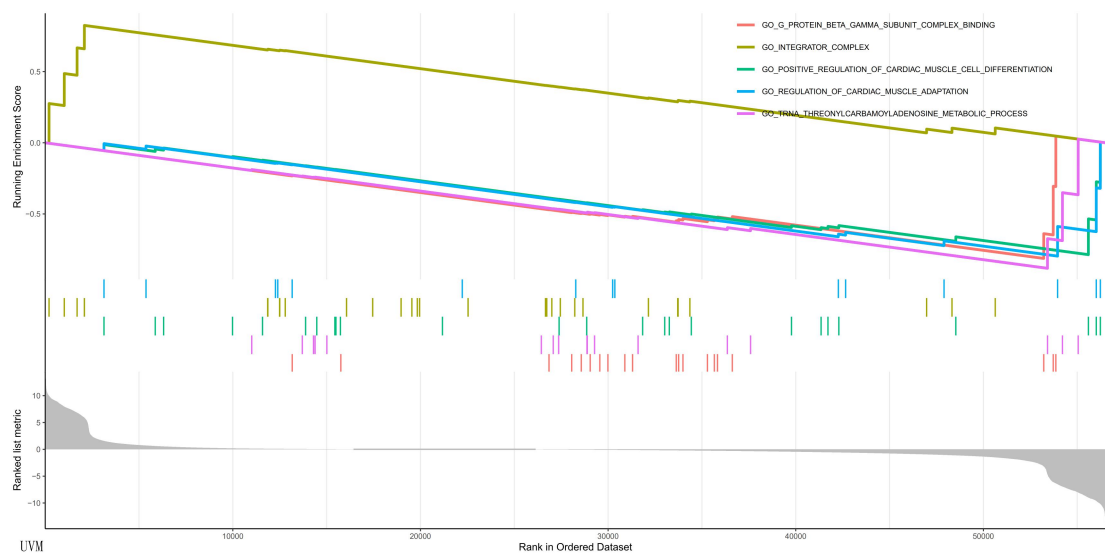

Supplement: Supplementary 3 — Supplementary file1: tumor microenvironment (TME) relevance analysis was listed. Supplementary file2: visualization of relevance analysis between UBE2C expression and 22 immune cell levels. Supplementary file3: the gene set enrichment analysis (GSEA) results of the other 23 cancer types. [file 9250207.f3.zip › Supplementary file3.pdf]
