# Supplementary material for: Pan-Cancer Analyses of the Tumor Microenvironment Reveal That Ubiquitin-Conjugating Enzyme E2C Might Be a Potential Immunotherapy Target
Source: J Immunol Res. 2021 Dec 13;2021:9250207. doi: 10.1155/2021/9250207 (PMC8689232; doi:10.1155/2021/9250207)
Supplement: Supplementary 3 — Supplementary file1: tumor microenvironment (TME) relevance analysis was listed. Supplementary file2: visualization of relevance analysis between UBE2C expression and 22 immune cell levels. Supplementary file3: the gene set enrichment analysis (GSEA) results of the other 23 cancer types. [file 9250207.f3.zip › Supplementary file2.pdf]

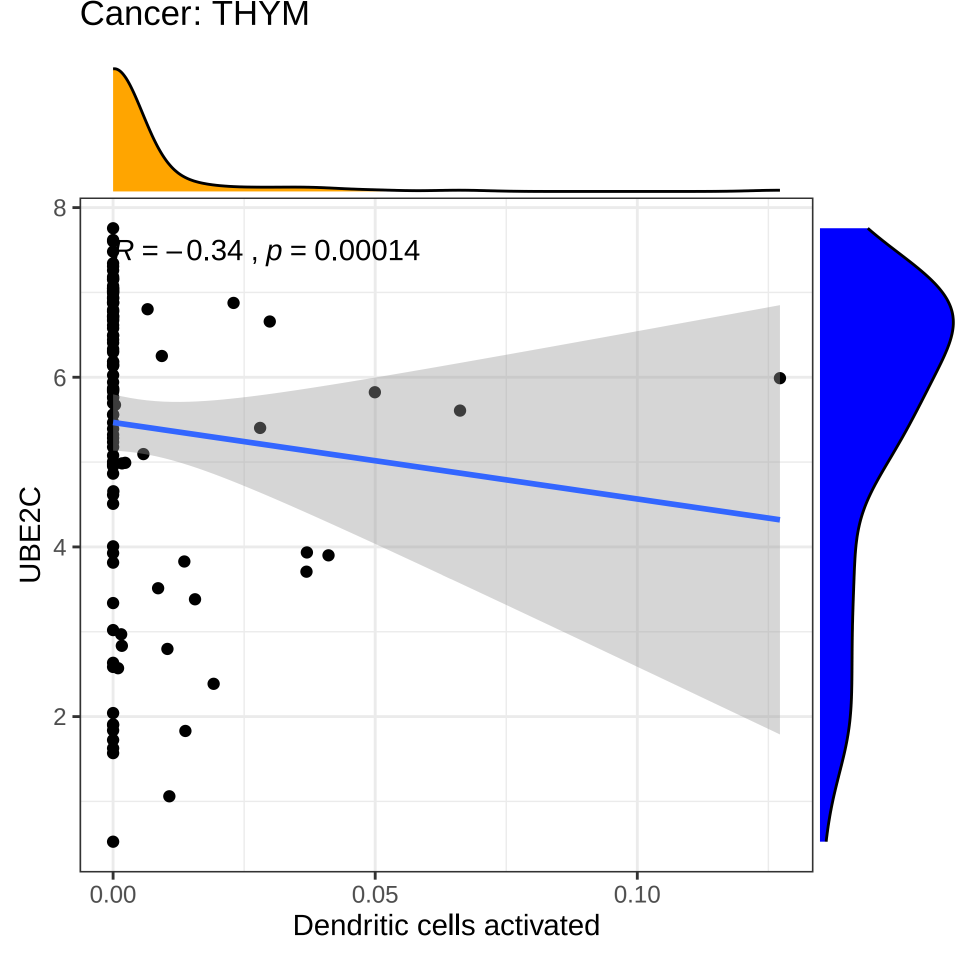
Cancer: THYM
R = −0.34 , p = 0.00014
8
6
4
2
0.00
0.05
0.10
UBE2C
Dendritic cells activated

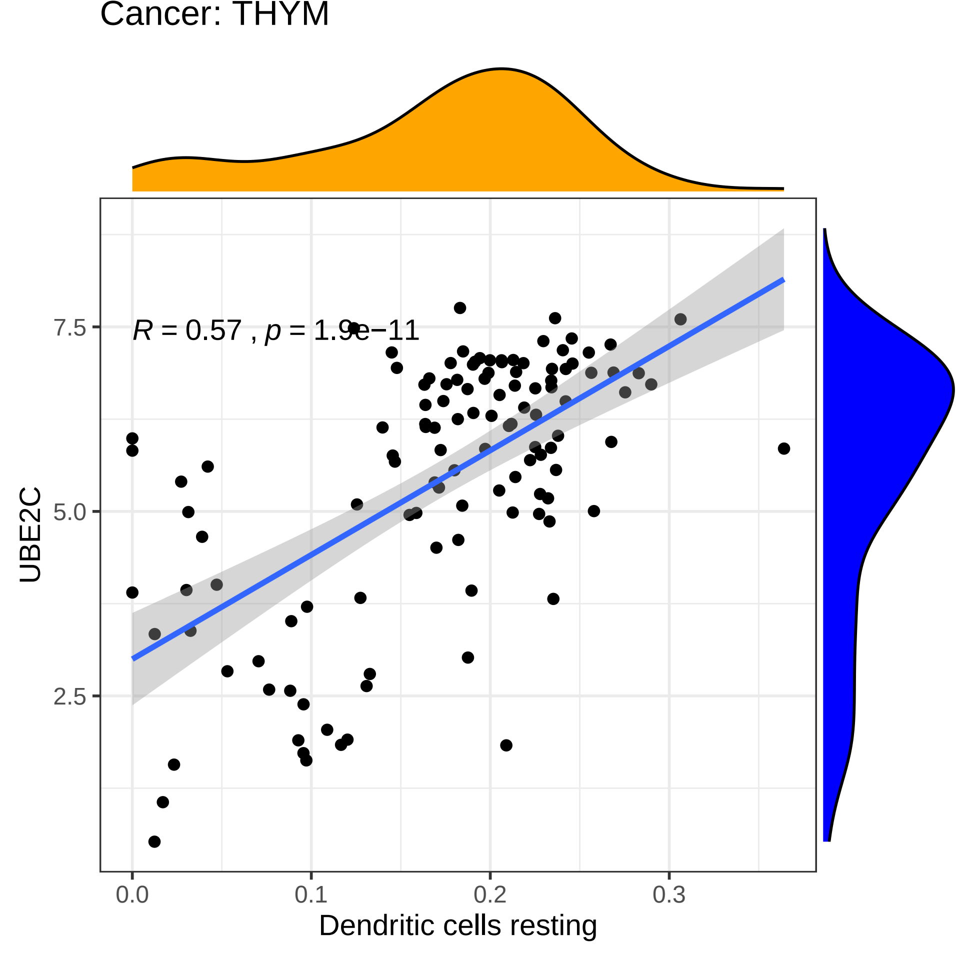
Cancer: THYM
R = 0.57 , p = 1.9e−11
7.5
5.0
2.5
UBE2C
0.0
0.1
0.2
0.3
Dendritic cells resting

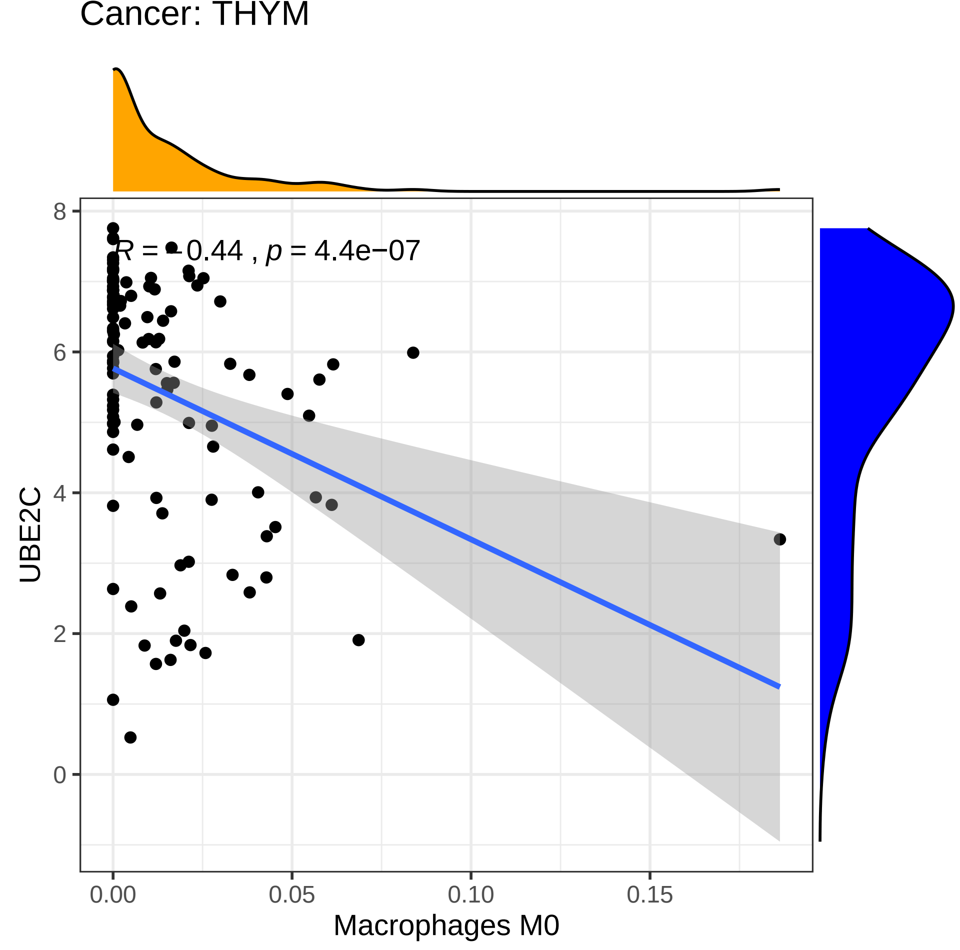

Cancer: THYM
R = −0.44 , p = 4.4e−07
UBE2C
Macrophages M0
0
2
4
6
8
0.00
0.05
0.10
0.15

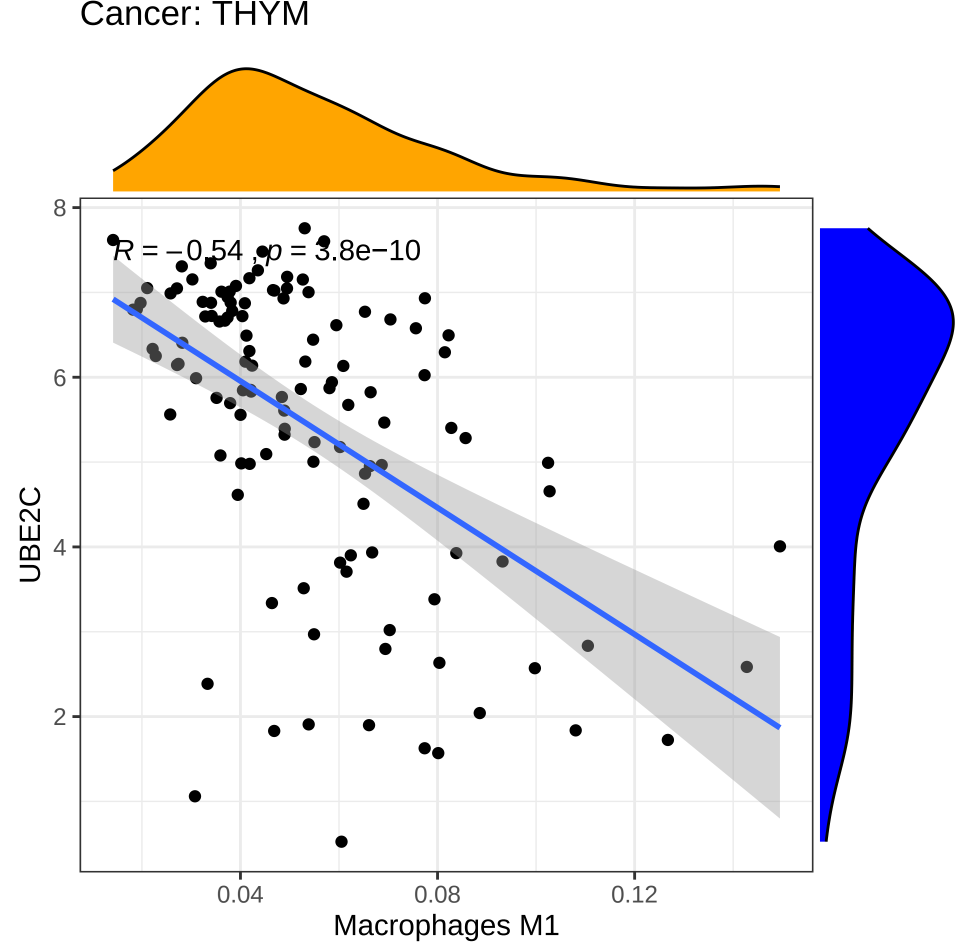
Cancer: THYM
R = −0.54 , p = 3.8e−10
8
6
4
2
UBE2C
0.04
0.08
0.12
Macrophages M1

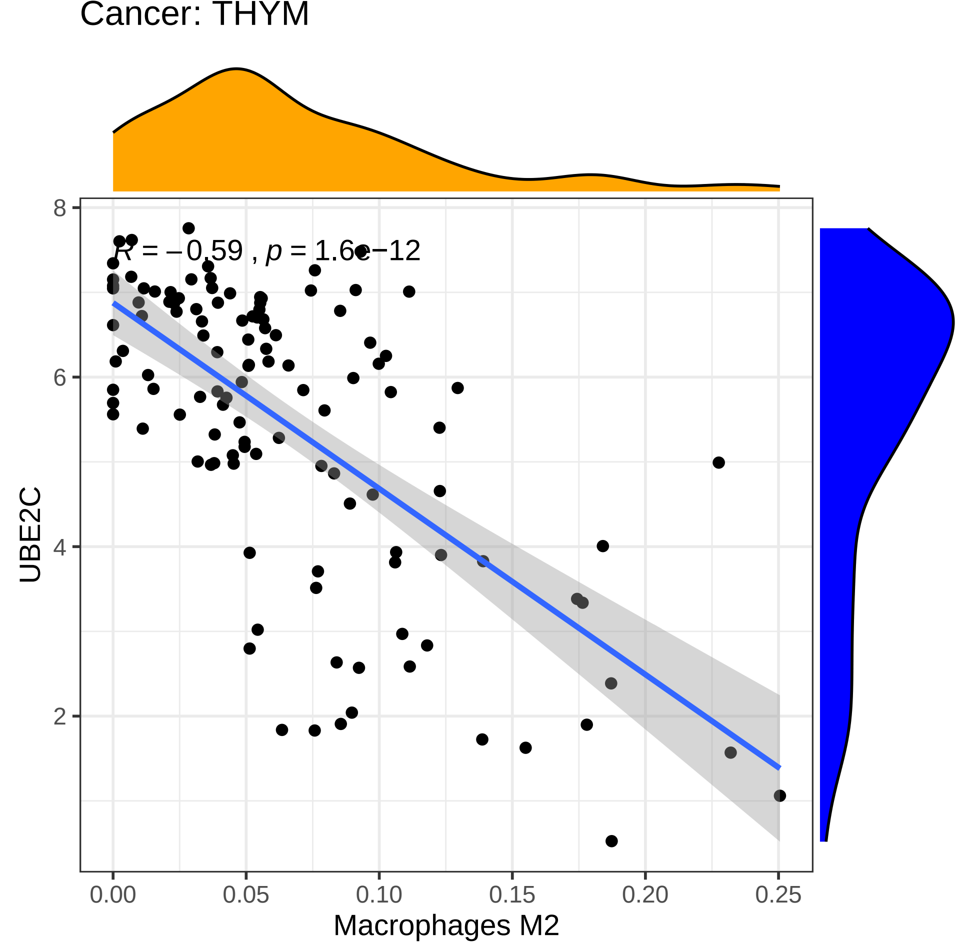

Cancer: THYM
R = −0.59 , p = 1.6e−12
8
6
4
2
UBE2C
0.00
0.05
0.10
0.15
0.20
0.25
Macrophages M2

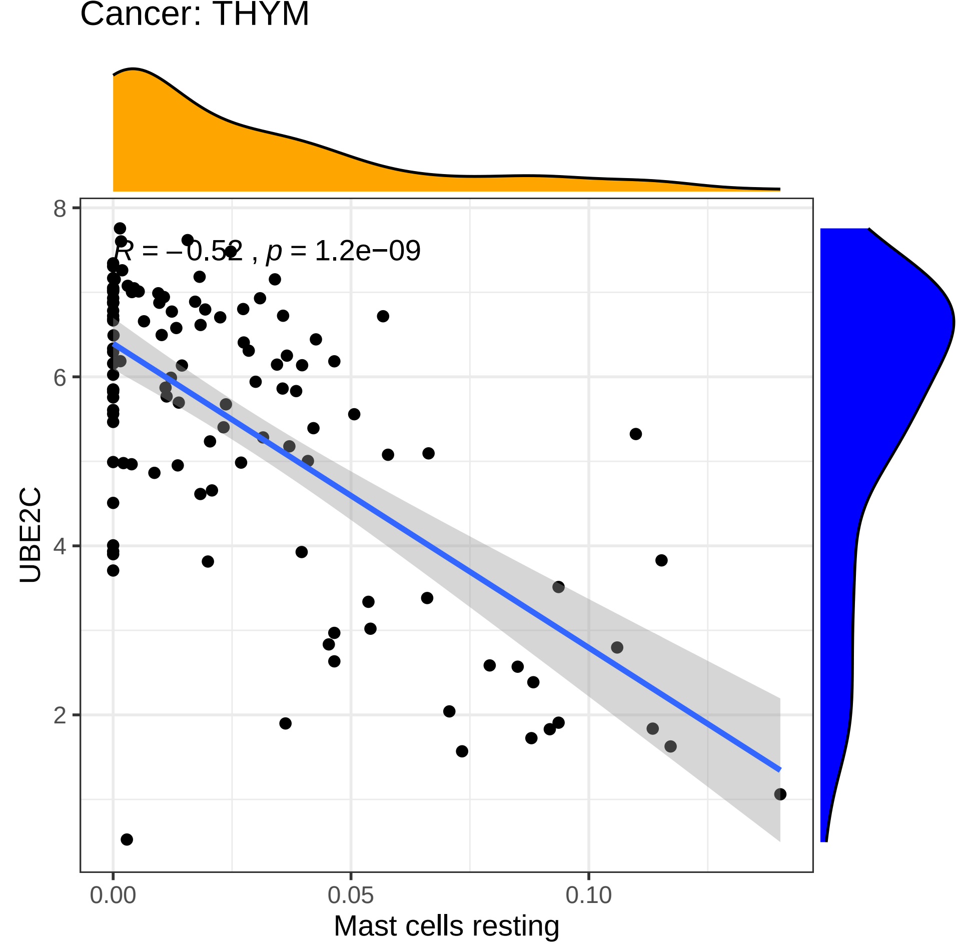

Cancer: THYM
R = −0.52 , p = 1.2e−09
8
6
4
2
UBE2C
0.00
0.05
0.10
Mast cells resting

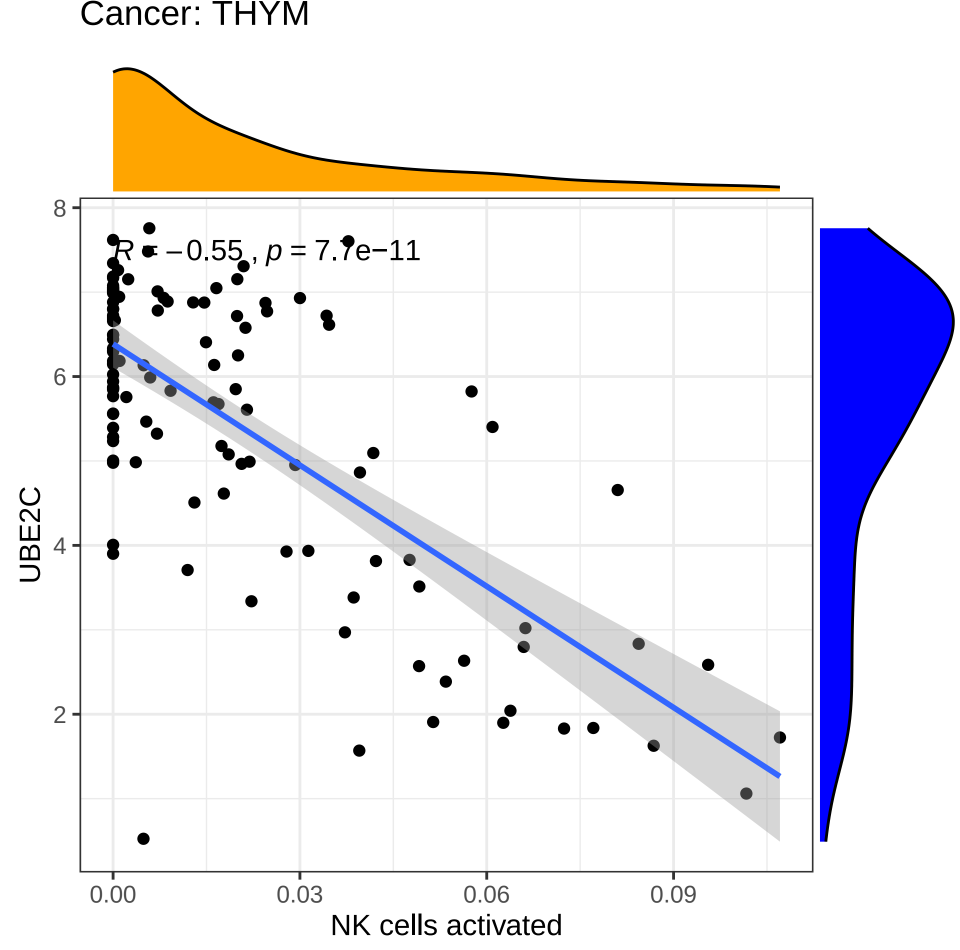

Cancer: THYM
R = −0.55 , p = 7.7e−11
UBE2C
8
6
4
2
0.00
0.03
0.06
0.09
NK cells activated

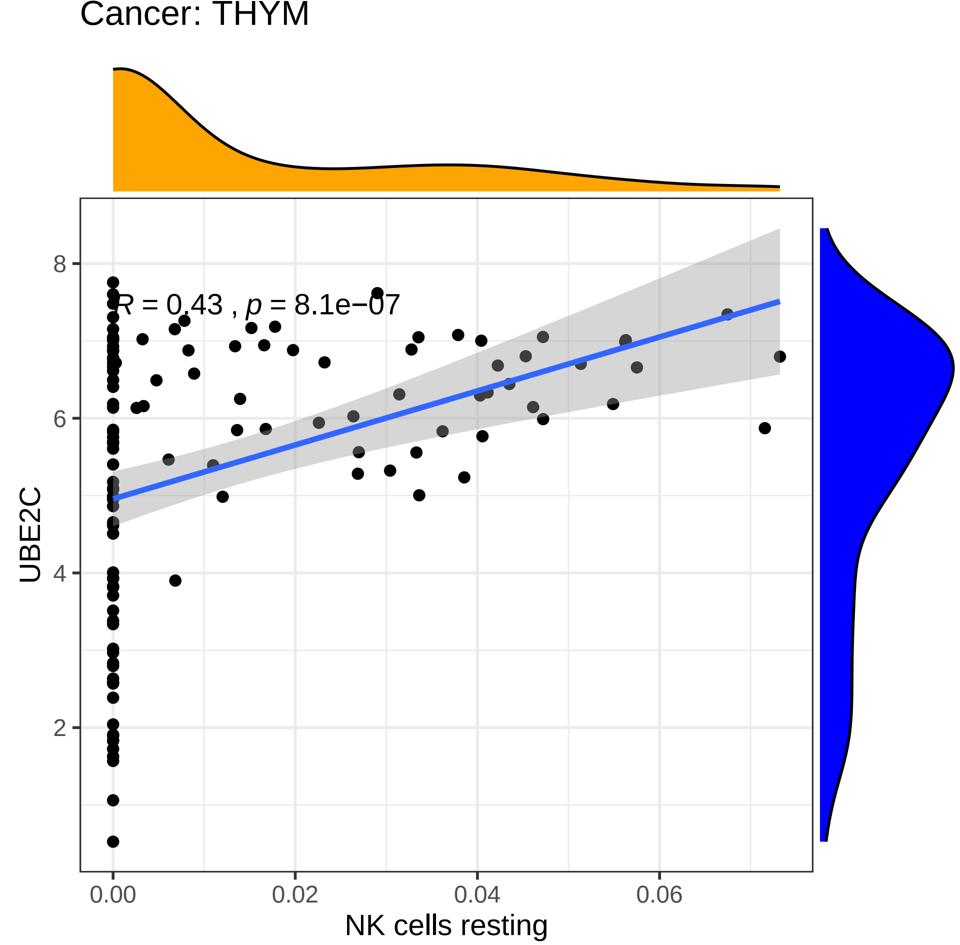
Cancer: THYM
R = 0.43 , p = 8.1e−07
8
6
4
2
UBE2C
0.00
0.02
0.04
0.06
NK cells resting

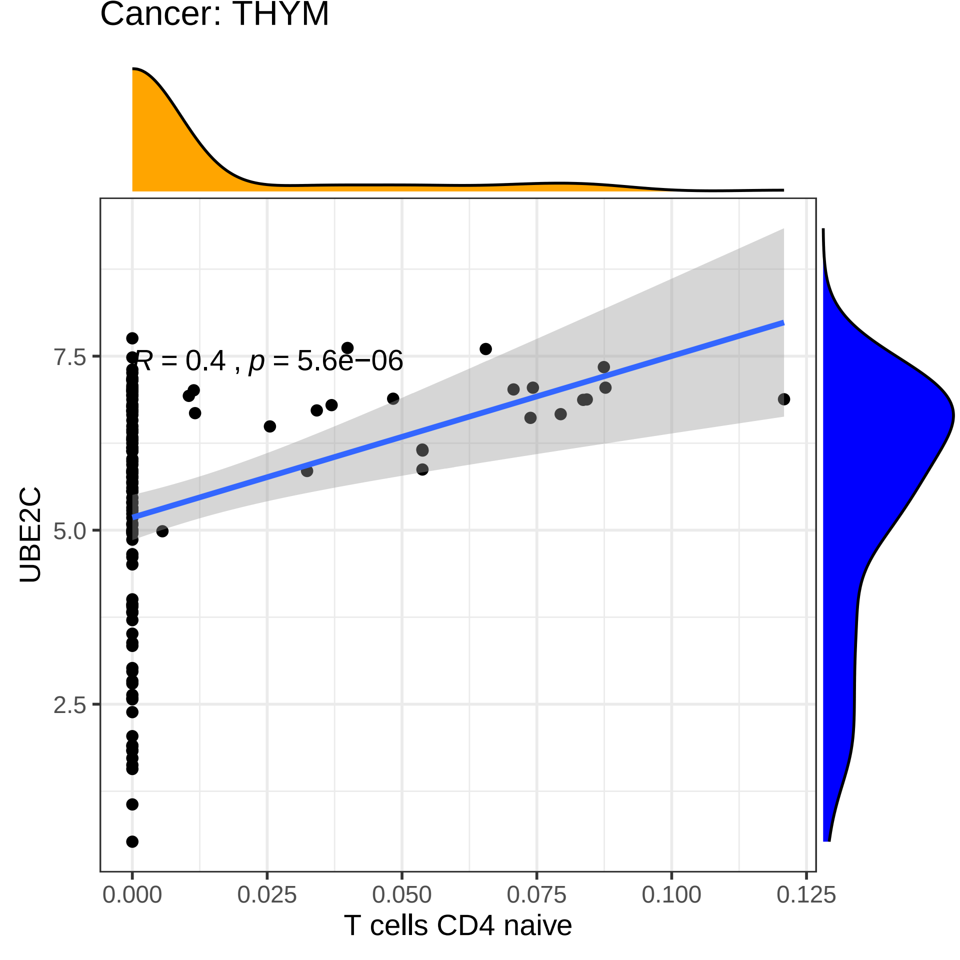
Cancer: THYM
R = 0.4 , p = 5.6e−06
UBE2C
7.5
5.0
2.5
0.000
0.025
0.050
0.075
0.100
0.125
T cells CD4 naive

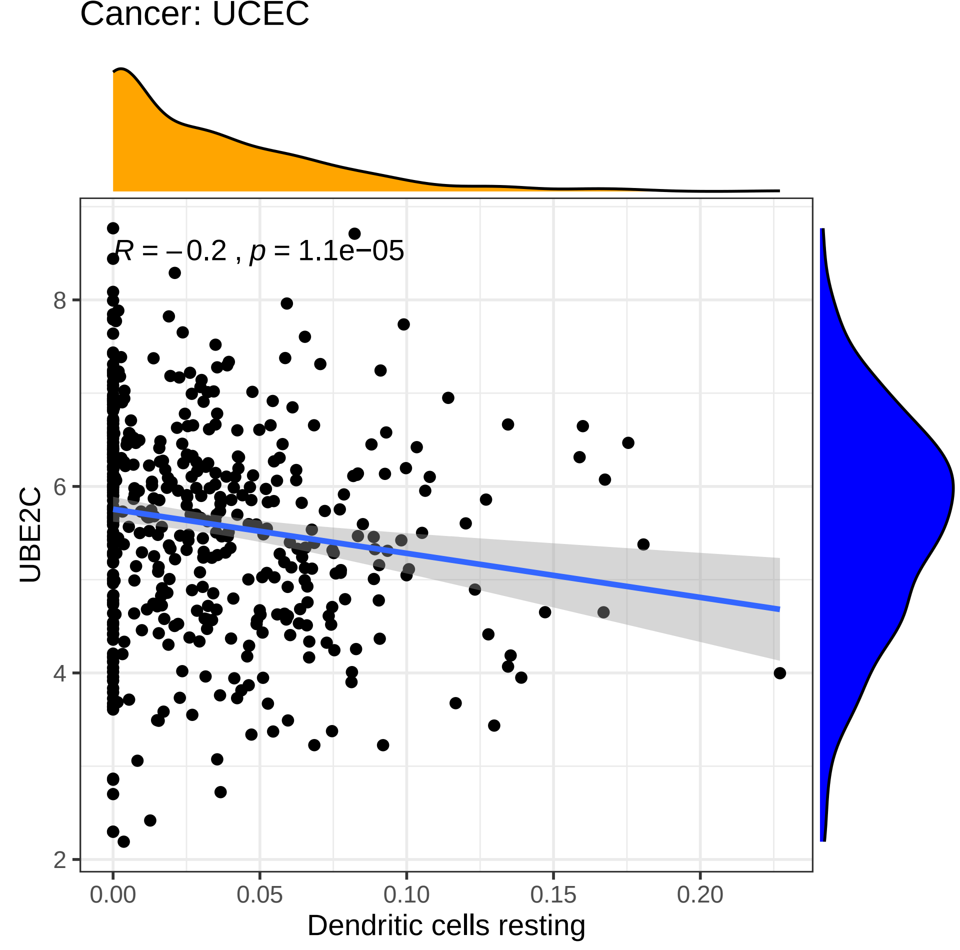

Cancer: UCEC
R = −0.2 , p = 1.1e−05
8
6
4
2
UBE2C
0.00
0.05
0.10
0.15
0.20
Dendritic cells resting

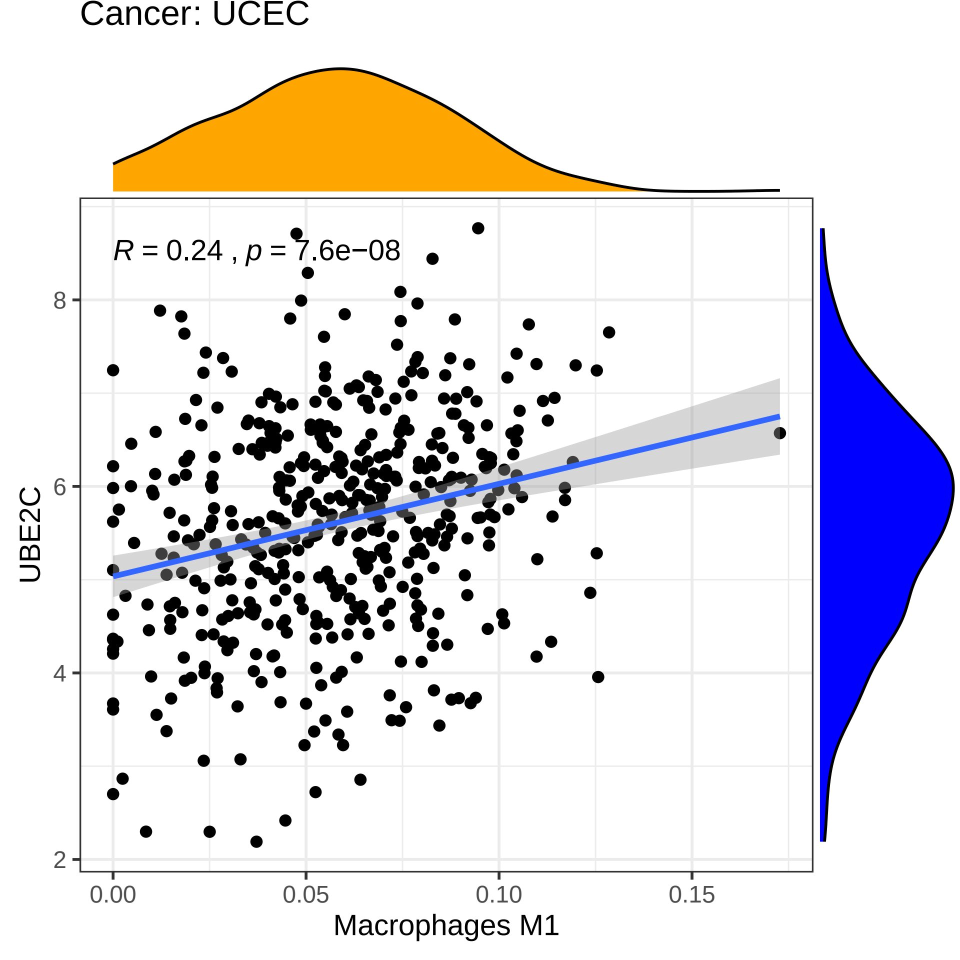

Cancer: UCEC
R = 0.24 , p = 7.6e−08
UBE2C
8
6
4
2
0.00
0.05
0.10
0.15
Macrophages M1

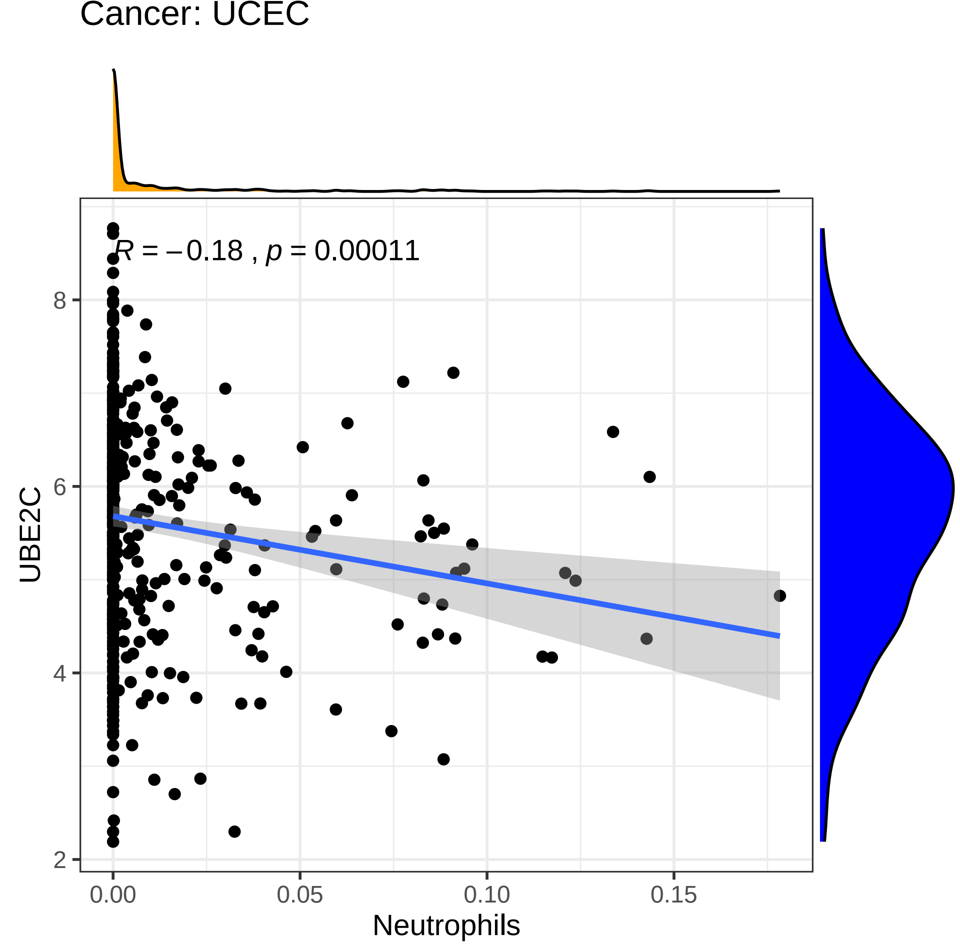

Cancer: UCEC
R = −0.18 , p = 0.00011
UBE2C
Neutrophils
8
6
4
2
0.00
0.05
0.10
0.15

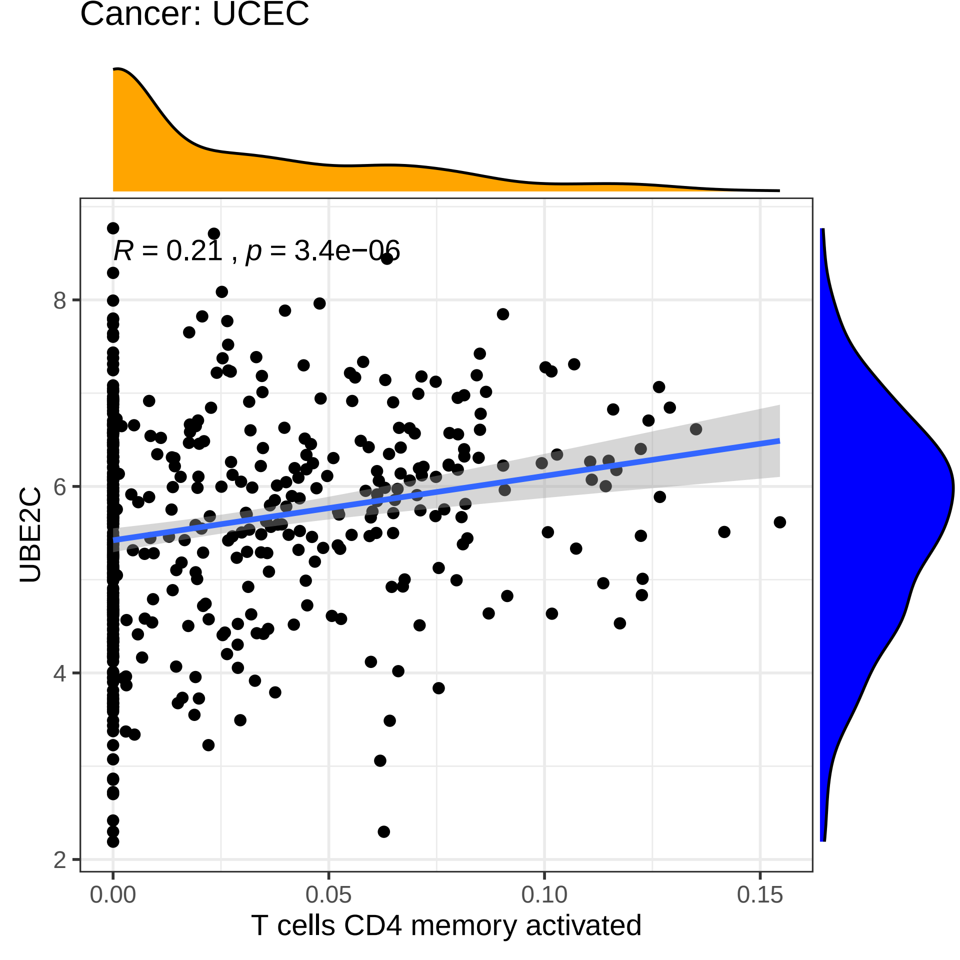
Cancer: UCEC
R = 0.21 , p = 3.4e−06
UBE2C
T cells CD4 memory activated
2
4
6
8
0.00
0.05
0.10
0.15

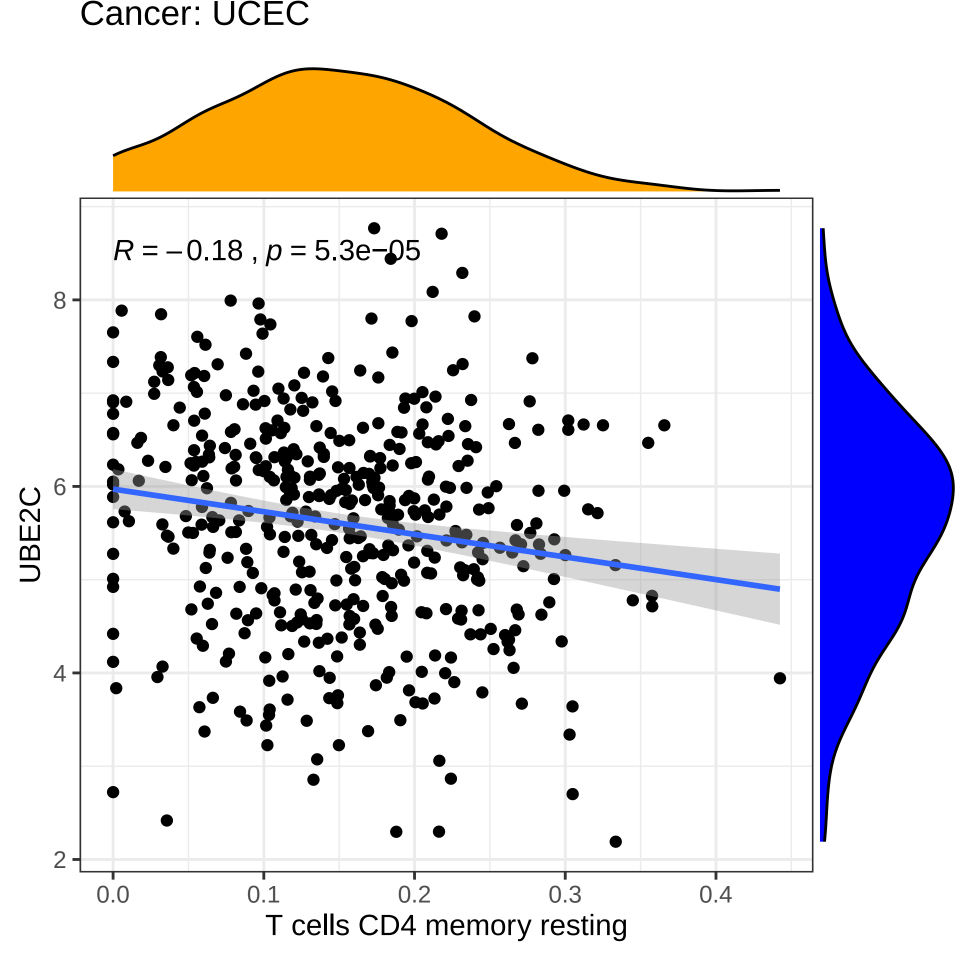
Cancer: UCEC
R = −0.18 , p = 5.3e−05
8
6
4
2
UBE2C
0.0
0.1
0.2
0.3
0.4
T cells CD4 memory resting

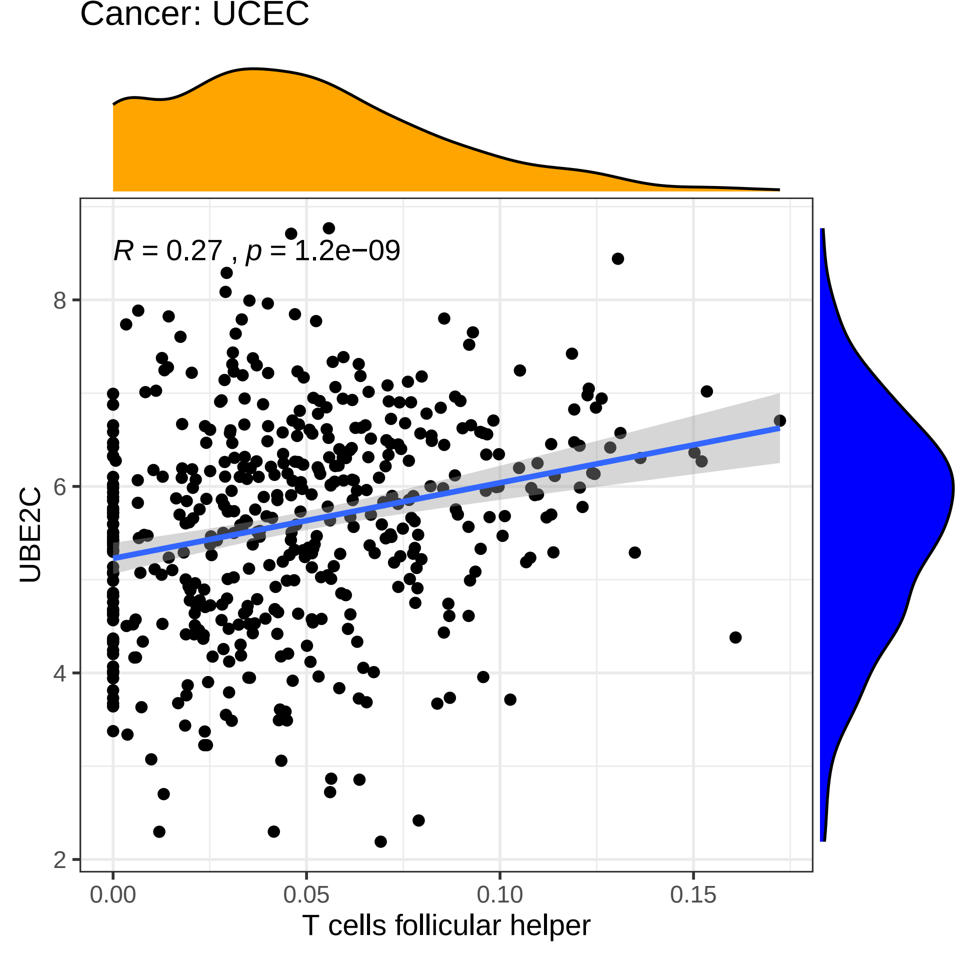

Cancer: UCEC
R = 0.27 , p = 1.2e−09
UBE2C
2
4
6
8
0.00
0.05
0.10
0.15
T cells follicular helper

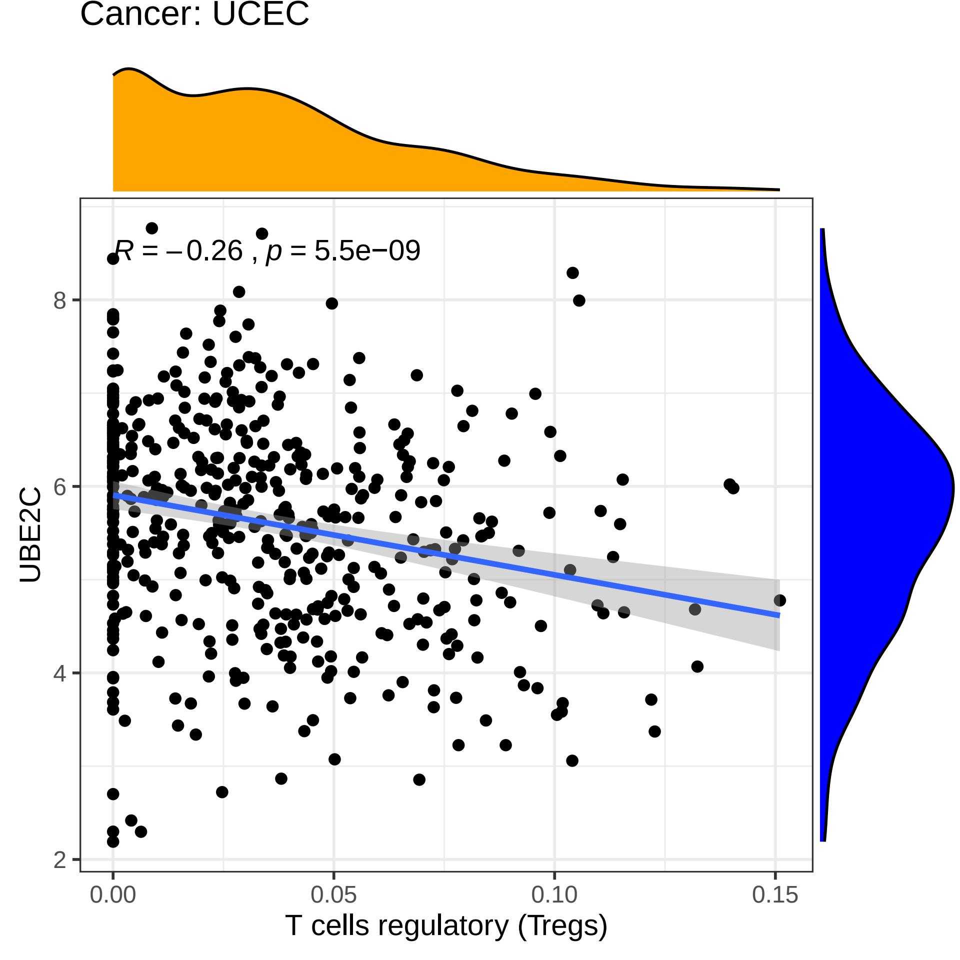
Cancer: UCEC
R = −0.26 , p = 5.5e−09
UBE2C
2
4
6
8
0.00
0.05
0.10
0.15
T cells regulatory (Tregs)

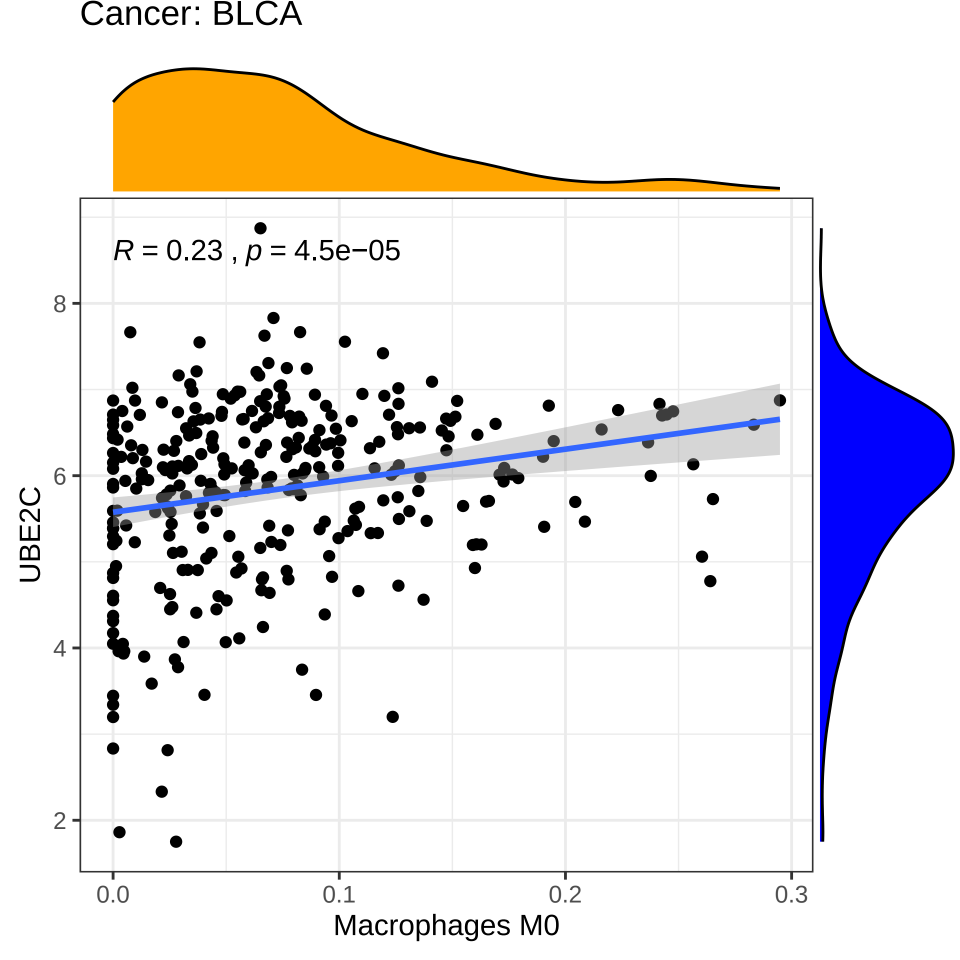
Cancer: BLCA
R = 0.23 , p = 4.5e−05
UBE2C
2
4
6
8
0.0
0.1
0.2
0.3
Macrophages M0

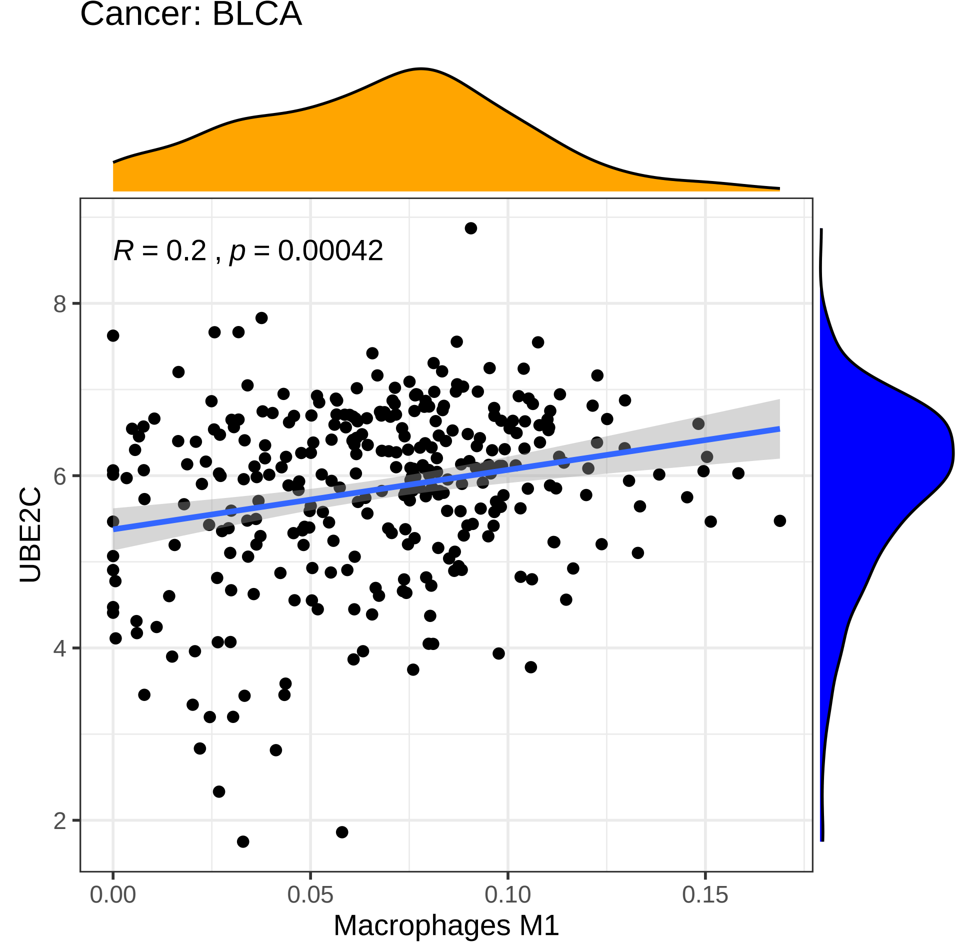

Cancer: BLCA
R = 0.2 , p = 0.00042
UBE2C
2
4
6
8
0.00
0.05
0.10
0.15
Macrophages M1

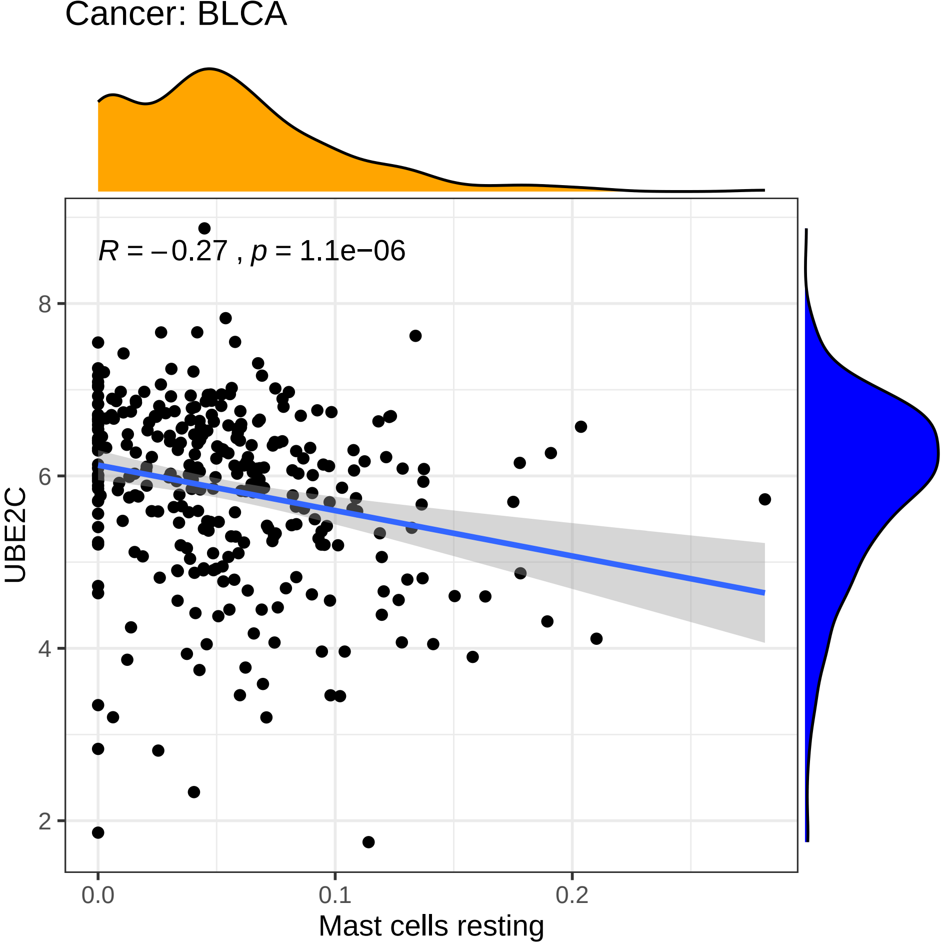

Cancer: BLCA
R = −0.27 , p = 1.1e−06
UBE2C
8
6
4
2
0.0
0.1
0.2
Mast cells resting

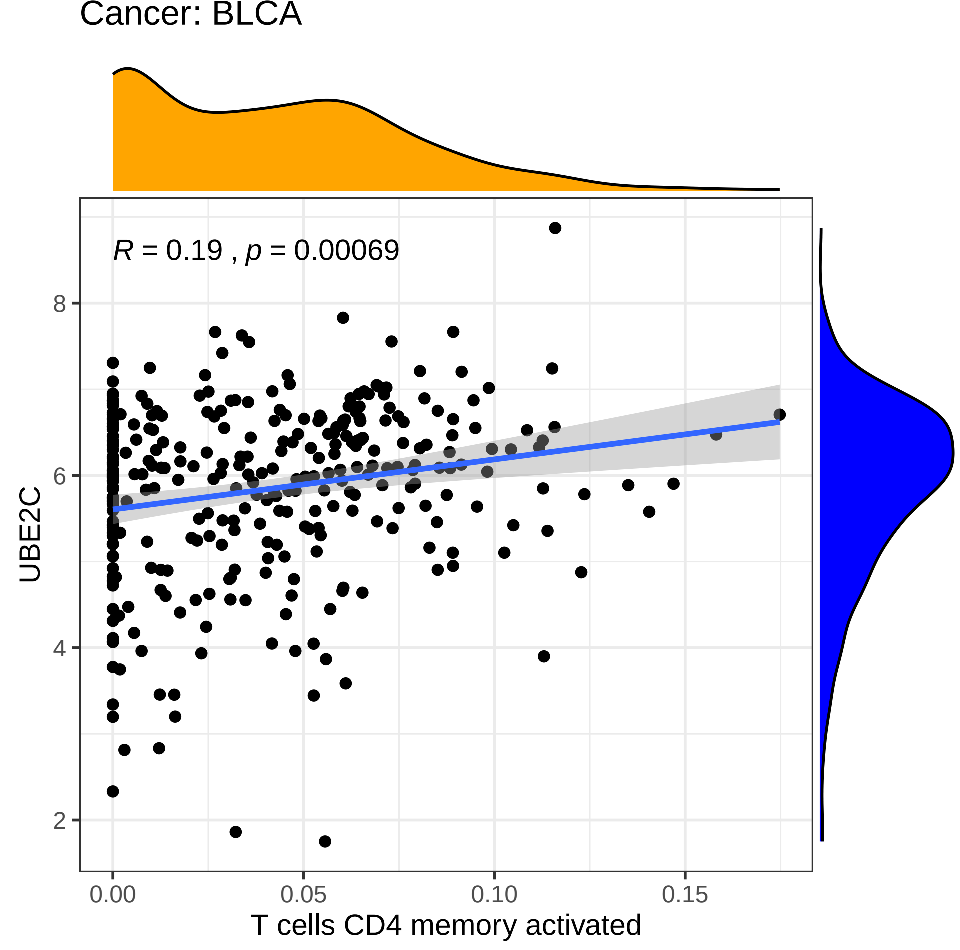
Cancer: BLCA
R = 0.19 , p = 0.00069
UBE2C
2
4
6
8
0.00
0.05
0.10
0.15
T cells CD4 memory activated

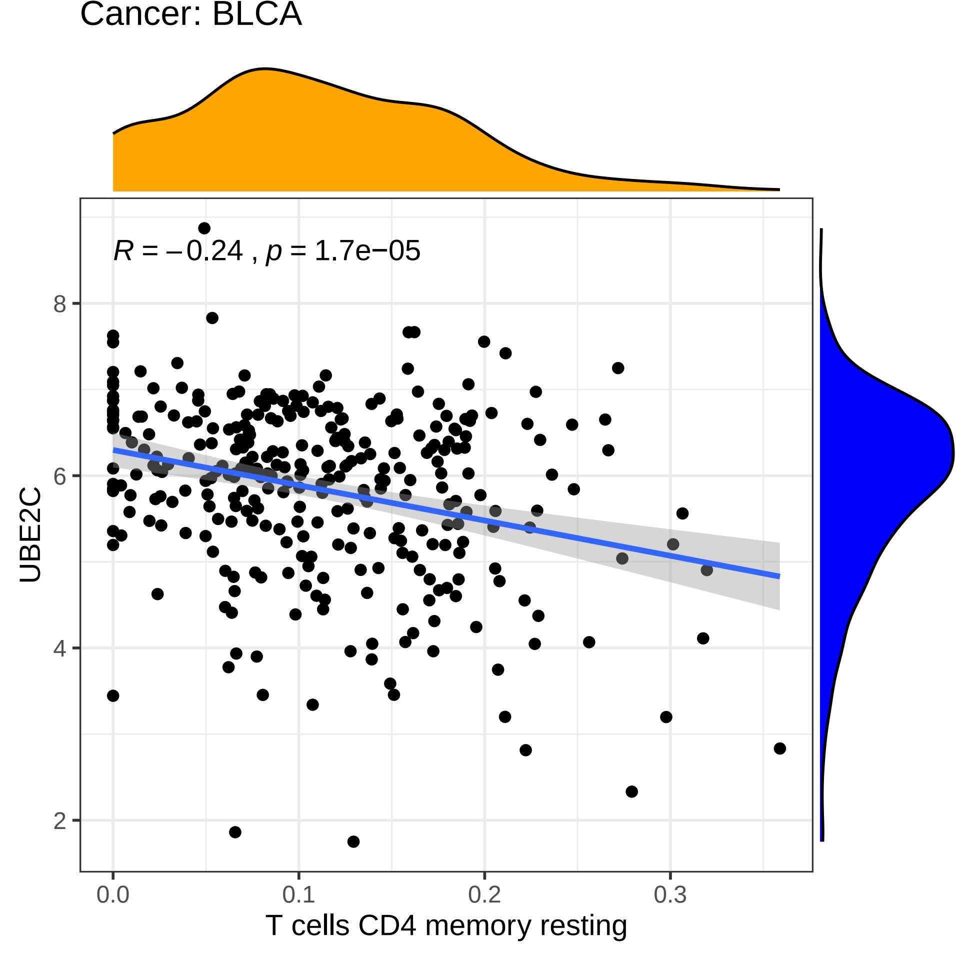
Cancer: BLCA
R = −0.24 , p = 1.7e−05
UBE2C
8
6
4
2
0.0
0.1
0.2
0.3
T cells CD4 memory resting

Cancer: BLCA

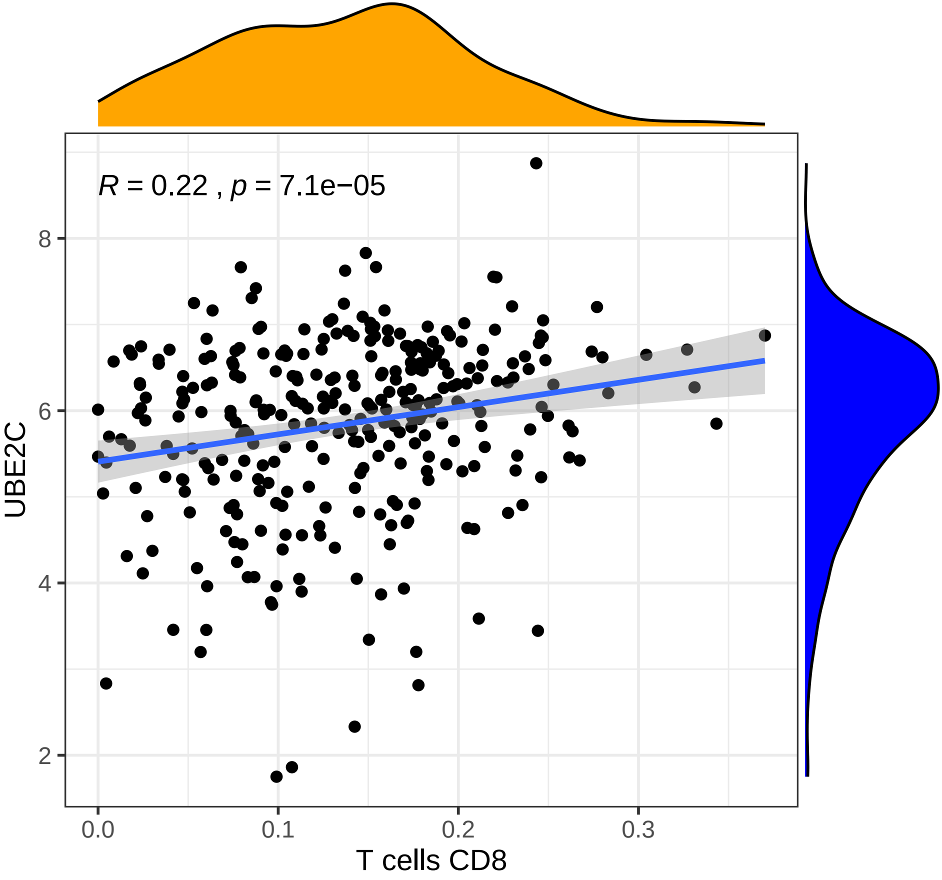

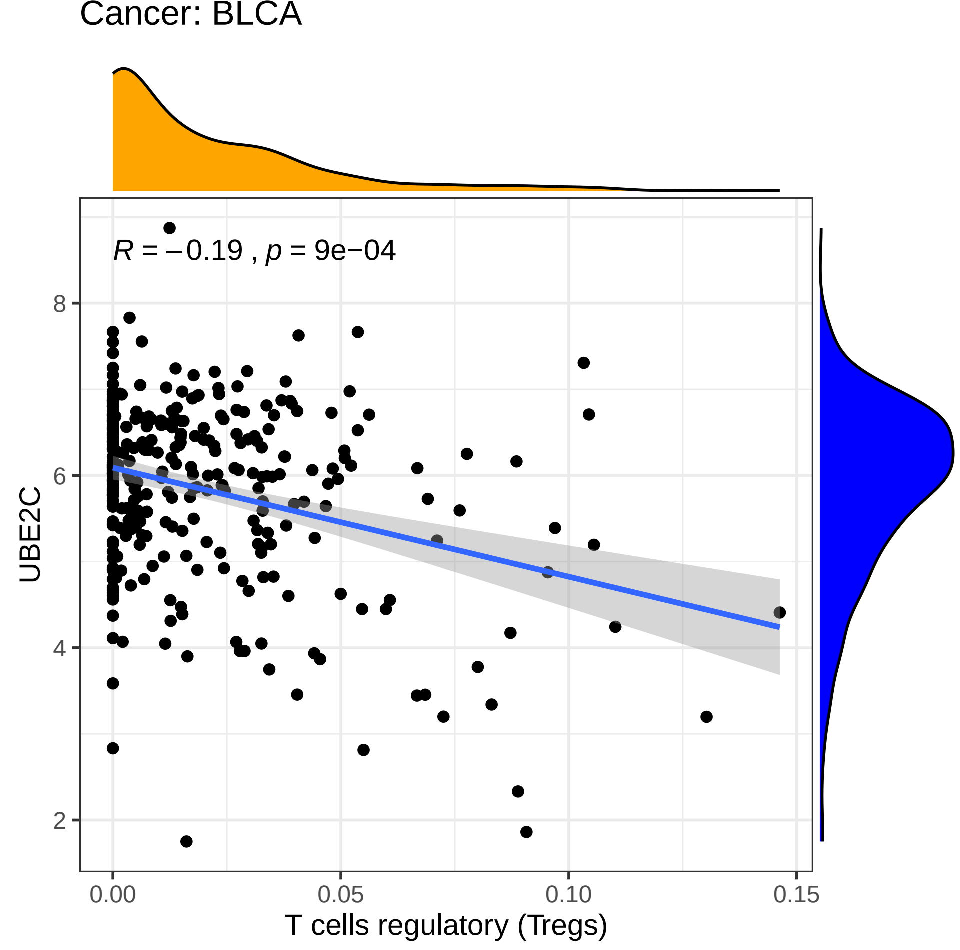
Cancer: BLCA
R = −0.19 , p = 9e−04
UBE2C
8
6
4
2
0.00
0.05
0.10
0.15
T cells regulatory (Tregs)

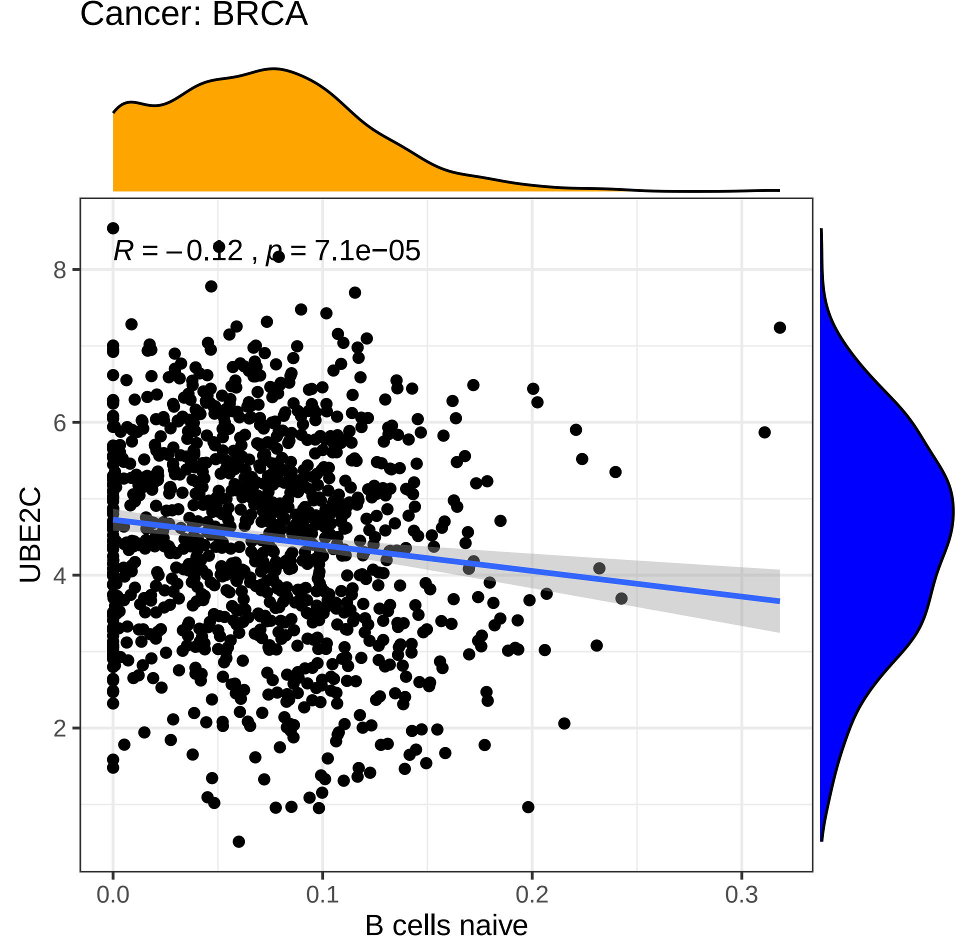

Cancer: BRCA
R = −0.12 , p = 7.1e−05
UBE2C
2
4
6
8
0.0
0.1
0.2
0.3
B cells naive

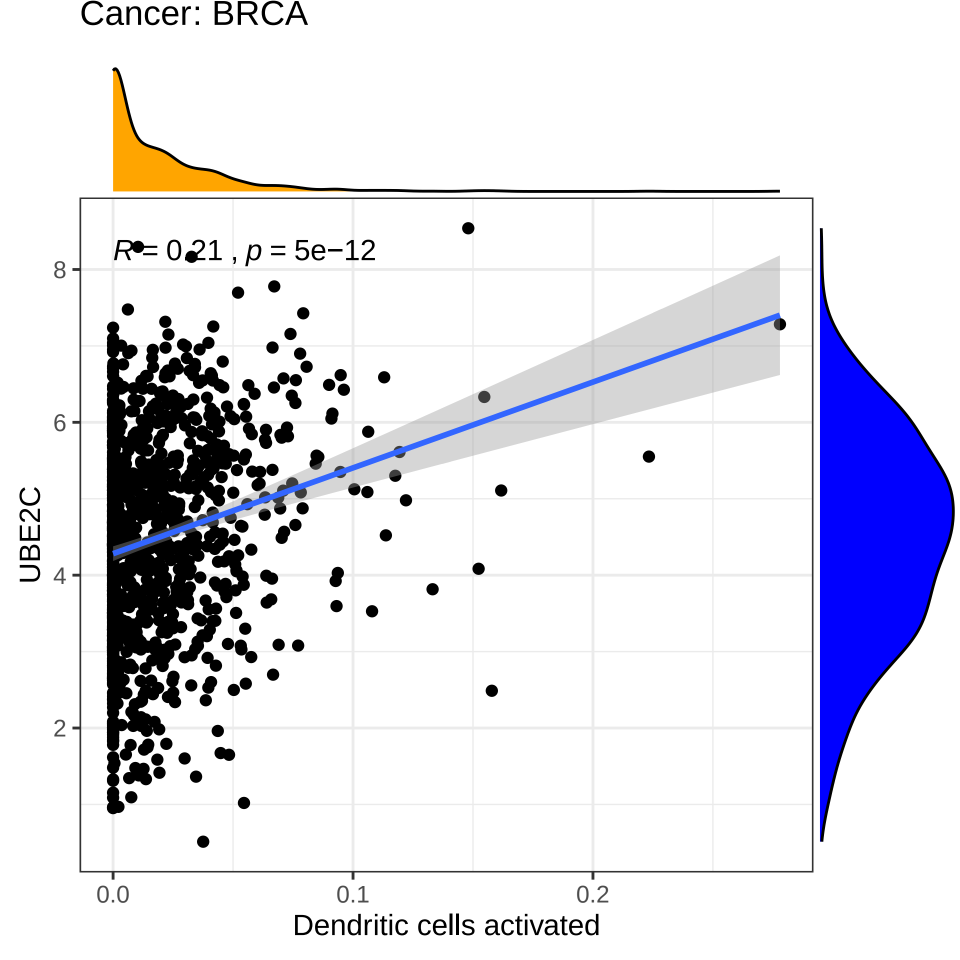
Cancer: BRCA
R = 0.21 , p = 5e−12
UBE2C
8
6
4
2
0.0
0.1
0.2
Dendritic cells activated

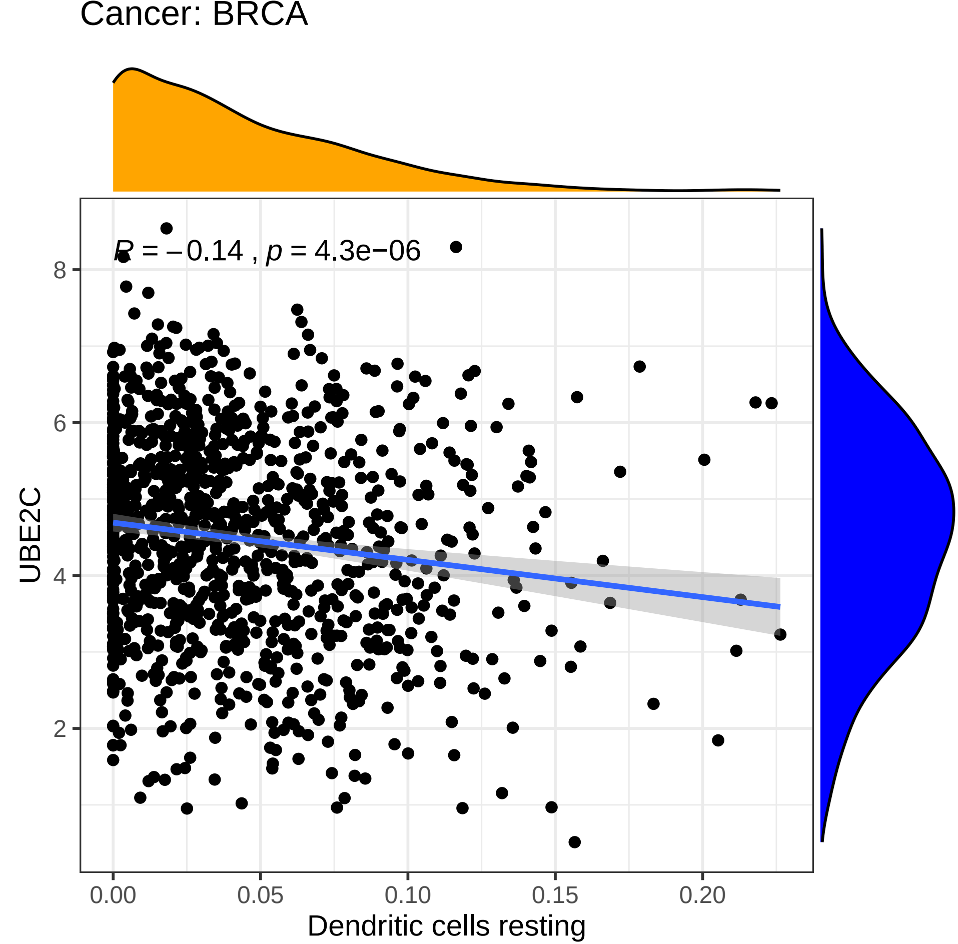

Cancer: BRCA
R = −0.14 , p = 4.3e−06
8
6
4
2
UBE2C
0.00
0.05
0.10
0.15
0.20
Dendritic cells resting

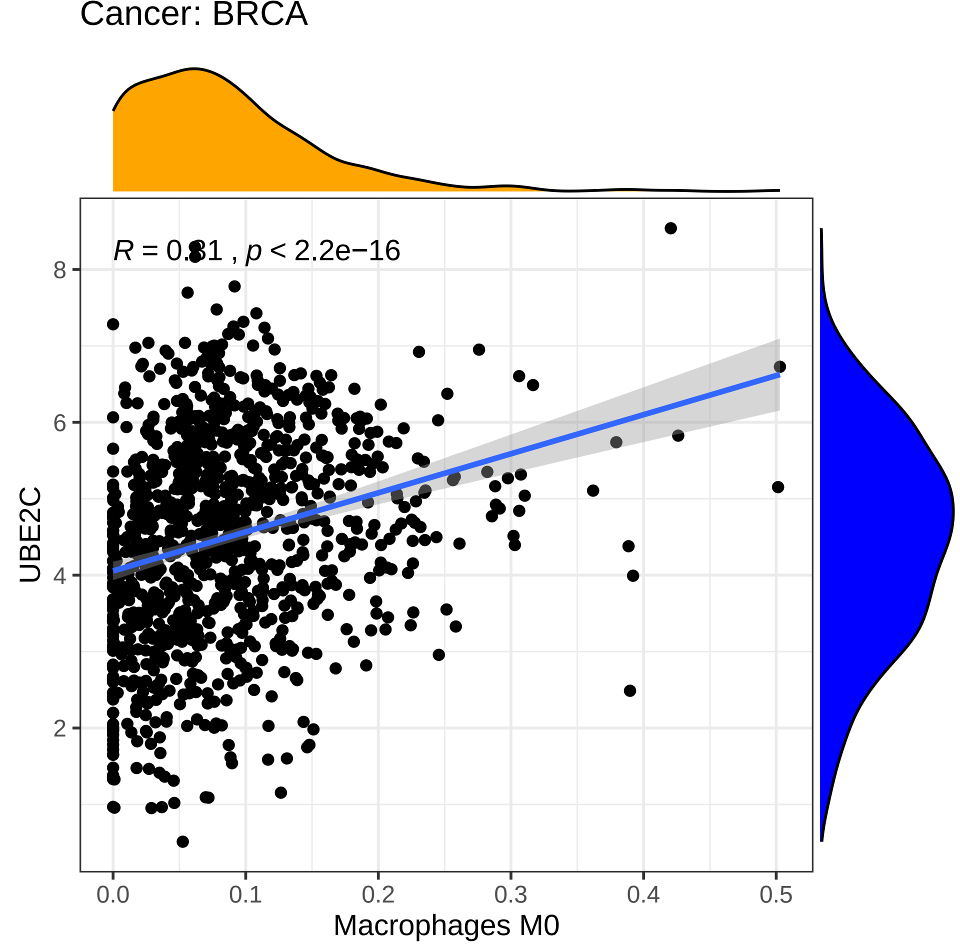

Cancer: BRCA
R = 0.31 , p < 2.2e−16
UBE2C
8
6
4
2
0.0
0.1
0.2
0.3
0.4
0.5
Macrophages M0

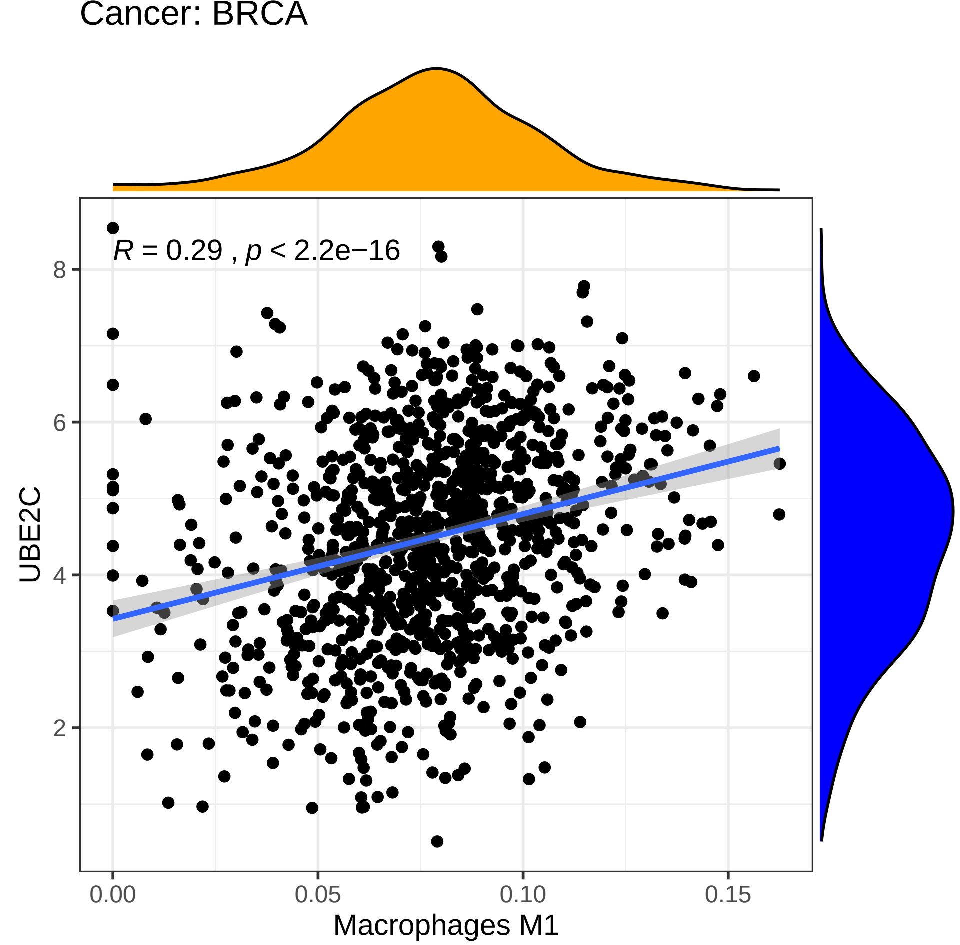

Cancer: BRCA
R = 0.29 , p < 2.2e−16
8
6
4
2
UBE2C
0.00
0.05
0.10
0.15
Macrophages M1

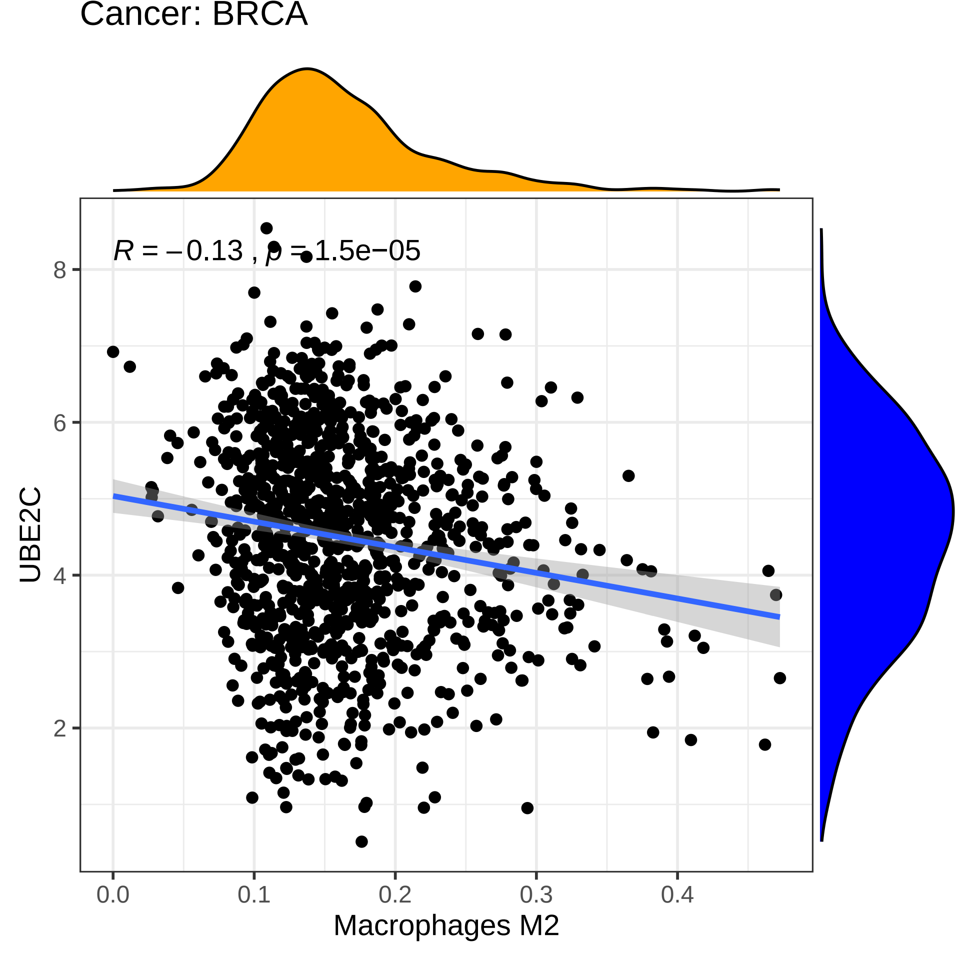

Cancer: BRCA
R = −0.13 , p = 1.5e−05
8
6
4
2
UBE2C
0.0
0.1
0.2
0.3
0.4
Macrophages M2

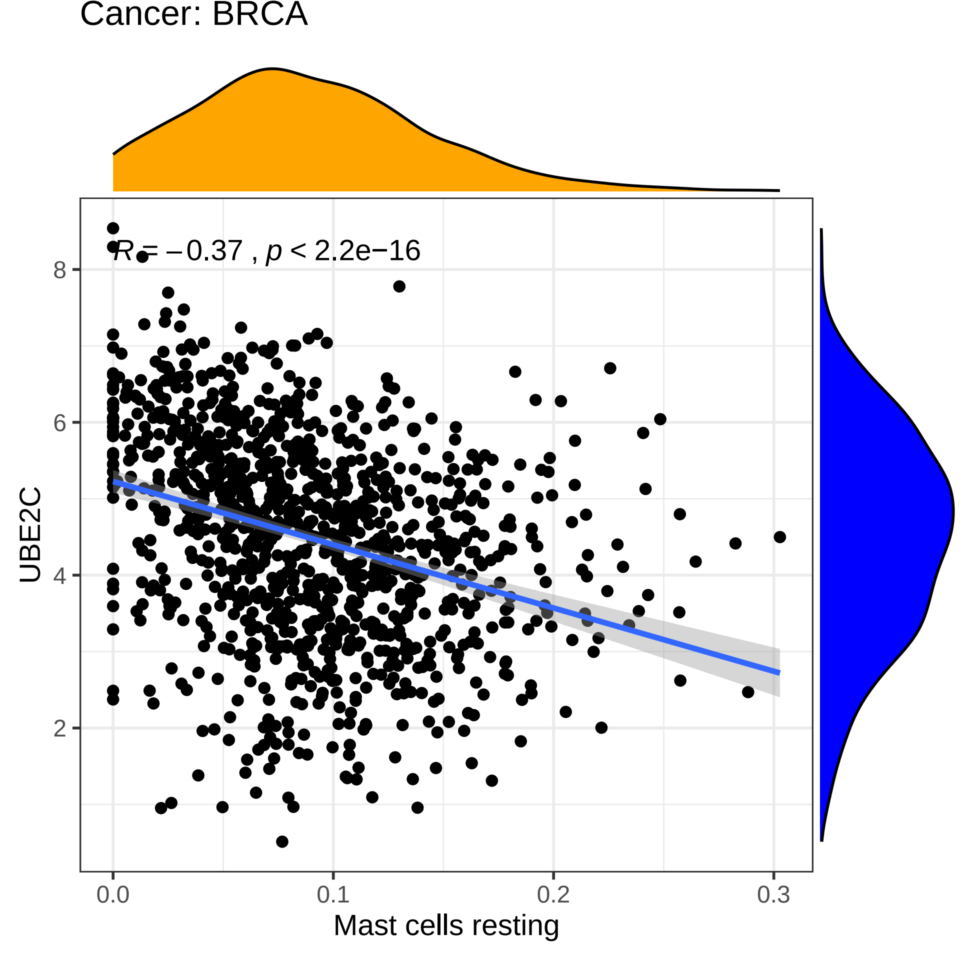
Cancer: BRCA
R = −0.37 , p < 2.2e−16
UBE2C
8
6
4
2
0.0
0.1
0.2
0.3
Mast cells resting

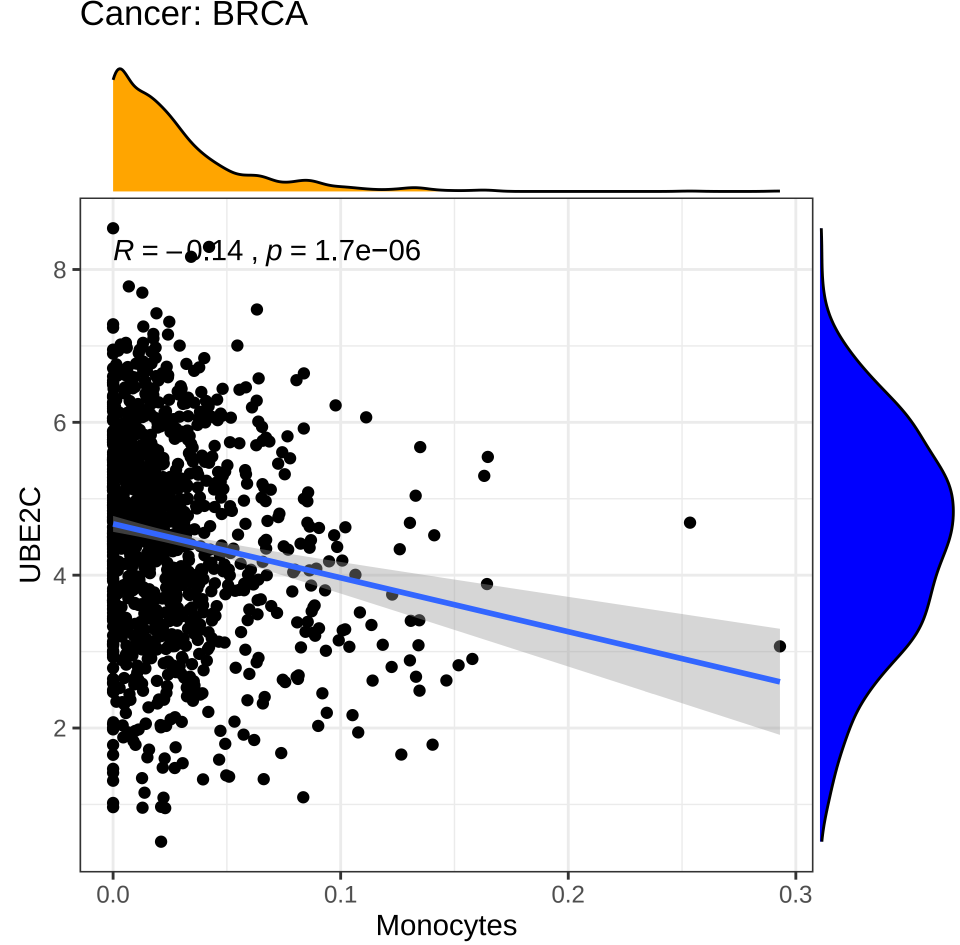

Cancer: BRCA
R = −0.14 , p = 1.7e−06
UBE2C
8
6
4
2
0.0
0.1
0.2
0.3
Monocytes

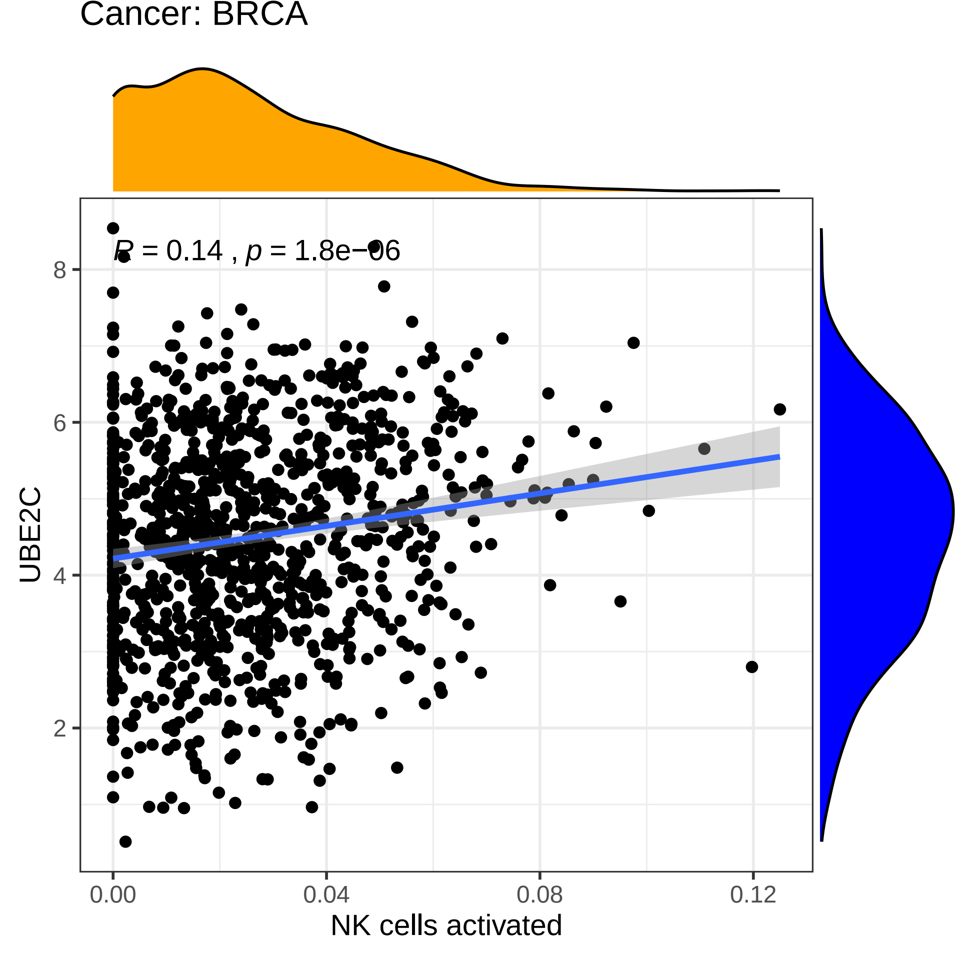
Cancer: BRCA
R = 0.14 , p = 1.8e−06
UBE2C
8
6
4
2
0.00
0.04
0.08
0.12
NK cells activated

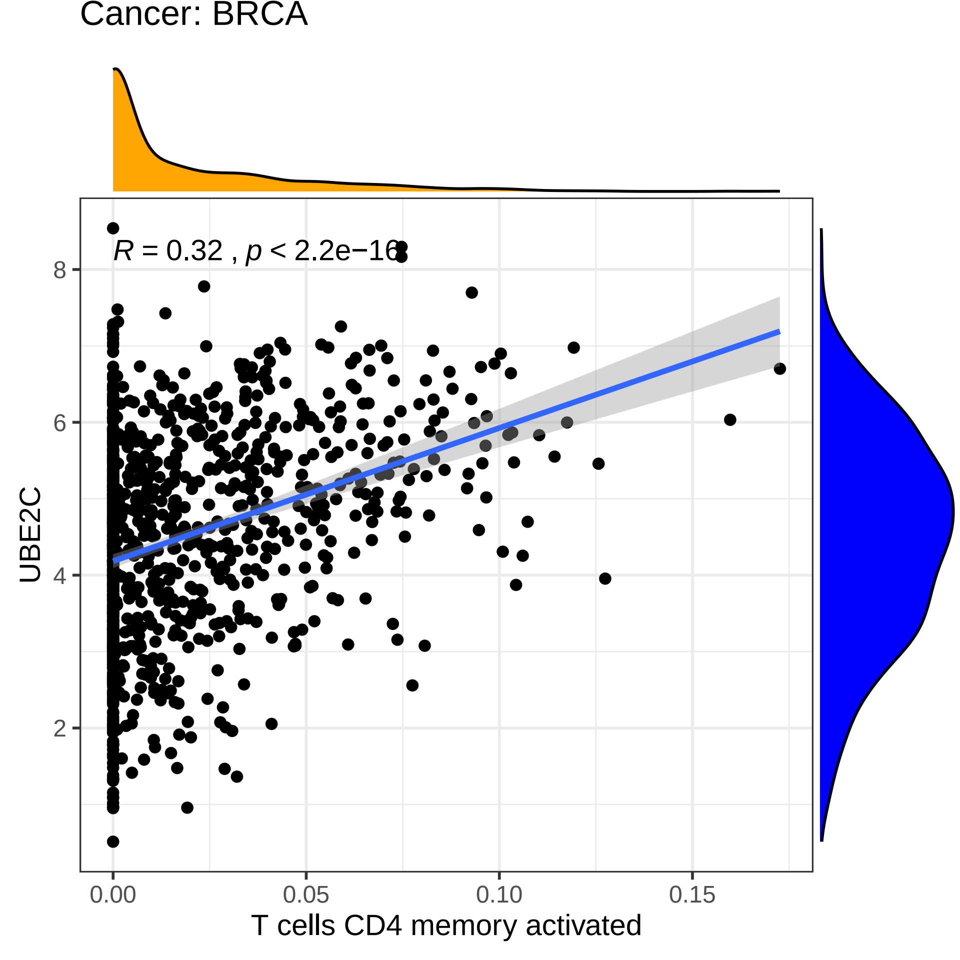

Cancer: BRCA
R = 0.32 , p < 2.2e−16
UBE2C
2
4
6
8
0.00
0.05
0.10
0.15
T cells CD4 memory activated

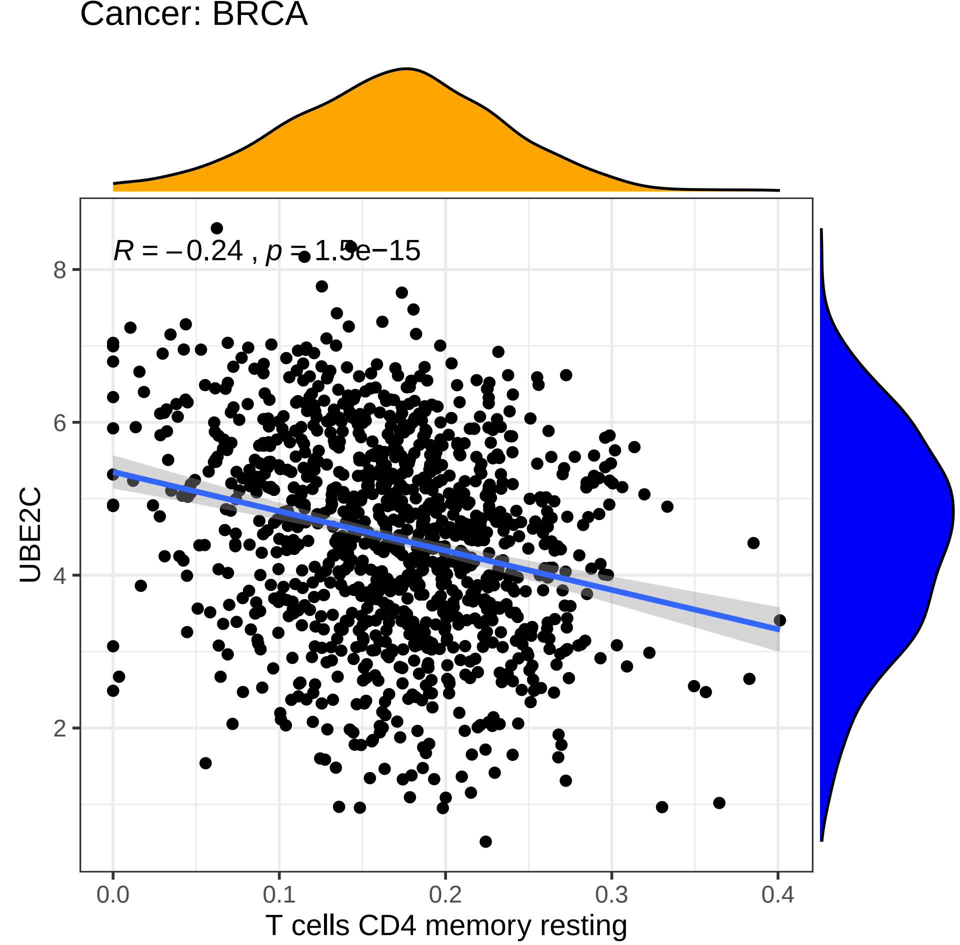

Cancer: BRCA
R = −0.24 , p = 1.5e−15
UBE2C
2
4
6
8
0.0
0.1
0.2
0.3
0.4
T cells CD4 memory resting

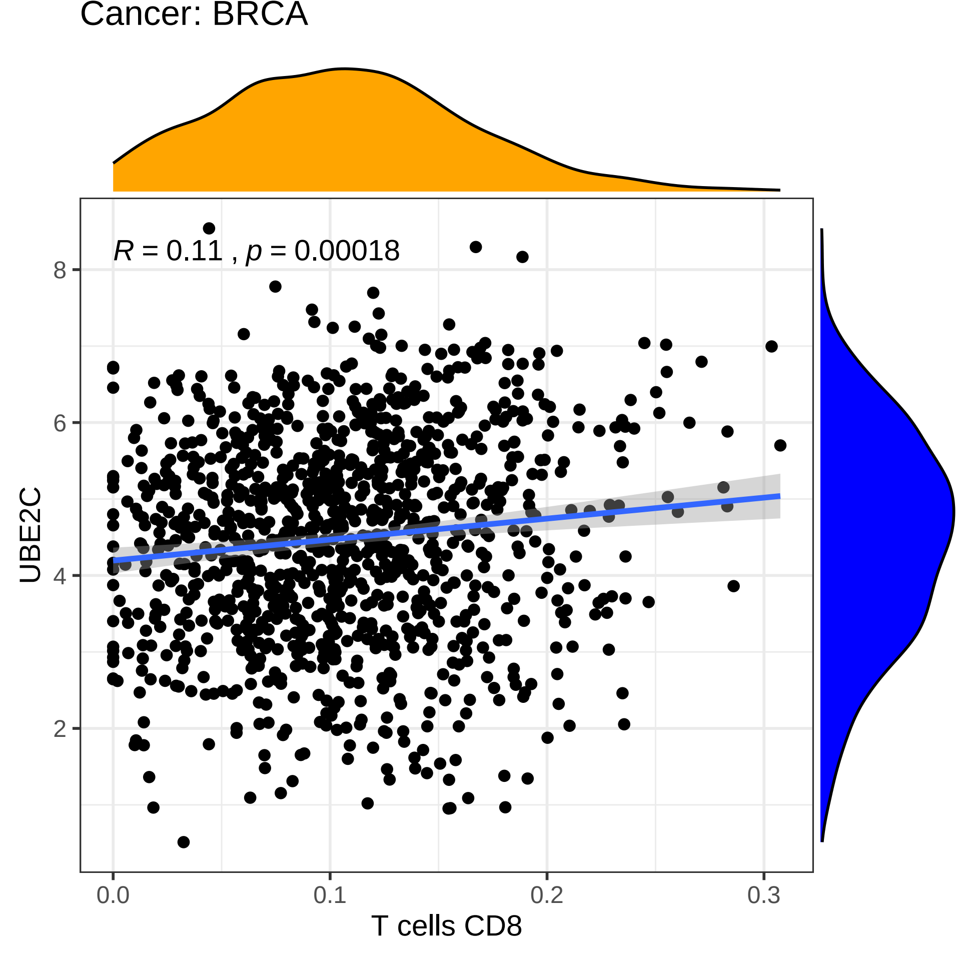
Cancer: BRCA
R = 0.11 , p = 0.00018
UBE2C
2
4
6
8
0.0
0.1
0.2
0.3
T cells CD8

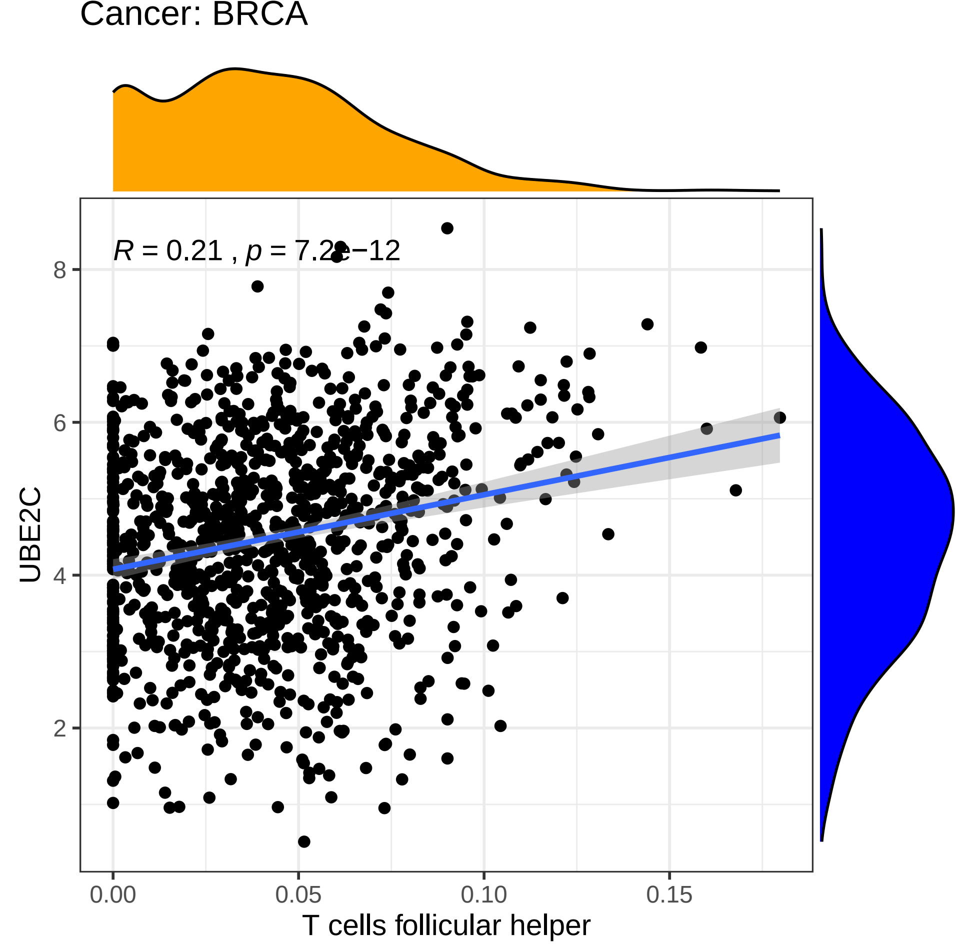
Cancer: BRCA
R = 0.21 , p = 7.2e−12
UBE2C
2
4
6
8
0.00
0.05
0.10
0.15
T cells follicular helper

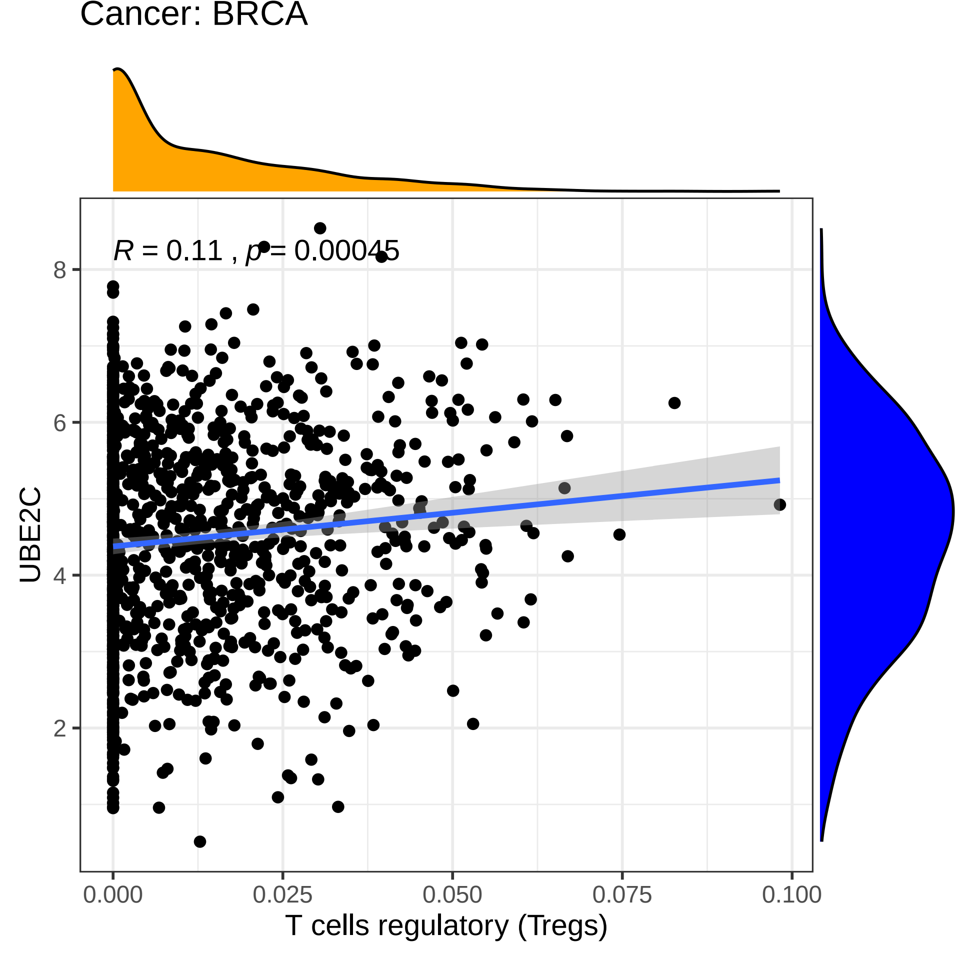

Cancer: BRCA
R = 0.11 , p = 0.00045
8
6
4
2
UBE2C
0.000
0.025
0.050
0.075
0.100
T cells regulatory (Tregs)

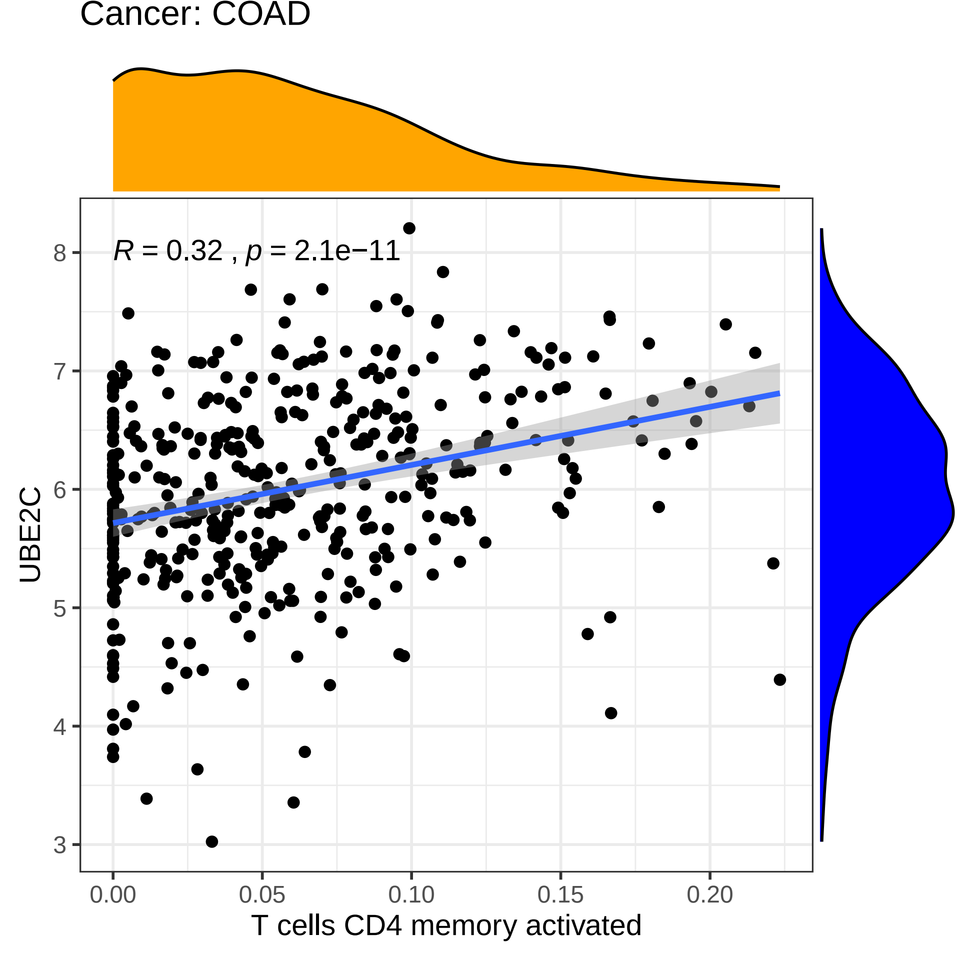

Cancer: COAD
R = 0.32 , p = 2.1e−11
UBE2C
3
4
5
6
7
8
0.00
0.05
0.10
0.15
0.20
T cells CD4 memory activated

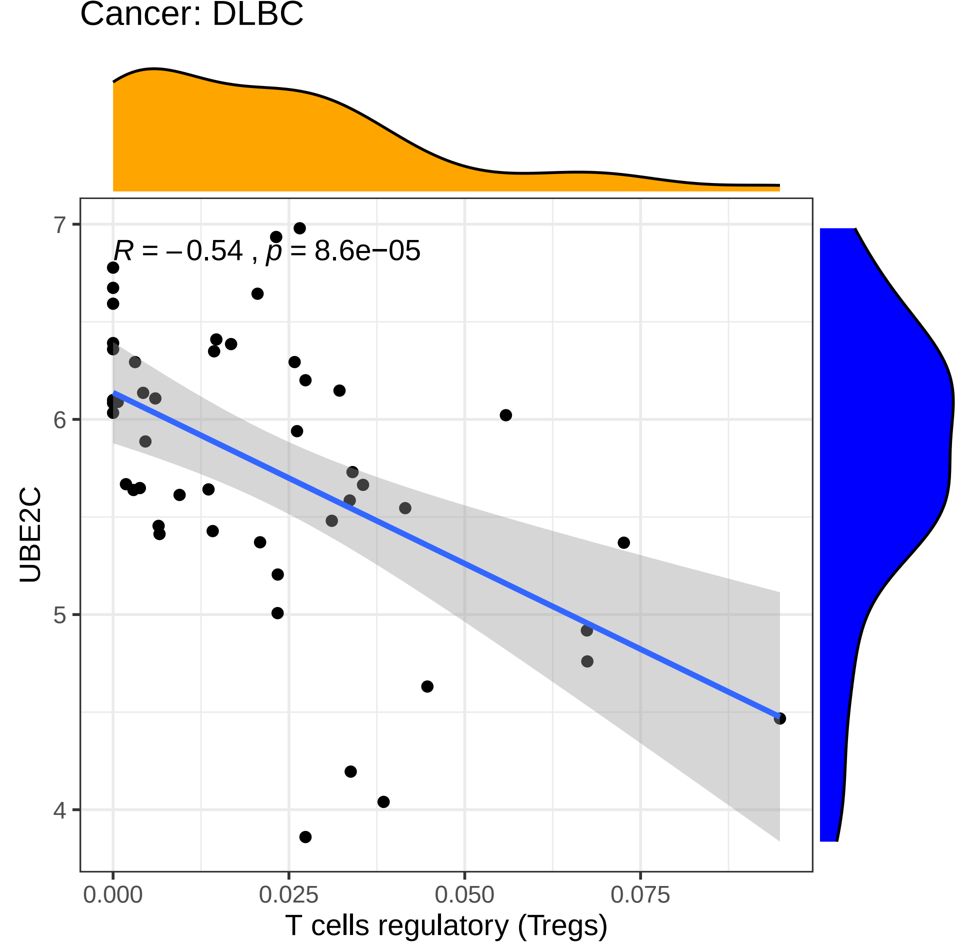

Cancer: DLBC
R = −0.54 , p = 8.6e−05
7
6
5
4
UBE2C
0.000
0.025
0.050
0.075
T cells regulatory (Tregs)

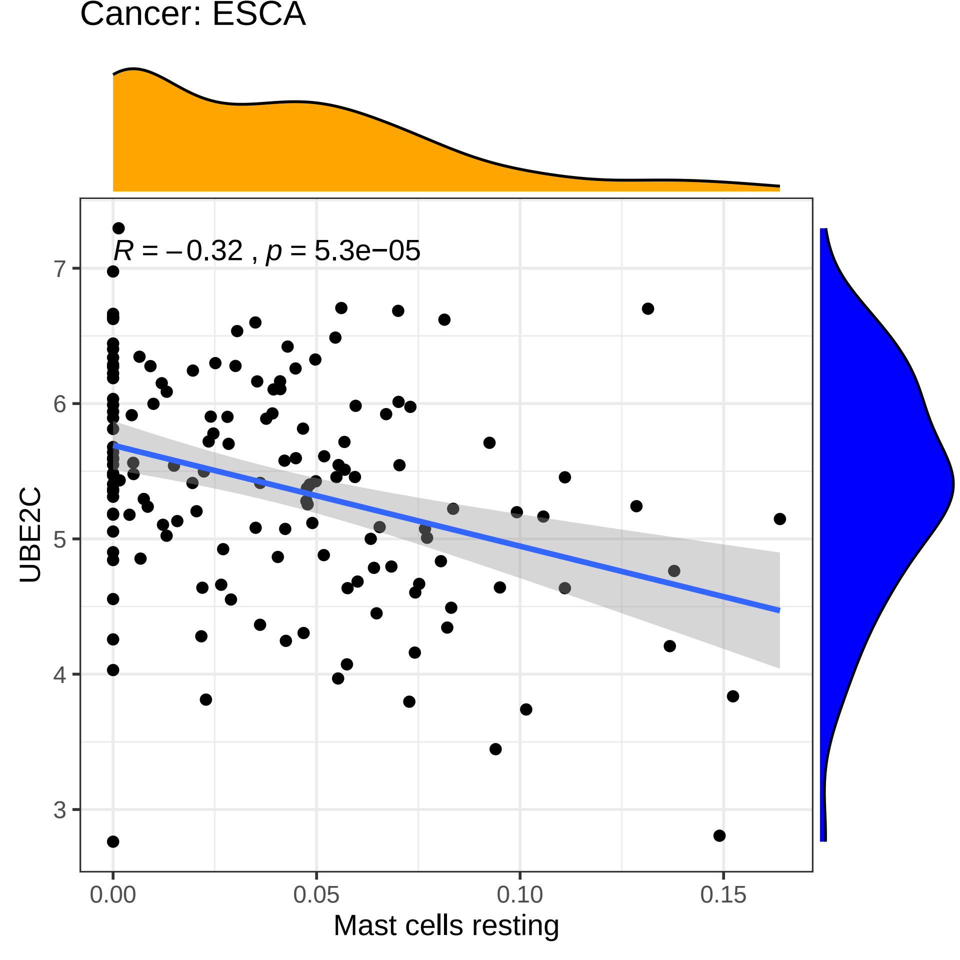

Cancer: ESCA
R = −0.32 , p = 5.3e−05
UBE2C
3
4
5
6
7
0.00
0.05
0.10
0.15
Mast cells resting

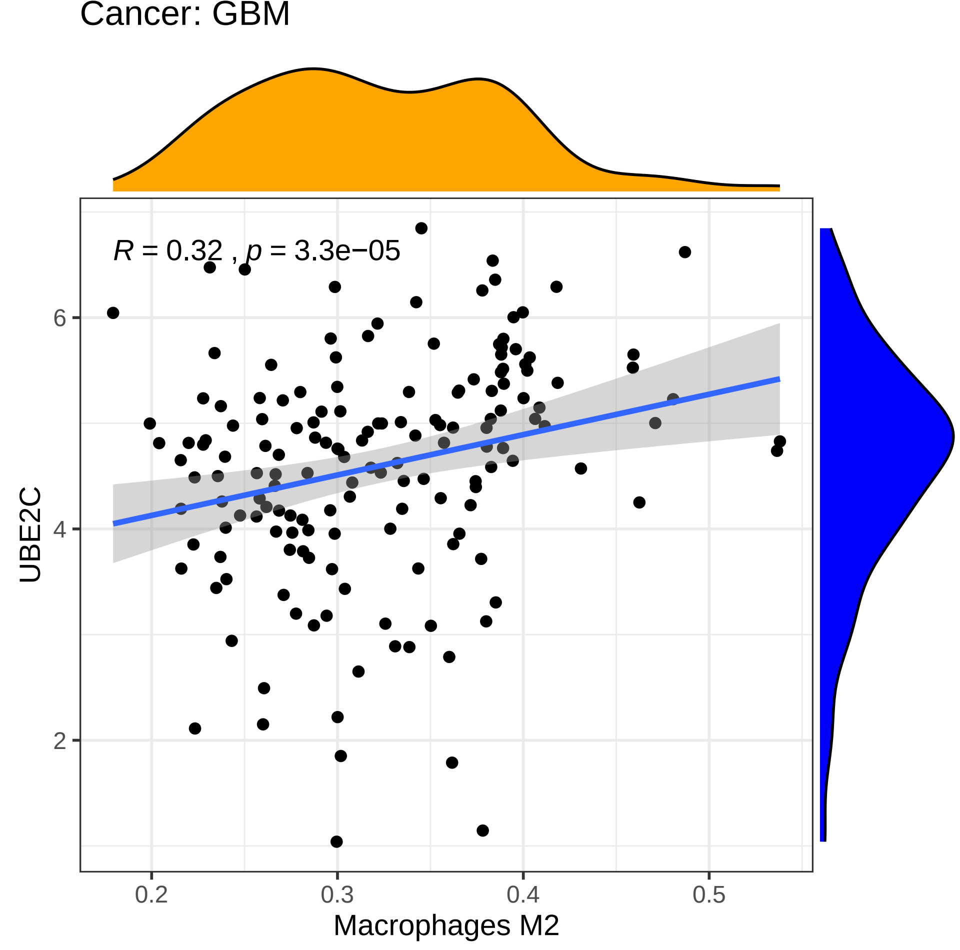
Cancer: GBM
R = 0.32 , p = 3.3e−05
UBE2C
6
4
2
0.2
0.3
0.4
0.5
Macrophages M2

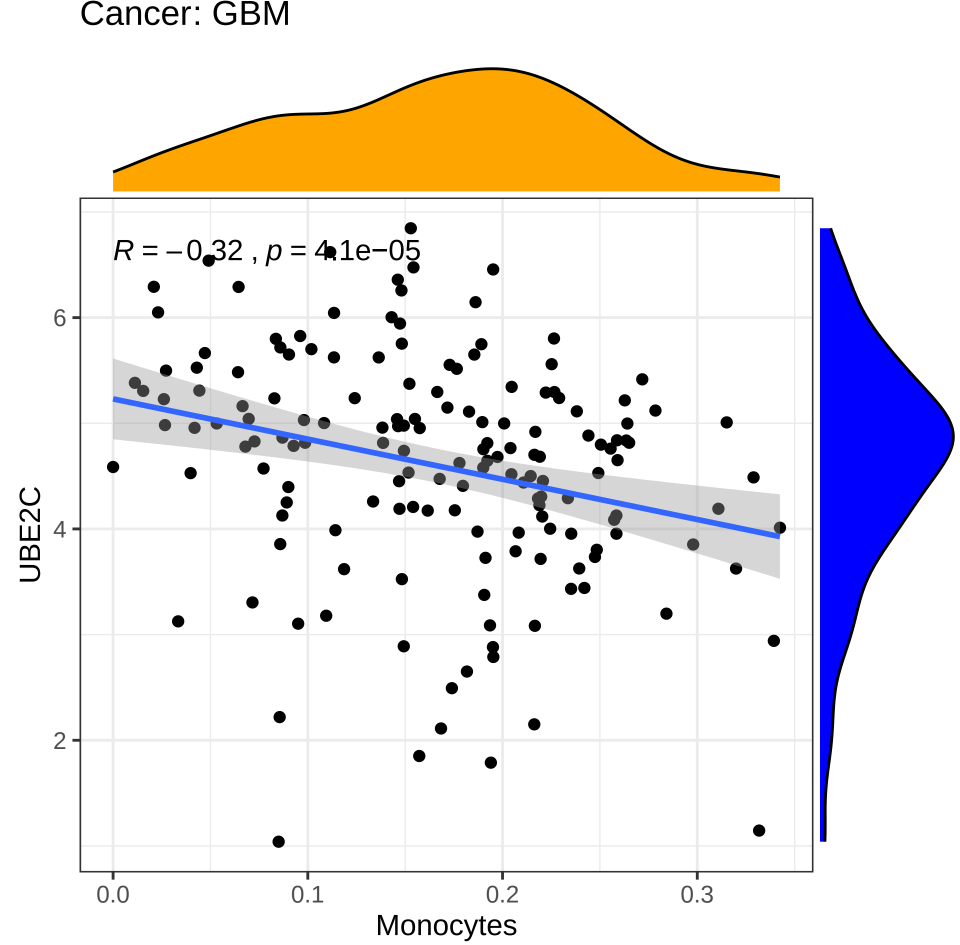

Cancer: GBM
R = −0.32 , p = 4.1e−05
UBE2C
Monocytes
0.0
0.1
0.2
0.3
2
4
6

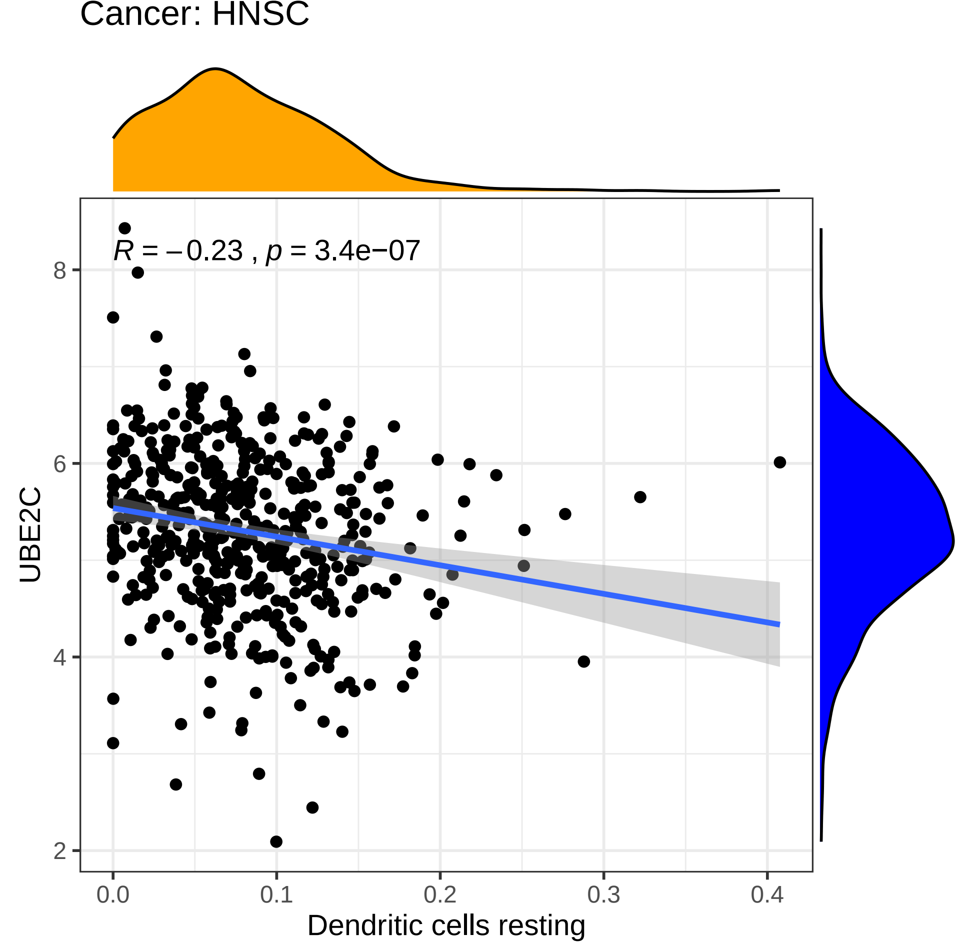
Cancer: HNSC
R = −0.23 , p = 3.4e−07
UBE2C
2
4
6
8
0.0
0.1
0.2
0.3
0.4
Dendritic cells resting

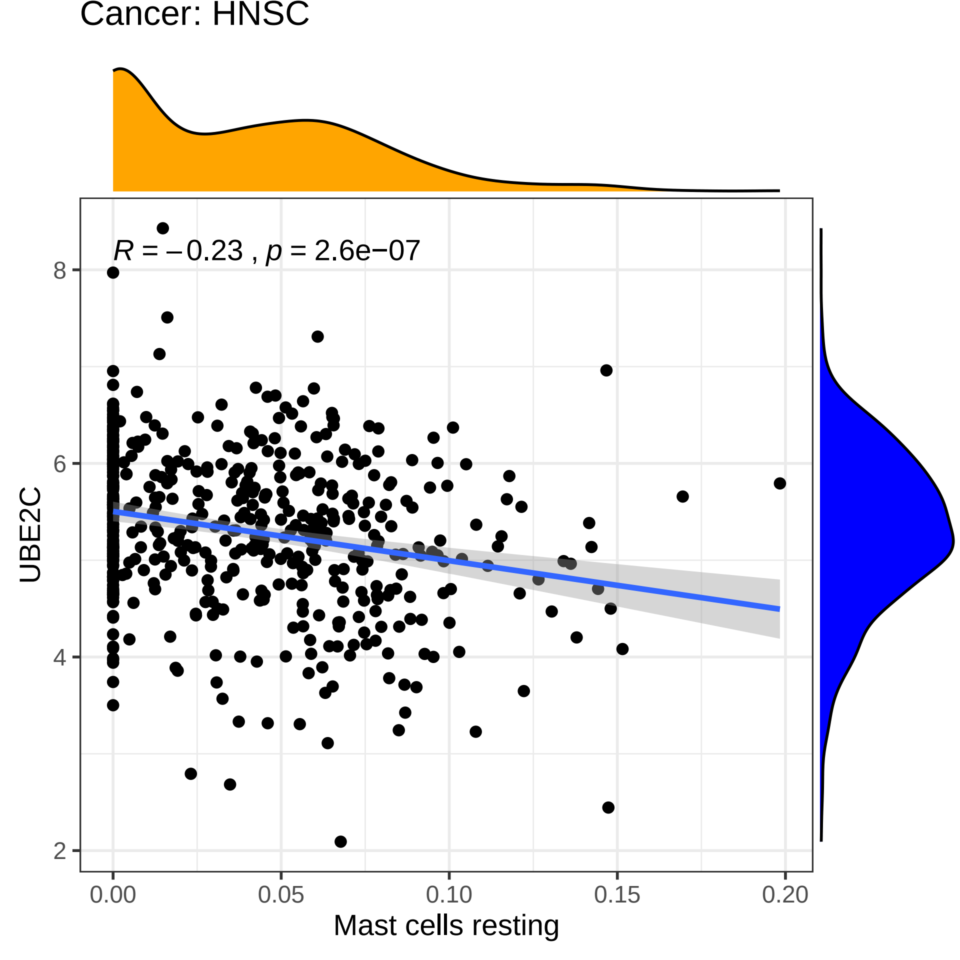

Cancer: HNSC
R = −0.23 , p = 2.6e−07
8
6
4
2
UBE2C
0.00
0.05
0.10
0.15
0.20
Mast cells resting

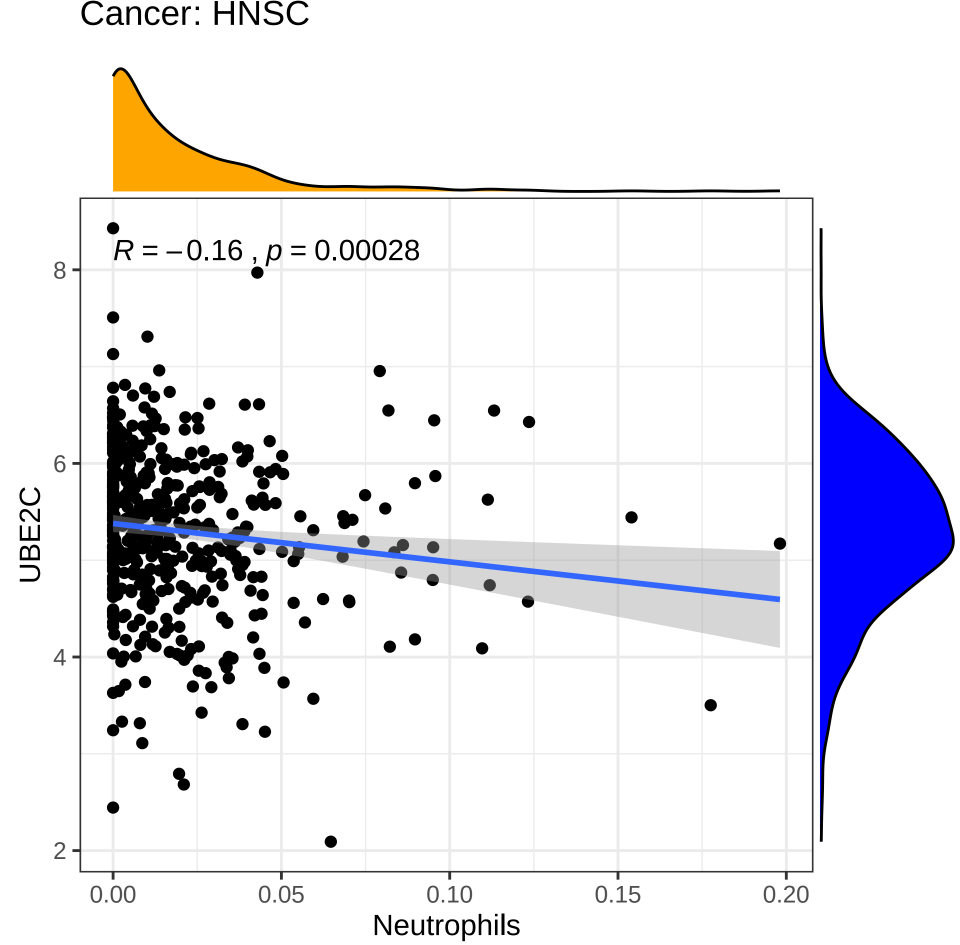
Cancer: HNSC
R = −0.16 , p = 0.00028
UBE2C
2
4
6
8
0.00
0.05
0.10
0.15
0.20
Neutrophils

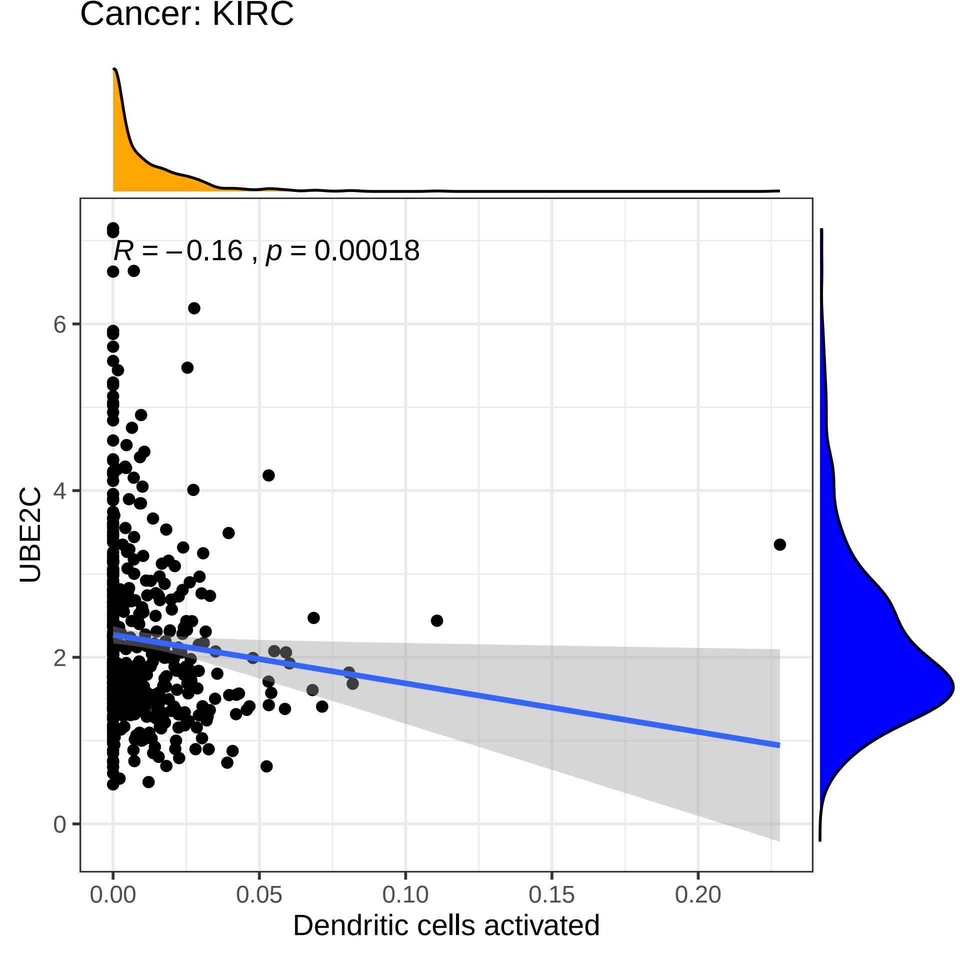

Cancer: KIRC
R = −0.16 , p = 0.00018
UBE2C
0
2
4
6
0.00
0.05
0.10
0.15
0.20
Dendritic cells activated

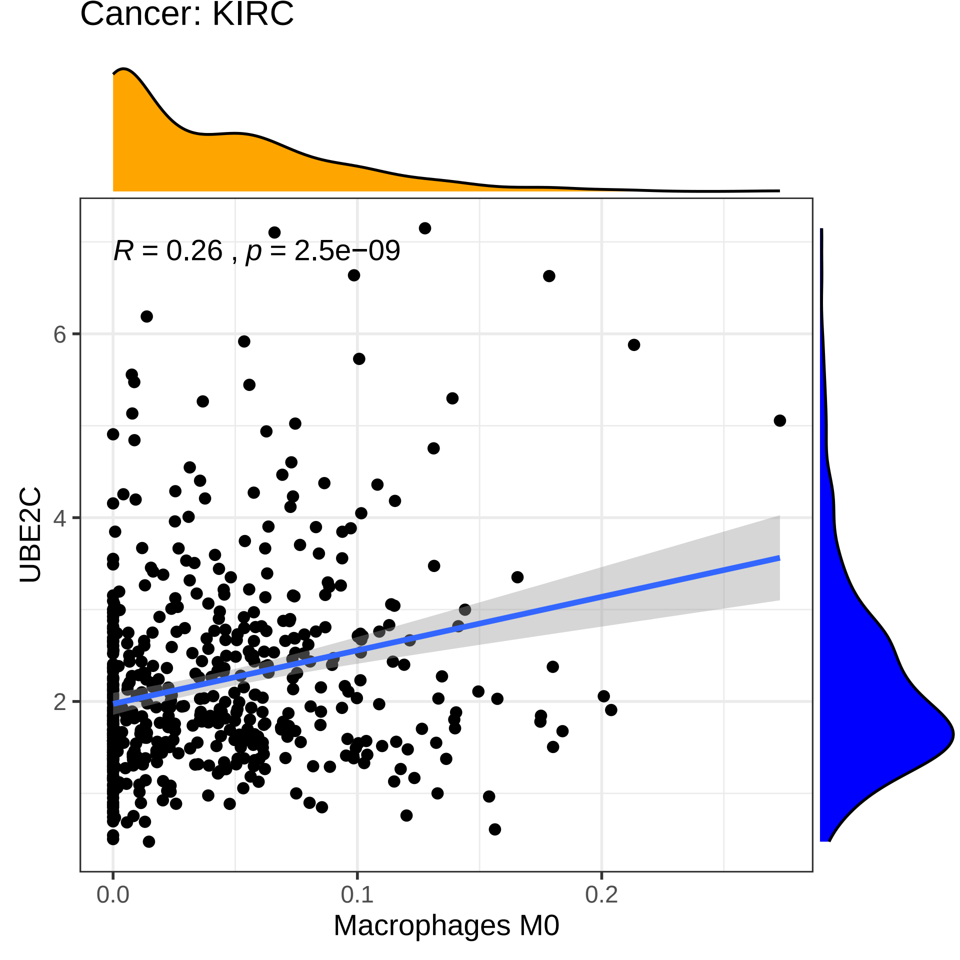

Cancer: KIRC
R = 0.26 , p = 2.5e−09
UBE2C
2
4
6
0.0
0.1
0.2
Macrophages M0

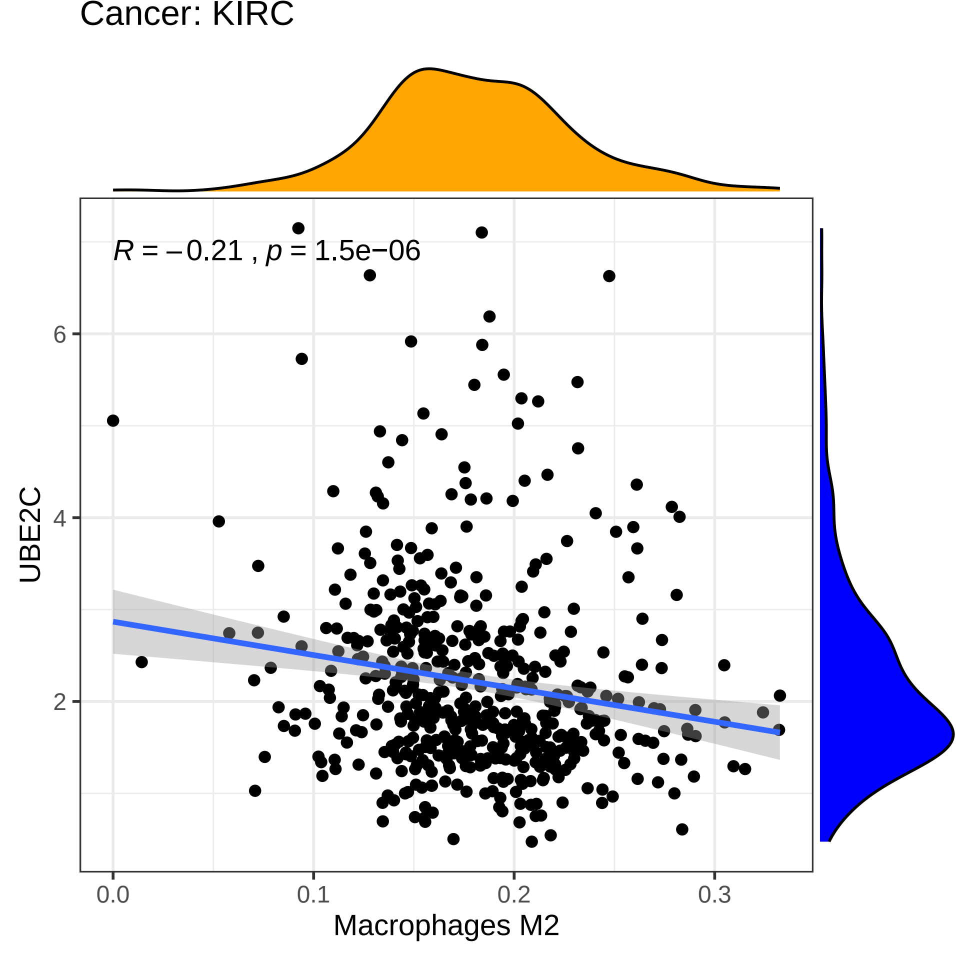

Cancer: KIRC
R = −0.21 , p = 1.5e−06
UBE2C
6
4
2
0.0
0.1
0.2
0.3
Macrophages M2

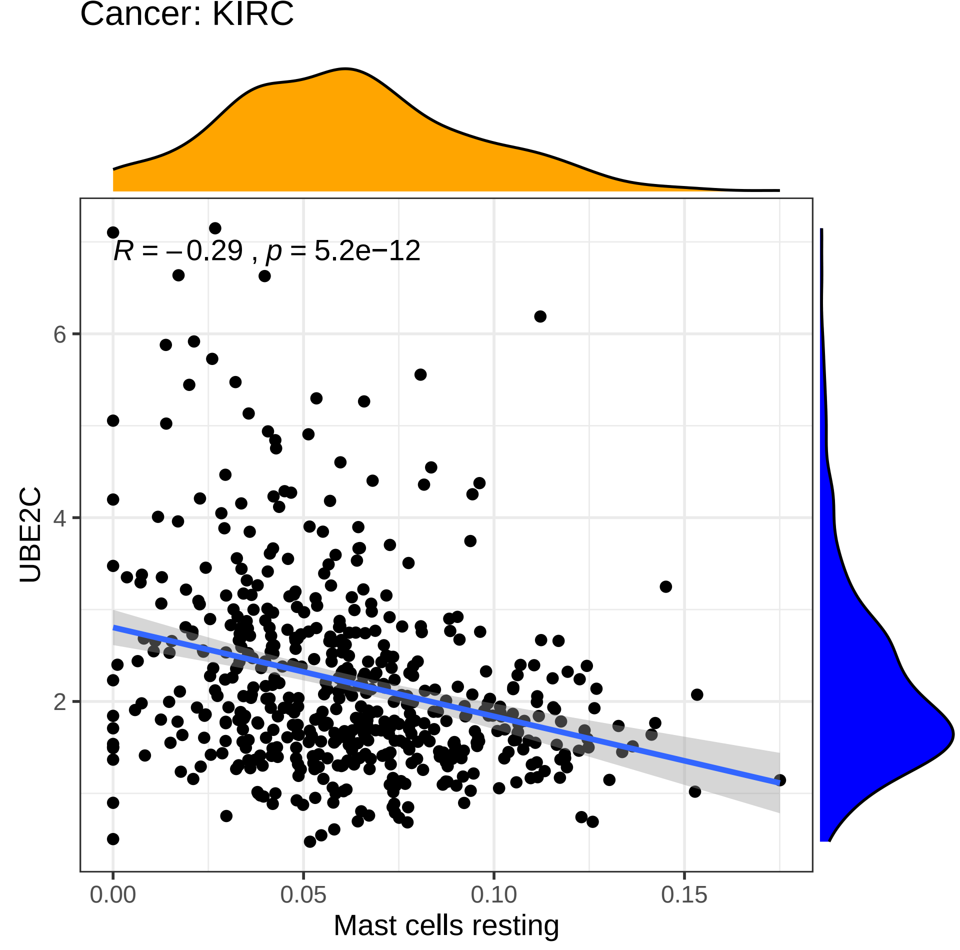
Cancer: KIRC
R = −0.29 , p = 5.2e−12
UBE2C
6
4
2
0.00
0.05
0.10
0.15
Mast cells resting

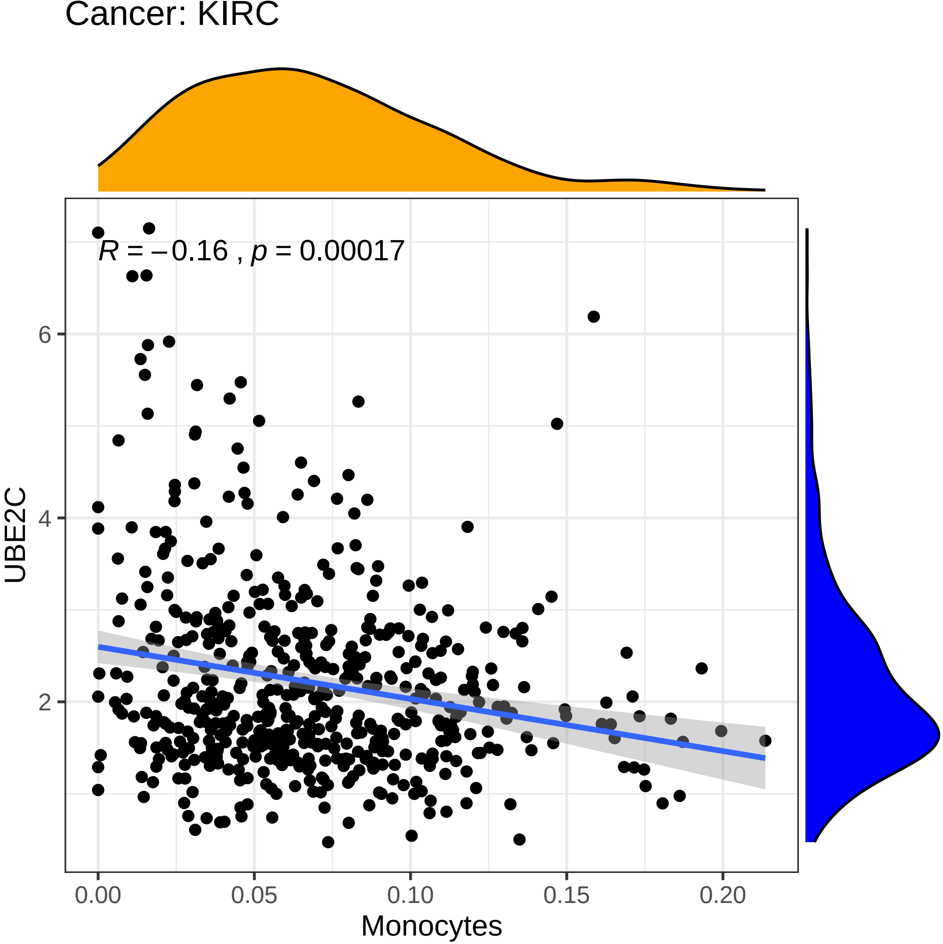

Cancer: KIRC
R = −0.16 , p = 0.00017
UBE2C
6
4
2
0.00
0.05
0.10
0.15
0.20
Monocytes

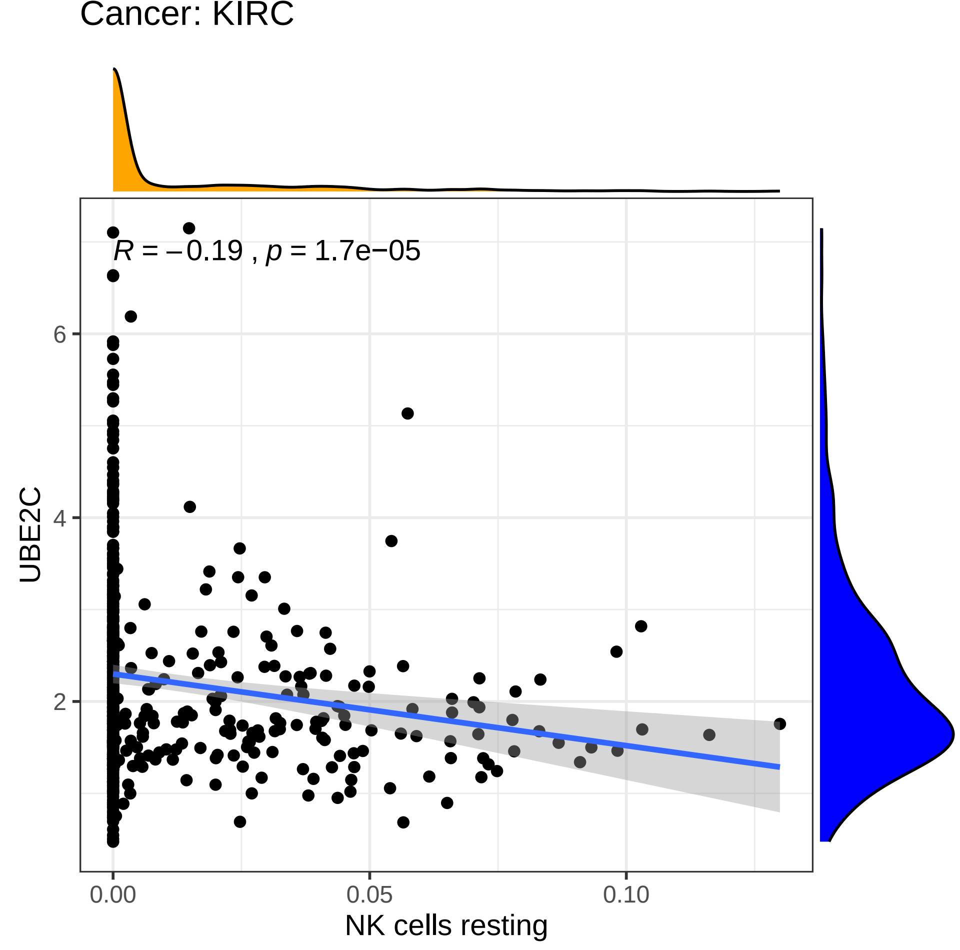
Cancer: KIRC
R = −0.19 , p = 1.7e−05
UBE2C
6
4
2
0.00
0.05
0.10
NK cells resting

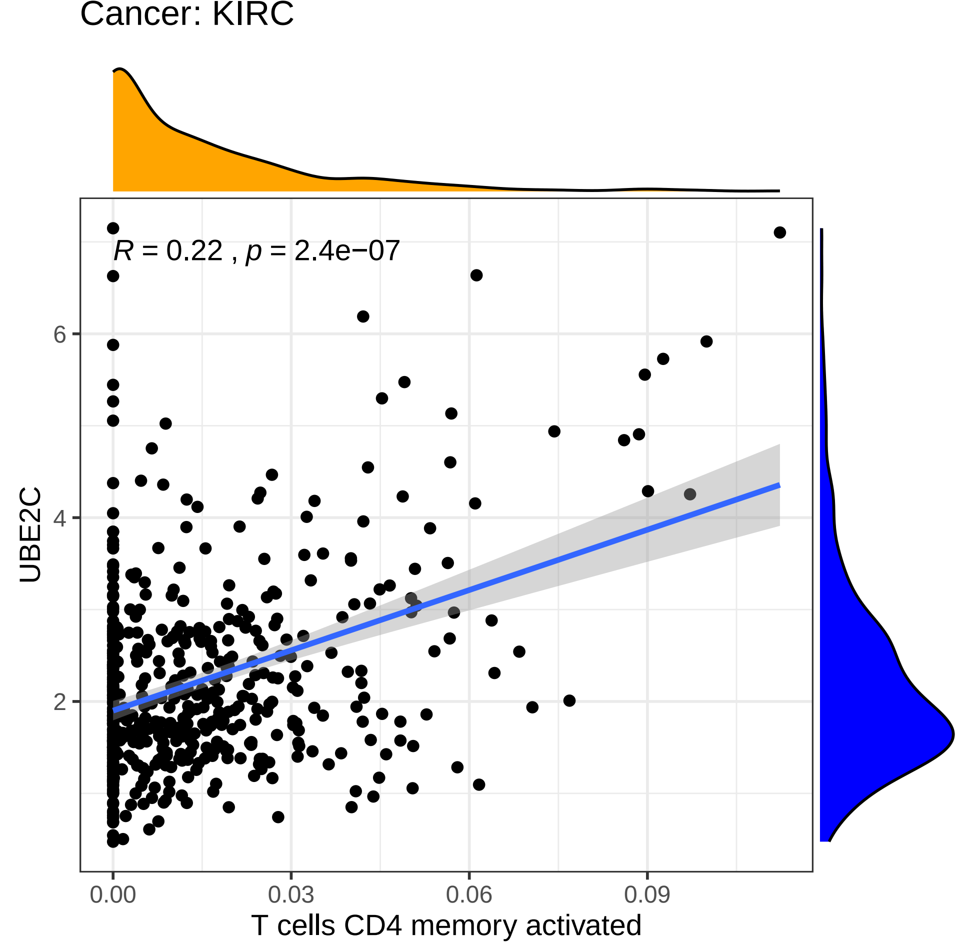

Cancer: KIRC
R = 0.22 , p = 2.4e−07
UBE2C
2
4
6
0.00
0.03
0.06
0.09
T cells CD4 memory activated

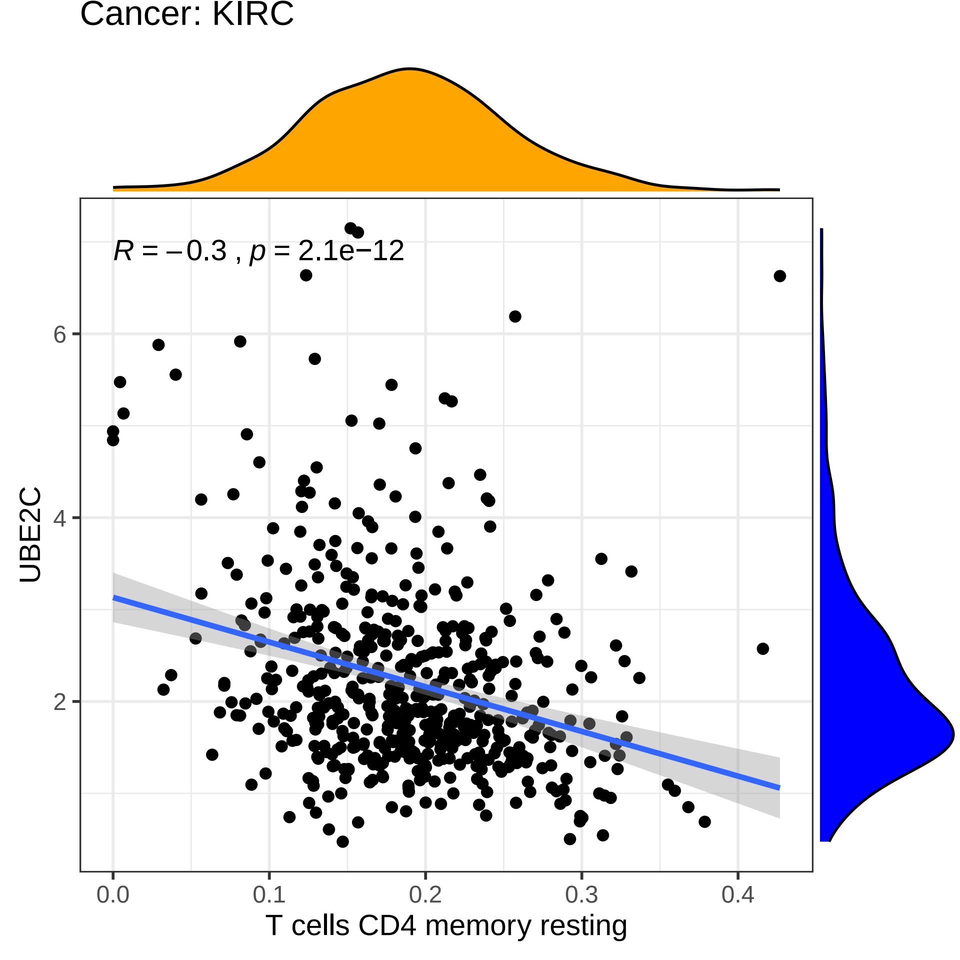
Cancer: KIRC
R = −0.3 , p = 2.1e−12
UBE2C
6
4
2
0.0
0.1
0.2
0.3
0.4
T cells CD4 memory resting

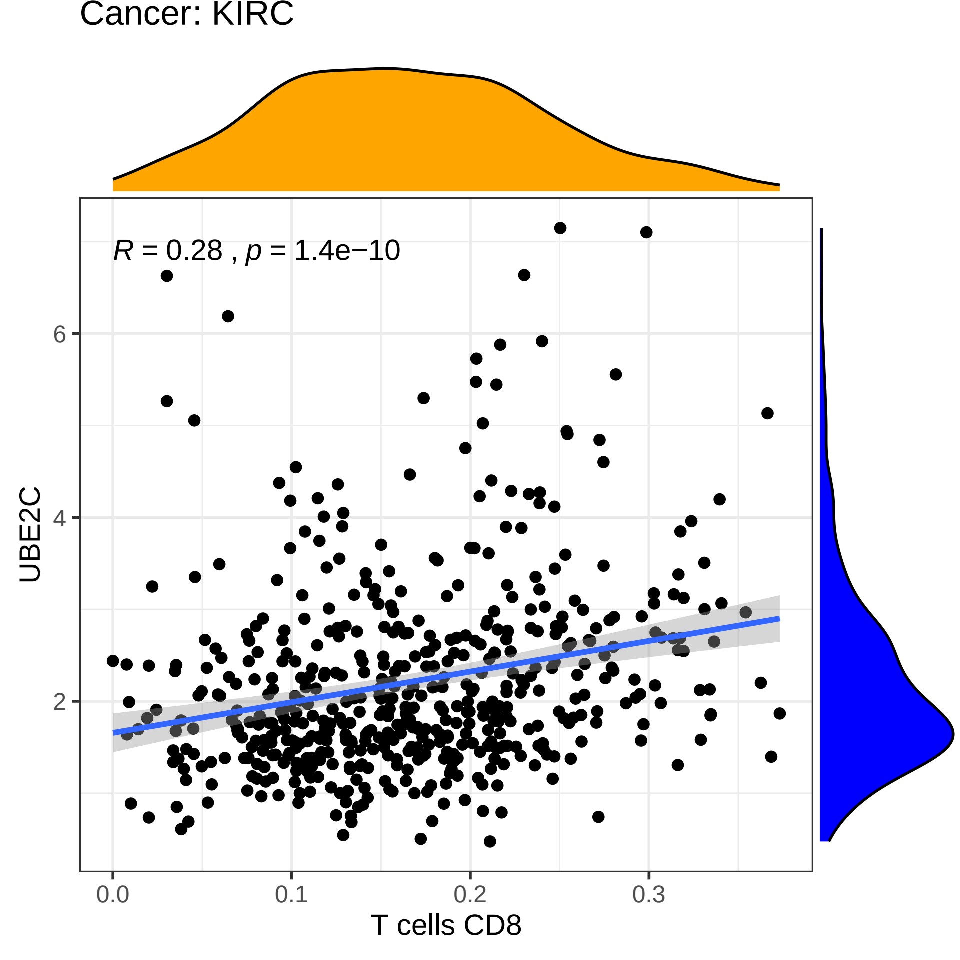
Cancer: KIRC
R = 0.28 , p = 1.4e−10
UBE2C
T cells CD8
0.0
0.1
0.2
0.3
2
4
6

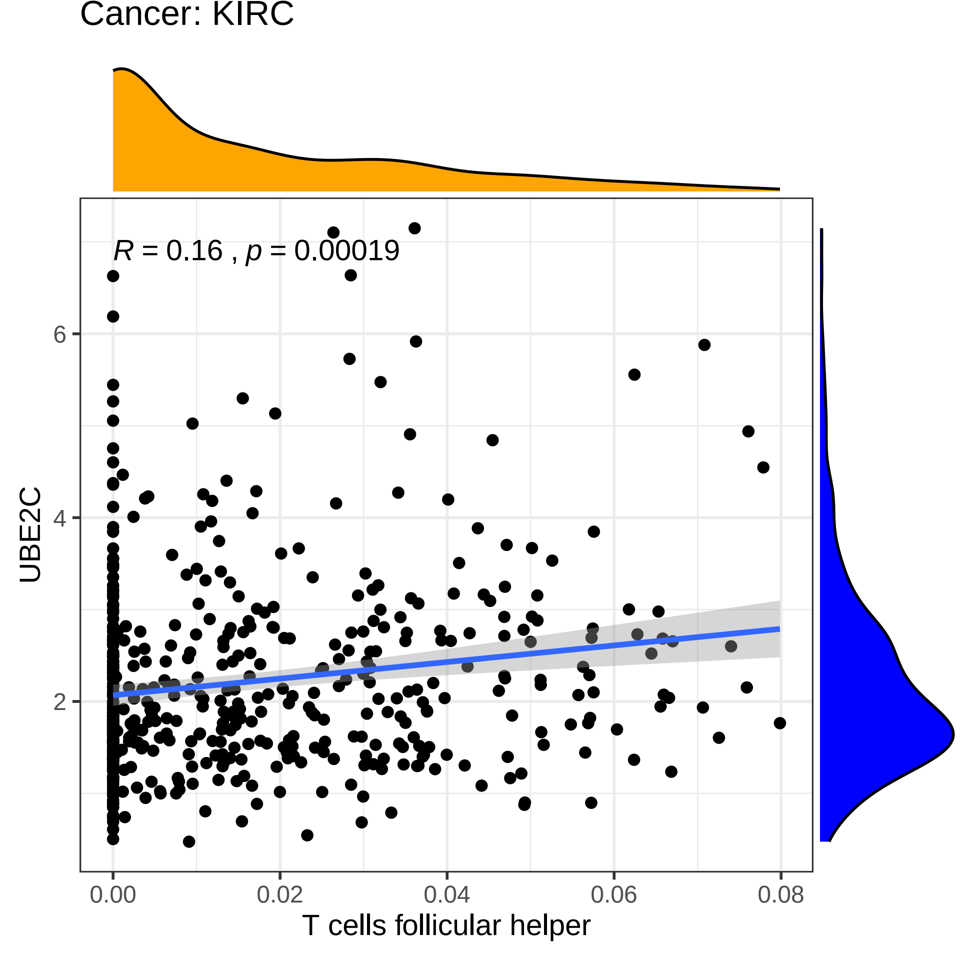

Cancer: KIRC
R = 0.16 , p = 0.00019
UBE2C
2
4
6
0.00
0.02
0.04
0.06
0.08
T cells follicular helper

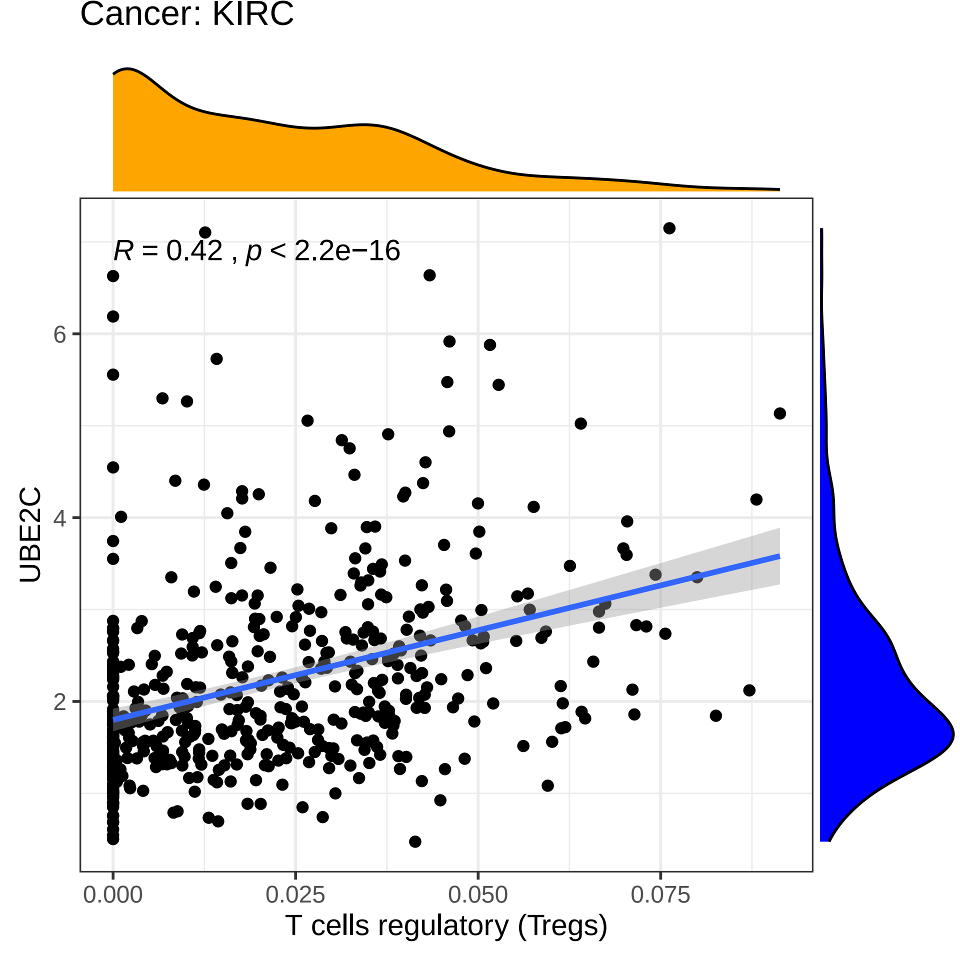

Cancer: KIRC
R = 0.42 , p < 2.2e−16
UBE2C
6
4
2
0.000
0.025
0.050
0.075
T cells regulatory (Tregs)

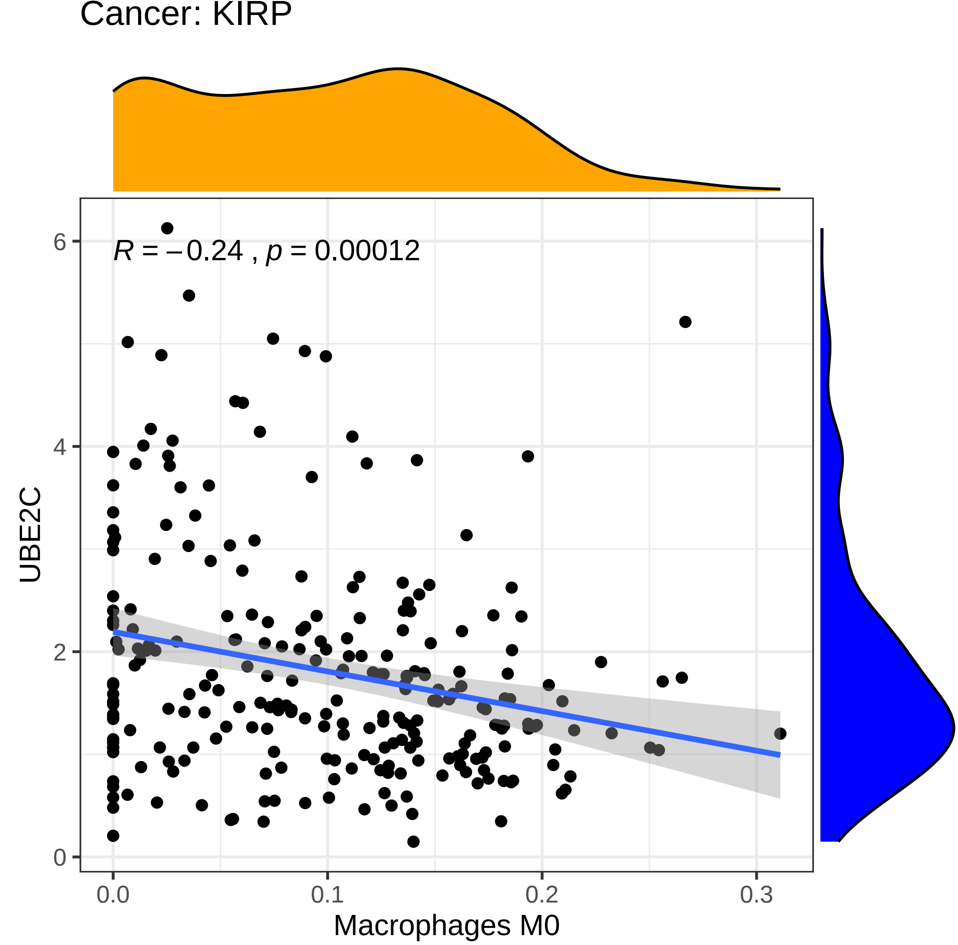

Cancer: KIRP
R = −0.24 , p = 0.00012
UBE2C
Macrophages M0
0
2
4
6
0.0
0.1
0.2
0.3

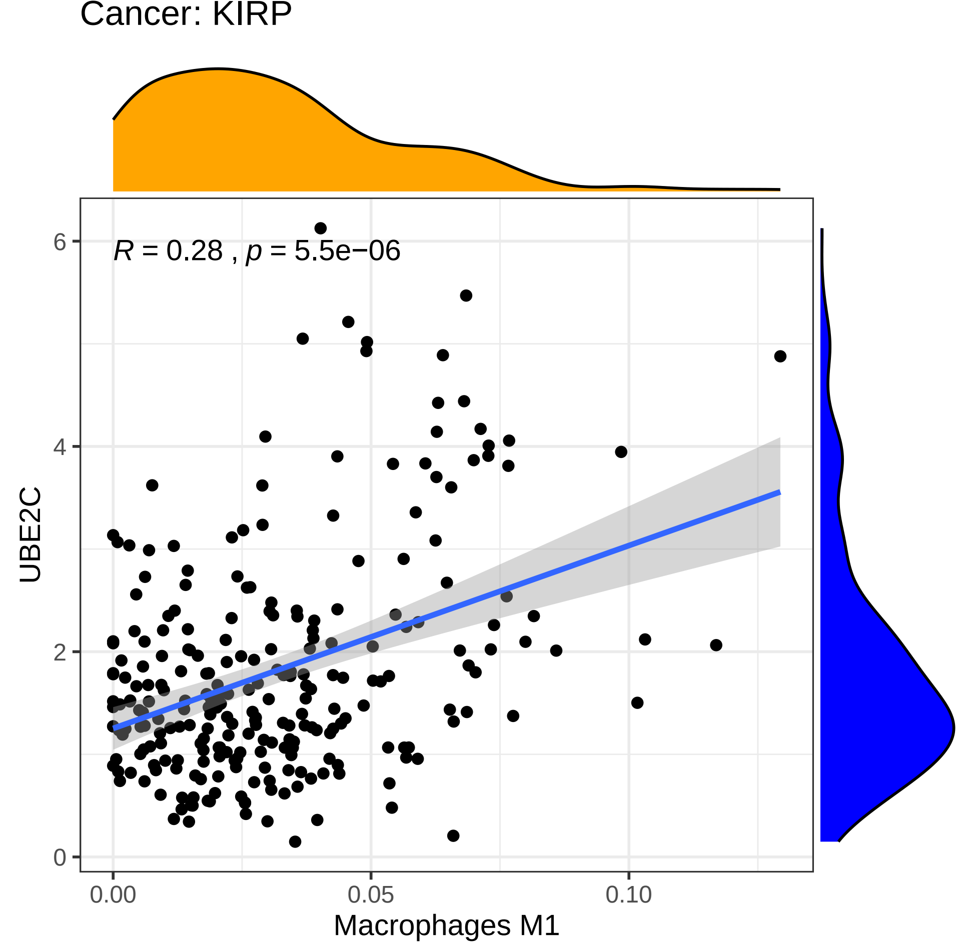
Cancer: KIRP
R = 0.28 , p = 5.5e−06
UBE2C
Macrophages M1
0
2
4
6
0.00
0.05
0.10

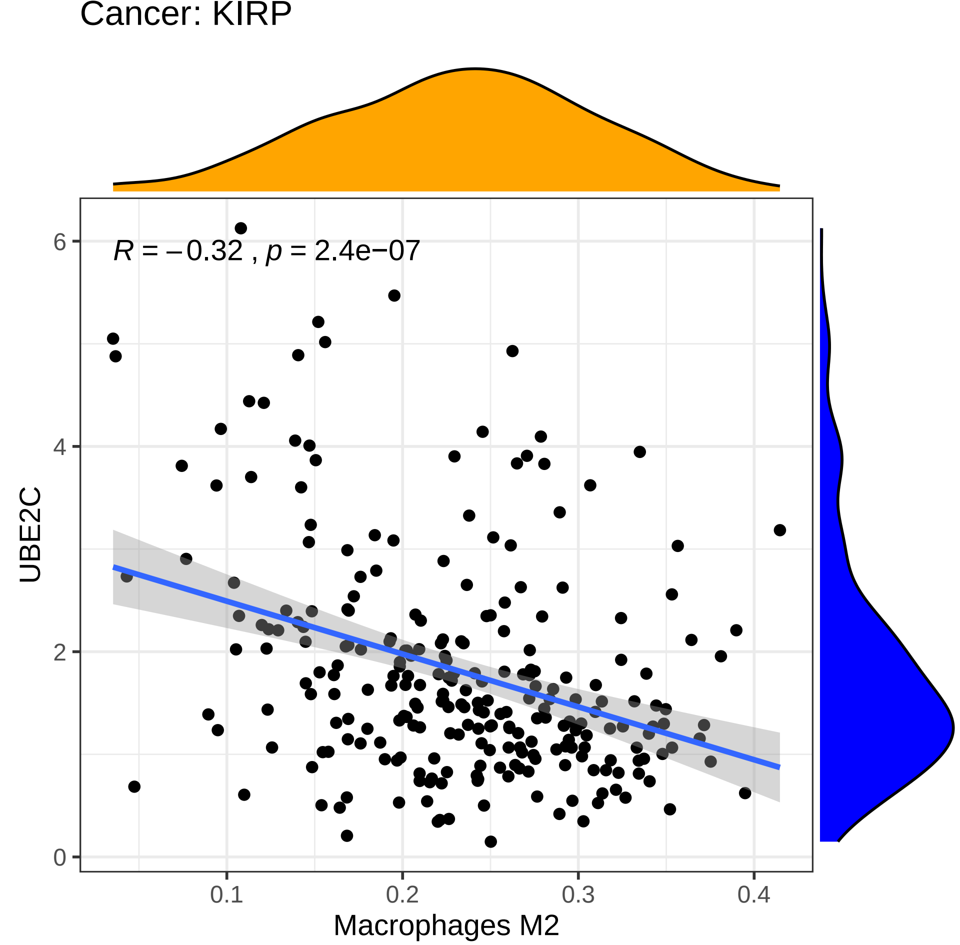

Cancer: KIRP
R = −0.32 , p = 2.4e−07
UBE2C
Macrophages M2
0
2
4
6
0.1
0.2
0.3
0.4

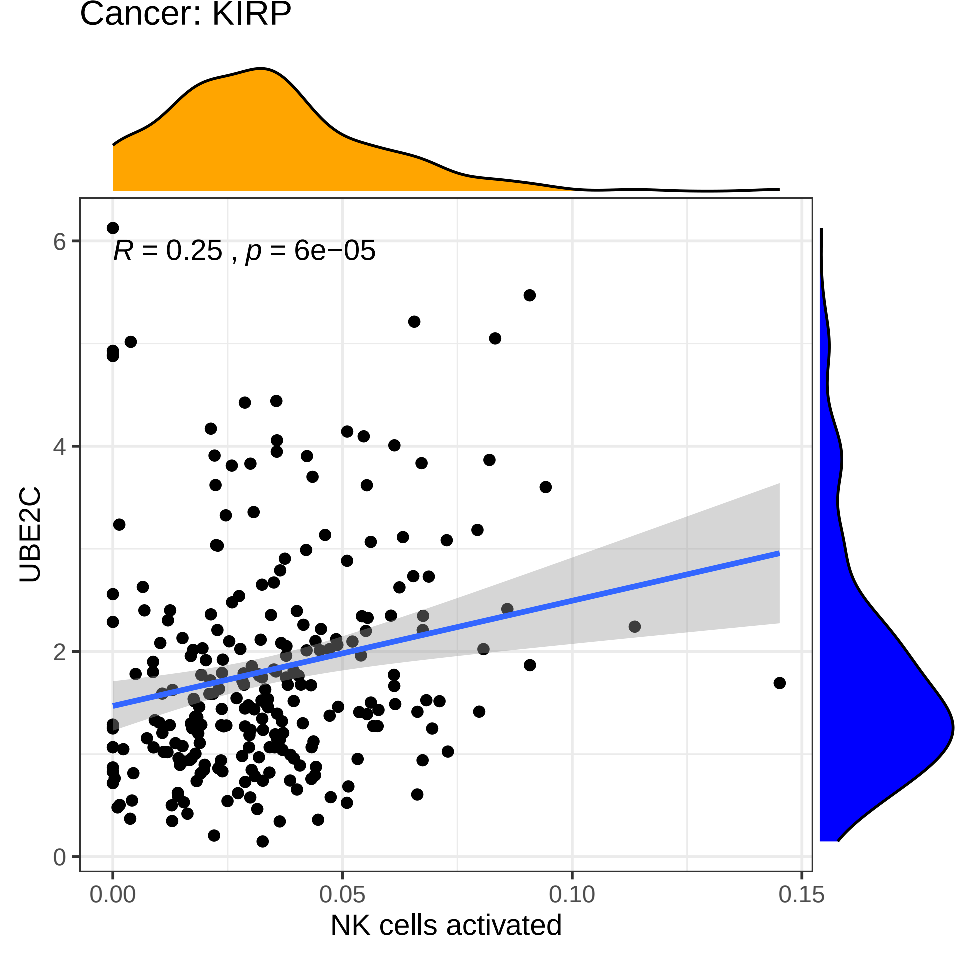
Cancer: KIRP
R = 0.25 , p = 6e−05
UBE2C
NK cells activated
0
2
4
6
0.00
0.05
0.10
0.15

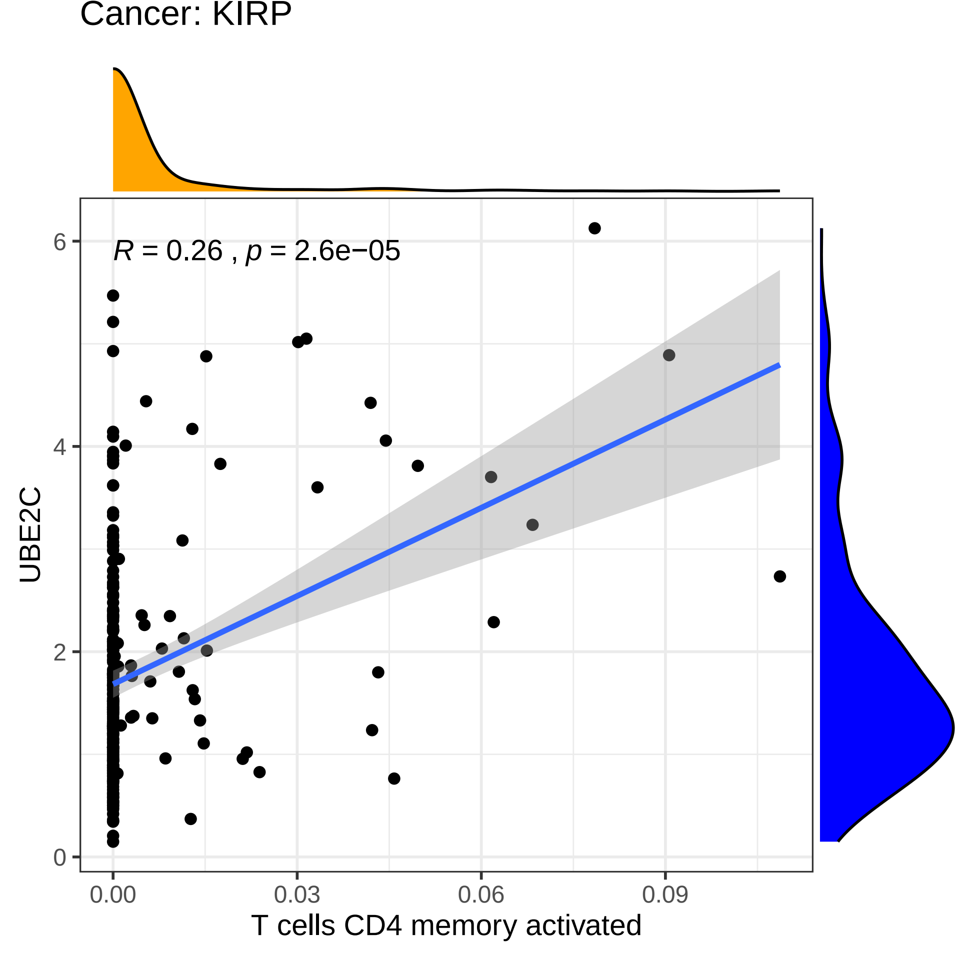

Cancer: KIRP
R = 0.26 , p = 2.6e−05
UBE2C
0
2
4
6
0.00
0.03
0.06
0.09
T cells CD4 memory activated

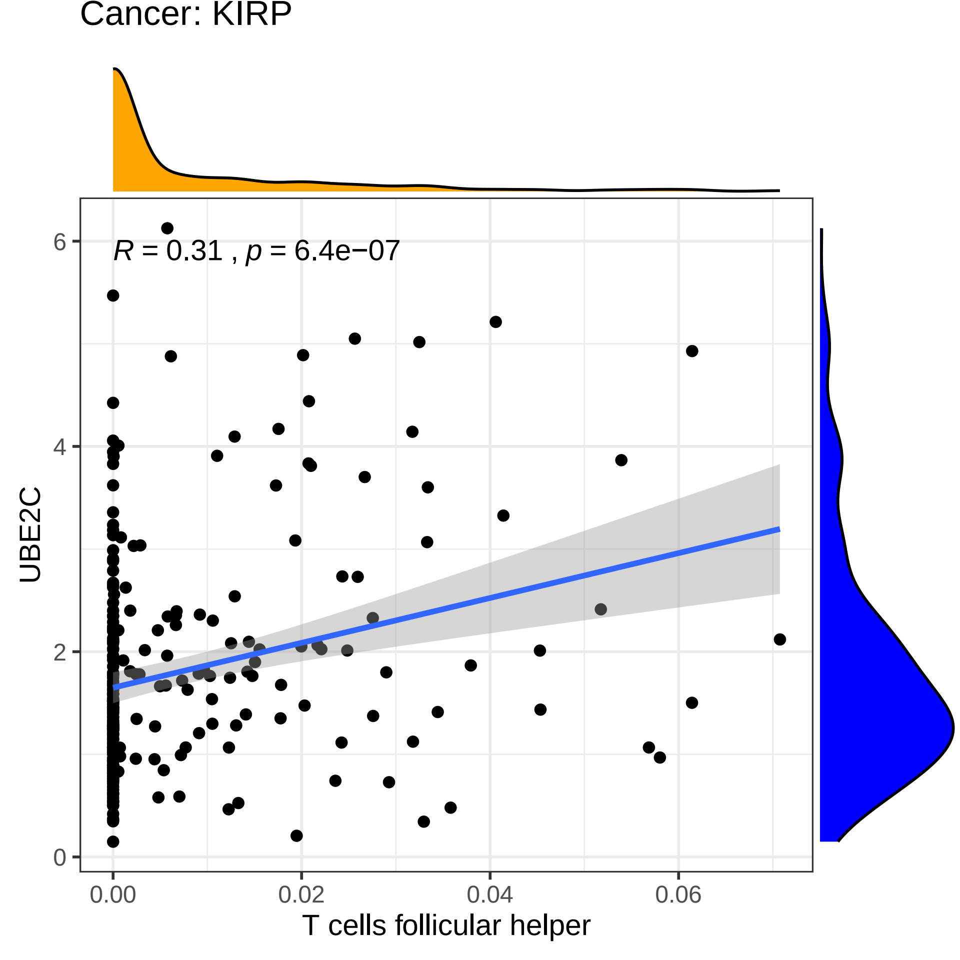

Cancer: KIRP
R = 0.31 , p = 6.4e−07
UBE2C
T cells follicular helper
0
2
4
6
0.00
0.02
0.04
0.06

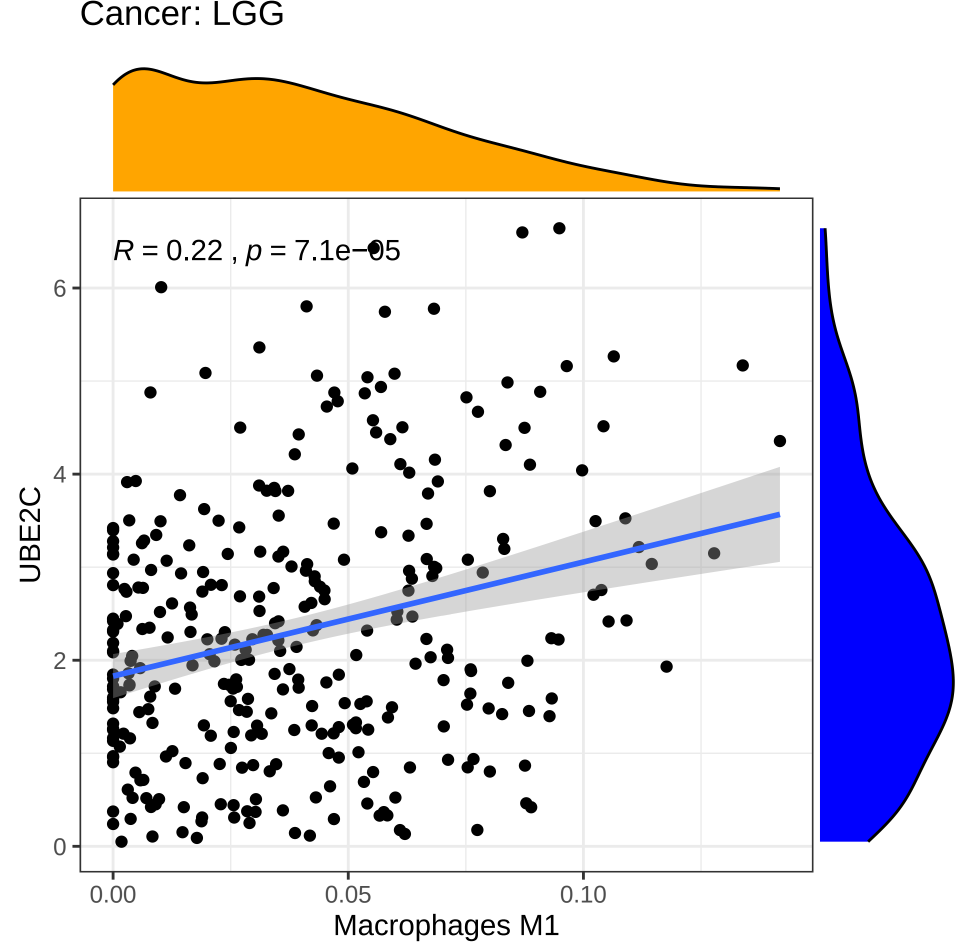

Cancer: LGG
R = 0.22 , p = 7.1e−05
UBE2C
0
2
4
6
0.00
0.05
0.10
Macrophages M1

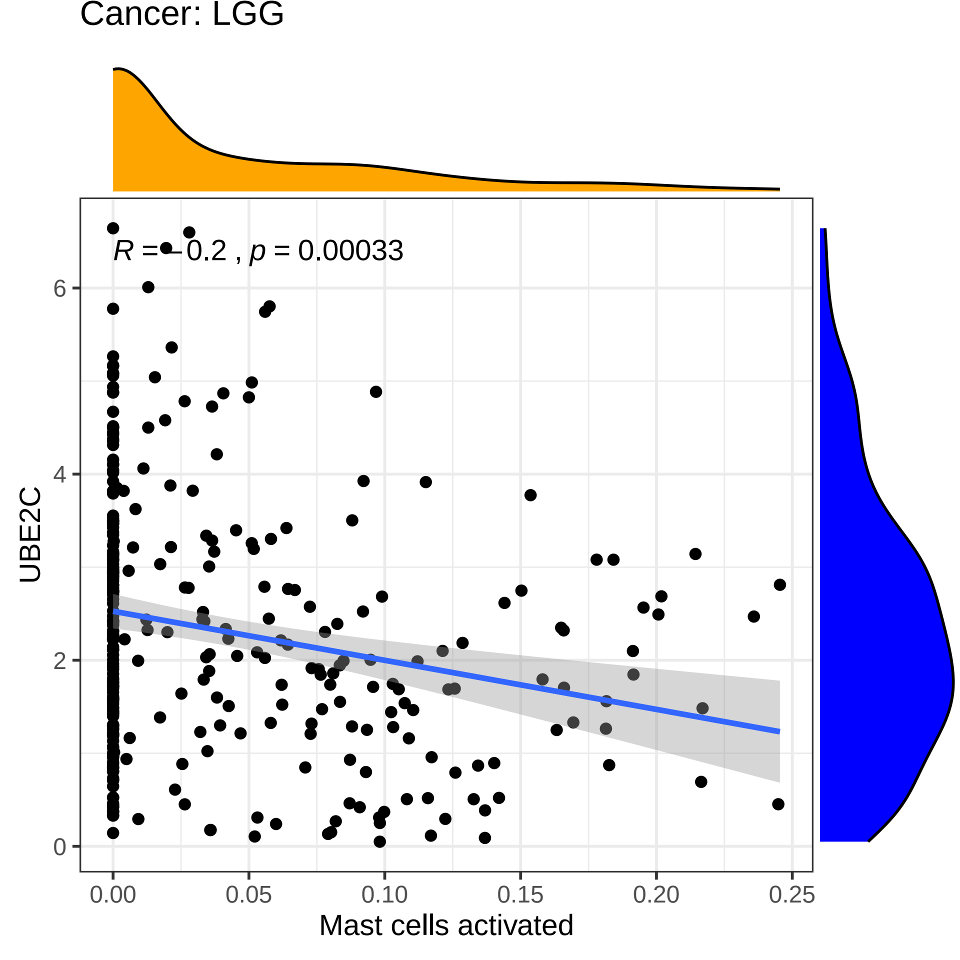

Cancer: LGG
R = −0.2 , p = 0.00033
UBE2C
0
2
4
6
0.00
0.05
0.10
0.15
0.20
0.25
Mast cells activated

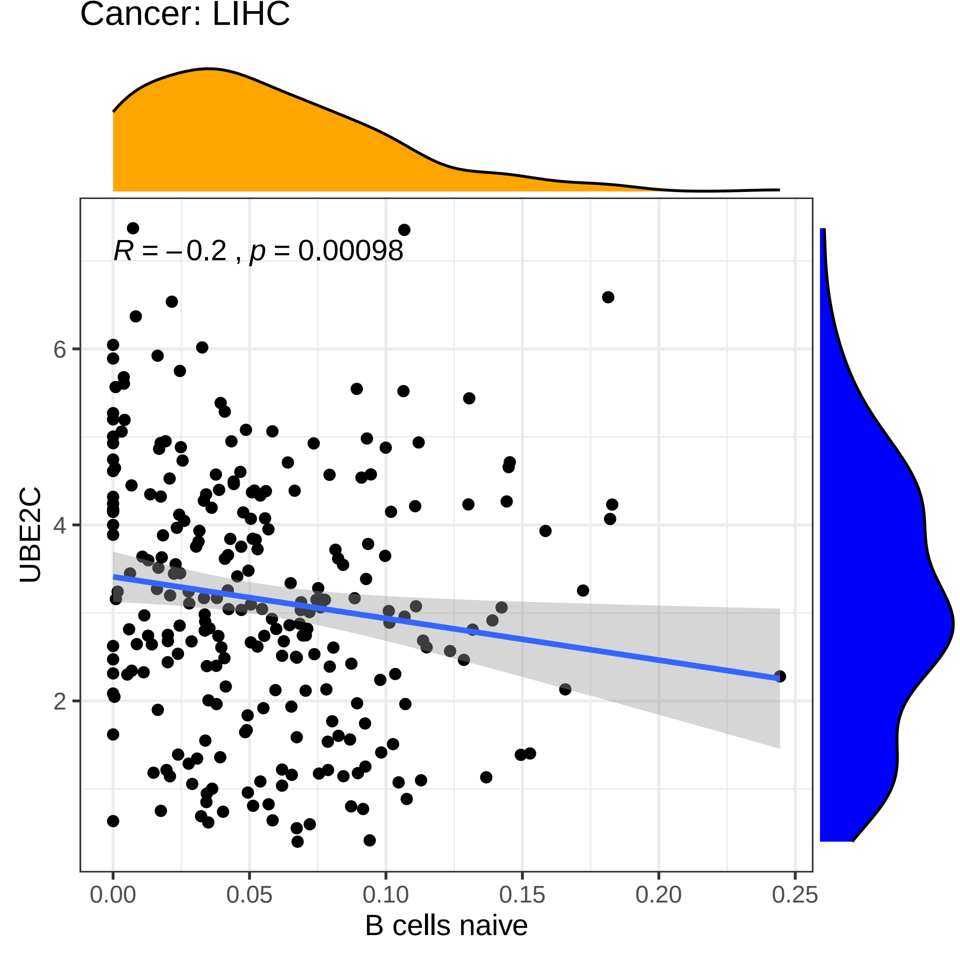

Cancer: LIHC
R = −0.2 , p = 0.00098
UBE2C
2
4
6
0.00
0.05
0.10
0.15
0.20
0.25
B cells naive

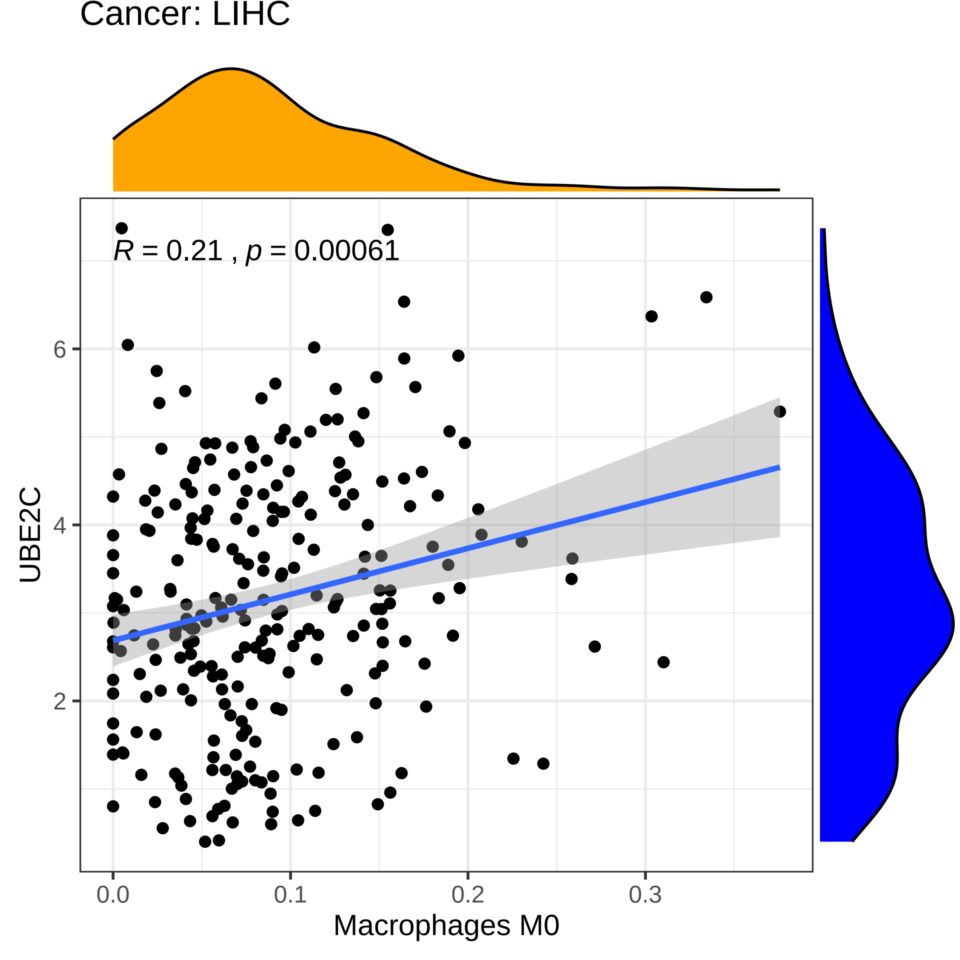
Cancer: LIHC
R = 0.21 , p = 0.00061
UBE2C
2
4
6
0.0
0.1
0.2
0.3
Macrophages M0

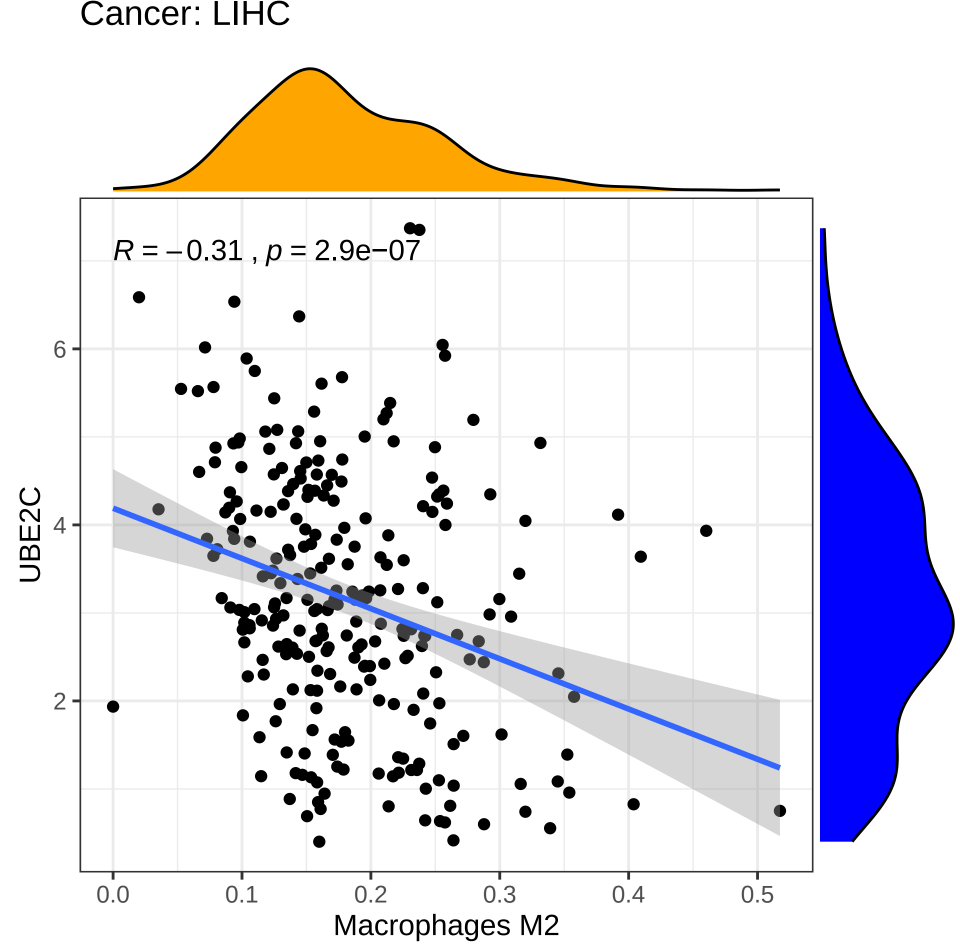
Cancer: LIHC
R = −0.31 , p = 2.9e−07
UBE2C
6
4
2
0.0
0.1
0.2
0.3
0.4
0.5
Macrophages M2

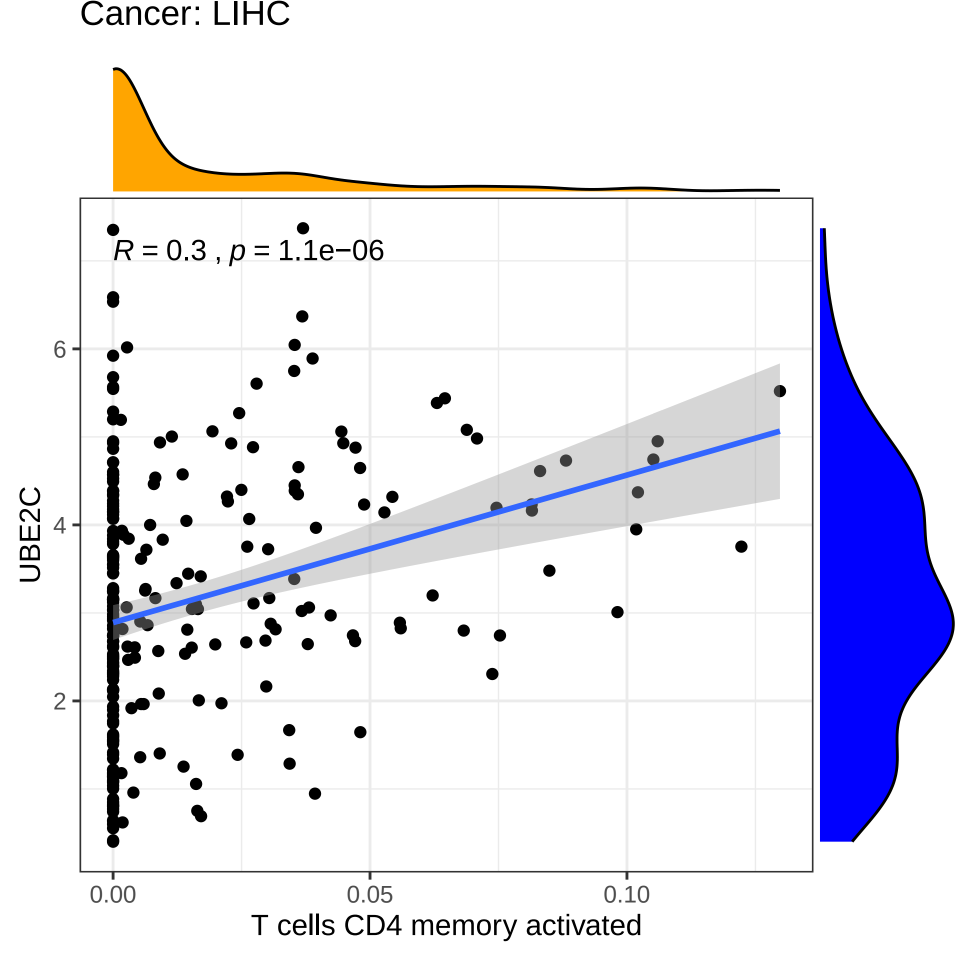

Cancer: LIHC
R = 0.3 , p = 1.1e−06
6
4
2
UBE2C
0.00
0.05
0.10
T cells CD4 memory activated

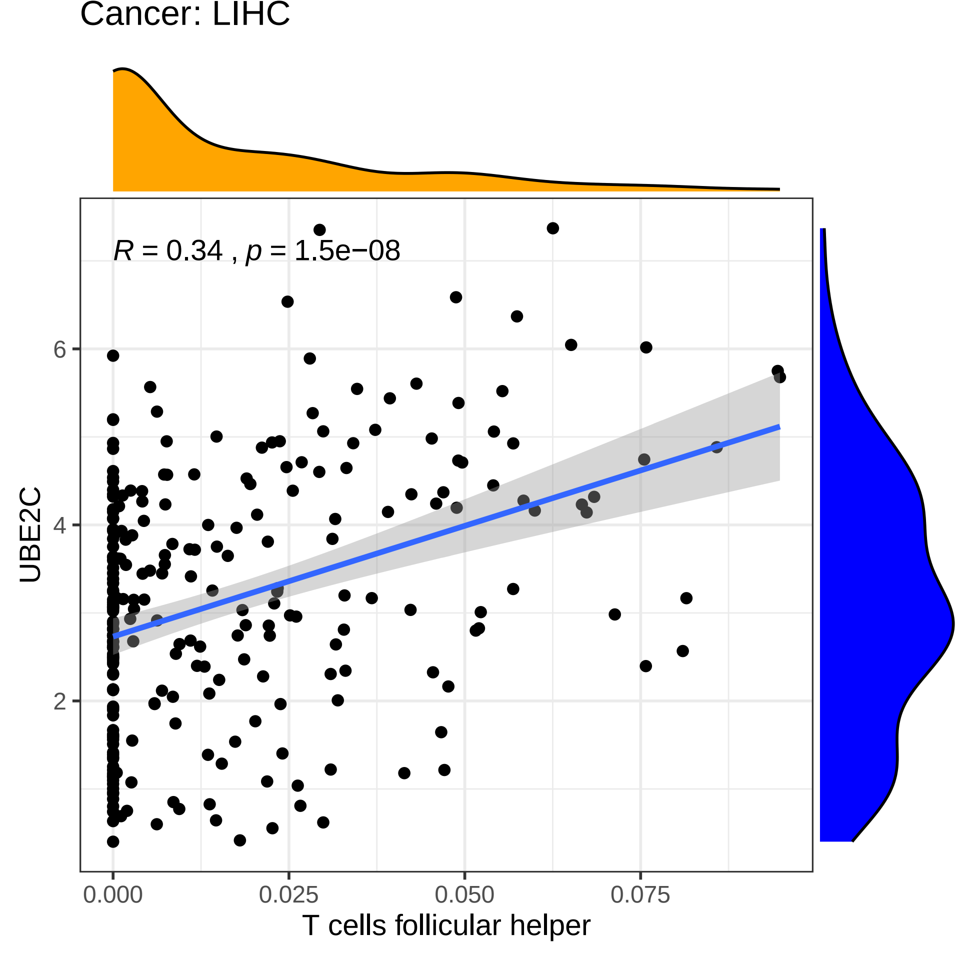
Cancer: LIHC
R = 0.34 , p = 1.5e−08
UBE2C
2
4
6
0.000
0.025
0.050
0.075
T cells follicular helper

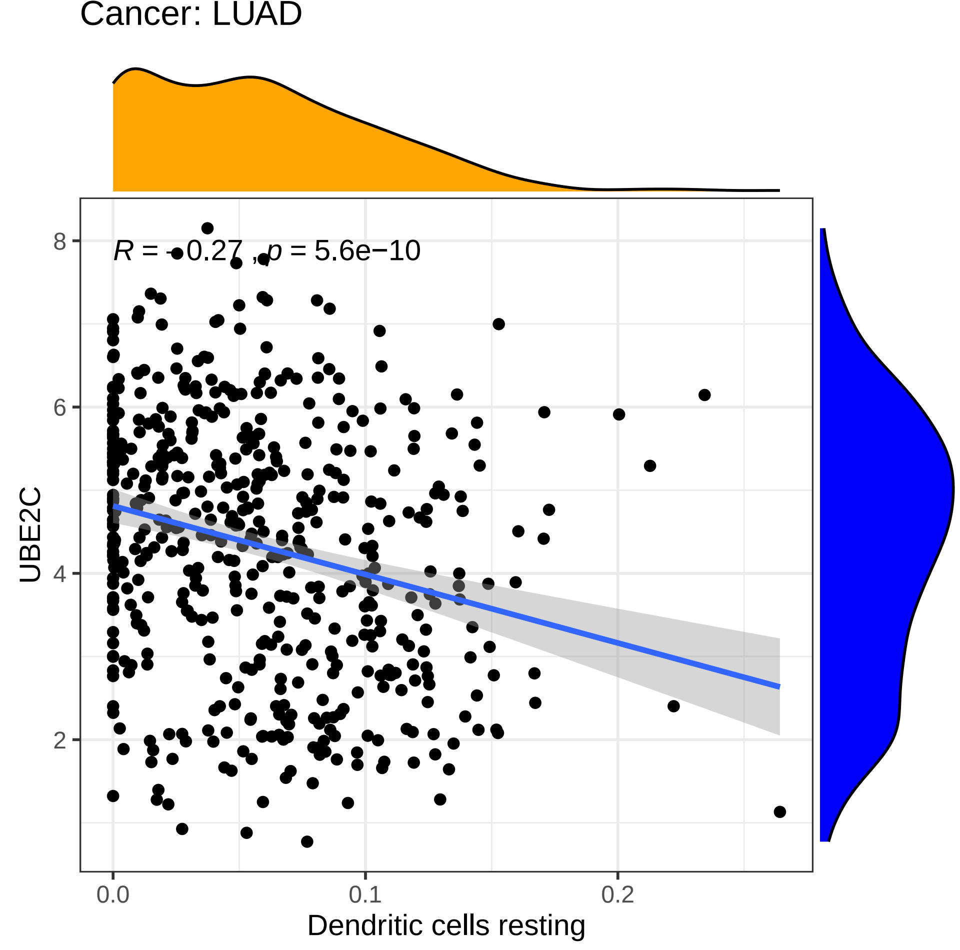

Cancer: LUAD
R = -0.27 , p = 5.6e−10
8
6
4
2
UBE2C
0.0
0.1
0.2
Dendritic cells resting

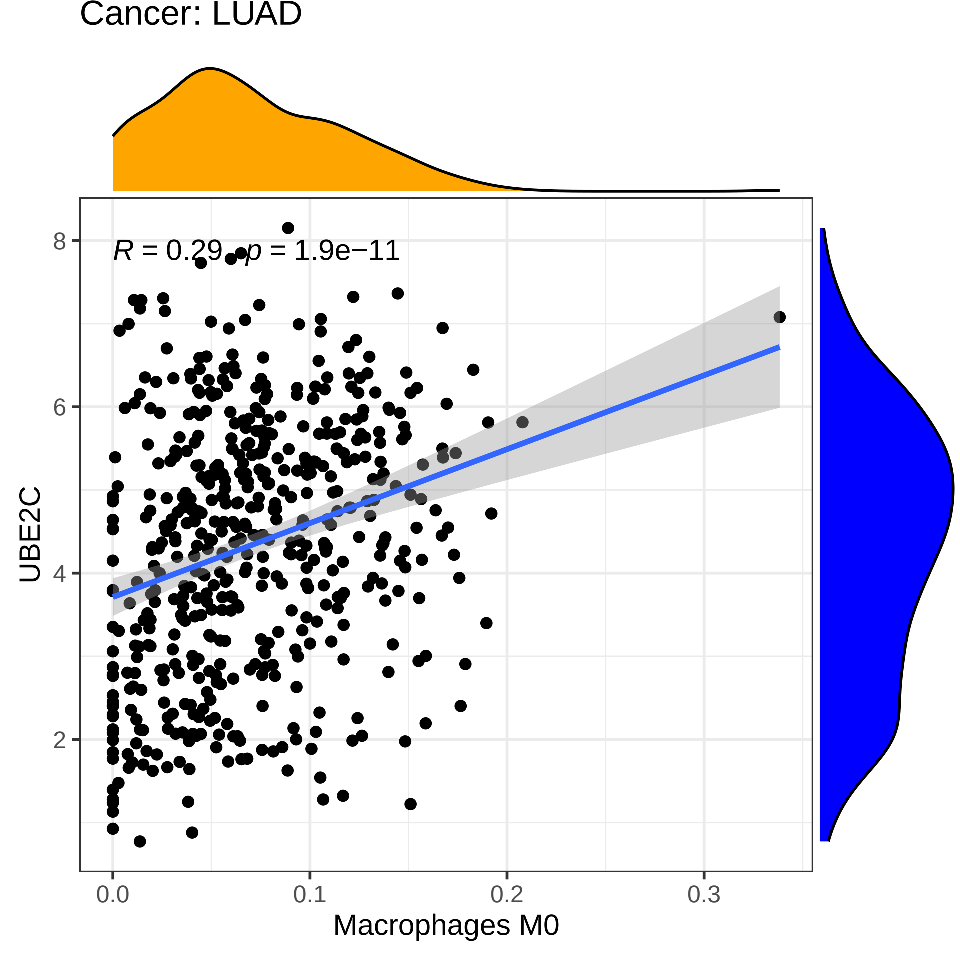
Cancer: LUAD
R = 0.29, p = 1.9e−11
UBE2C
8
6
4
2
0.0
0.1
0.2
0.3
Macrophages M0

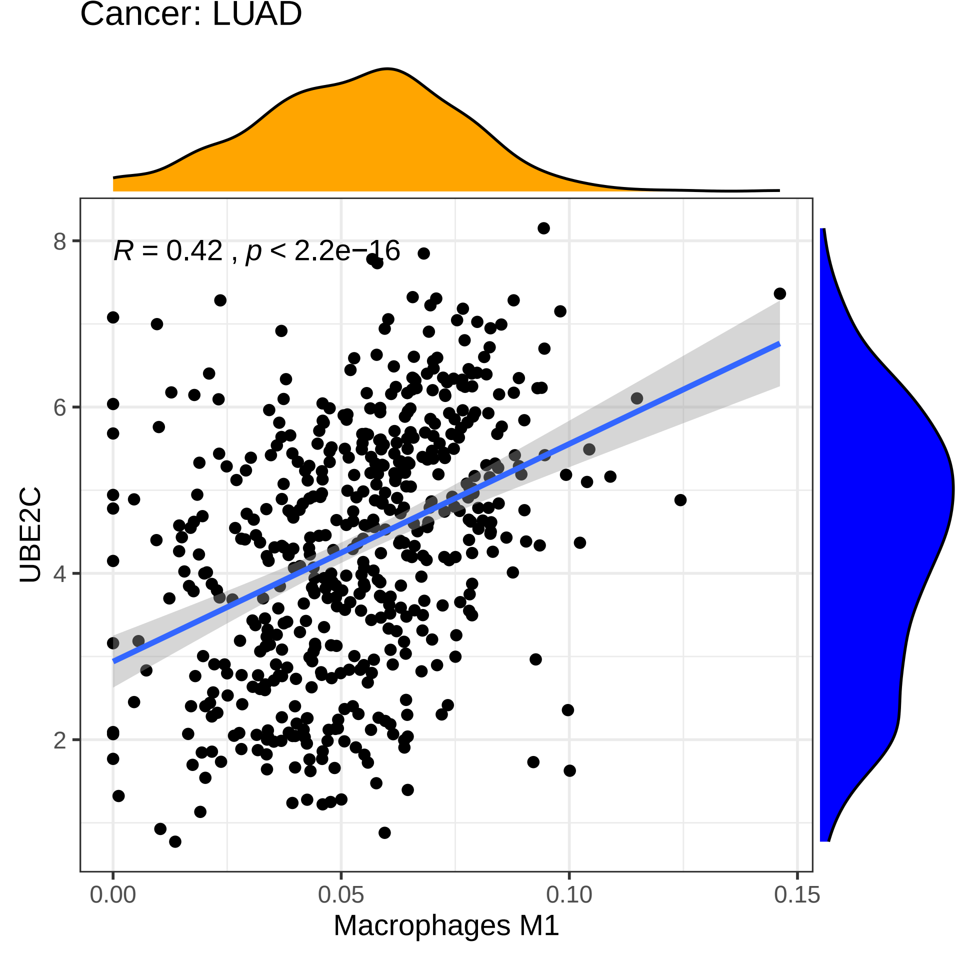
Cancer: LUAD
R = 0.42 , p < 2.2e−16
UBE2C
8
6
4
2
0.00
0.05
0.10
0.15
Macrophages M1

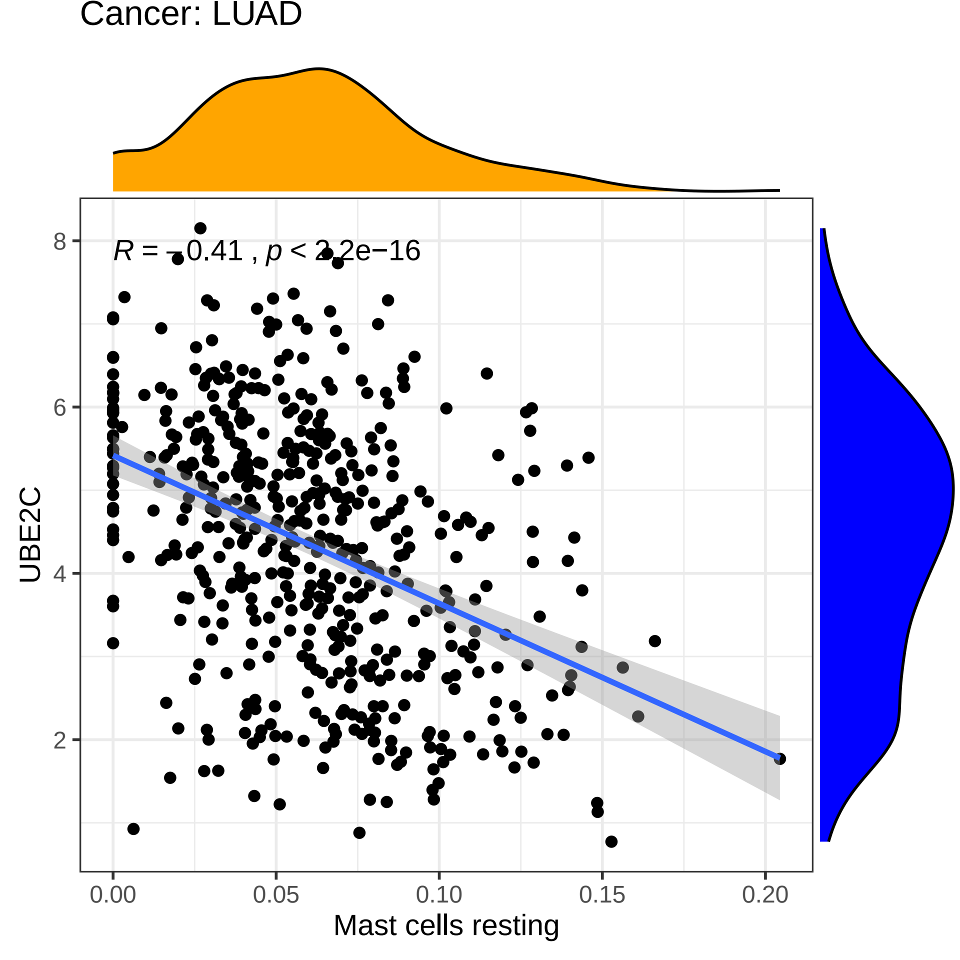

Cancer: LUAD
R = −0.41 , p < 2.2e−16
UBE2C
Mast cells resting
8
6
4
2
0.00
0.05
0.10
0.15
0.20

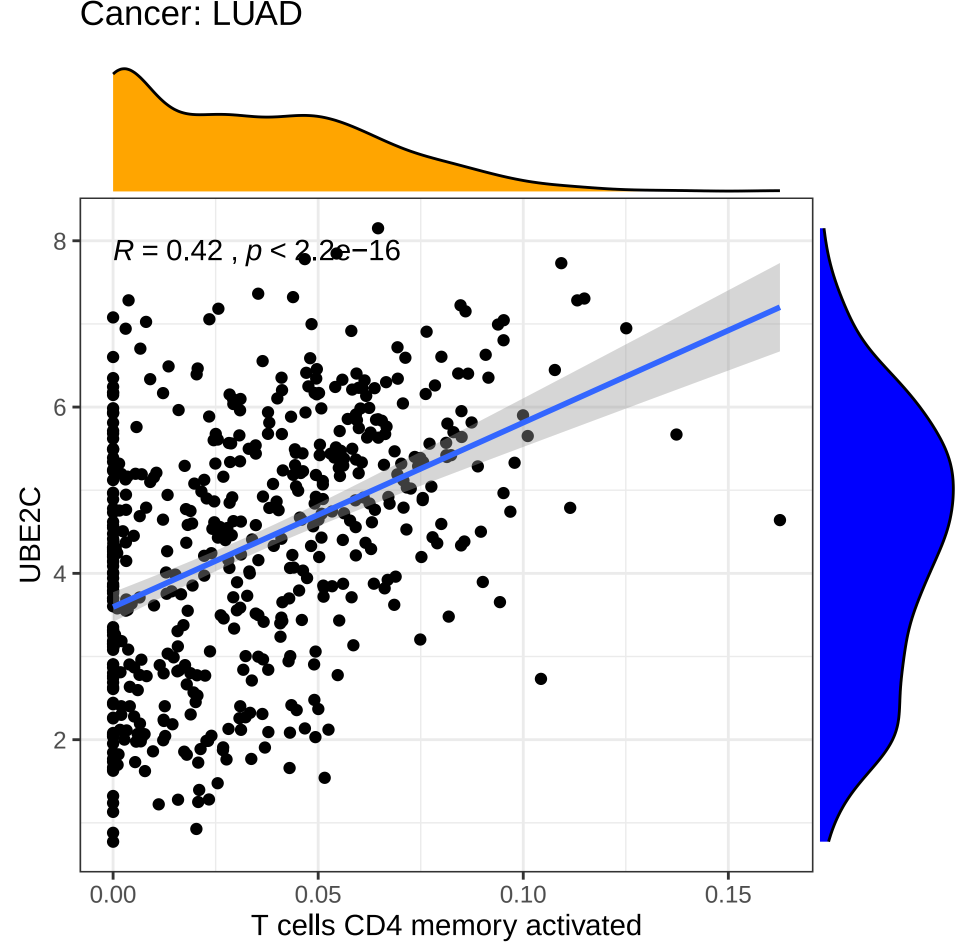
Cancer: LUAD
R = 0.42 , p < 2.2e−16
UBE2C
8
6
4
2
0.00
0.05
0.10
0.15
T cells CD4 memory activated

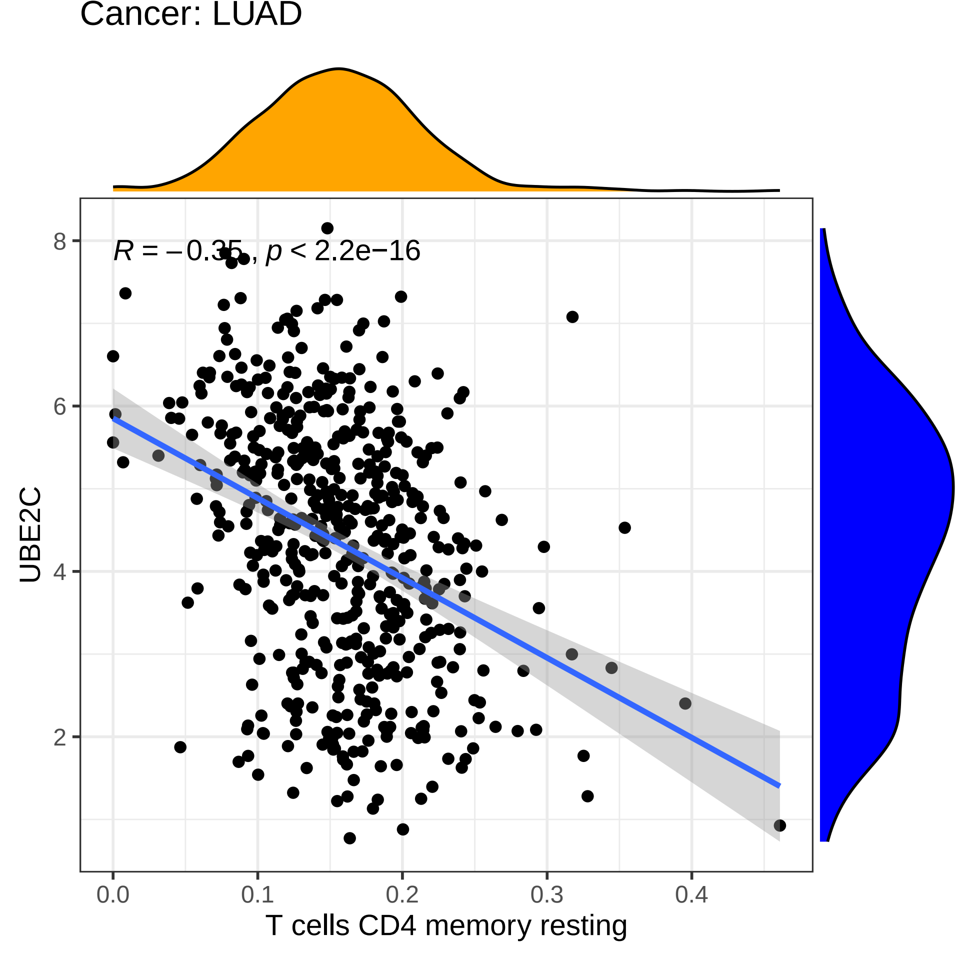
Cancer: LUAD
R = −0.35 , p < 2.2e−16
8
6
4
2
UBE2C
0.0
0.1
0.2
0.3
0.4
T cells CD4 memory resting

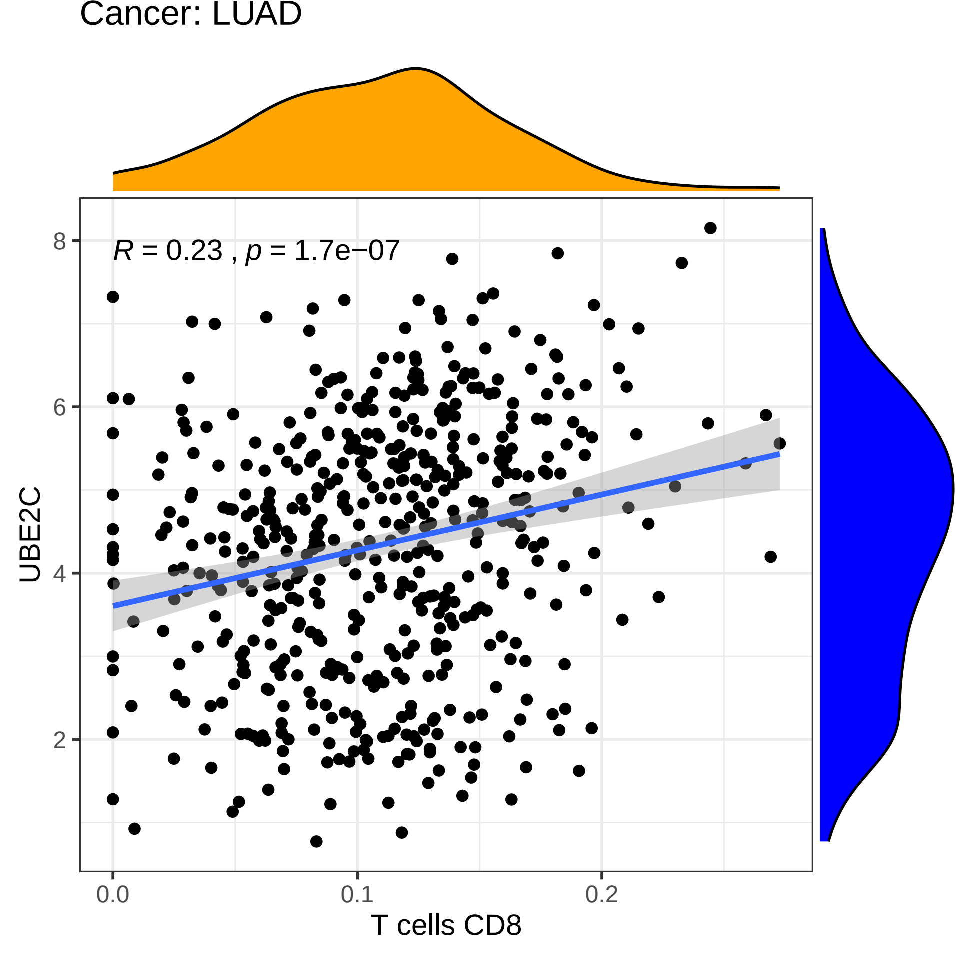
Cancer: LUAD
R = 0.23 , p = 1.7e−07
UBE2C
8
6
4
2
0.0
0.1
0.2
T cells CD8

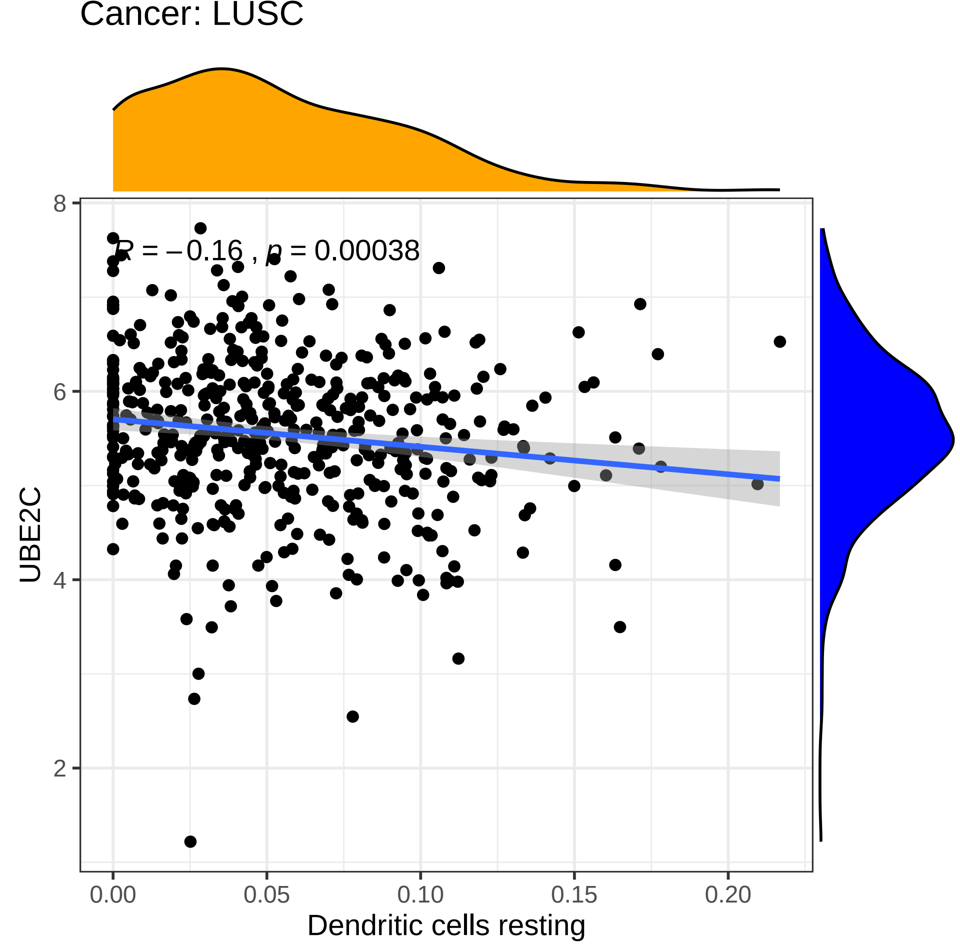

Cancer: LUSC
R = −0.16 , p = 0.00038
UBE2C
8
6
4
2
0.00
0.05
0.10
0.15
0.20
Dendritic cells resting

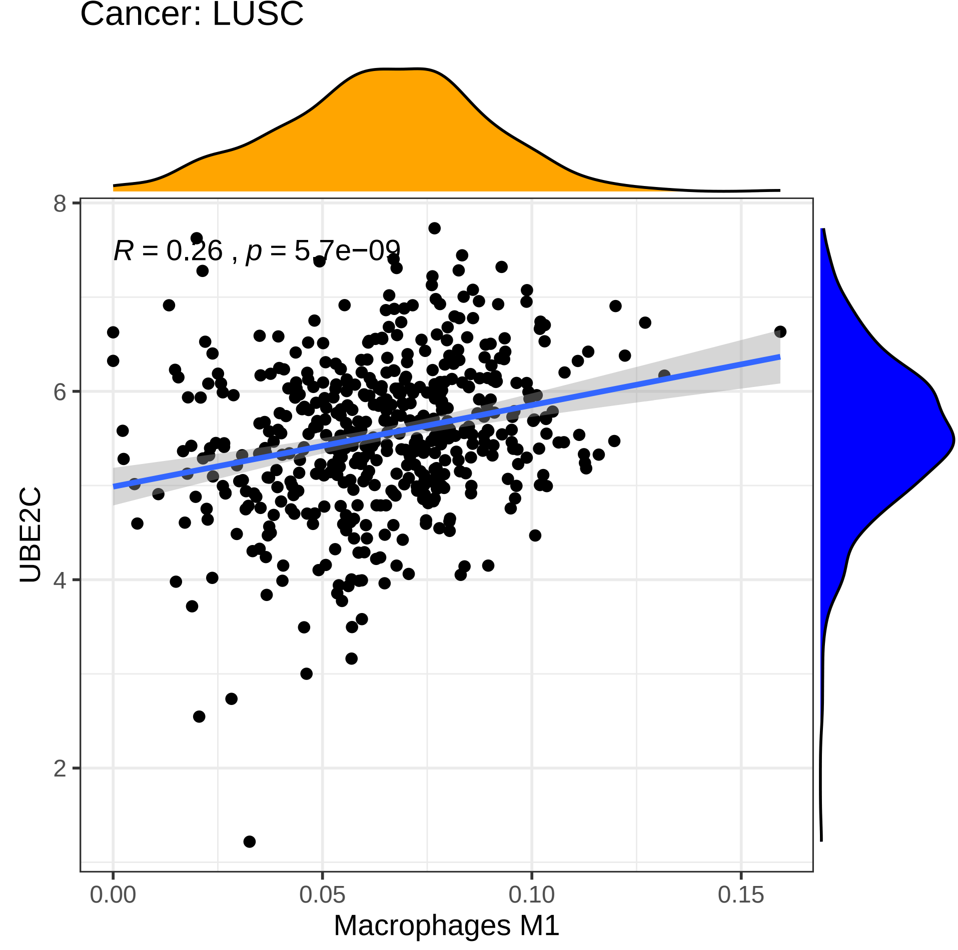

Cancer: LUSC
R = 0.26 , p = 5.7e−09
UBE2C
8
6
4
2
0.00
0.05
0.10
0.15
Macrophages M1

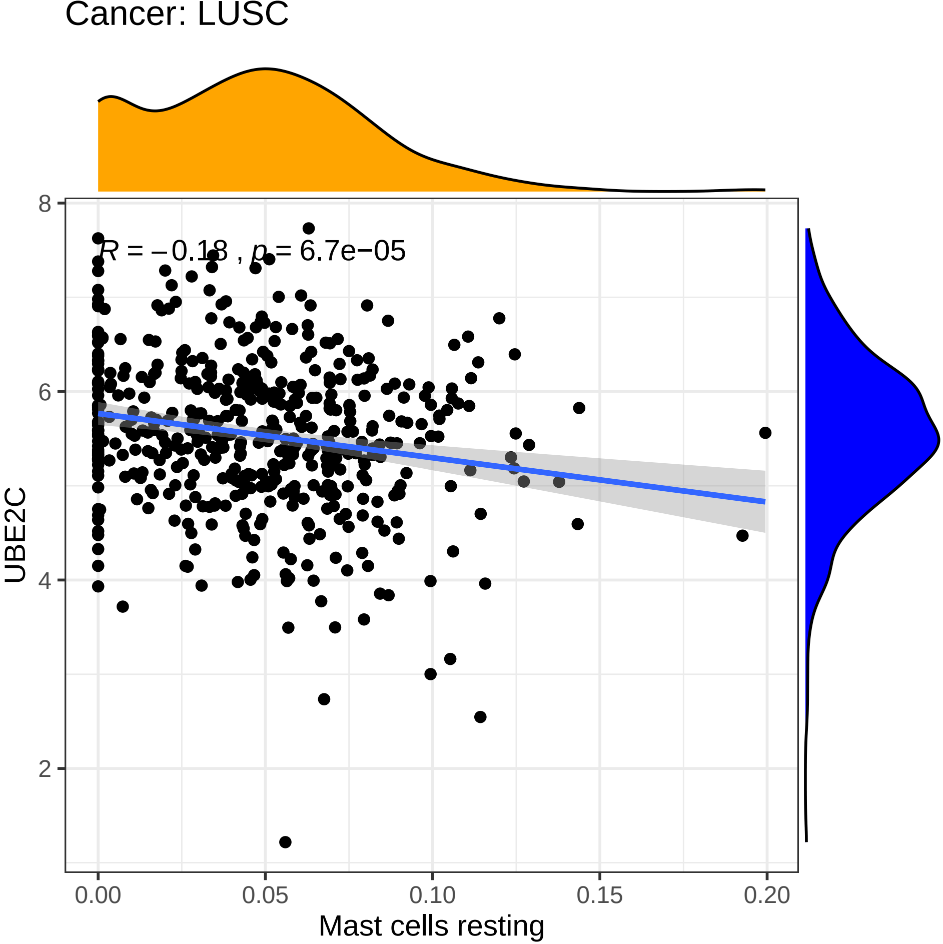

Cancer: LUSC
R = −0.18 , p = 6.7e−05
UBE2C
8
6
4
2
0.00
0.05
0.10
0.15
0.20
Mast cells resting

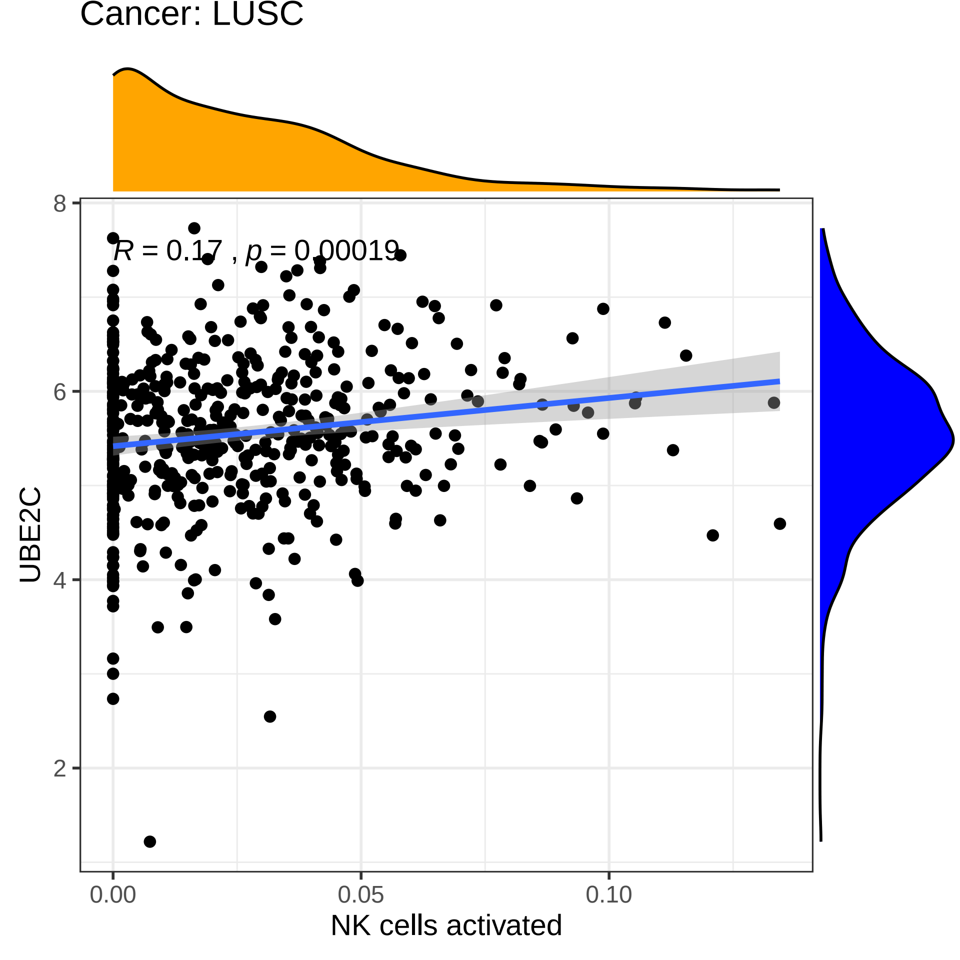

Cancer: LUSC
R = 0.17 , p = 0.00019
UBE2C
8
6
4
2
0.00
0.05
0.10
NK cells activated

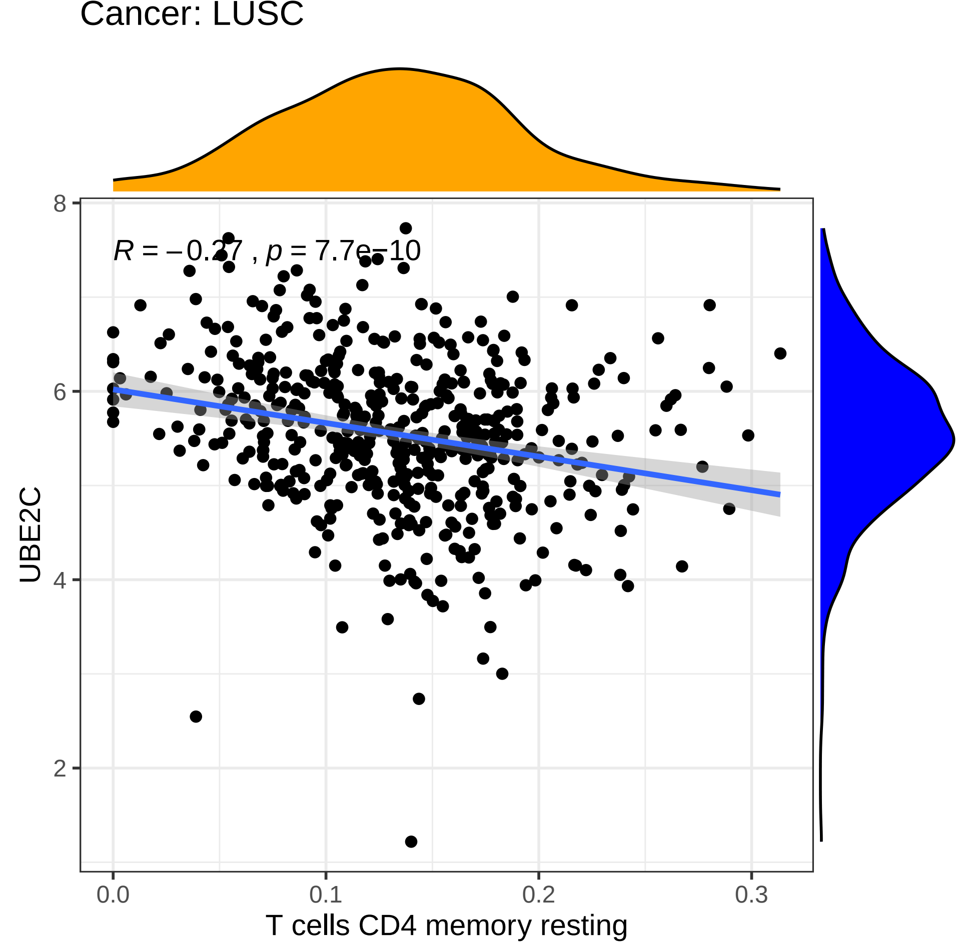

Cancer: LUSC
R = −0.27 , p = 7.7e−10
UBE2C
T cells CD4 memory resting
8
6
4
2
0.0
0.1
0.2
0.3

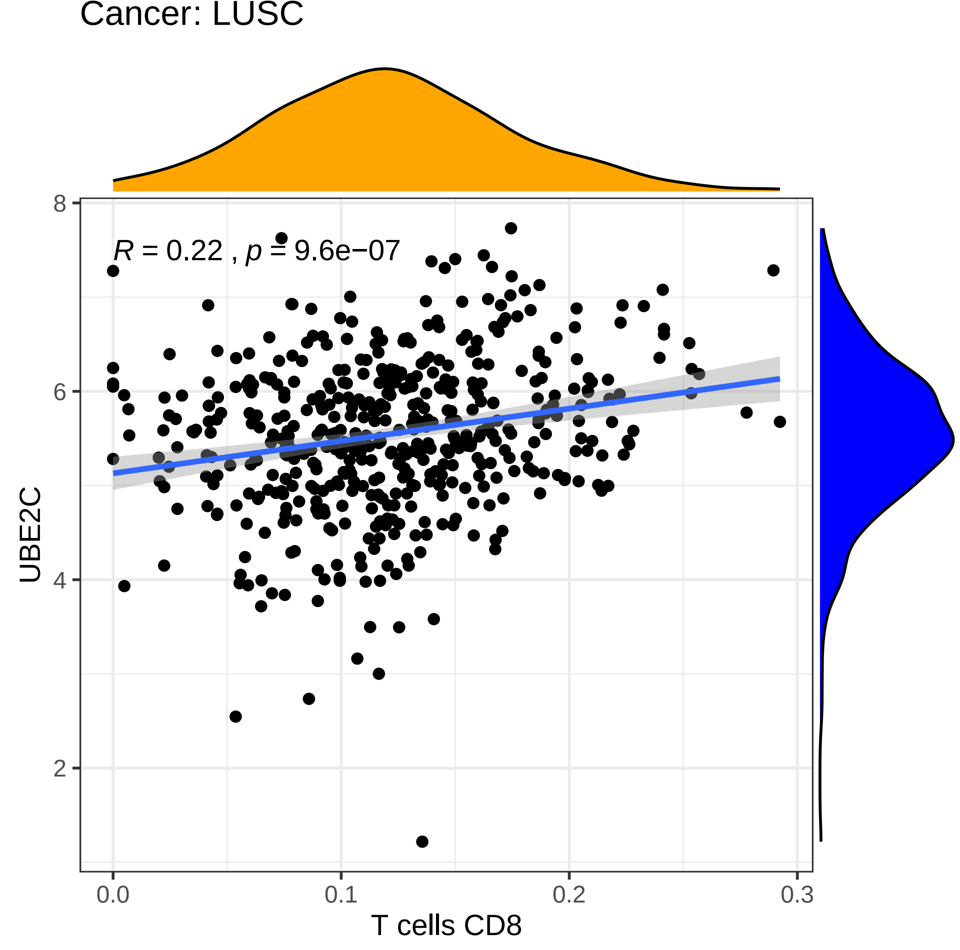

Cancer: LUSC
R = 0.22 , p = 9.6e−07
UBE2C
8
6
4
2
0.0
0.1
0.2
0.3
T cells CD8

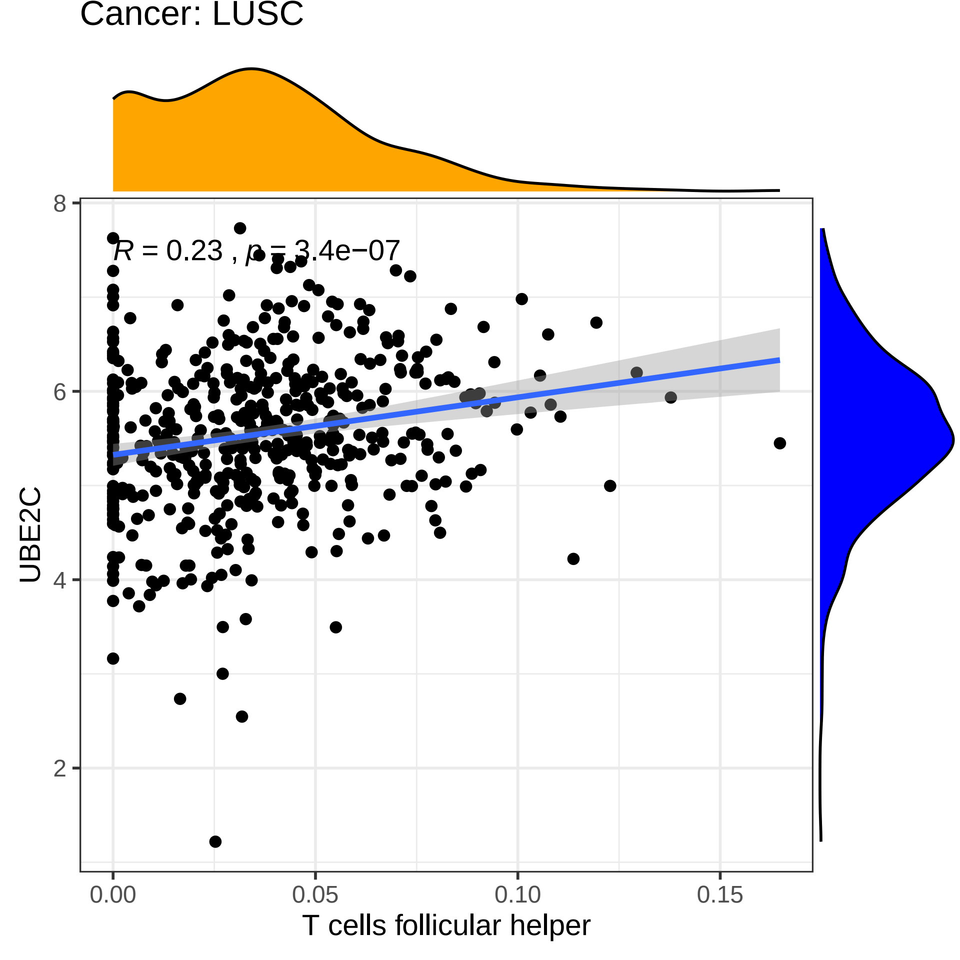

Cancer: LUSC
R = 0.23 , p = 3.4e−07
8
6
4
2
UBE2C
0.00
0.05
0.10
0.15
T cells follicular helper

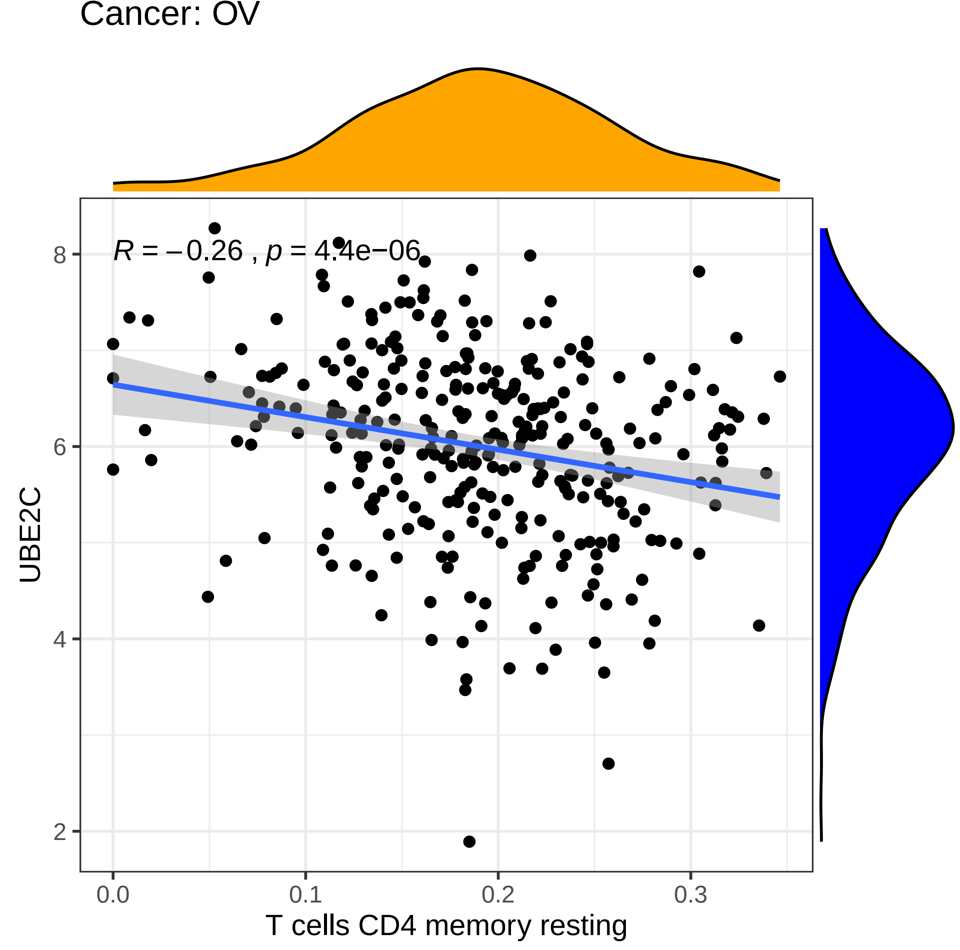

Cancer: OV
R = −0.26 , p = 4.4e−06
8
6
4
2
UBE2C
0.0
0.1
0.2
0.3
T cells CD4 memory resting

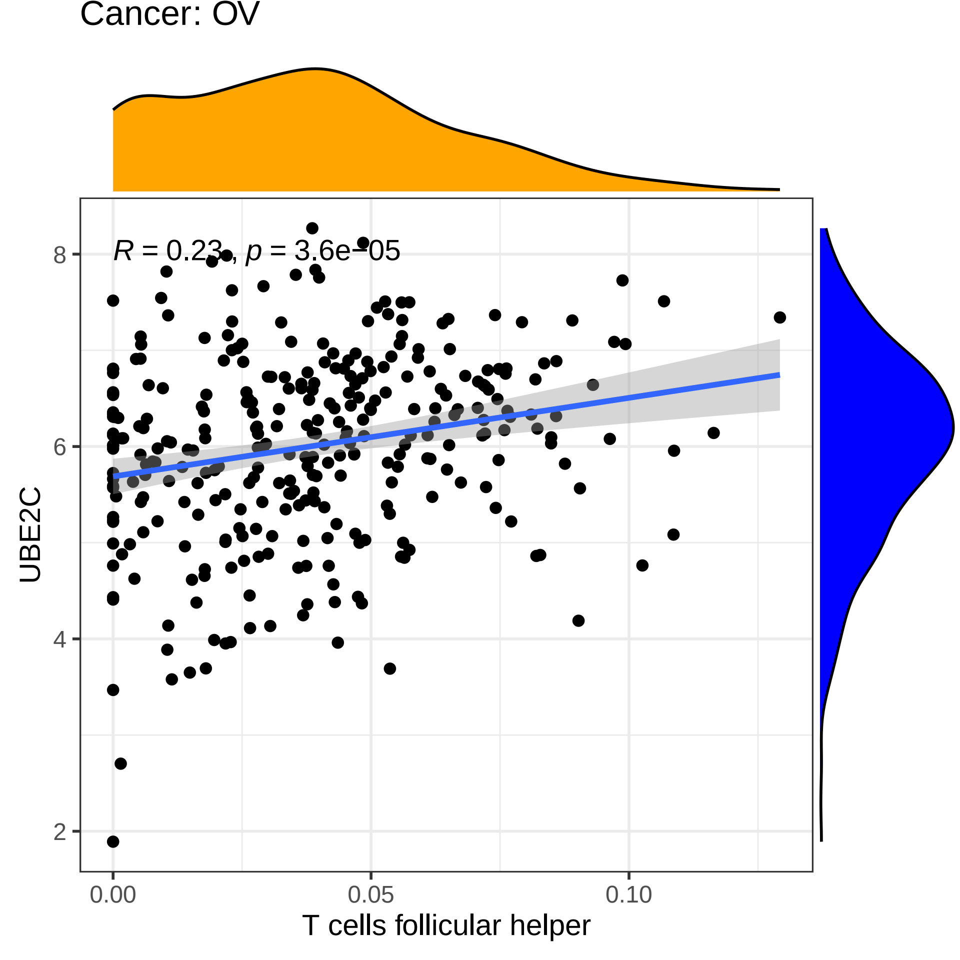

Cancer: OV
R = 0.23 , p = 3.6e−05
8
6
4
2
UBE2C
0.00
0.05
0.10
T cells follicular helper

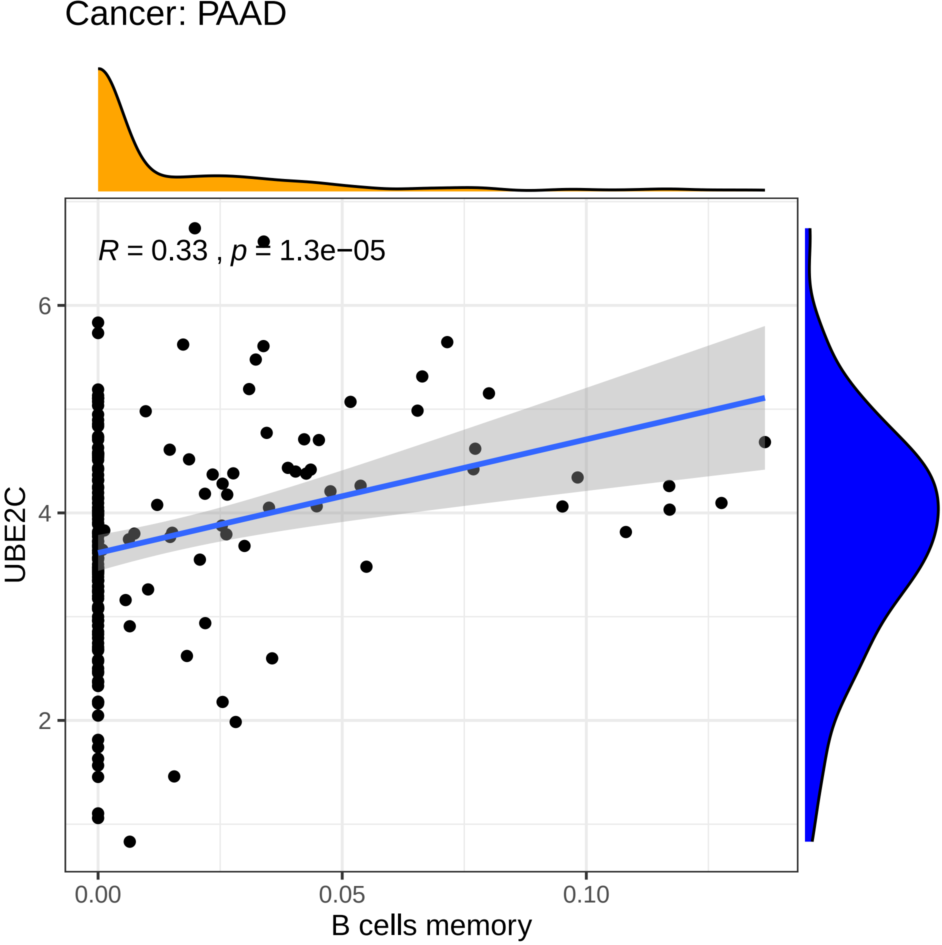
Cancer: PAAD
R = 0.33 , p = 1.3e−05
6
4
2
0.00
0.05
0.10
UBE2C
B cells memory

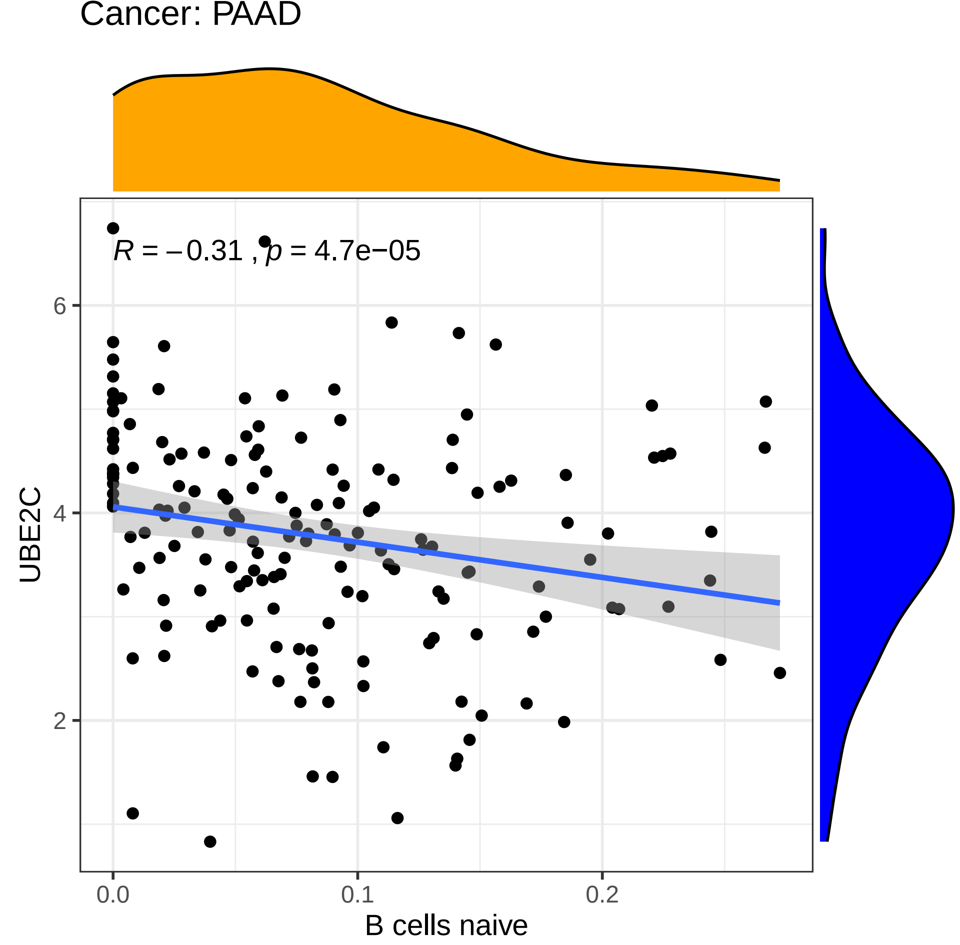

Cancer: PAAD
R = −0.31 , p = 4.7e−05
UBE2C
2
4
6
0.0
0.1
0.2
B cells naive

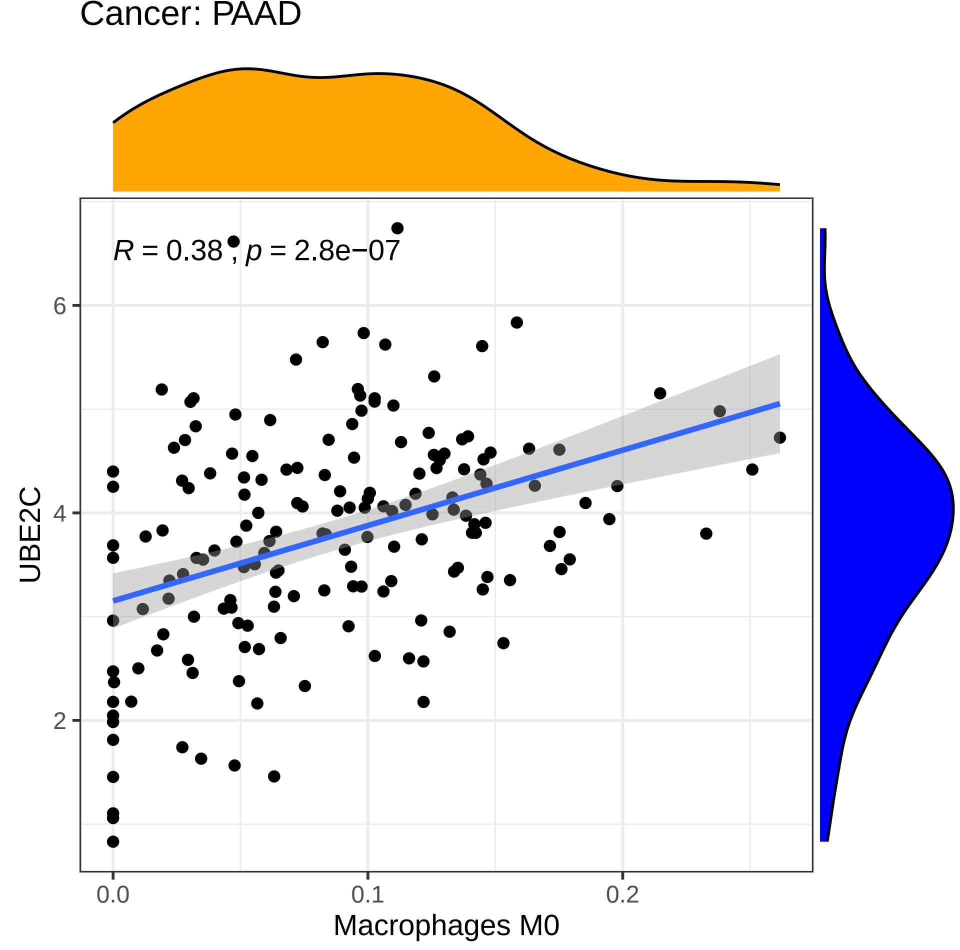

Cancer: PAAD
R = 0.38 , p = 2.8e−07
UBE2C
6
4
2
0.0
0.1
0.2
Macrophages M0

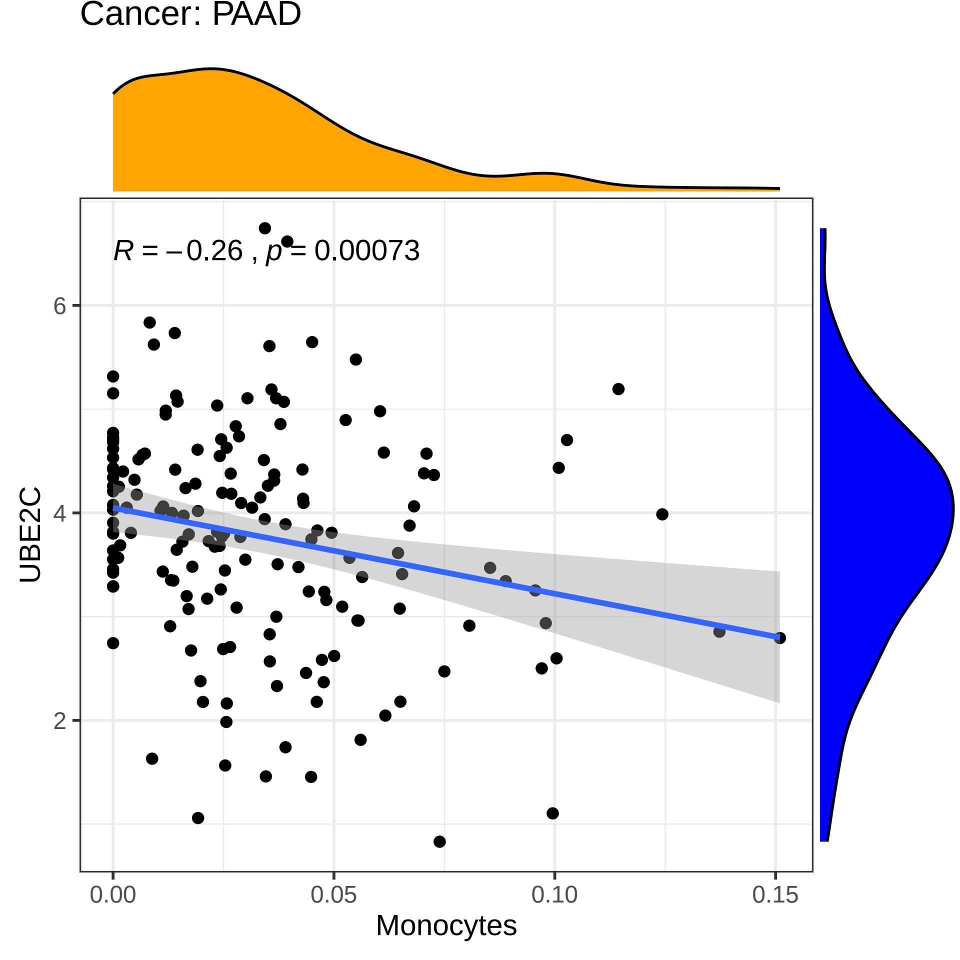
Cancer: PAAD
R = −0.26 , p = 0.00073
UBE2C
6
4
2
0.00
0.05
0.10
0.15
Monocytes

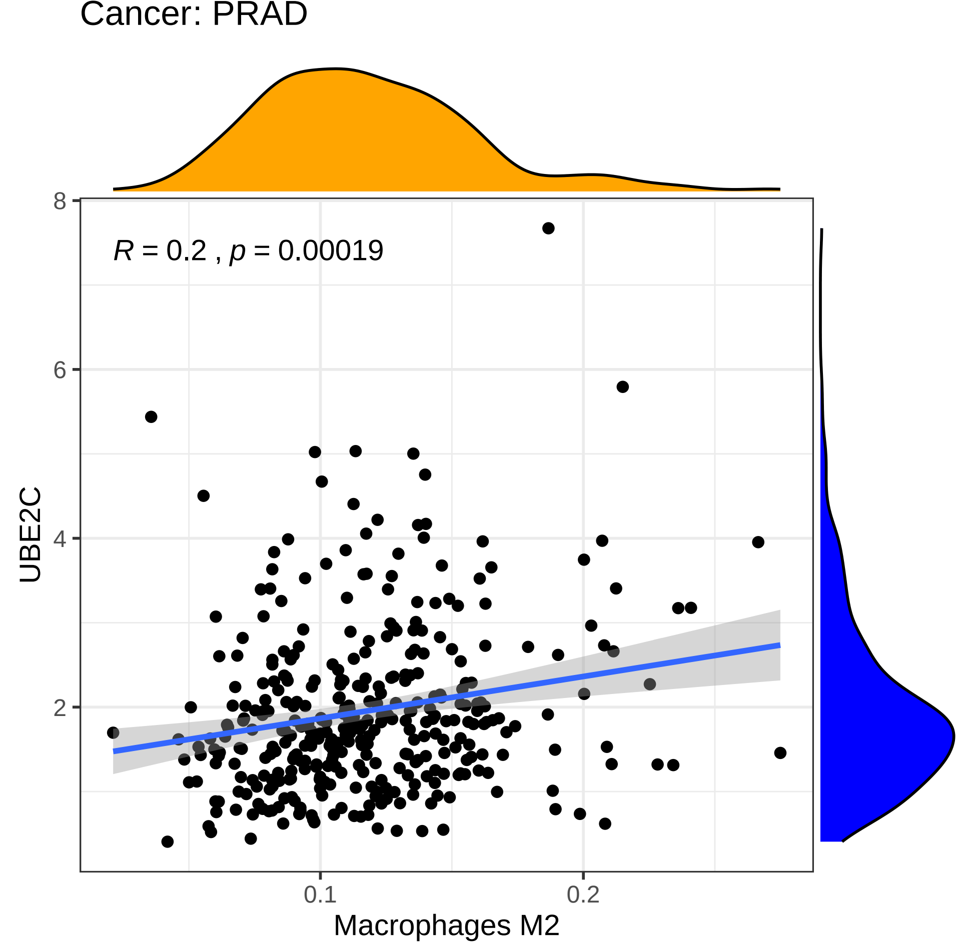
Cancer: PRAD
R = 0.2 , p = 0.00019
UBE2C
Macrophages M2
8
6
4
2
0.1
0.2

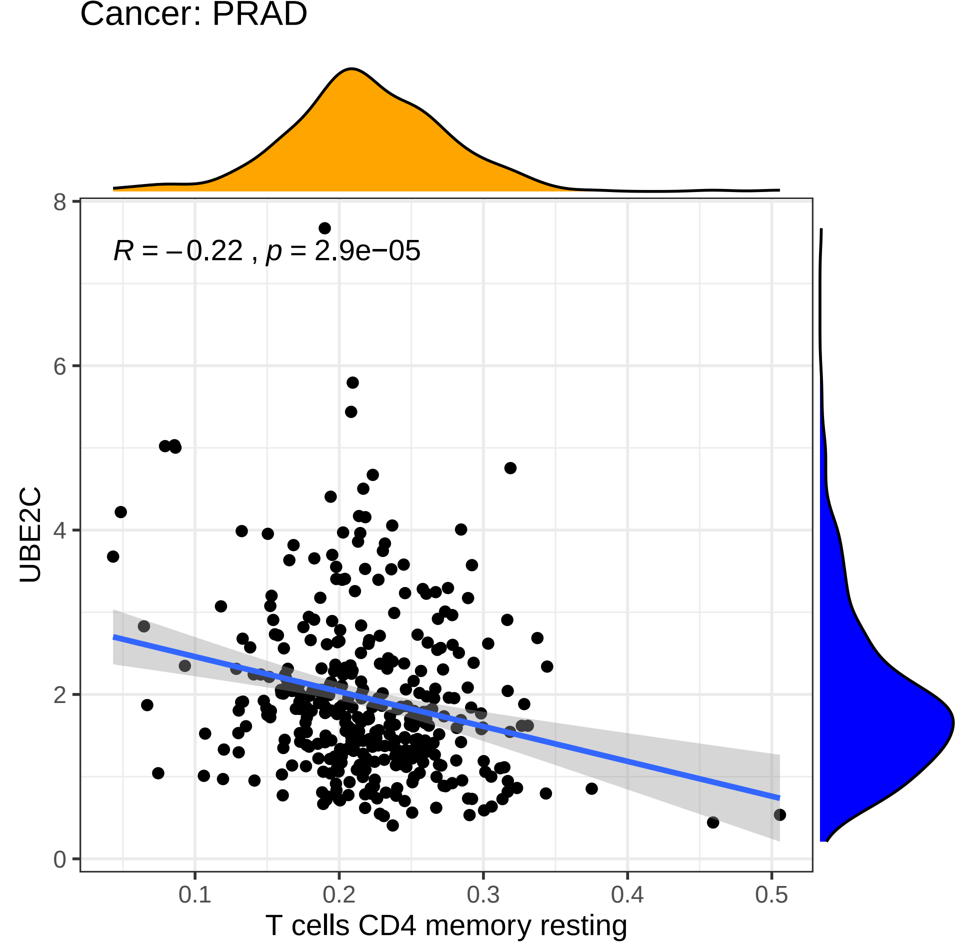

Cancer: PRAD
R = −0.22 , p = 2.9e−05
UBE2C
0
2
4
6
8
0.1
0.2
0.3
0.4
0.5
T cells CD4 memory resting

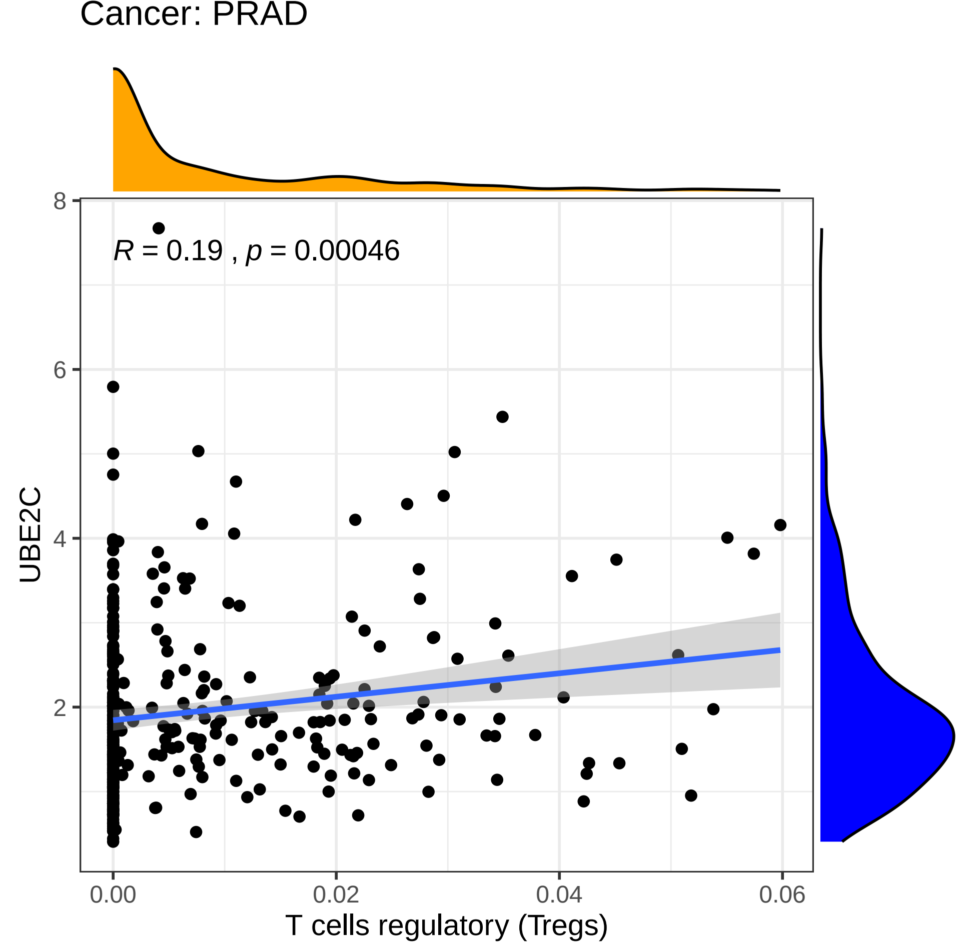

Cancer: PRAD
R = 0.19 , p = 0.00046
UBE2C
T cells regulatory (Tregs)
8
6
4
2
0.00
0.02
0.04
0.06

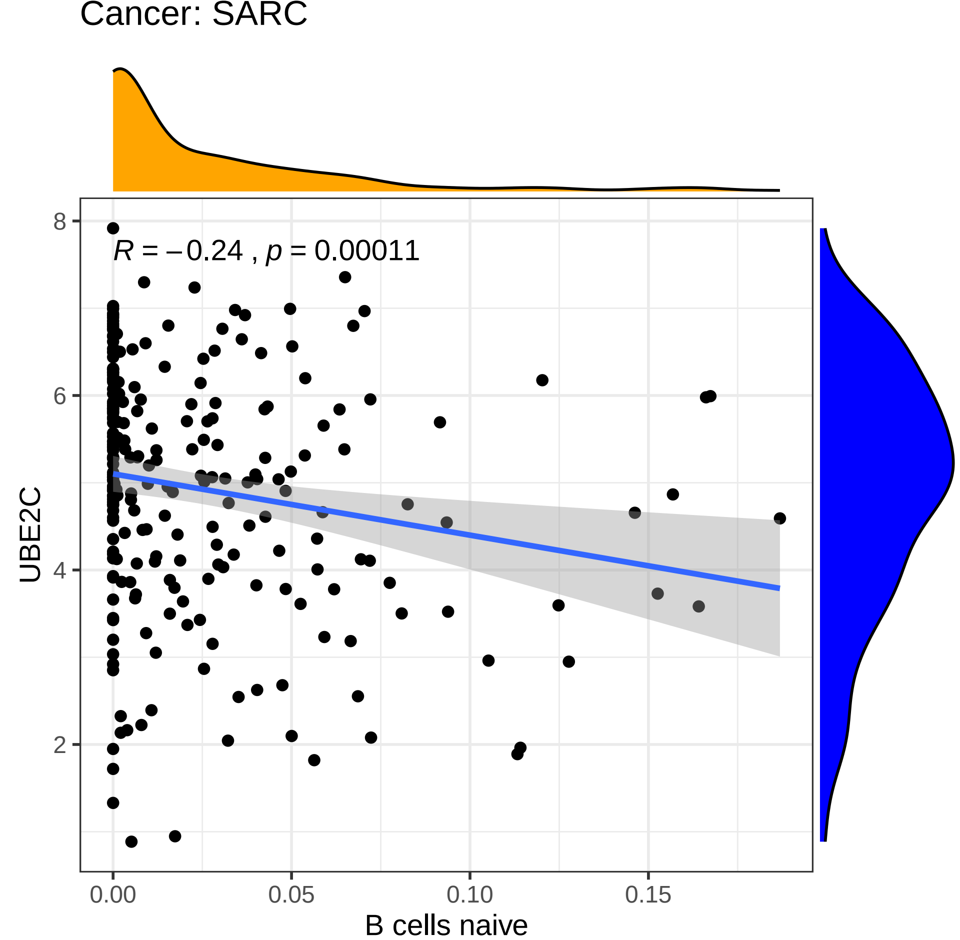
Cancer: SARC
R = −0.24 , p = 0.00011
UBE2C
8
6
4
2
0.00
0.05
0.10
0.15
B cells naive

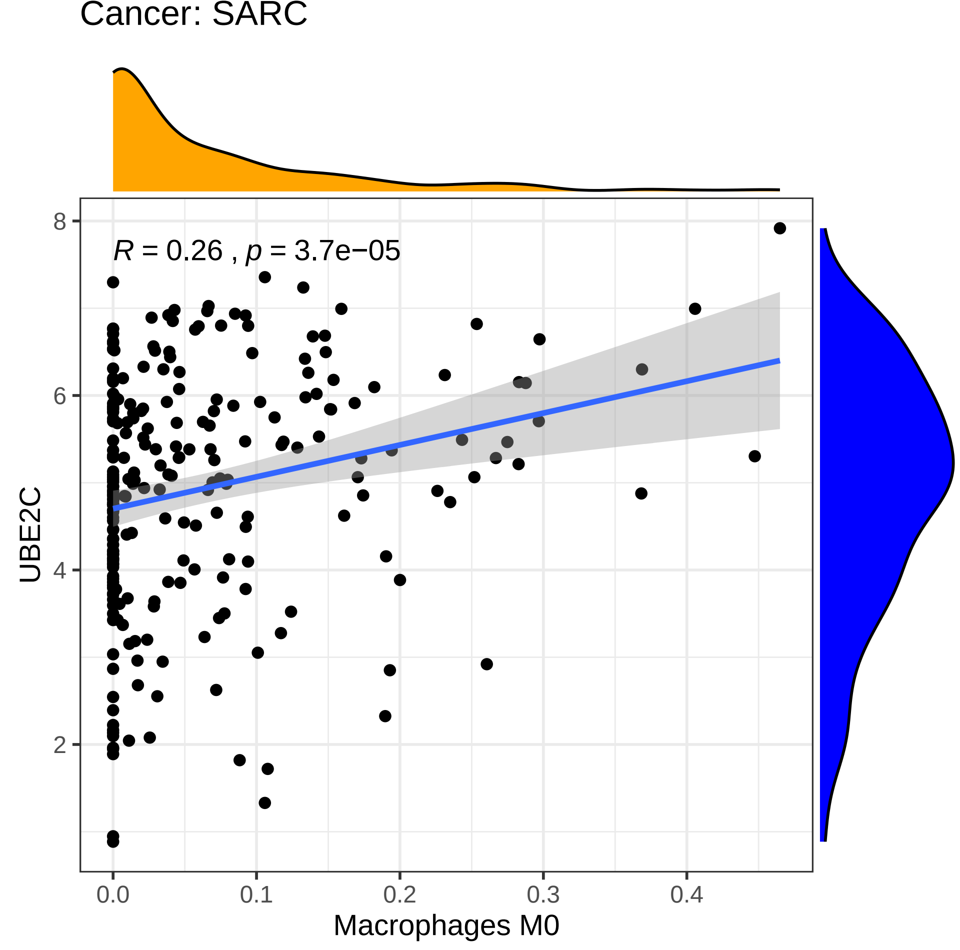

Cancer: SARC
R = 0.26 , p = 3.7e−05
UBE2C
8
6
4
2
0.0
0.1
0.2
0.3
0.4
Macrophages M0

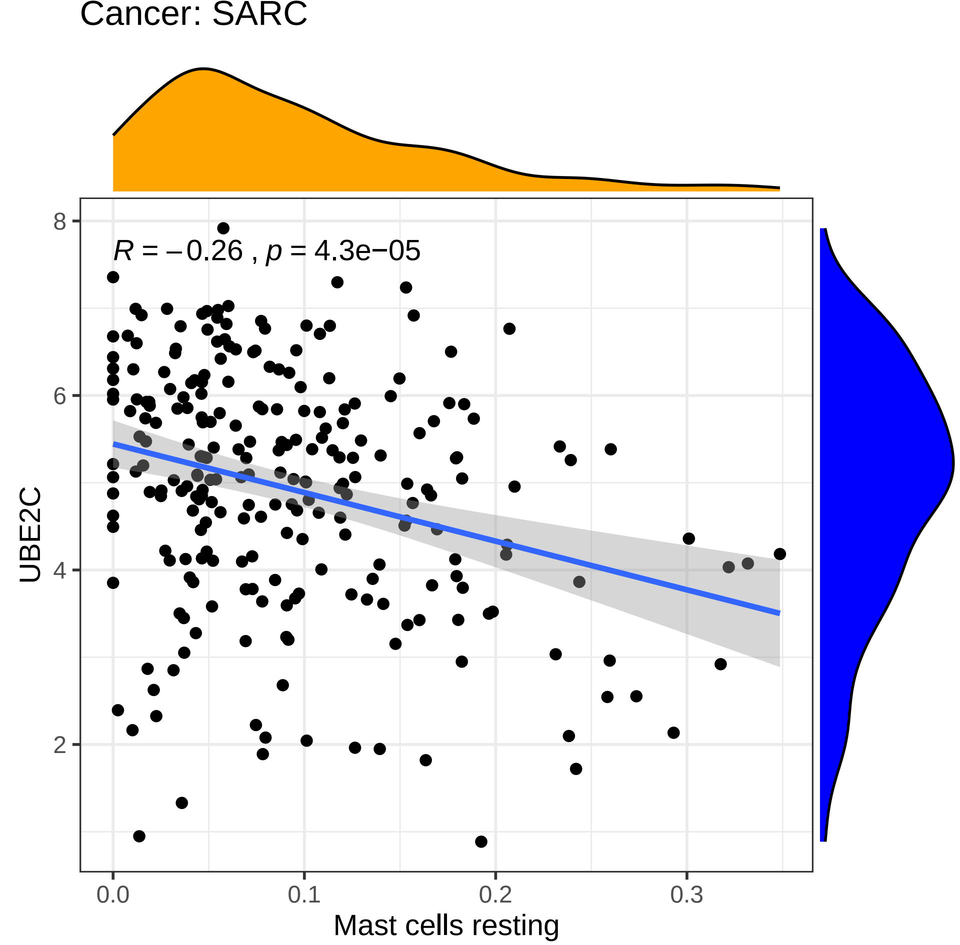

Cancer: SARC
R = −0.26 , p = 4.3e−05
UBE2C
Mast cells resting
8
6
4
2
0.0
0.1
0.2
0.3

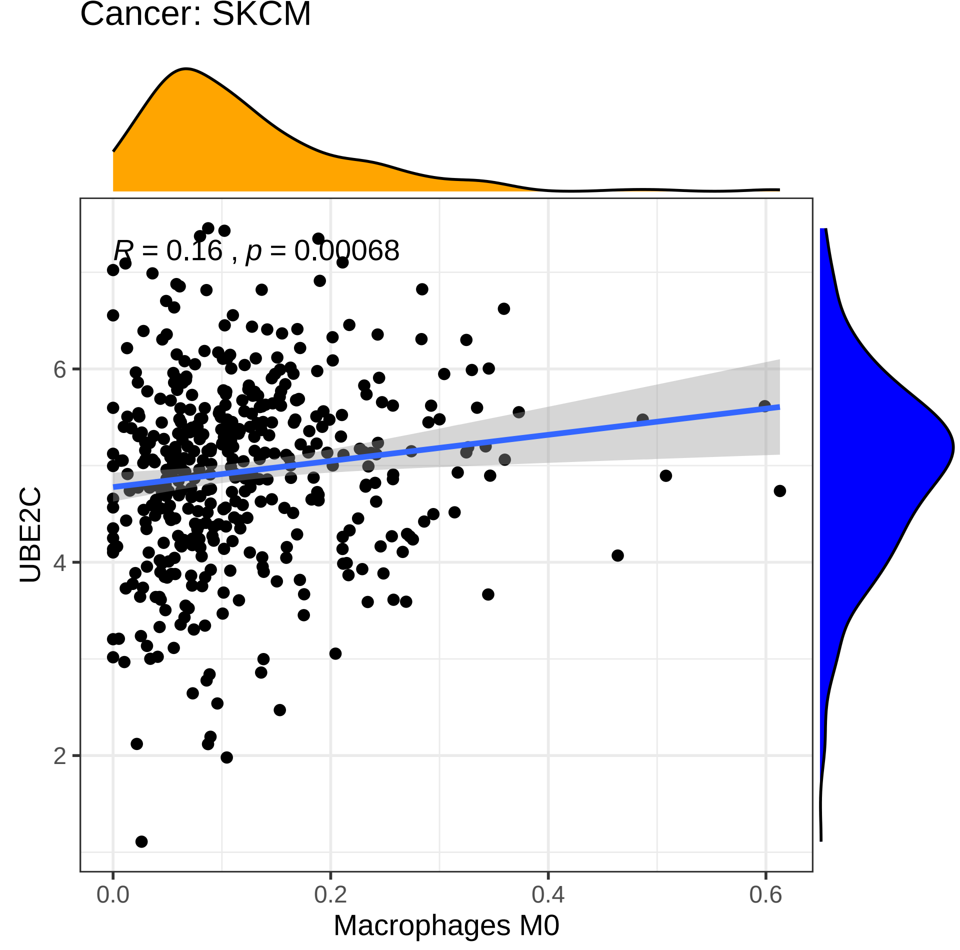

Cancer: SKCM
R = 0.16 , p = 0.00068
UBE2C
2
4
6
0.0
0.2
0.4
0.6
Macrophages M0

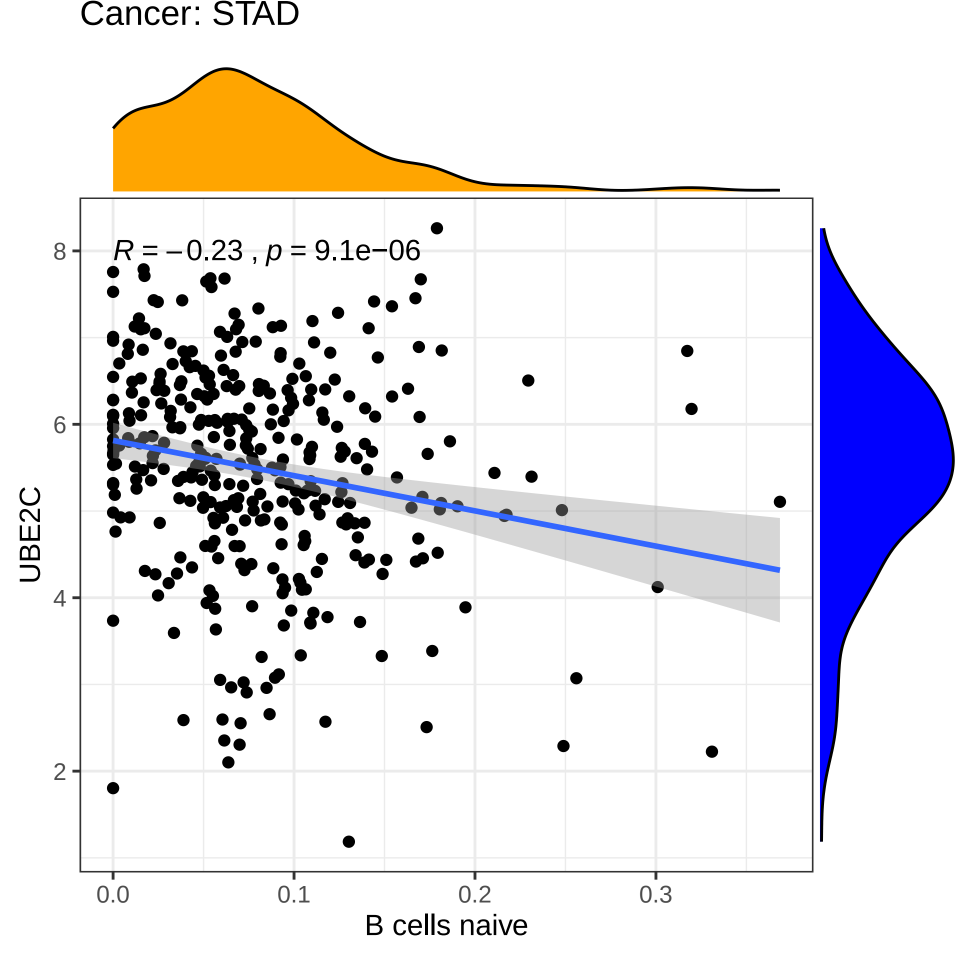
Cancer: STAD
R = −0.23 , p = 9.1e−06
UBE2C
8
6
4
2
0.0
0.1
0.2
0.3
B cells naive

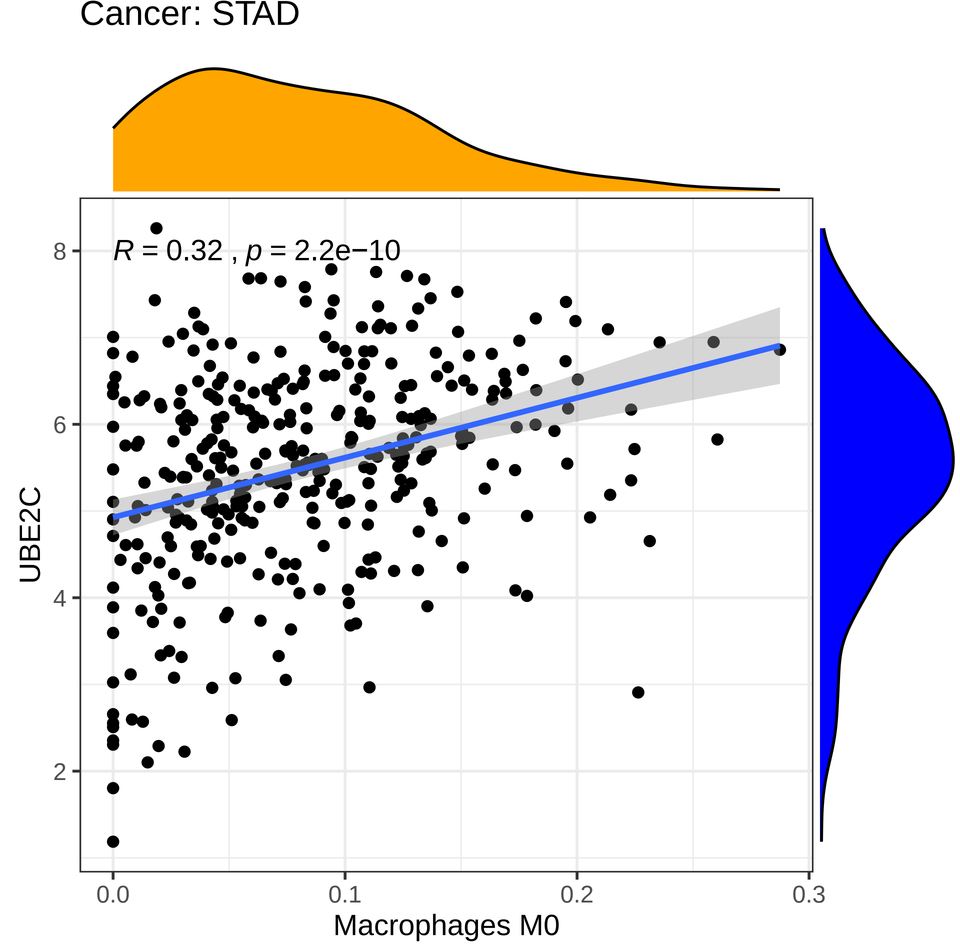

Cancer: STAD
R = 0.32 , p = 2.2e−10
UBE2C
Macrophages M0
0.0
0.1
0.2
0.3
2
4
6
8

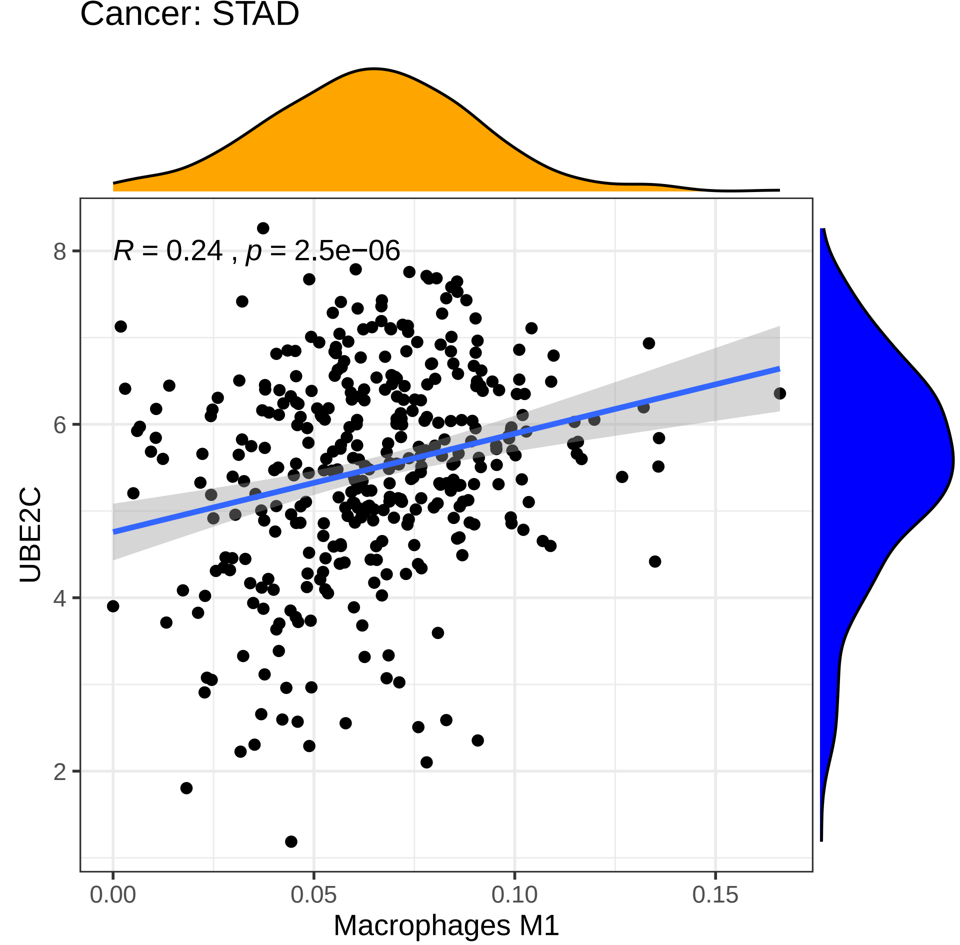
Cancer: STAD
R = 0.24 , p = 2.5e−06
UBE2C
8
6
4
2
0.00
0.05
0.10
0.15
Macrophages M1

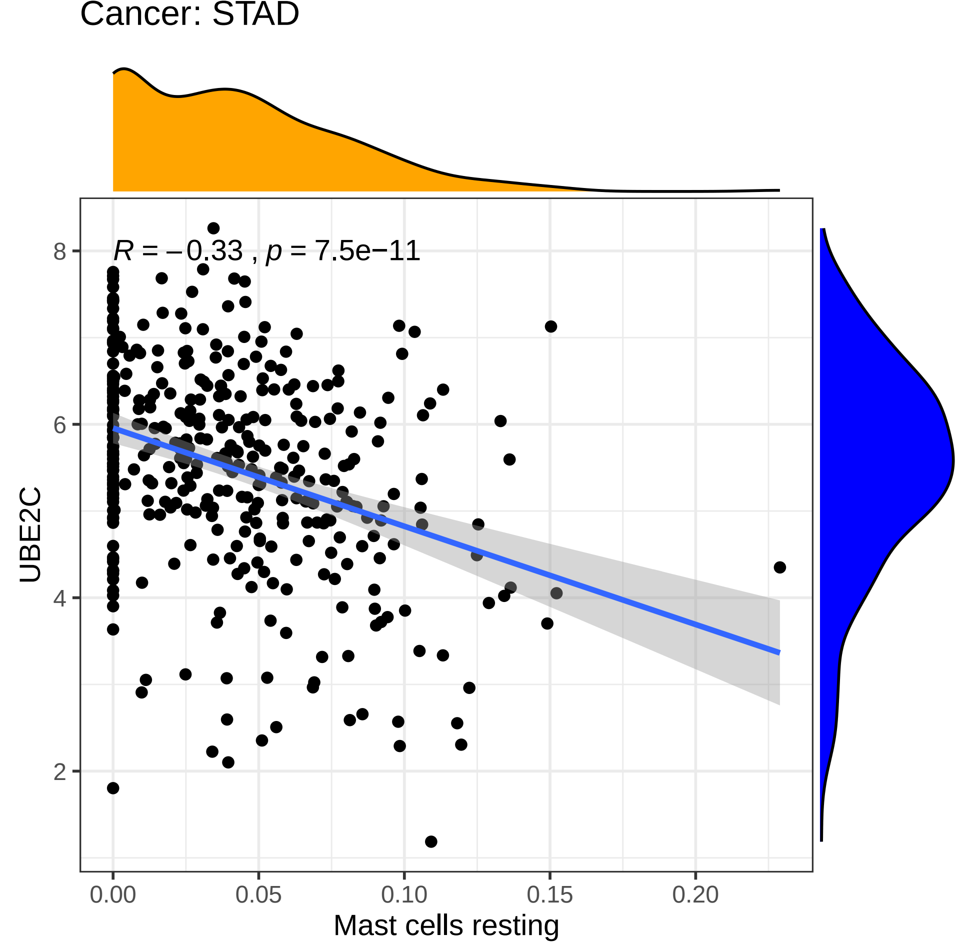
Cancer: STAD
R = −0.33 , p = 7.5e−11
UBE2C
8
6
4
2
0.00
0.05
0.10
0.15
0.20
Mast cells resting

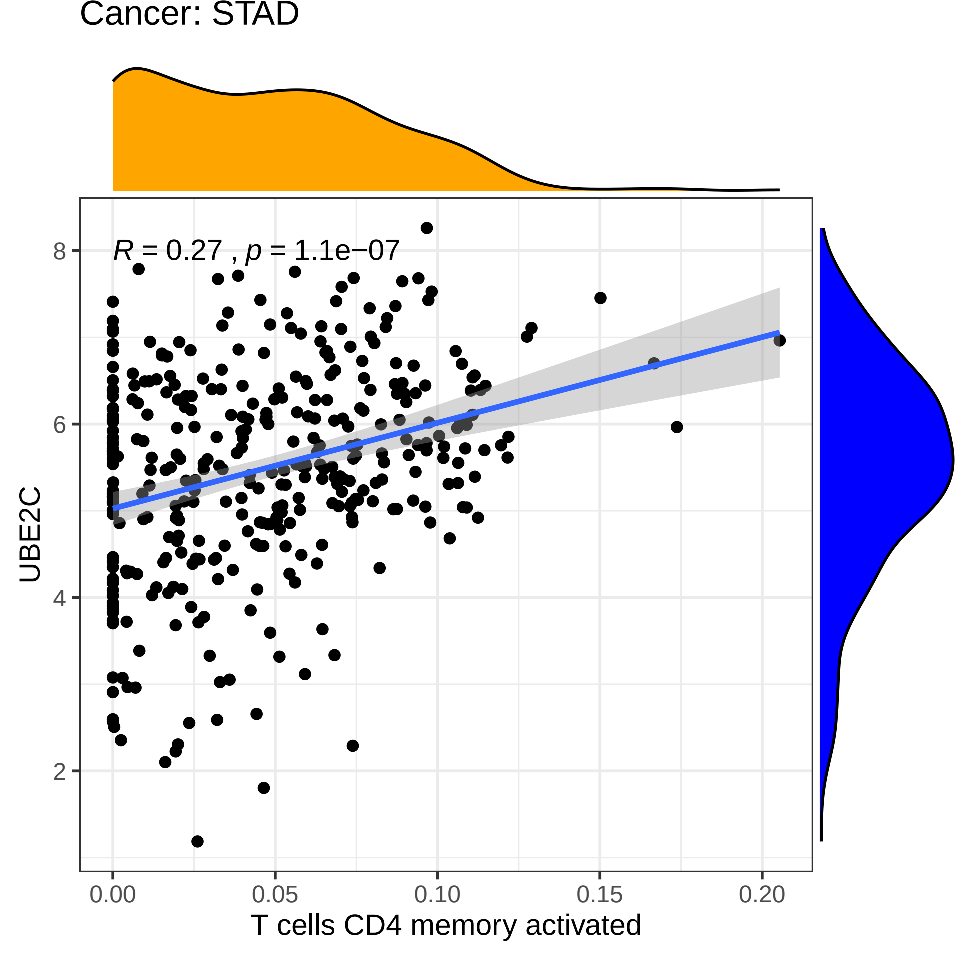

Cancer: STAD
R = 0.27 , p = 1.1e−07
8
6
4
2
UBE2C
0.00
0.05
0.10
0.15
0.20
T cells CD4 memory activated

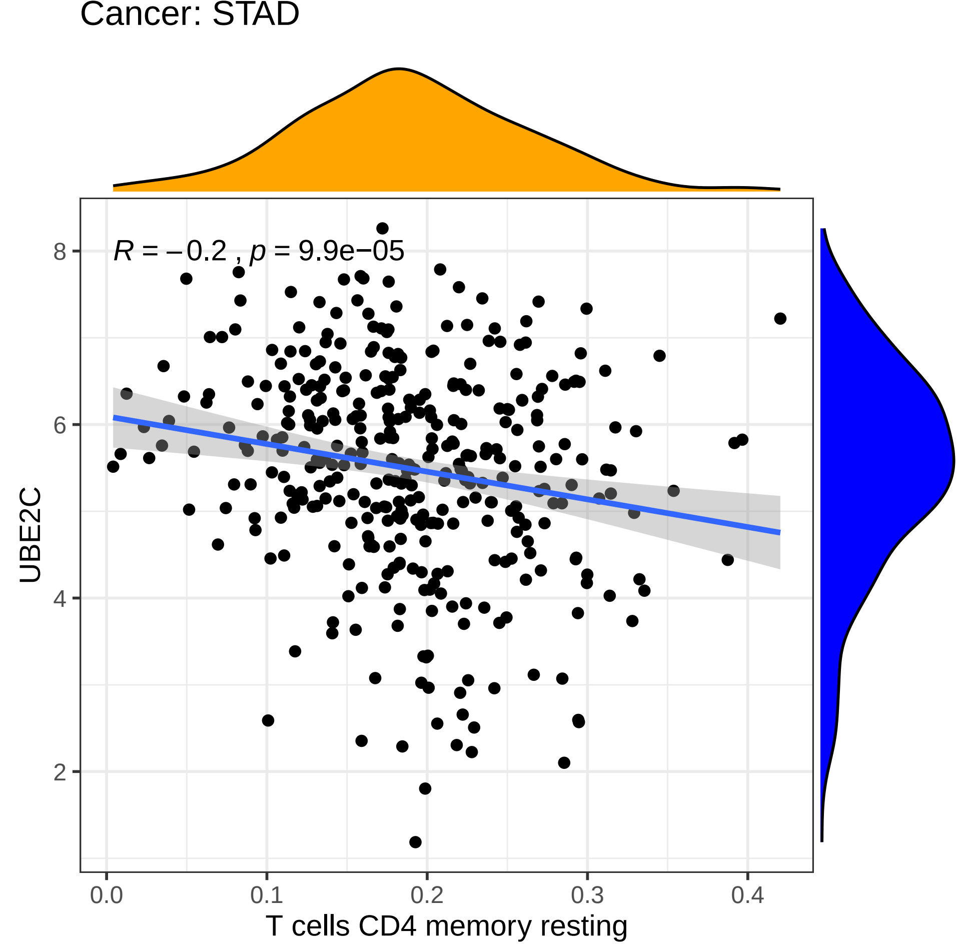

Cancer: STAD
R = −0.2 , p = 9.9e−05
UBE2C
8
6
4
2
0.0
0.1
0.2
0.3
0.4
T cells CD4 memory resting

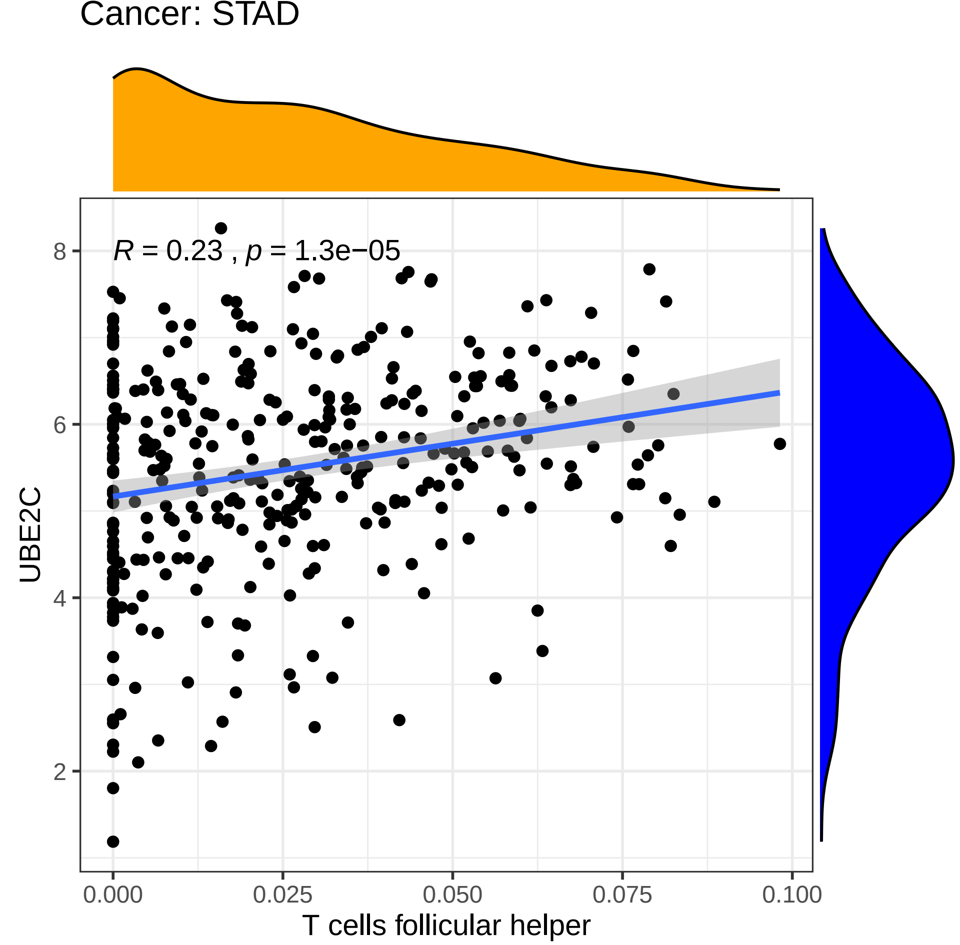
Cancer: STAD
R = 0.23 , p = 1.3e−05
UBE2C
8
6
4
2
0.000
0.025
0.050
0.075
0.100
T cells follicular helper

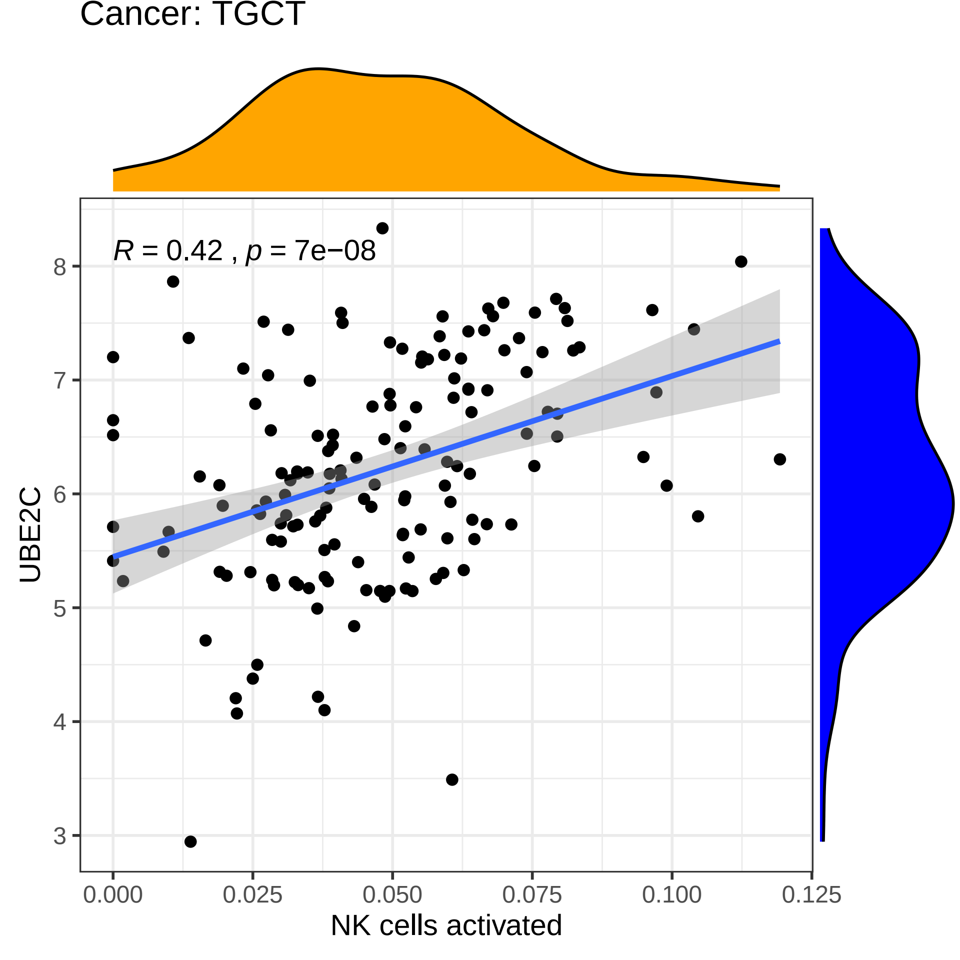
Cancer: TGCT
R = 0.42 , p = 7e−08
UBE2C
8
7
6
5
4
3
0.000
0.025
0.050
0.075
0.100
0.125
NK cells activated

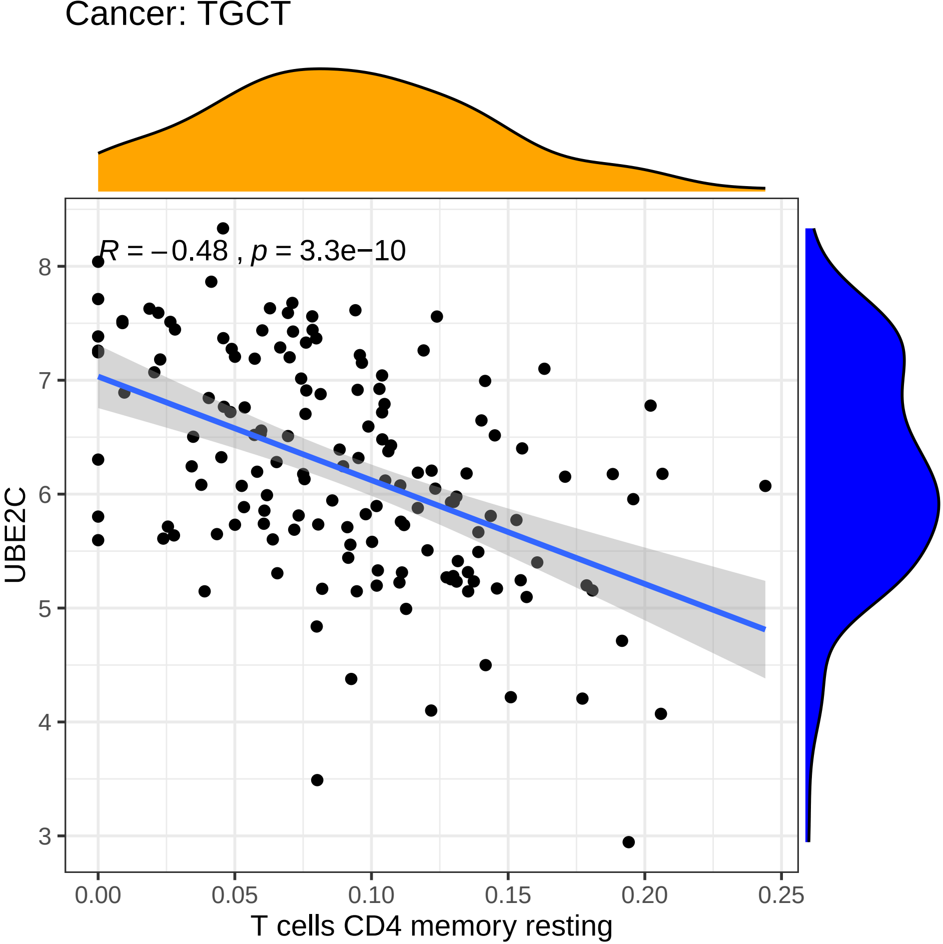

Cancer: TGCT
R = −0.48 , p = 3.3e−10
UBE2C
T cells CD4 memory resting
3
4
5
6
7
8
0.00
0.05
0.10
0.15
0.20
0.25

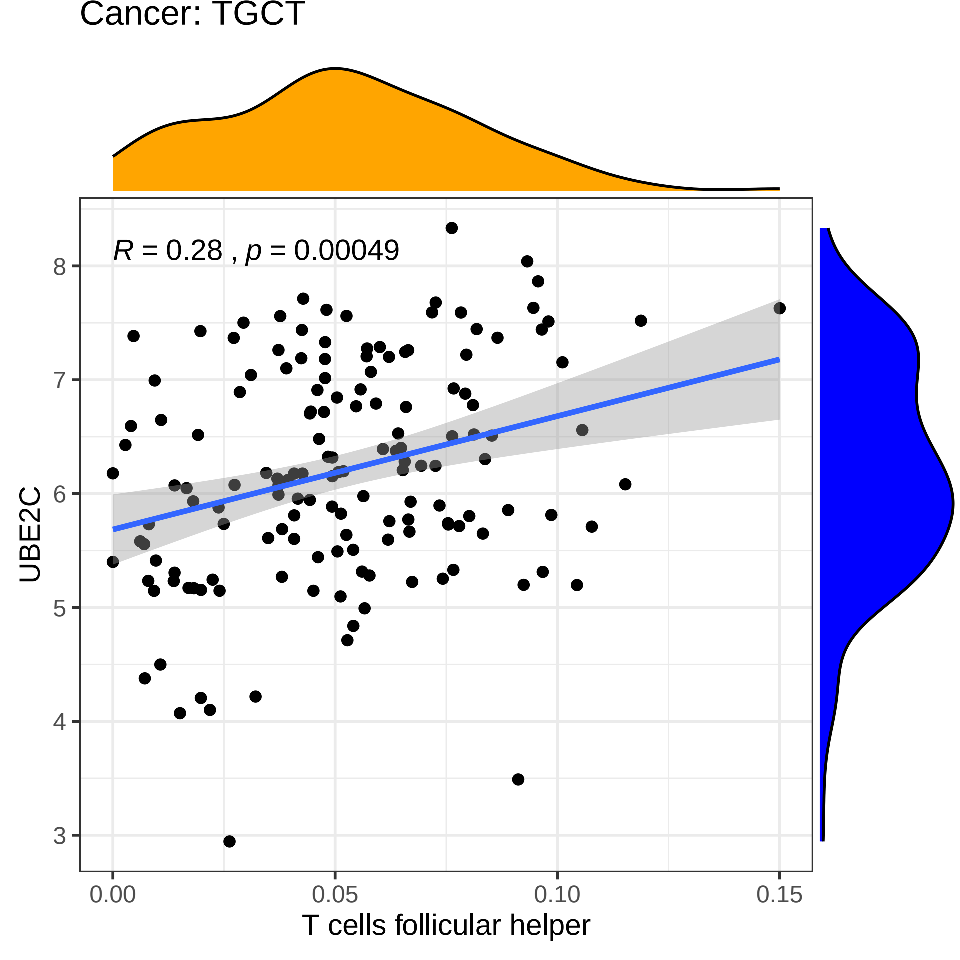

Cancer: TGCT
R = 0.28 , p = 0.00049
UBE2C
8
7
6
5
4
3
0.00
0.05
0.10
0.15
T cells follicular helper

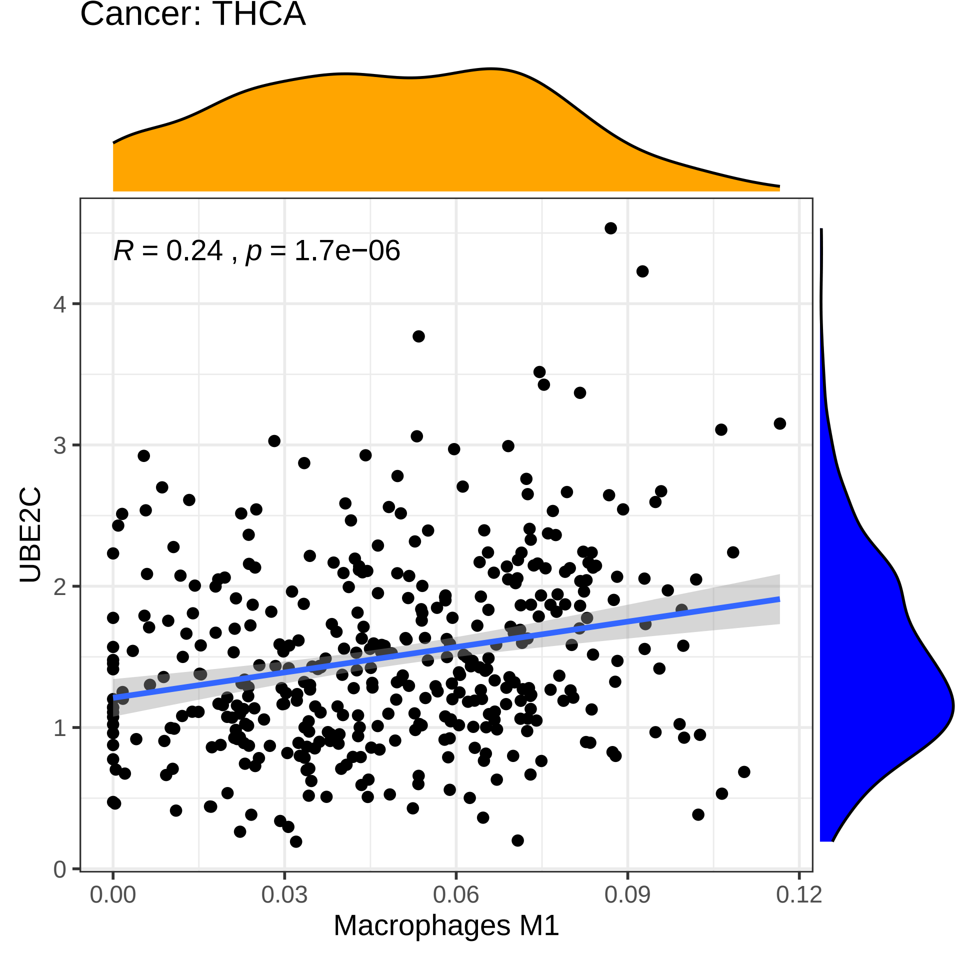
Cancer: THCA
R = 0.24 , p = 1.7e−06
UBE2C
0
1
2
3
4
0.00
0.03
0.06
0.09
0.12
Macrophages M1

Cancer: THCA

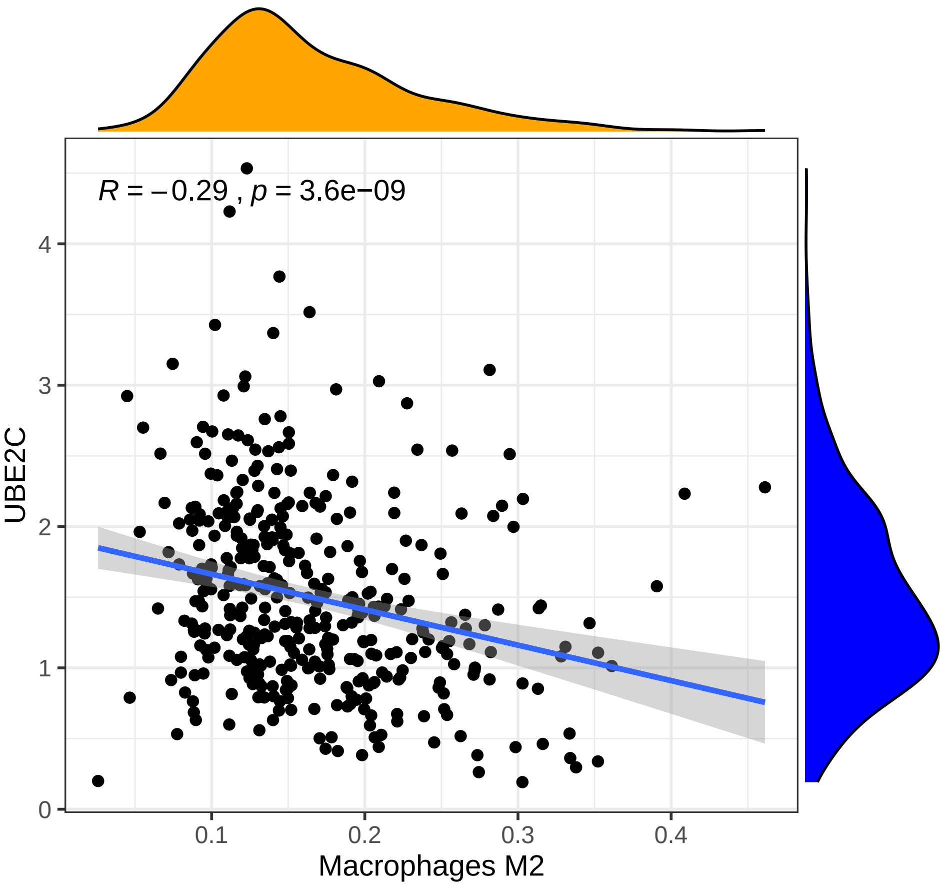

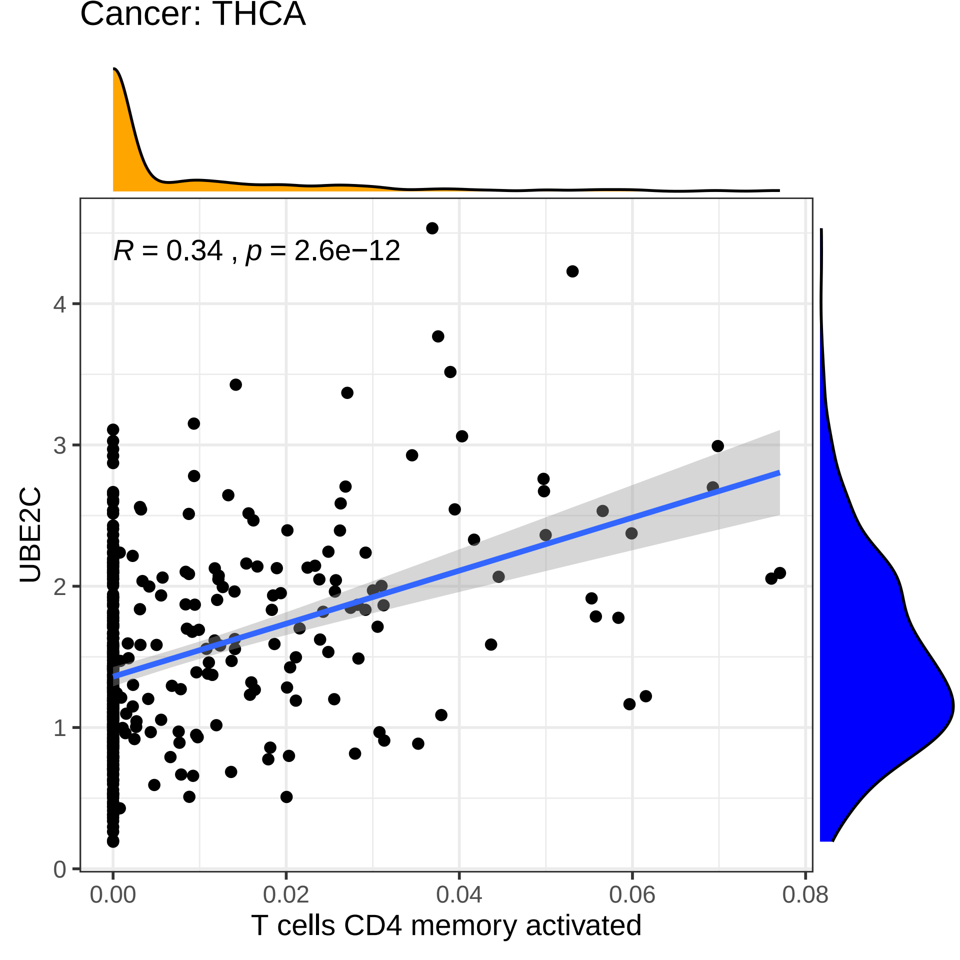
Cancer: THCA
R = 0.34 , p = 2.6e−12
UBE2C
T cells CD4 memory activated
0
1
2
3
4
0.00
0.02
0.04
0.06
0.08

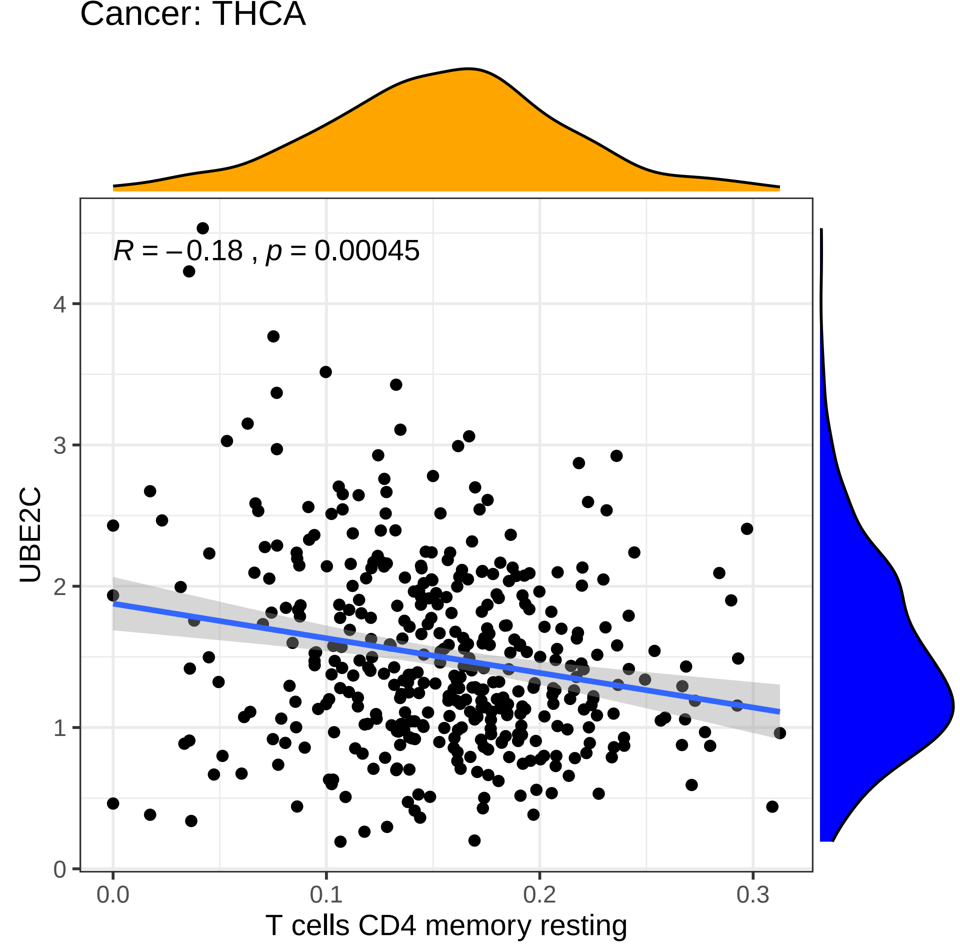

Cancer: THCA
R = −0.18 , p = 0.00045
UBE2C
0
1
2
3
4
0.0
0.1
0.2
0.3
T cells CD4 memory resting

Cancer: THCA

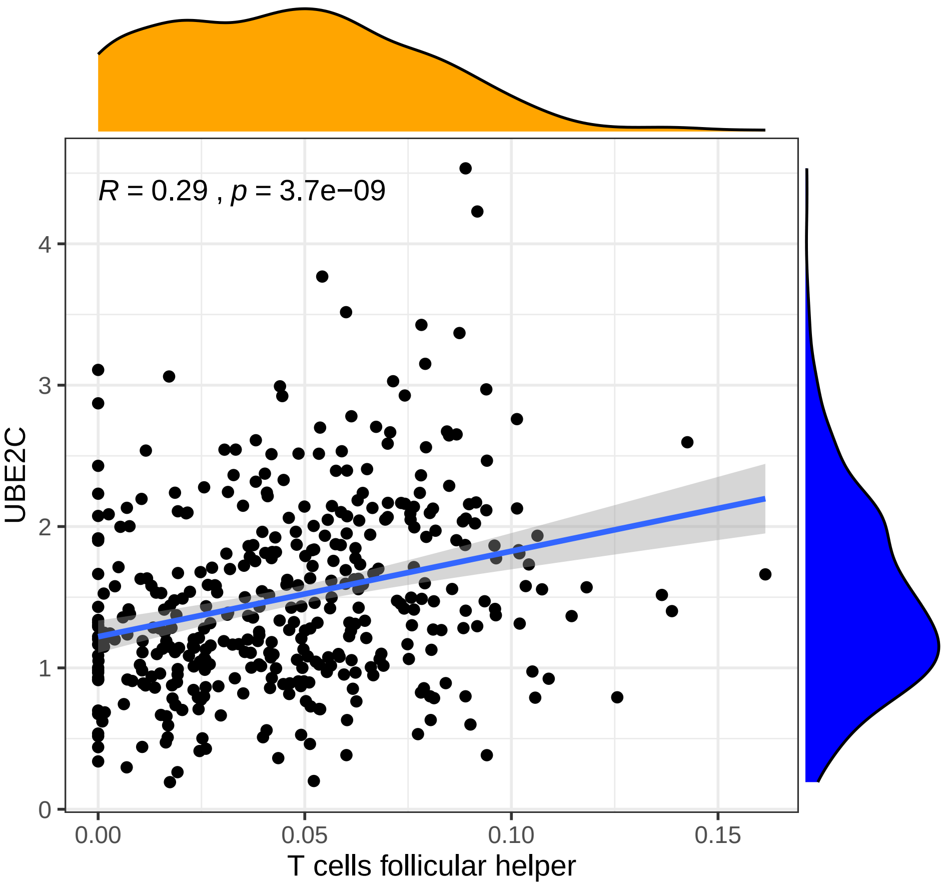

R = 0.29 , p = 3.7e−09
UBE2C
0
1
2
3
4
0.00
0.05
0.10
0.15
T cells follicular helper
