# Supplementary material for: Pan-Cancer Analyses of the Tumor Microenvironment Reveal That Ubiquitin-Conjugating Enzyme E2C Might Be a Potential Immunotherapy Target
Source: J Immunol Res. 2021 Dec 13;2021:9250207. doi: 10.1155/2021/9250207 (PMC8689232; doi:10.1155/2021/9250207)
Supplement: Supplementary 2 — Supplementary Table1: the p value of differentially expressed analysis in Figure 1(b). Supplementary Table2: the Spearman's correlation coefficient between UBE2C and immune markers. Supplementary Table3: the p value of correlation analysis between UBE2C and immune markers. Supplementary Table4: the p value between UBE2C expression and 22 immune cells correlation analysis. [file 9250207.f2.zip › Supplementary Table1.doc]

| Supplement Table1 p-value of differentially expressed analysis in Figure 1B | | | | | |
| --- | --- | --- | --- | --- | --- |
| CancerType | p-value | CancerType | p-value | CancerType | p-value |
| ACC | / | KIRC | <0.0001 | PRAD | <0.0001 |
| BLCA | <0.0001 | KIRP | <0.0001 | READ | <0.0001 |
| BRCA | <0.0001 | LAML | / | SARC | 0.0011 |
| CESC | <0.0001 | LGG | / | SKCM | / |
| CHOL | <0.0001 | LIHC | <0.0001 | STAD | <0.0001 |
| COAD | <0.0001 | LUAD | <0.0001 | TGCT | / |
| DLBC | / | LUSC | <0.0001 | THCA | <0.0001 |
| ESCA | <0.0001 | MESO | / | THYM | 0.9401 |
| GBM | <0.0001 | OV | / | UCEC | <0.0001 |
| HNSC | <0.0001 | PAAD | 0.0408 | UCS | / |
| KICH | <0.0001 | PCPG | 0.0372 | UVM | / |
