# Supplementary material for: Pan-Cancer Analyses of the Tumor Microenvironment Reveal That Ubiquitin-Conjugating Enzyme E2C Might Be a Potential Immunotherapy Target
Source: J Immunol Res. 2021 Dec 13;2021:9250207. doi: 10.1155/2021/9250207 (PMC8689232; doi:10.1155/2021/9250207)
Supplement: Supplementary 2 — Supplementary Table1: the p value of differentially expressed analysis in Figure 1(b). Supplementary Table2: the Spearman's correlation coefficient between UBE2C and immune markers. Supplementary Table3: the p value of correlation analysis between UBE2C and immune markers. Supplementary Table4: the p value between UBE2C expression and 22 immune cells correlation analysis. [file 9250207.f2.zip › Supplementary Table2.doc]

| **Supplementary Table2 The Spearman's correlation coefficient between UBE2C and immune markers** | | | | | | | | | | | |
| --- | --- | --- | --- | --- | --- | --- | --- | --- | --- | --- | --- |
| **Cancer Type** | **ACC** | **BLCA** | **BRCA** | **CESC** | **CHOL** | **COAD** | **DLBC** | **ESCA** | **GBM** | **HNSC** | **KICH** |
| **BTLA** | -0.0108 | -0.0650 | 0.0728 | -0.1099 | 0.0074 | -0.2767 | -0.1817 | -0.1000 | -0.2027 | -0.0325 | 0.2568 |
| **CD200** | 0.1134 | -0.1331 | -0.2419 | -0.1402 | -0.0422 | -0.2783 | -0.0744 | -0.0112 | 0.0585 | 0.0058 | -0.3014 |
| **TNFRSF14** | -0.0769 | -0.2783 | -0.1928 | -0.0666 | -0.0238 | -0.0168 | -0.2562 | 0.0368 | -0.3414 | 0.1450 | -0.0131 |
| **NRP1** | 0.2345 | 0.0323 | -0.3045 | -0.1631 | -0.1010 | -0.3288 | -0.0758 | -0.0058 | -0.1078 | -0.2331 | 0.0610 |
| **LAIR1** | -0.1165 | 0.0256 | 0.0487 | -0.1851 | -0.0025 | -0.3020 | -0.3160 | -0.0322 | -0.4602 | -0.0415 | 0.0275 |
| **TNFSF4** | 0.0239 | -0.0246 | -0.0452 | -0.1308 | 0.2163 | -0.2538 | -0.2129 | -0.0237 | -0.0920 | -0.0663 | -0.3081 |
| **CD244** | -0.1947 | -0.0937 | 0.0639 | -0.1511 | 0.0918 | -0.1819 | -0.1444 | -0.2125 | -0.2912 | -0.1114 | -0.2640 |
| **LAG3** | 0.2619 | 0.2464 | 0.3762 | 0.0323 | 0.4051 | -0.0876 | -0.1900 | 0.0858 | 0.1525 | 0.1856 | 0.2760 |
| **ICOS** | 0.0906 | 0.0854 | 0.2430 | -0.0807 | 0.0811 | -0.1570 | -0.4140 | -0.0433 | -0.1521 | -0.0053 | -0.1093 |
| **CD40LG** | -0.2088 | -0.1723 | 0.0022 | -0.1161 | -0.0956 | -0.1778 | -0.2165 | -0.2221 | -0.1295 | -0.0539 | -0.1347 |
| **CTLA4** | 0.0231 | 0.1090 | 0.2780 | -0.0818 | 0.1391 | -0.2487 | -0.4091 | -0.0189 | -0.0832 | 0.0622 | -0.0361 |
| **CD48** | -0.1054 | -0.0237 | 0.0480 | -0.1347 | -0.0325 | -0.1585 | -0.1082 | -0.1260 | -0.1301 | 0.0002 | -0.1576 |
| **CD28** | -0.0742 | -0.0995 | -0.0020 | -0.1326 | -0.0010 | -0.3037 | -0.4594 | -0.1236 | -0.1519 | -0.1547 | 0.2112 |
| **CD200R1** | -0.2282 | -0.1568 | -0.0897 | -0.1652 | 0.1057 | -0.2735 | -0.2213 | -0.1632 | -0.2889 | -0.0971 | 0.2835 |
| **HAVCR2** | -0.0206 | 0.1114 | 0.0418 | -0.1728 | 0.0230 | -0.2458 | -0.1327 | 0.0182 | -0.3785 | 0.0120 | -0.0989 |
| **ADORA2A** | 0.2409 | -0.0499 | -0.0745 | 0.0324 | -0.1684 | -0.1596 | 0.0253 | -0.0063 | 0.1332 | 0.0406 | -0.1977 |
| **CD276** | 0.3307 | 0.1621 | 0.1278 | -0.0045 | -0.0351 | -0.2041 | -0.1317 | 0.2940 | 0.2012 | 0.1289 | 0.2704 |
| **KIR3DL1** | -0.0491 | 0.0776 | 0.1016 | -0.0639 | -0.1112 | -0.1546 | -0.2074 | -0.0213 | -0.0914 | 0.0441 | -0.0077 |
| **CD80** | 0.1426 | 0.1314 | 0.1937 | -0.1738 | 0.1053 | -0.2305 | -0.0273 | -0.0064 | -0.1741 | 0.0228 | -0.1910 |
| **PDCD1** | 0.0714 | 0.1136 | 0.2135 | -0.0783 | 0.2134 | -0.1382 | -0.2532 | 0.0131 | -0.0347 | 0.0846 | 0.0049 |
| **LGALS9** | 0.0204 | -0.0936 | 0.1580 | -0.0812 | 0.0468 | -0.1063 | -0.3752 | -0.0091 | -0.3275 | 0.1390 | -0.0298 |
| **CD160** | -0.0303 | -0.0581 | -0.1389 | -0.0505 | -0.0271 | -0.3018 | -0.2695 | -0.0615 | -0.0052 | 0.0994 | -0.0931 |
| **TNFSF14** | -0.2972 | -0.0582 | 0.0882 | -0.2024 | -0.1319 | -0.4270 | -0.2563 | -0.0876 | -0.3081 | -0.1027 | 0.0110 |
| **IDO2** | -0.1549 | 0.0491 | 0.1232 | -0.0725 | -0.0158 | -0.1567 | -0.3505 | -0.0652 | 0.0710 | 0.1194 | 0.0178 |
| **ICOSLG** | -0.0205 | 0.0838 | 0.1277 | -0.0171 | 0.0751 | -0.0741 | -0.0326 | 0.0145 | -0.2223 | 0.0850 | 0.0088 |
| **TMIGD2** | -0.0918 | -0.0312 | 0.0965 | -0.1743 | -0.0523 | -0.1050 | -0.3232 | 0.0131 | -0.2752 | 0.0892 | -0.1102 |
| **VTCN1** | -0.1881 | -0.0230 | -0.2088 | -0.0233 | -0.0977 | -0.2069 | 0.0239 | -0.0673 | 0.3184 | -0.0238 | -0.2062 |
| **IDO1** | 0.0706 | 0.1406 | 0.3502 | -0.1185 | 0.1802 | -0.0594 | -0.1727 | -0.0228 | -0.0981 | 0.0983 | 0.1086 |
| **PDCD1LG2** | -0.0007 | 0.1540 | 0.1086 | -0.1526 | 0.2123 | -0.2833 | -0.1998 | -0.0144 | -0.3959 | -0.0374 | 0.1162 |
| **HHLA2** | -0.0957 | 0.0456 | 0.0366 | -0.0190 | 0.2128 | 0.1367 | -0.1112 | 0.1037 | -0.2471 | 0.0098 | -0.2389 |
| **TNFSF18** | -0.1841 | -0.0340 | 0.0061 | -0.1021 | 0.0596 | -0.1258 | 0.1116 | -0.0615 | 0.0286 | -0.0853 | -0.1752 |
| **BTNL2** | -0.0794 | -0.0450 | -0.0145 | -0.0467 | -0.1072 | -0.1102 | -0.1126 | 0.0521 | 0.2007 | -0.0011 | -0.0252 |
| **CD70** | 0.2458 | 0.1341 | 0.2060 | 0.1772 | 0.3535 | -0.1092 | 0.0589 | 0.1735 | -0.0298 | 0.2412 | 0.4620 |
| **TNFSF9** | 0.0652 | 0.0435 | 0.1891 | -0.0258 | 0.3846 | -0.2465 | 0.0669 | 0.0415 | -0.1516 | 0.0679 | 0.3976 |
| **TNFRSF8** | 0.1162 | 0.0516 | 0.1229 | -0.0924 | -0.2855 | -0.1889 | -0.0367 | -0.0161 | -0.2013 | 0.0275 | 0.0206 |
| **CD27** | -0.0600 | -0.0263 | 0.0812 | -0.1023 | 0.1416 | -0.1939 | -0.3070 | -0.1298 | -0.1954 | -0.0171 | 0.1640 |
| **TNFRSF25** | 0.1440 | 0.0249 | 0.0764 | 0.0490 | 0.2313 | 0.1344 | -0.4109 | 0.0495 | -0.0658 | 0.2059 | 0.0057 |
| **VSIR** | -0.2433 | -0.2643 | -0.1559 | -0.2289 | -0.1073 | -0.3291 | -0.3623 | -0.2250 | -0.5047 | -0.0792 | -0.0268 |
| **TNFRSF4** | 0.1897 | 0.0681 | 0.1576 | -0.1033 | -0.0771 | -0.0167 | -0.3238 | 0.0326 | 0.1130 | 0.0836 | 0.1152 |
| **CD40** | -0.1615 | -0.1818 | 0.0788 | -0.0136 | -0.0696 | 0.0389 | 0.1464 | 0.1672 | -0.2000 | 0.2663 | 0.2029 |
| **TNFRSF18** | 0.1034 | 0.1580 | 0.0885 | -0.0799 | -0.0296 | -0.0469 | -0.2089 | 0.0571 | 0.0068 | 0.1825 | 0.0647 |
| **TNFSF15** | 0.1045 | -0.2389 | 0.0479 | -0.1147 | -0.0701 | -0.3338 | -0.4055 | 0.0273 | -0.1103 | 0.0822 | -0.2532 |
| **TIGIT** | 0.0708 | 0.0868 | 0.2063 | -0.1261 | 0.1203 | -0.2702 | -0.4710 | -0.0753 | -0.2402 | -0.0281 | -0.0416 |
| **CD274** | 0.0467 | 0.2106 | 0.0943 | -0.2008 | 0.1160 | -0.2430 | -0.1362 | -0.1241 | -0.2850 | -0.0264 | -0.0966 |
| **CD86** | -0.0844 | 0.0798 | 0.1007 | -0.1816 | -0.0083 | -0.2565 | -0.2229 | -0.0027 | -0.4107 | -0.0046 | -0.1224 |
| **CD44** | 0.0571 | 0.0041 | -0.2467 | -0.1749 | -0.1142 | -0.2323 | -0.1455 | -0.1553 | -0.3235 | -0.1307 | 0.0918 |
| **TNFRSF9** | 0.1982 | 0.1004 | 0.2036 | -0.0588 | 0.0485 | -0.2646 | -0.3659 | 0.0107 | -0.0543 | -0.0233 | 0.0981 |
| **Cancer Type** | **KIRC** | **KIRP** | **LAML** | **LGG** | **LIHC** | **LUAD** | **LUSC** | **MESO** | **OV** | **PAAD** | **PCPG** |
| **BTLA** | 0.2032 | 0.2105 | 0.1378 | -0.0145 | 0.1443 | -0.1535 | -0.1977 | 0.0692 | -0.0868 | -0.1694 | -0.1907 |
| **CD200** | -0.2159 | -0.2157 | 0.0101 | -0.2350 | 0.0717 | -0.0128 | -0.1816 | -0.3656 | -0.1292 | -0.4918 | -0.2192 |
| **TNFRSF14** | -0.0935 | -0.1742 | 0.0419 | 0.1613 | 0.2668 | -0.1294 | -0.1495 | -0.0787 | -0.2328 | 0.3498 | -0.0061 |
| **NRP1** | -0.2763 | -0.1559 | 0.1542 | 0.3172 | 0.0744 | -0.0598 | -0.2921 | 0.2721 | -0.1209 | -0.2583 | -0.0010 |
| **LAIR1** | 0.2861 | -0.0806 | 0.1213 | 0.1102 | 0.2791 | 0.0086 | -0.2548 | 0.1722 | -0.1255 | -0.0865 | 0.0189 |
| **TNFSF4** | 0.3571 | 0.1258 | -0.1166 | -0.1072 | 0.3624 | 0.2168 | -0.1624 | 0.3836 | -0.1345 | 0.0022 | -0.2325 |
| **CD244** | 0.2022 | -0.0638 | -0.1494 | -0.0301 | 0.1466 | 0.0109 | -0.0677 | -0.0891 | -0.1639 | -0.1064 | -0.1327 |
| **LAG3** | 0.4558 | 0.3482 | -0.2368 | 0.3004 | 0.3385 | 0.3184 | 0.0940 | 0.0222 | 0.1045 | 0.1183 | -0.0516 |
| **ICOS** | 0.2513 | 0.1713 | 0.0143 | 0.0978 | 0.2593 | 0.0408 | -0.1601 | 0.1090 | -0.0261 | -0.1151 | -0.0048 |
| **CD40LG** | 0.0900 | -0.0442 | 0.0360 | 0.1064 | 0.0183 | -0.3588 | -0.2647 | -0.2214 | -0.1293 | -0.2248 | -0.1726 |
| **CTLA4** | 0.3331 | 0.2067 | -0.0239 | 0.0788 | 0.3309 | 0.1199 | -0.0923 | 0.1180 | 0.0017 | -0.0389 | -0.0357 |
| **CD48** | 0.1574 | -0.0815 | 0.0234 | 0.1713 | 0.1691 | -0.0729 | -0.1406 | 0.0064 | -0.0087 | -0.1901 | -0.1053 |
| **CD28** | 0.2103 | -0.0324 | -0.0572 | 0.0834 | 0.1193 | -0.1779 | -0.3320 | -0.0805 | -0.1259 | -0.2573 | 0.0717 |
| **CD200R1** | 0.1327 | 0.0664 | 0.0449 | 0.0139 | 0.1183 | -0.1985 | -0.1662 | 0.1364 | -0.0680 | -0.2238 | 0.0259 |
| **HAVCR2** | -0.0664 | -0.2077 | -0.2465 | 0.1228 | 0.2458 | 0.0756 | -0.2033 | 0.1067 | -0.1070 | -0.0224 | -0.0738 |
| **ADORA2A** | -0.1158 | 0.0727 | -0.0995 | 0.1775 | 0.1424 | -0.0723 | -0.1309 | 0.0526 | -0.0418 | -0.3164 | 0.1373 |
| **CD276** | 0.3369 | 0.0954 | 0.0537 | 0.6026 | 0.5214 | 0.2901 | -0.0093 | 0.4715 | -0.0030 | 0.3922 | 0.1203 |
| **KIR3DL1** | -0.1241 | 0.0617 | -0.0916 | 0.0291 | 0.0148 | 0.1096 | -0.0349 | -0.2273 | -0.0616 | -0.0947 | -0.1045 |
| **CD80** | 0.2842 | 0.1684 | -0.1552 | 0.1475 | 0.1983 | -0.0269 | -0.1487 | 0.1874 | -0.0364 | 0.0694 | 0.0143 |
| **PDCD1** | 0.3825 | 0.2897 | -0.2198 | 0.1087 | 0.3542 | 0.2648 | -0.0567 | 0.2429 | -0.0079 | -0.0314 | 0.0874 |
| **LGALS9** | 0.3440 | 0.1007 | -0.0901 | 0.1725 | 0.3022 | -0.0963 | -0.1207 | 0.1464 | -0.0923 | 0.5136 | 0.0801 |
| **CD160** | 0.0190 | 0.0624 | 0.0002 | 0.1708 | 0.0088 | -0.1397 | -0.0844 | 0.0138 | -0.1072 | -0.2491 | -0.0368 |
| **TNFSF14** | 0.2913 | 0.1531 | -0.0319 | 0.0956 | -0.0057 | -0.2111 | -0.2707 | -0.1791 | -0.1898 | -0.0664 | -0.1060 |
| **IDO2** | 0.1279 | 0.1991 | 0.1308 | 0.0360 | -0.1767 | -0.0409 | -0.0481 | -0.0870 | 0.0531 | -0.1023 | -0.0605 |
| **ICOSLG** | 0.0198 | 0.0610 | 0.0109 | 0.0222 | 0.0598 | -0.0098 | -0.0523 | -0.0113 | -0.0088 | -0.1720 | 0.1053 |
| **TMIGD2** | 0.2893 | 0.0805 | 0.0893 | 0.0600 | -0.0512 | 0.1106 | -0.0564 | -0.1269 | -0.1011 | -0.0603 | 0.0748 |
| **VTCN1** | -0.0648 | -0.1382 | -0.0025 | 0.3147 | 0.1037 | 0.0032 | -0.0789 | 0.1578 | -0.0055 | -0.0113 | 0.0480 |
| **IDO1** | -0.1009 | 0.3673 | 0.0731 | 0.1807 | 0.1282 | 0.2458 | 0.0527 | 0.2204 | -0.0383 | 0.0761 | 0.0315 |
| **PDCD1LG2** | 0.1496 | 0.1198 | -0.1530 | 0.1244 | 0.0919 | 0.1456 | -0.1548 | 0.2041 | -0.0970 | -0.0311 | -0.0739 |
| **HHLA2** | -0.2198 | -0.1834 | 0.0539 | -0.3160 | 0.1023 | -0.1133 | -0.1888 | -0.2644 | -0.1583 | 0.1223 | -0.1199 |
| **TNFSF18** | -0.0786 | 0.0210 | -0.0684 | -0.0805 | 0.1049 | -0.0470 | -0.0814 | 0.2073 | -0.0446 | -0.2209 | -0.2158 |
| **BTNL2** | -0.0211 | 0.0635 | 0.2066 | -0.0646 | 0.1018 | -0.0147 | 0.0001 | 0.0589 | 0.0074 | -0.0699 | -0.0647 |
| **CD70** | 0.2292 | 0.1315 | 0.1017 | 0.1372 | 0.2696 | 0.2536 | 0.1122 | 0.3628 | -0.0226 | 0.3435 | 0.1069 |
| **TNFSF9** | 0.2499 | 0.2179 | 0.1492 | -0.1180 | 0.2665 | 0.0302 | 0.1173 | 0.2996 | -0.0516 | 0.4335 | 0.0483 |
| **TNFRSF8** | 0.3671 | 0.2504 | -0.2746 | 0.0456 | 0.2894 | 0.1350 | -0.1052 | -0.0728 | 0.0587 | -0.1075 | -0.0332 |
| **CD27** | 0.3310 | 0.2158 | 0.0910 | 0.1114 | 0.2543 | -0.0463 | -0.2215 | 0.1139 | -0.0526 | -0.0947 | -0.1229 |
| **TNFRSF25** | 0.1308 | 0.1741 | -0.1672 | 0.0065 | 0.2651 | 0.2107 | 0.1029 | 0.3282 | -0.0546 | 0.2714 | 0.0310 |
| **VSIR** | -0.1113 | -0.0999 | -0.1827 | -0.1898 | 0.1274 | -0.1416 | -0.3601 | -0.5086 | -0.3431 | 0.2219 | -0.1173 |
| **TNFRSF4** | -0.0152 | 0.3841 | -0.1878 | 0.2824 | 0.3749 | 0.1851 | -0.1201 | 0.2322 | 0.1007 | 0.1022 | 0.1765 |
| **CD40** | 0.1036 | 0.0614 | 0.0042 | 0.1436 | 0.0654 | 0.0700 | 0.0166 | 0.0586 | 0.0543 | 0.1650 | -0.1940 |
| **TNFRSF18** | 0.4572 | 0.2221 | -0.0703 | 0.0614 | 0.4061 | 0.3098 | 0.0901 | 0.1495 | 0.0057 | 0.3186 | 0.0077 |
| **TNFSF15** | -0.0953 | 0.0702 | 0.0145 | 0.0683 | 0.2676 | -0.4101 | -0.1023 | -0.1163 | -0.2584 | 0.1598 | 0.0883 |
| **TIGIT** | 0.3734 | 0.2988 | -0.0605 | -0.1534 | 0.2674 | 0.1009 | -0.1133 | 0.1619 | -0.0412 | -0.1247 | -0.1145 |
| **CD274** | 0.0011 | -0.0930 | -0.0645 | -0.0021 | 0.1482 | 0.2680 | -0.0704 | 0.1114 | -0.0807 | 0.1119 | -0.3779 |
| **CD86** | 0.1848 | -0.0816 | -0.0812 | 0.0409 | 0.2531 | 0.0770 | -0.2367 | 0.0891 | -0.0994 | -0.0506 | -0.0430 |
| **CD44** | 0.4187 | 0.1719 | -0.2636 | 0.0420 | 0.0514 | -0.1797 | -0.2042 | 0.0834 | -0.1231 | 0.2629 | -0.1785 |
| **TNFRSF9** | 0.3541 | 0.2566 | 0.0871 | -0.0009 | 0.1288 | 0.3033 | -0.0768 | 0.2825 | -0.0017 | 0.0730 | 0.0841 |
| **Cancer Type** | **PRAD** | **READ** | **SARC** | **SKCM** | **STAD** | **TGCT** | **THCA** | **THYM** | **UCEC** | **UCS** | **UVM** |
| **BTLA** | 0.1865 | -0.2884 | -0.0526 | -0.1221 | -0.2604 | 0.3811 | 0.4985 | 0.0585 | 0.0006 | -0.2309 | -0.0451 |
| **CD200** | -0.1317 | -0.2588 | -0.1607 | -0.1388 | -0.2651 | 0.4561 | 0.0413 | 0.3898 | -0.0903 | -0.1934 | -0.3069 |
| **TNFRSF14** | -0.0918 | 0.0712 | -0.1117 | 0.0261 | 0.1045 | -0.3718 | 0.3159 | 0.1774 | -0.1840 | -0.2309 | -0.1544 |
| **NRP1** | 0.1909 | -0.3472 | -0.0307 | -0.0169 | -0.2586 | -0.3990 | -0.0190 | -0.6338 | -0.1410 | -0.1609 | 0.0101 |
| **LAIR1** | 0.1322 | -0.3340 | 0.0105 | -0.0839 | -0.1617 | -0.1880 | 0.4786 | 0.8188 | -0.1118 | -0.2254 | -0.0608 |
| **TNFSF4** | -0.0176 | -0.2430 | 0.0533 | -0.0289 | 0.0116 | -0.0179 | 0.4135 | 0.3777 | 0.0391 | -0.1080 | -0.1057 |
| **CD244** | -0.0038 | -0.2597 | -0.0213 | -0.1531 | -0.0320 | -0.2025 | 0.4404 | 0.4978 | -0.0462 | -0.1205 | -0.1499 |
| **LAG3** | 0.0735 | -0.1388 | 0.1099 | 0.0229 | 0.1237 | -0.0126 | 0.5009 | -0.2260 | 0.2429 | 0.0624 | 0.0554 |
| **ICOS** | 0.1400 | -0.1622 | 0.0519 | -0.0932 | -0.0249 | -0.1233 | 0.5323 | 0.6982 | 0.0293 | -0.2371 | -0.0581 |
| **CD40LG** | 0.0428 | -0.2138 | -0.2207 | -0.1302 | -0.2361 | -0.3341 | 0.5012 | 0.2400 | -0.2405 | -0.2124 | -0.0416 |
| **CTLA4** | 0.1593 | -0.1968 | 0.0884 | 0.0127 | 0.0878 | -0.1410 | 0.5605 | -0.2757 | -0.1325 | -0.2196 | -0.0905 |
| **CD48** | 0.0698 | -0.2887 | -0.0963 | -0.1196 | -0.2277 | -0.2228 | 0.5065 | 0.5944 | -0.0479 | 0.0200 | -0.1581 |
| **CD28** | 0.0481 | -0.3735 | -0.0428 | -0.1658 | -0.2982 | -0.3238 | 0.5292 | 0.6323 | -0.0938 | -0.2522 | -0.0186 |
| **CD200R1** | 0.0920 | -0.2647 | 0.0139 | -0.1883 | -0.1954 | -0.3004 | 0.4815 | -0.2435 | -0.0426 | 0.0220 | -0.1641 |
| **HAVCR2** | 0.2342 | -0.2555 | 0.0741 | -0.0514 | -0.0493 | -0.0943 | 0.4912 | -0.3403 | -0.0124 | -0.3384 | -0.0666 |
| **ADORA2A** | 0.1017 | -0.1192 | 0.0890 | -0.0733 | 0.0395 | 0.2924 | 0.3495 | 0.6094 | -0.0599 | 0.0023 | -0.1792 |
| **CD276** | 0.2183 | -0.1413 | 0.2129 | 0.0700 | 0.1127 | -0.2438 | 0.3729 | -0.5993 | -0.1599 | -0.0015 | 0.3974 |
| **KIR3DL1** | 0.0925 | -0.1210 | -0.1328 | 0.0324 | -0.0427 | -0.0126 | 0.2825 | -0.2346 | 0.0258 | -0.1220 | -0.0870 |
| **CD80** | 0.2285 | -0.1960 | 0.1329 | -0.0645 | 0.0496 | -0.1487 | 0.4820 | -0.2889 | 0.0581 | -0.2699 | -0.0007 |
| **PDCD1** | 0.1576 | -0.1904 | 0.0516 | -0.0489 | 0.0126 | -0.0955 | 0.4387 | 0.7630 | -0.0029 | -0.1903 | -0.0294 |
| **LGALS9** | 0.1422 | -0.0666 | -0.0067 | -0.0500 | 0.0389 | -0.2030 | 0.5566 | 0.4543 | -0.1564 | -0.2985 | 0.0498 |
| **CD160** | -0.1158 | -0.1442 | -0.0509 | -0.1488 | 0.0284 | -0.2256 | 0.1809 | -0.3541 | -0.0804 | -0.1831 | 0.0045 |
| **TNFSF14** | 0.0227 | -0.4094 | -0.1146 | -0.1363 | -0.1756 | -0.3581 | 0.4334 | -0.2454 | -0.3597 | -0.3239 | -0.0335 |
| **IDO2** | -0.0089 | -0.2194 | -0.0878 | -0.0789 | -0.1208 | -0.0905 | 0.1598 | 0.0353 | 0.0530 | -0.1936 | 0.0499 |
| **ICOSLG** | 0.1219 | -0.1022 | 0.0534 | -0.0335 | -0.0937 | 0.3682 | 0.1340 | -0.2350 | 0.2131 | -0.1114 | 0.1087 |
| **TMIGD2** | -0.0179 | -0.1011 | -0.1045 | -0.0263 | 0.0208 | -0.2059 | 0.4624 | 0.8343 | 0.0039 | 0.1150 | -0.1036 |
| **VTCN1** | -0.1737 | -0.2712 | 0.0560 | -0.1001 | -0.1140 | -0.2046 | 0.2480 | -0.0750 | -0.0539 | -0.4146 | -0.1847 |
| **IDO1** | 0.1367 | 0.0003 | 0.0470 | -0.0277 | 0.1422 | 0.0188 | 0.4535 | -0.0478 | -0.0179 | -0.4097 | -0.0401 |
| **PDCD1LG2** | -0.0152 | -0.2853 | 0.0320 | -0.0715 | -0.1370 | -0.2534 | 0.3902 | -0.0310 | 0.0699 | -0.3428 | -0.2057 |
| **HHLA2** | 0.1834 | 0.1714 | -0.0689 | -0.0358 | 0.0875 | -0.2726 | 0.0322 | 0.2561 | -0.1725 | 0.0952 | 0.0000 |
| **TNFSF18** | 0.2042 | -0.2600 | -0.1188 | -0.1617 | 0.0036 | -0.0326 | 0.2929 | -0.2342 | -0.0233 | -0.2188 | 0.2205 |
| **BTNL2** | 0.0198 | 0.0583 | -0.1855 | -0.1341 | -0.0622 | -0.2594 | 0.0518 | -0.2995 | 0.0875 | 0.2835 | -0.2058 |
| **CD70** | 0.2404 | -0.0918 | 0.2718 | 0.0513 | 0.0914 | 0.6447 | 0.3880 | -0.1402 | 0.0337 | 0.2266 | 0.0660 |
| **TNFSF9** | 0.0459 | -0.1650 | 0.2584 | 0.0085 | 0.0197 | 0.7176 | 0.3443 | -0.3619 | 0.1348 | 0.2501 | 0.2873 |
| **TNFRSF8** | 0.2006 | -0.2316 | -0.0285 | -0.0788 | -0.0295 | 0.6717 | 0.3540 | 0.0298 | 0.0563 | 0.2426 | 0.1888 |
| **CD27** | 0.1404 | -0.2536 | 0.0112 | -0.0934 | -0.1492 | -0.2067 | 0.1987 | 0.4115 | -0.1227 | -0.2261 | -0.0153 |
| **TNFRSF25** | 0.0640 | 0.2318 | -0.0876 | -0.0563 | 0.3137 | -0.2558 | 0.4313 | -0.1635 | 0.0820 | 0.1784 | -0.1026 |
| **VSIR** | -0.2024 | -0.3293 | -0.1499 | -0.1777 | -0.3481 | -0.4579 | 0.0244 | -0.3658 | -0.1694 | -0.4425 | 0.0259 |
| **TNFRSF4** | 0.2118 | -0.0724 | -0.0195 | 0.0394 | 0.1537 | -0.1794 | 0.4473 | -0.0844 | -0.0456 | 0.2051 | 0.1060 |
| **CD40** | -0.2323 | 0.0300 | 0.0875 | 0.0068 | 0.0646 | -0.2416 | 0.2576 | -0.5465 | 0.2761 | 0.1382 | -0.4106 |
| **TNFRSF18** | 0.3450 | -0.1091 | 0.0578 | -0.0034 | 0.2130 | -0.0218 | 0.4977 | -0.1333 | 0.0174 | -0.1360 | 0.0893 |
| **TNFSF15** | -0.0592 | -0.1040 | -0.0863 | 0.0109 | -0.0171 | -0.3970 | 0.0933 | -0.2097 | -0.2326 | -0.2165 | -0.0283 |
| **TIGIT** | 0.1769 | -0.2780 | 0.0408 | -0.0855 | -0.0799 | -0.1058 | 0.5461 | -0.3139 | 0.0397 | -0.2060 | -0.0419 |
| **CD274** | -0.0386 | -0.1794 | 0.0623 | -0.0682 | 0.0558 | -0.1912 | 0.2187 | -0.2094 | 0.0292 | -0.3742 | -0.2131 |
| **CD86** | 0.1672 | -0.2707 | 0.0519 | -0.0863 | -0.0556 | -0.1466 | 0.5236 | -0.4763 | 0.0078 | -0.2371 | -0.0607 |
| **CD44** | -0.3155 | -0.1123 | 0.1327 | -0.3052 | -0.2294 | -0.3534 | 0.0532 | -0.0416 | -0.2618 | -0.4245 | -0.3698 |
| **TNFRSF9** | 0.1951 | -0.1957 | 0.1382 | -0.0625 | -0.0375 | -0.0652 | 0.5510 | -0.2752 | -0.0037 | -0.1377 | -0.0077 |
