# Supplementary material for: Pan-Cancer Analyses of the Tumor Microenvironment Reveal That Ubiquitin-Conjugating Enzyme E2C Might Be a Potential Immunotherapy Target
Source: J Immunol Res. 2021 Dec 13;2021:9250207. doi: 10.1155/2021/9250207 (PMC8689232; doi:10.1155/2021/9250207)
Supplement: Supplementary 2 — Supplementary Table1: the p value of differentially expressed analysis in Figure 1(b). Supplementary Table2: the Spearman's correlation coefficient between UBE2C and immune markers. Supplementary Table3: the p value of correlation analysis between UBE2C and immune markers. Supplementary Table4: the p value between UBE2C expression and 22 immune cells correlation analysis. [file 9250207.f2.zip › Supplementary Table3.doc]

| **Supplementary Table3 The p-value of correlation analysis between UBE2C and immune markers** | | | | | | | | | | | |
| --- | --- | --- | --- | --- | --- | --- | --- | --- | --- | --- | --- |
| **Cancer Type** | **ACC** | **BLCA** | **BRCA** | **CESC** | **CHOL** | **COAD** | **DLBC** | **ESCA** | **GBM** | **HNSC** | **KICH** |
| **BTLA** | 0.9245 | 0.1883 | 0.0155 | 0.0547 | 0.9656 | <0.0001 | 0.2165 | 0.2054 | 0.0084 | 0.4675 | 0.0389 |
| **CD200** | 0.3196 | 0.0069 | <0.0001 | 0.0141 | 0.8070 | <0.0001 | 0.6153 | 0.8873 | 0.4516 | 0.8963 | 0.0147 |
| **TNFRSF14** | 0.5003 | <0.0001 | <0.0001 | 0.2458 | 0.8906 | 0.7167 | 0.0788 | 0.6422 | <0.0001 | 0.0011 | 0.9176 |
| **NRP1** | 0.0375 | 0.5136 | <0.0001 | 0.0042 | 0.5579 | <0.0001 | 0.6085 | 0.9418 | 0.1643 | <0.0001 | 0.6292 |
| **LAIR1** | 0.3065 | 0.6048 | 0.1059 | 0.0011 | 0.9883 | <0.0001 | 0.0286 | 0.6838 | <0.0001 | 0.3530 | 0.8277 |
| **TNFSF4** | 0.8343 | 0.6194 | 0.1336 | 0.0221 | 0.2051 | <0.0001 | 0.1463 | 0.7646 | 0.2355 | 0.1379 | 0.0125 |
| **CD244** | 0.0855 | 0.0577 | 0.0339 | 0.0081 | 0.5942 | 0.0001 | 0.3274 | 0.0066 | 0.0001 | 0.0125 | 0.0336 |
| **LAG3** | 0.0197 | <0.0001 | <0.0001 | 0.5736 | 0.0143 | 0.0574 | 0.1958 | 0.2777 | 0.0485 | <0.0001 | 0.0261 |
| **ICOS** | 0.4271 | 0.0837 | <0.0001 | 0.1593 | 0.6383 | 0.0006 | 0.0034 | 0.5840 | 0.0490 | 0.9060 | 0.3860 |
| **CD40LG** | 0.0648 | 0.0005 | 0.9418 | 0.0424 | 0.5791 | 0.0001 | 0.1393 | 0.0045 | 0.0942 | 0.2280 | 0.2846 |
| **CTLA4** | 0.8399 | 0.0271 | <0.0001 | 0.1533 | 0.4184 | <0.0001 | 0.0039 | 0.8109 | 0.2839 | 0.1638 | 0.7754 |
| **CD48** | 0.3551 | 0.6316 | 0.1110 | 0.0184 | 0.8507 | 0.0006 | 0.4640 | 0.1101 | 0.0929 | 0.9966 | 0.2098 |
| **CD28** | 0.5157 | 0.0438 | 0.9465 | 0.0203 | 0.9953 | <0.0001 | 0.0010 | 0.1171 | 0.0494 | 0.0005 | 0.0913 |
| **CD200R1** | 0.0431 | 0.0014 | 0.0028 | 0.0038 | 0.5396 | <0.0001 | 0.1306 | 0.0380 | 0.0001 | 0.0297 | 0.0221 |
| **HAVCR2** | 0.8571 | 0.0239 | 0.1656 | 0.0024 | 0.8940 | <0.0001 | 0.3687 | 0.8185 | <0.0001 | 0.7884 | 0.4332 |
| **ADORA2A** | 0.0325 | 0.3132 | 0.0133 | 0.5723 | 0.3262 | 0.0005 | 0.8642 | 0.9370 | 0.0853 | 0.3637 | 0.1144 |
| **CD276** | 0.0029 | 0.0010 | <0.0001 | 0.9379 | 0.8387 | <0.0001 | 0.3723 | 0.0001 | 0.0089 | 0.0038 | 0.0294 |
| **KIR3DL1** | 0.6676 | 0.1164 | 0.0007 | 0.2650 | 0.5183 | 0.0008 | 0.1571 | 0.7876 | 0.2389 | 0.3246 | 0.9512 |
| **CD80** | 0.2099 | 0.0076 | <0.0001 | 0.0023 | 0.5409 | <0.0001 | 0.8541 | 0.9353 | 0.0240 | 0.6096 | 0.1275 |
| **PDCD1** | 0.5318 | 0.0212 | <0.0001 | 0.1717 | 0.2114 | 0.0026 | 0.0825 | 0.8682 | 0.6548 | 0.0582 | 0.9688 |
| **LGALS9** | 0.8583 | 0.0581 | <0.0001 | 0.1565 | 0.7864 | 0.0210 | 0.0086 | 0.9083 | <0.0001 | 0.0018 | 0.8138 |
| **CD160** | 0.7907 | 0.2398 | <0.0001 | 0.3782 | 0.8751 | <0.0001 | 0.0639 | 0.4369 | 0.9466 | 0.0259 | 0.4606 |
| **TNFSF14** | 0.0078 | 0.2389 | 0.0034 | 0.0004 | 0.4433 | <0.0001 | 0.0787 | 0.2678 | <0.0001 | 0.0214 | 0.9310 |
| **IDO2** | 0.1729 | 0.3208 | <0.0001 | 0.2061 | 0.9270 | 0.0006 | 0.0146 | 0.4096 | 0.3604 | 0.0074 | 0.8882 |
| **ICOSLG** | 0.8574 | 0.0898 | <0.0001 | 0.7654 | 0.6635 | 0.1084 | 0.8260 | 0.8551 | 0.0038 | 0.0570 | 0.9443 |
| **TMIGD2** | 0.4209 | 0.5285 | 0.0013 | 0.0022 | 0.7621 | 0.0227 | 0.0250 | 0.8690 | 0.0003 | 0.0457 | 0.3823 |
| **VTCN1** | 0.0970 | 0.6427 | <0.0001 | 0.6849 | 0.5709 | <0.0001 | 0.8717 | 0.3948 | <0.0001 | 0.5952 | 0.0993 |
| **IDO1** | 0.5362 | 0.0043 | <0.0001 | 0.0382 | 0.2930 | 0.1979 | 0.2404 | 0.7736 | 0.2060 | 0.0277 | 0.3891 |
| **PDCD1LG2** | 0.9949 | 0.0017 | 0.0003 | 0.0075 | 0.2138 | <0.0001 | 0.1734 | 0.8555 | <0.0001 | 0.4034 | 0.3568 |
| **HHLA2** | 0.4015 | 0.3566 | 0.2242 | 0.7408 | 0.2127 | 0.0030 | 0.4519 | 0.1890 | 0.0012 | 0.8264 | 0.0553 |
| **TNFSF18** | 0.1044 | 0.4917 | 0.8394 | 0.0746 | 0.7298 | 0.0062 | 0.4500 | 0.4365 | 0.7131 | 0.0561 | 0.1627 |
| **BTNL2** | 0.4865 | 0.3631 | 0.6304 | 0.4161 | 0.5336 | 0.0167 | 0.4459 | 0.5101 | 0.0091 | 0.9799 | 0.8423 |
| **CD70** | 0.0290 | 0.0065 | <0.0001 | 0.0019 | 0.0344 | 0.0177 | 0.6909 | 0.0273 | 0.7018 | <0.0001 | 0.0001 |
| **TNFSF9** | 0.5679 | 0.3793 | <0.0001 | 0.6531 | 0.0206 | <0.0001 | 0.6514 | 0.5997 | 0.0498 | 0.1285 | 0.0010 |
| **TNFRSF8** | 0.3078 | 0.2969 | <0.0001 | 0.1068 | 0.0914 | <0.0001 | 0.8046 | 0.8391 | 0.0089 | 0.5384 | 0.8707 |
| **CD27** | 0.5994 | 0.5950 | 0.0070 | 0.0739 | 0.4101 | <0.0001 | 0.0338 | 0.0996 | 0.0111 | 0.7018 | 0.1916 |
| **TNFRSF25** | 0.2055 | 0.6144 | 0.0112 | 0.3935 | 0.1747 | 0.0035 | 0.0037 | 0.5320 | 0.3970 | <0.0001 | 0.9641 |
| **VSIR** | 0.0307 | <0.0001 | <0.0001 | 0.0001 | 0.5335 | <0.0001 | 0.0114 | 0.0040 | <0.0001 | 0.0763 | 0.8322 |
| **TNFRSF4** | 0.0941 | 0.1680 | <0.0001 | 0.0710 | 0.6550 | 0.7172 | 0.0248 | 0.6803 | 0.1446 | 0.0613 | 0.3609 |
| **CD40** | 0.1550 | 0.0002 | 0.0088 | 0.8131 | 0.6868 | 0.3993 | 0.3206 | 0.0335 | 0.0093 | <0.0001 | 0.1049 |
| **TNFRSF18** | 0.3647 | 0.0013 | 0.0033 | 0.1634 | 0.8640 | 0.3095 | 0.1542 | 0.4704 | 0.9302 | <0.0001 | 0.6088 |
| **TNFSF15** | 0.3596 | <0.0001 | 0.1117 | 0.0449 | 0.6846 | <0.0001 | 0.0042 | 0.7298 | 0.1547 | 0.0658 | 0.0418 |
| **TIGIT** | 0.5351 | 0.0789 | <0.0001 | 0.0274 | 0.4848 | <0.0001 | 0.0007 | 0.3409 | 0.0017 | 0.5295 | 0.7419 |
| **CD274** | 0.6829 | <0.0001 | 0.0017 | 0.0004 | 0.5006 | <0.0001 | 0.3561 | 0.1157 | 0.0002 | 0.5550 | 0.4439 |
| **CD86** | 0.4594 | 0.1063 | 0.0008 | 0.0014 | 0.9616 | <0.0001 | 0.1279 | 0.9729 | <0.0001 | 0.9181 | 0.3312 |
| **CD44** | 0.6173 | 0.9344 | <0.0001 | 0.0021 | 0.5073 | <0.0001 | 0.3237 | 0.0485 | <0.0001 | 0.0033 | 0.4671 |
| **TNFRSF9** | 0.0799 | 0.0419 | <0.0001 | 0.3051 | 0.7787 | <0.0001 | 0.0106 | 0.8927 | 0.4843 | 0.6031 | 0.4367 |
| **Cancer Type** | **KIRC** | **KIRP** | **LAML** | **LGG** | **LIHC** | **LUAD** | **LUSC** | **MESO** | **OV** | **PAAD** | **PCPG** |
| **BTLA** | <0.0001 | 0.0003 | 0.0916 | 0.7391 | 0.0052 | 0.0004 | <0.0001 | 0.5267 | 0.0915 | 0.0238 | 0.0097 |
| **CD200** | <0.0001 | 0.0002 | 0.9025 | <0.0001 | 0.1667 | 0.7705 | <0.0001 | 0.0005 | 0.0118 | <0.0001 | 0.0029 |
| **TNFRSF14** | 0.0307 | 0.0030 | 0.6096 | 0.0002 | <0.0001 | 0.0029 | 0.0008 | 0.4712 | <0.0001 | <0.0001 | 0.9343 |
| **NRP1** | <0.0001 | 0.0079 | 0.0587 | <0.0001 | 0.1513 | 0.1711 | <0.0001 | 0.0113 | 0.0186 | 0.0005 | 0.9897 |
| **LAIR1** | <0.0001 | 0.1716 | 0.1378 | 0.0112 | <0.0001 | 0.8444 | <0.0001 | 0.1130 | 0.0145 | 0.2507 | 0.7992 |
| **TNFSF4** | <0.0001 | 0.0326 | 0.1538 | 0.0136 | <0.0001 | <0.0001 | 0.0003 | 0.0003 | 0.0088 | 0.9769 | 0.0015 |
| **CD244** | <0.0001 | 0.2798 | 0.0672 | 0.4900 | 0.0045 | 0.8033 | 0.1303 | 0.4146 | 0.0014 | 0.1575 | 0.0733 |
| **LAG3** | <0.0001 | <0.0001 | 0.0034 | <0.0001 | <0.0001 | <0.0001 | 0.0355 | 0.8392 | 0.0420 | 0.1157 | 0.4878 |
| **ICOS** | <0.0001 | 0.0035 | 0.8621 | 0.0245 | <0.0001 | 0.3506 | 0.0003 | 0.3177 | 0.6125 | 0.1259 | 0.9484 |
| **CD40LG** | 0.0374 | 0.4542 | 0.6609 | 0.0143 | 0.7247 | <0.0001 | <0.0001 | 0.0405 | 0.0118 | 0.0026 | 0.0194 |
| **CTLA4** | <0.0001 | 0.0004 | 0.7713 | 0.0702 | <0.0001 | 0.0059 | 0.0390 | 0.2794 | 0.9731 | 0.6065 | 0.6310 |
| **CD48** | 0.0003 | 0.1669 | 0.7754 | 0.0001 | 0.0010 | 0.0949 | 0.0016 | 0.9534 | 0.8656 | 0.0110 | 0.1562 |
| **CD28** | <0.0001 | 0.5838 | 0.4854 | 0.0551 | 0.0210 | <0.0001 | <0.0001 | 0.4610 | 0.0142 | 0.0005 | 0.3345 |
| **CD200R1** | 0.0021 | 0.2608 | 0.5840 | 0.7498 | 0.0221 | <0.0001 | 0.0002 | 0.2103 | 0.1863 | 0.0027 | 0.7275 |
| **HAVCR2** | 0.1250 | 0.0004 | 0.0023 | 0.0047 | <0.0001 | 0.0832 | <0.0001 | 0.3280 | 0.0373 | 0.7665 | 0.3206 |
| **ADORA2A** | 0.0073 | 0.2181 | 0.2243 | <0.0001 | 0.0058 | 0.0975 | 0.0033 | 0.6306 | 0.4177 | <0.0001 | 0.0639 |
| **CD276** | <0.0001 | 0.1057 | 0.5125 | <0.0001 | <0.0001 | <0.0001 | 0.8349 | <0.0001 | 0.9529 | <0.0001 | 0.1049 |
| **KIR3DL1** | 0.0040 | 0.2962 | 0.2634 | 0.5045 | 0.7760 | 0.0119 | 0.4355 | 0.0353 | 0.2315 | 0.2088 | 0.1594 |
| **CD80** | <0.0001 | 0.0041 | 0.0570 | 0.0007 | 0.0001 | 0.5383 | 0.0008 | 0.0841 | 0.4799 | 0.3571 | 0.8477 |
| **PDCD1** | <0.0001 | <0.0001 | 0.0067 | 0.0124 | <0.0001 | <0.0001 | 0.2053 | 0.0242 | 0.8779 | 0.6773 | 0.2392 |
| **LGALS9** | <0.0001 | 0.0874 | 0.2713 | 0.0001 | <0.0001 | 0.0273 | 0.0068 | 0.1787 | 0.0726 | <0.0001 | 0.2809 |
| **CD160** | 0.6613 | 0.2908 | 0.9976 | 0.0001 | 0.8654 | 0.0013 | 0.0592 | 0.8997 | 0.0370 | 0.0008 | 0.6206 |
| **TNFSF14** | <0.0001 | 0.0092 | 0.6972 | 0.0279 | 0.9121 | <0.0001 | <0.0001 | 0.0990 | 0.0002 | 0.3789 | 0.1531 |
| **IDO2** | 0.0030 | 0.0007 | 0.1093 | 0.4085 | 0.0006 | 0.3496 | 0.2827 | 0.4256 | 0.3023 | 0.1741 | 0.4162 |
| **ICOSLG** | 0.6477 | 0.3015 | 0.8940 | 0.6097 | 0.2483 | 0.8228 | 0.2423 | 0.9180 | 0.8645 | 0.0217 | 0.1558 |
| **TMIGD2** | <0.0001 | 0.1722 | 0.2757 | 0.1683 | 0.3231 | 0.0111 | 0.2079 | 0.2444 | 0.0493 | 0.4237 | 0.3141 |
| **VTCN1** | 0.1342 | 0.0188 | 0.9759 | <0.0001 | 0.0450 | 0.9415 | 0.0778 | 0.1469 | 0.9144 | 0.8808 | 0.5190 |
| **IDO1** | 0.0196 | <0.0001 | 0.3721 | <0.0001 | 0.0131 | <0.0001 | 0.2386 | 0.0414 | 0.4567 | 0.3130 | 0.6718 |
| **PDCD1LG2** | 0.0005 | 0.0418 | 0.0607 | 0.0042 | 0.0757 | 0.0008 | 0.0005 | 0.0594 | 0.0592 | 0.6803 | 0.3204 |
| **HHLA2** | <0.0001 | 0.0017 | 0.5108 | <0.0001 | 0.0481 | 0.0093 | <0.0001 | 0.0139 | 0.0020 | 0.1037 | 0.1060 |
| **TNFSF18** | 0.0694 | 0.7217 | 0.4040 | 0.0642 | 0.0426 | 0.2824 | 0.0686 | 0.0555 | 0.3868 | 0.0031 | 0.0033 |
| **BTNL2** | 0.6270 | 0.2817 | 0.0109 | 0.1378 | 0.0492 | 0.7373 | 0.9981 | 0.5901 | 0.8859 | 0.3535 | 0.3842 |
| **CD70** | <0.0001 | 0.0253 | 0.2139 | 0.0016 | <0.0001 | <0.0001 | 0.0120 | 0.0006 | 0.6609 | <0.0001 | 0.1500 |
| **TNFSF9** | <0.0001 | 0.0002 | 0.0676 | 0.0066 | <0.0001 | 0.4889 | 0.0086 | 0.0051 | 0.3165 | <0.0001 | 0.5159 |
| **TNFRSF8** | <0.0001 | <0.0001 | 0.0006 | 0.2953 | <0.0001 | 0.0019 | 0.0185 | 0.5054 | 0.2543 | 0.1530 | 0.6554 |
| **CD27** | <0.0001 | 0.0002 | 0.2663 | 0.0103 | <0.0001 | 0.2891 | <0.0001 | 0.2964 | 0.3073 | 0.2085 | 0.0974 |
| **TNFRSF25** | 0.0024 | 0.0030 | 0.0402 | 0.8814 | <0.0001 | <0.0001 | 0.0212 | 0.0020 | 0.2892 | 0.0002 | 0.6771 |
| **VSIR** | 0.0100 | 0.0900 | 0.0247 | <0.0001 | 0.0137 | 0.0011 | <0.0001 | <0.0001 | <0.0001 | 0.0029 | 0.1138 |
| **TNFRSF4** | 0.7258 | <0.0001 | 0.0209 | <0.0001 | <0.0001 | <0.0001 | 0.0071 | 0.0314 | 0.0501 | 0.1748 | 0.0169 |
| **CD40** | 0.0165 | 0.2982 | 0.9590 | 0.0009 | 0.2071 | 0.1086 | 0.7105 | 0.5917 | 0.2915 | 0.0277 | 0.0085 |
| **TNFRSF18** | <0.0001 | 0.0001 | 0.3913 | 0.1587 | <0.0001 | <0.0001 | 0.0438 | 0.1694 | 0.9112 | <0.0001 | 0.9181 |
| **TNFSF15** | 0.0275 | 0.2343 | 0.8599 | 0.1168 | <0.0001 | <0.0001 | 0.0220 | 0.2864 | <0.0001 | 0.0331 | 0.2348 |
| **TIGIT** | <0.0001 | <0.0001 | 0.4606 | 0.0004 | <0.0001 | 0.0206 | 0.0112 | 0.1363 | 0.4239 | 0.0973 | 0.1227 |
| **CD274** | 0.9794 | 0.1146 | 0.4313 | 0.9623 | 0.0041 | <0.0001 | 0.1158 | 0.3071 | 0.1166 | 0.1370 | <0.0001 |
| **CD86** | <0.0001 | 0.1667 | 0.3215 | 0.3477 | <0.0001 | 0.0777 | <0.0001 | 0.4146 | 0.0532 | 0.5026 | 0.5632 |
| **CD44** | <0.0001 | 0.0034 | 0.0011 | 0.3350 | 0.3215 | <0.0001 | <0.0001 | 0.4451 | 0.0165 | 0.0004 | 0.0156 |
| **TNFRSF9** | <0.0001 | <0.0001 | 0.2873 | 0.9831 | 0.0127 | <0.0001 | 0.0861 | 0.0084 | 0.9733 | 0.3328 | 0.2579 |
| **Cancer Type** | **PRAD** | **READ** | **SARC** | **SKCM** | **STAD** | **TGCT** | **THCA** | **THYM** | **UCEC** | **UCS** | **UVM** |
| **BTLA** | <0.0001 | 0.0002 | 0.3955 | 0.0080 | <0.0001 | <0.0001 | <0.0001 | 0.5272 | 0.9888 | 0.0868 | 0.6909 |
| **CD200** | 0.0032 | 0.0007 | 0.0090 | 0.0025 | <0.0001 | <0.0001 | 0.3516 | <0.0001 | 0.0346 | 0.1533 | 0.0056 |
| **TNFRSF14** | 0.0404 | 0.3603 | 0.0706 | 0.5726 | 0.0431 | <0.0001 | <0.0001 | 0.0536 | <0.0001 | 0.0868 | 0.1716 |
| **NRP1** | <0.0001 | <0.0001 | 0.6207 | 0.7142 | <0.0001 | <0.0001 | 0.6693 | <0.0001 | 0.0009 | 0.2361 | 0.9291 |
| **LAIR1** | 0.0031 | <0.0001 | 0.8661 | 0.0690 | 0.0017 | 0.0187 | <0.0001 | <0.0001 | 0.0088 | 0.0948 | 0.5919 |
| **TNFSF4** | 0.6955 | 0.0016 | 0.3891 | 0.5309 | 0.8225 | 0.8242 | <0.0001 | <0.0001 | 0.3606 | 0.4281 | 0.3506 |
| **CD244** | 0.9324 | 0.0007 | 0.7309 | 0.0009 | 0.5371 | 0.0112 | <0.0001 | <0.0001 | 0.2808 | 0.3762 | 0.1845 |
| **LAG3** | 0.1011 | 0.0736 | 0.0751 | 0.6199 | 0.0166 | 0.8759 | <0.0001 | 0.0134 | <0.0001 | 0.6479 | 0.6255 |
| **ICOS** | 0.0017 | 0.0362 | 0.4015 | 0.0432 | 0.6312 | 0.1250 | <0.0001 | <0.0001 | 0.4938 | 0.0785 | 0.6085 |
| **CD40LG** | 0.3401 | 0.0055 | 0.0003 | 0.0047 | <0.0001 | <0.0001 | <0.0001 | 0.0086 | <0.0001 | 0.1161 | 0.7141 |
| **CTLA4** | 0.0004 | 0.0108 | 0.1528 | 0.7830 | 0.0895 | 0.0791 | <0.0001 | 0.0024 | 0.0019 | 0.1039 | 0.4245 |
| **CD48** | 0.1195 | 0.0002 | 0.1194 | 0.0094 | <0.0001 | 0.0052 | <0.0001 | <0.0001 | 0.2625 | 0.8838 | 0.1614 |
| **CD28** | 0.2832 | <0.0001 | 0.4895 | 0.0003 | <0.0001 | <0.0001 | <0.0001 | <0.0001 | 0.0281 | 0.0607 | 0.8697 |
| **CD200R1** | 0.0400 | 0.0005 | 0.8222 | <0.0001 | 0.0001 | 0.0001 | <0.0001 | 0.0076 | 0.3193 | 0.8721 | 0.1457 |
| **HAVCR2** | <0.0001 | 0.0009 | 0.2310 | 0.2651 | 0.3408 | 0.2416 | <0.0001 | 0.0002 | 0.7724 | 0.0107 | 0.5573 |
| **ADORA2A** | 0.0231 | 0.1250 | 0.1500 | 0.1121 | 0.4454 | 0.0002 | <0.0001 | <0.0001 | 0.1611 | 0.9867 | 0.1118 |
| **CD276** | <0.0001 | 0.0685 | 0.0005 | 0.1293 | 0.0292 | 0.0022 | <0.0001 | <0.0001 | 0.0002 | 0.9910 | 0.0003 |
| **KIR3DL1** | 0.0389 | 0.1193 | 0.0313 | 0.4834 | 0.4101 | 0.8763 | <0.0001 | 0.0102 | 0.5472 | 0.3703 | 0.4428 |
| **CD80** | <0.0001 | 0.0112 | 0.0312 | 0.1620 | 0.3385 | 0.0639 | <0.0001 | 0.0014 | 0.1742 | 0.0442 | 0.9951 |
| **PDCD1** | 0.0004 | 0.0137 | 0.4050 | 0.2892 | 0.8077 | 0.2359 | <0.0001 | <0.0001 | 0.9462 | 0.1601 | 0.7960 |
| **LGALS9** | 0.0014 | 0.3922 | 0.9132 | 0.2786 | 0.4525 | 0.0110 | <0.0001 | <0.0001 | 0.0002 | 0.0254 | 0.6608 |
| **CD160** | 0.0097 | 0.0631 | 0.4109 | 0.0012 | 0.5841 | 0.0046 | <0.0001 | 0.0001 | 0.0601 | 0.1767 | 0.9685 |
| **TNFSF14** | 0.6131 | <0.0001 | 0.0635 | 0.0030 | 0.0006 | <0.0001 | <0.0001 | 0.0071 | <0.0001 | 0.0149 | 0.7678 |
| **IDO2** | 0.8432 | 0.0044 | 0.1555 | 0.0871 | 0.0193 | 0.2612 | 0.0003 | 0.7031 | 0.2155 | 0.1529 | 0.6602 |
| **ICOSLG** | 0.0064 | 0.1888 | 0.3883 | 0.4685 | 0.0698 | <0.0001 | 0.0024 | 0.0101 | <0.0001 | 0.4138 | 0.3371 |
| **TMIGD2** | 0.6905 | 0.1934 | 0.0909 | 0.5691 | 0.6879 | 0.0099 | <0.0001 | <0.0001 | 0.9270 | 0.3988 | 0.3605 |
| **VTCN1** | 0.0001 | 0.0004 | 0.3658 | 0.0298 | 0.0272 | 0.0104 | <0.0001 | 0.4173 | 0.2080 | 0.0015 | 0.1010 |
| **IDO1** | 0.0022 | 0.9969 | 0.4478 | 0.5480 | 0.0058 | 0.8154 | <0.0001 | 0.6055 | 0.6763 | 0.0017 | 0.7242 |
| **PDCD1LG2** | 0.7347 | 0.0002 | 0.6054 | 0.1210 | 0.0079 | 0.0014 | <0.0001 | 0.7376 | 0.1021 | 0.0097 | 0.0672 |
| **HHLA2** | <0.0001 | 0.0268 | 0.2652 | 0.4386 | 0.0906 | 0.0006 | 0.4684 | 0.0049 | <0.0001 | 0.4850 | 1.0000 |
| **TNFSF18** | <0.0001 | 0.0007 | 0.0542 | 0.0004 | 0.9447 | 0.6865 | <0.0001 | 0.0104 | 0.5868 | 0.1052 | 0.0493 |
| **BTNL2** | 0.6588 | 0.4545 | 0.0025 | 0.0035 | 0.2294 | 0.0011 | 0.2433 | 0.0009 | 0.0405 | 0.0342 | 0.0671 |
| **CD70** | <0.0001 | 0.2380 | <0.0001 | 0.2664 | 0.0770 | <0.0001 | <0.0001 | 0.1284 | 0.4307 | 0.0930 | 0.5608 |
| **TNFSF9** | 0.3066 | 0.0331 | <0.0001 | 0.8547 | 0.7030 | <0.0001 | <0.0001 | 0.0001 | 0.0016 | 0.0630 | 0.0098 |
| **TNFRSF8** | <0.0001 | 0.0026 | 0.6454 | 0.0875 | 0.5688 | <0.0001 | <0.0001 | 0.7473 | 0.1884 | 0.0716 | 0.0935 |
| **CD27** | 0.0017 | 0.0009 | 0.8569 | 0.0427 | 0.0038 | 0.0096 | <0.0001 | <0.0001 | 0.0040 | 0.0939 | 0.8929 |
| **TNFRSF25** | 0.1533 | 0.0026 | 0.1566 | 0.2224 | <0.0001 | 0.0013 | <0.0001 | 0.0756 | 0.0551 | 0.1883 | 0.3653 |
| **VSIR** | <0.0001 | <0.0001 | 0.0150 | 0.0001 | <0.0001 | <0.0001 | 0.5822 | <0.0001 | 0.0001 | 0.0006 | 0.8194 |
| **TNFRSF4** | <0.0001 | 0.3527 | 0.7532 | 0.3935 | 0.0028 | 0.0250 | <0.0001 | 0.3617 | 0.2865 | 0.1295 | 0.3492 |
| **CD40** | <0.0001 | 0.6999 | 0.1570 | 0.8833 | 0.2121 | 0.0024 | <0.0001 | <0.0001 | <0.0001 | 0.3097 | 0.0002 |
| **TNFRSF18** | <0.0001 | 0.1605 | 0.3508 | 0.9417 | <0.0001 | 0.7870 | <0.0001 | 0.1485 | 0.6838 | 0.3175 | 0.4308 |
| **TNFSF15** | 0.1869 | 0.1812 | 0.1630 | 0.8137 | 0.7416 | <0.0001 | 0.0352 | 0.0221 | <0.0001 | 0.1090 | 0.8035 |
| **TIGIT** | 0.0001 | 0.0003 | 0.5099 | 0.0639 | 0.1223 | 0.1885 | <0.0001 | 0.0005 | 0.3540 | 0.1278 | 0.7120 |
| **CD274** | 0.3890 | 0.0204 | 0.3141 | 0.1396 | 0.2814 | 0.0168 | <0.0001 | 0.0223 | 0.4950 | 0.0045 | 0.0577 |
| **CD86** | 0.0002 | 0.0004 | 0.4021 | 0.0613 | 0.2825 | 0.0678 | <0.0001 | <0.0001 | 0.8551 | 0.0785 | 0.5927 |
| **CD44** | <0.0001 | 0.1483 | 0.0315 | <0.0001 | <0.0001 | <0.0001 | 0.2304 | 0.6534 | <0.0001 | 0.0011 | 0.0007 |
| **TNFRSF9** | <0.0001 | 0.0113 | 0.0250 | 0.1754 | 0.4692 | 0.4188 | <0.0001 | 0.0025 | 0.9308 | 0.3115 | 0.9463 |
