# Supplementary material for: Pan-Cancer Analyses of the Tumor Microenvironment Reveal That Ubiquitin-Conjugating Enzyme E2C Might Be a Potential Immunotherapy Target
Source: J Immunol Res. 2021 Dec 13;2021:9250207. doi: 10.1155/2021/9250207 (PMC8689232; doi:10.1155/2021/9250207)
Supplement: Supplementary 2 — Supplementary Table1: the p value of differentially expressed analysis in Figure 1(b). Supplementary Table2: the Spearman's correlation coefficient between UBE2C and immune markers. Supplementary Table3: the p value of correlation analysis between UBE2C and immune markers. Supplementary Table4: the p value between UBE2C expression and 22 immune cells correlation analysis. [file 9250207.f2.zip › Supplementary Table4.doc]

| **Supplementary Table4 The p-value between UBE2C expression and 22 immune cells correlation analysis** | | | | | | | | | | | |
| --- | --- | --- | --- | --- | --- | --- | --- | --- | --- | --- | --- |
| **Cancer Type** | **ACC** | **BLCA** | **BRCA** | **CESC** | **CHOL** | **COAD** | **DLBC** | **ESCA** | **GBM** | **HNSC** | **KICH** |
| **B cells  naive** | 0.0350 | 0.0293 | 0.0001 | 0.7037 | 0.5005 | 0.3507 | 0.9581 | 0.5703 | 0.3148 | 0.2106 | 0.2471 |
| **B cells  memory** | 0.5806 | 0.3895 | 0.0951 | 0.7103 | 0.0247 | 0.8468 | 0.3013 | 0.3249 | 0.7850 | 0.0968 | 1.0000 |
| **Plasma  cells** | 0.9539 | 0.1718 | 0.7948 | 0.9707 | 0.3645 | 0.0459 | 1.0000 | 0.3447 | 0.6903 | 0.9832 | 0.4987 |
| **T cells  CD8** | 0.2610 | 0.0001 | 0.0002 | 0.0847 | 0.1436 | 0.9619 | 0.9294 | 0.8016 | 0.4751 | 0.0301 | 0.9406 |
| **T cells  CD4 naive** | 1.0000 | 1.0000 | 1.0000 | 1.0000 | 1.0000 | 1.0000 | 1.0000 | 1.0000 | 1.0000 | 1.0000 | 1.0000 |
| **T cells CD4 memory resting** | 0.0838 | <0.0001 | <0.0001 | 0.2015 | 0.0022 | 0.0166 | 0.8179 | 0.4933 | 0.0016 | 0.0389 | 0.2018 |
| **T cells CD4 memory activated** | 0.0396 | 0.0007 | <0.0001 | 0.2339 | 0.2943 | <0.0001 | 0.3406 | 0.3894 | 0.8910 | 0.0091 | 1.0000 |
| **T cells follicular helper** | 0.0152 | 0.0408 | <0.0001 | 0.8980 | 0.6235 | 0.2214 | 0.8251 | 0.0168 | 0.0090 | 0.1155 | 0.0467 |
| **T cells regulatory (Tregs)** | 0.8551 | 0.0009 | 0.0004 | 0.1849 | 0.9129 | 0.0166 | 0.0001 | 0.7781 | 1.0000 | 0.2425 | 0.5515 |
| **T cells gamma delta** | 0.0030 | 0.6339 | 0.8818 | 0.7164 | 1.0000 | 1.0000 | 0.3663 | 1.0000 | 0.9653 | 1.0000 | 0.7608 |
| **NK cells resting** | 0.0311 | 0.0956 | 1.0000 | 0.2306 | 1.0000 | 0.2175 | 1.0000 | 0.2241 | 0.7482 | 0.0248 | 0.0846 |
| **NK cells activated** | 0.1268 | 0.0635 | <0.0001 | 0.8007 | 0.3784 | 0.0490 | 0.2637 | 0.0325 | 0.1006 | 0.9479 | 0.0762 |
| **Monocytes** | 0.7812 | 0.4948 | <0.0001 | 0.0264 | 0.1298 | 0.2853 | 0.2958 | 0.1091 | <0.0001 | 0.0227 | 0.0153 |
| **Macrophages M0** | 0.0011 | <0.0001 | <0.0001 | 0.2484 | 0.1897 | 0.1187 | 0.4838 | 0.0353 | 0.0752 | 0.9972 | 0.2408 |
| **Macrophages M1** | 0.2424 | 0.0004 | <0.0001 | 0.5909 | 0.9120 | 0.6139 | 0.4017 | 0.8902 | 0.1959 | 0.0491 | 0.1630 |
| **Macrophages M2** | 0.8286 | 0.0554 | <0.0001 | 0.8851 | 0.9919 | 0.0517 | 0.6098 | 0.1527 | <0.0001 | 0.1205 | 0.9314 |
| **Dendritic cells resting** | 0.0383 | 0.0097 | <0.0001 | 0.0369 | 0.7824 | 0.9633 | 0.5890 | 0.0508 | 0.1185 | <0.0001 | 0.3413 |
| **Dendritic cells activated** | 0.3675 | 0.6545 | <0.0001 | 0.4134 | 0.8550 | 0.6642 | 0.1437 | 0.3035 | 1.0000 | 0.4168 | 0.9125 |
| **Mast cells resting** | 0.0019 | <0.0001 | <0.0001 | 0.0980 | 0.4294 | 0.2051 | 0.1412 | 0.0001 | 0.1650 | <0.0001 | 0.1410 |
| **Mast cells activated** | 0.2396 | 0.4358 | 1.0000 | 0.3735 | 0.9249 | 0.1185 | 1.0000 | 0.1149 | 0.9880 | 0.0726 | 1.0000 |
| **Eosinophils** | 0.1444 | 0.5401 | 1.0000 | 1.0000 | 1.0000 | 0.9947 | 1.0000 | 1.0000 | 0.0062 | 1.0000 | 0.6718 |
| **Neutrophils** | 0.6436 | 0.8545 | 0.0657 | 0.0185 | 0.5101 | 0.3402 | 1.0000 | 0.6703 | 0.0881 | 0.0003 | 0.6493 |
| **Cancer Type** | **KIRC** | **KIRP** | **LAML** | **LGG** | **LIHC** | **LUAD** | **LUSC** | **MESO** | **OV** | **PAAD** | **PCPG** |
| **B cells  naive** | 0.0072 | 0.8966 | 0.5570 | 0.6124 | 0.0010 | 0.2659 | 0.8793 | 0.0674 | 0.1848 | <0.0001 | 0.2438 |
| **B cells  memory** | 0.1017 | 0.2769 | 0.6277 | 0.7361 | 1.0000 | 0.7158 | 0.1046 | 0.3048 | 0.3859 | <0.0001 | 0.1682 |
| **Plasma  cells** | 0.0038 | 0.2760 | 0.0011 | 1.0000 | 0.0930 | 0.7557 | 0.0029 | 0.0056 | 0.3984 | 0.0389 | 0.0785 |
| **T cells  CD8** | <0.0001 | 0.1788 | 0.3471 | 0.8774 | 0.0707 | <0.0001 | <0.0001 | 0.2348 | 0.0021 | 0.0015 | 0.3325 |
| **T cells  CD4 naive** | 1.0000 | 1.0000 | 0.4255 | 1.0000 | 1.0000 | 1.0000 | 1.0000 | 1.0000 | 1.0000 | 1.0000 | 1.0000 |
| **T cells CD4 memory resting** | <0.0001 | 0.0715 | 0.0587 | 0.0030 | 0.0016 | <0.0001 | <0.0001 | 0.7227 | <0.0001 | 0.6094 | 0.9292 |
| **T cells CD4 memory activated** | <0.0001 | <0.0001 | 0.5978 | 1.0000 | <0.0001 | <0.0001 | 0.0818 | 0.6782 | 0.0189 | 0.0609 | 0.0200 |
| **T cells follicular helper** | 0.0002 | <0.0001 | 1.0000 | 0.0787 | <0.0001 | 0.0013 | <0.0001 | 0.4618 | <0.0001 | 0.0034 | 0.3753 |
| **T cells regulatory (Tregs)** | <0.0001 | 0.0314 | 1.0000 | 1.0000 | 0.1494 | 0.9341 | 0.4103 | 0.0042 | 0.5262 | 0.7463 | 0.5515 |
| **T cells gamma delta** | 0.7440 | 1.0000 | 0.9435 | 1.0000 | 0.0417 | 0.7115 | 1.0000 | 1.0000 | 0.5127 | 1.0000 | 1.0000 |
| **NK cells resting** | <0.0001 | 0.6903 | 0.2856 | 0.5351 | 0.1019 | 1.0000 | 0.5221 | 1.0000 | 1.0000 | 0.6477 | 0.0058 |
| **NK cells activated** | 0.0013 | 0.0001 | 0.7715 | 0.4815 | 0.4245 | 0.0017 | 0.0002 | 0.9006 | 0.6168 | 0.0040 | 0.0370 |
| **Monocytes** | 0.0002 | 0.0510 | 0.5278 | 0.0051 | 0.0253 | 0.0046 | 0.0073 | 0.2330 | 0.1319 | 0.0007 | 0.6698 |
| **Macrophages M0** | <0.0001 | 0.0001 | 1.0000 | 0.0023 | 0.0006 | <0.0001 | 0.0137 | 0.1993 | 0.0086 | <0.0001 | 0.0029 |
| **Macrophages M1** | 0.6952 | <0.0001 | 1.0000 | 0.0001 | 0.7442 | <0.0001 | <0.0001 | 0.0177 | 0.1270 | 0.0465 | 0.5070 |
| **Macrophages M2** | <0.0001 | <0.0001 | 0.0072 | 0.3389 | <0.0001 | 0.0613 | 0.5639 | 0.0873 | 0.2182 | 0.5041 | 0.1788 |
| **Dendritic cells resting** | 0.0068 | 0.2466 | 1.0000 | 0.1815 | 0.0108 | <0.0001 | 0.0004 | 0.0451 | 0.0221 | 0.0227 | 0.8418 |
| **Dendritic cells activated** | 0.0002 | 0.5292 | 1.0000 | 1.0000 | 0.5172 | 0.0287 | 0.0267 | 0.0863 | 0.4975 | 0.0110 | 0.5074 |
| **Mast cells resting** | <0.0001 | 0.4374 | 0.1822 | 0.4489 | 0.1683 | <0.0001 | 0.0001 | 0.0426 | 0.0061 | 0.0213 | 0.0283 |
| **Mast cells activated** | 1.0000 | 1.0000 | 1.0000 | 0.0003 | 1.0000 | 1.0000 | 0.9787 | 1.0000 | 0.3964 | 1.0000 | 0.0674 |
| **Eosinophils** | 1.0000 | 1.0000 | 0.5710 | 0.4131 | 0.2115 | 0.8355 | 0.2070 | 1.0000 | 1.0000 | 1.0000 | 0.3322 |
| **Neutrophils** | 0.0524 | 0.1047 | 0.7370 | 0.4844 | 0.3599 | 0.9491 | 0.0235 | 0.7473 | 0.0409 | 0.3275 | 0.3591 |
| **Cancer Type** | **PRAD** | **READ** | **SARC** | **SKCM** | **STAD** | **TGCT** | **THCA** | **THYM** | **UCEC** | **UCS** | **UVM** |
| **B cells  naive** | 0.3613 | 0.3459 | 0.0001 | 0.2130 | <0.0001 | 0.0321 | 0.0432 | 0.2243 | 0.6120 | 0.3827 | 0.0253 |
| **B cells  memory** | 0.0431 | 0.1997 | 1.0000 | 0.9937 | 0.5940 | 0.0383 | 0.2501 | 0.0027 | 0.0427 | 0.5799 | 1.0000 |
| **Plasma  cells** | 0.0259 | 0.9403 | 0.1249 | 0.2560 | 0.8366 | 0.3145 | 0.0022 | 0.6324 | 0.2552 | 0.7864 | 0.0060 |
| **T cells  CD8** | 0.9585 | 0.9257 | 0.8313 | 0.3931 | 0.2268 | 0.0089 | 0.2238 | 0.1225 | 0.1209 | 0.1742 | 0.2622 |
| **T cells  CD4 naive** | 1.0000 | 1.0000 | 1.0000 | 1.0000 | 1.0000 | 1.0000 | 1.0000 | <0.0001 | 1.0000 | 1.0000 | 1.0000 |
| **T cells CD4 memory resting** | <0.0001 | 0.7829 | 0.0038 | 0.0049 | 0.0001 | <0.0001 | 0.0004 | 0.1239 | 0.0001 | 0.4614 | 0.7725 |
| **T cells CD4 memory activated** | 0.1122 | 0.0288 | 0.0047 | 0.1881 | <0.0001 | 0.0402 | <0.0001 | 0.7401 | <0.0001 | 0.0388 | 0.3862 |
| **T cells follicular helper** | 0.8275 | 0.0355 | 0.0201 | 0.1785 | <0.0001 | 0.0005 | <0.0001 | 0.0173 | <0.0001 | 0.9886 | 0.8485 |
| **T cells regulatory (Tregs)** | 0.0005 | 0.1082 | 0.1114 | 0.9441 | 0.2339 | 0.0148 | 0.0059 | 0.0255 | <0.0001 | 0.0119 | 0.6565 |
| **T cells gamma delta** | 1.0000 | 1.0000 | 0.0169 | 0.0067 | 1.0000 | 1.0000 | 0.8627 | 0.0017 | 0.2680 | 0.1336 | 0.6818 |
| **NK cells resting** | 1.0000 | 0.2897 | 1.0000 | 0.1152 | 0.3945 | 1.0000 | 0.0039 | <0.0001 | 0.5235 | 0.6091 | 0.0072 |
| **NK cells activated** | 0.2602 | 0.0707 | 0.9078 | 0.3565 | 0.0028 | <0.0001 | 0.7820 | <0.0001 | 0.4227 | 0.5015 | 0.3397 |
| **Monocytes** | 0.5596 | 0.5977 | 0.1541 | 0.7556 | 0.0547 | 0.0329 | 0.5722 | 0.0089 | 0.6102 | 0.9679 | 0.6602 |
| **Macrophages M0** | 0.1565 | 0.1402 | <0.0001 | 0.0007 | <0.0001 | 0.6379 | 0.0265 | <0.0001 | 0.8153 | 0.2782 | 0.0271 |
| **Macrophages M1** | 0.0176 | 0.2004 | 0.2705 | 0.4026 | <0.0001 | 0.1944 | <0.0001 | <0.0001 | <0.0001 | 0.5989 | 0.9207 |
| **Macrophages M2** | 0.0002 | 0.1808 | 0.0503 | 0.3827 | 0.4398 | 0.0726 | <0.0001 | <0.0001 | 0.2128 | 0.7572 | 0.8462 |
| **Dendritic cells resting** | 0.7093 | 0.0959 | 0.1474 | 0.4554 | 0.0779 | 0.4394 | 0.0265 | <0.0001 | <0.0001 | 0.1117 | 0.1114 |
| **Dendritic cells activated** | 0.7920 | 0.0482 | 0.6048 | 0.8313 | 0.4908 | 0.0151 | 0.5426 | 0.0001 | 0.0580 | 0.0053 | 1.0000 |
| **Mast cells resting** | 0.0173 | 0.9244 | <0.0001 | 0.0530 | <0.0001 | 0.7086 | 0.0529 | <0.0001 | 0.4673 | 0.2808 | 0.6115 |
| **Mast cells activated** | 1.0000 | 0.1874 | 0.3054 | 0.8455 | 0.1333 | 1.0000 | 1.0000 | 1.0000 | 0.2930 | 0.2299 | 1.0000 |
| **Eosinophils** | 1.0000 | 0.0147 | 1.0000 | 1.0000 | 0.1591 | 1.0000 | 1.0000 | 1.0000 | 1.0000 | 0.8303 | 1.0000 |
| **Neutrophils** | 1.0000 | 0.3907 | 1.0000 | 0.6829 | 0.0365 | 1.0000 | 1.0000 | 1.0000 | 0.0001 | 1.0000 | 1.0000 |
